# Asymmetric synthesis of *syn*-propargylamines and unsaturated β-amino acids under Brønsted base catalysis

**DOI:** 10.1038/ncomms9544

**Published:** 2015-10-01

**Authors:** Yingcheng Wang, Mingjie Mo, Kongxi Zhu, Chao Zheng, Hongbin Zhang, Wei Wang, Zhihui Shao

**Affiliations:** 1 Key Laboratory of Medicinal Chemistry for Natural Resource, Ministry of Education, School of Chemical Science and Technology, Yunnan University, Kunming 650091, China; 2 State Key Laboratory of Organometallic Chemistry, Shanghai Institute of Organic Chemistry, Chinese Academy of Sciences, Shanghai 200032, China; 3 Department of Chemistry & Chemical Biology, University of New Mexico, Albuquerque, New Mexico 87131, USA

## Abstract

Propargylamines are important intermediates for the synthesis of polyfunctional amino derivatives and natural products and biologically active compounds. The classic method of synthesizing chiral propargylamines involves the asymmetric alkynylation of imines. Here, we report a significant advance in the catalytic asymmetric Mannich-type synthesis of propargylamines through catalytic asymmetric addition of carbon nucleophiles to C-alkynyl imines, culminating in a highly *syn*-selective catalytic asymmetric Mannich reaction of C-alkynyl imines that provide *syn*-configured propargylamines with two adjacent stereogenic centres and a transition metal-free organocatalytic asymmetric approach to β-alkynyl-β-amino acids with high efficiency and practicality, via a chiral Brønsted base-catalysed asymmetric Mannich-type reaction of *in situ* generated challenging *N*-Boc C-alkynyl imines from previously unreported C-alkynyl *N*-Boc-*N*,O-acetals, with α-substituted β-keto esters and less-acidic malonate (thio)esters as nucleophiles, respectively. A catalytic activation strategy is also disclosed, which may have broad implications for use in catalysis and synthesis.

## Abstract (web summary)

Propargylamines are of interest both as biologically active compounds and versatile synthetic intermediates. Here, the authors report a method for the organocatalytic asymmetric synthesis of propargylamines through addition of carbon nucleophiles to alkynyl imines.

## Graphical abstract

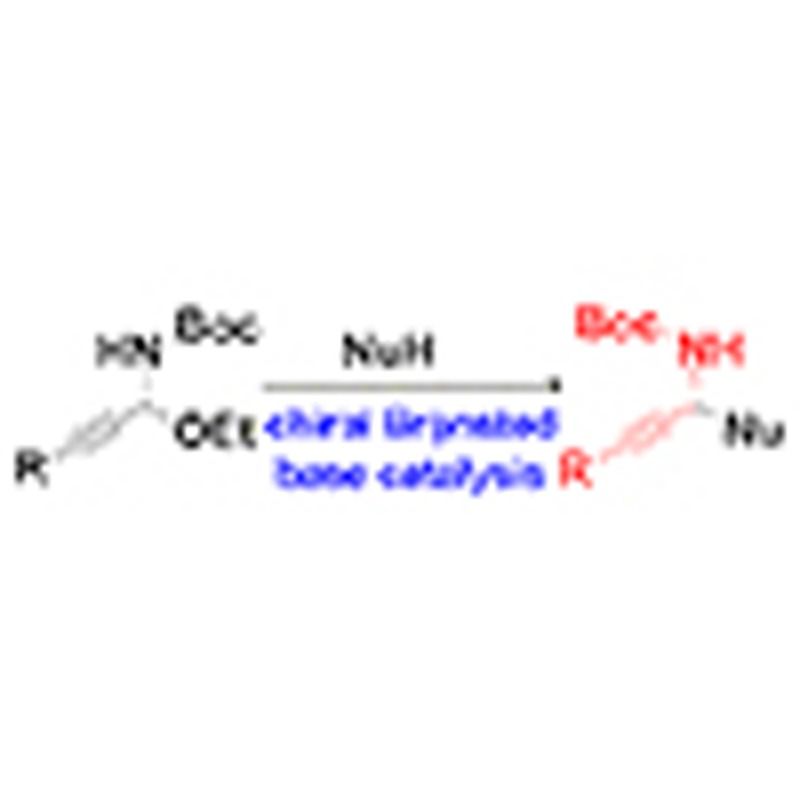

Propargylamines are important intermediates for the synthesis of polyfunctional amino derivatives as well as natural products and biologically active compounds^1^,^2^,^3^ due to the rich chemistry associated with the alkynyl group^4^. Consequently, significant efforts have been devoted to develop methodologies for preparing this important class of compounds in enantiomerically enriched form. Of these catalytic asymmetric approaches, the alkynylation of imines and the addition of carbon nucleophiles to C-alkynyl imines are particularly attractive (Fig. 1), because C–C bond formation and stereocenter creation occur simultaneously. The catalytic asymmetric alkynylation of imines, thereby creating one new stereogenic centre in the bond-forming reaction, has been studied extensively (Fig. 1a)^5^,^6^,^7^. In contrast, the catalytic asymmetric addition of carbon nucleophiles to C-alkynyl imines has been underdeveloped (Fig. 1b)^8^,^9^,^10^,^11^,^12^,^13^,^14^,^15^, and the reactions with prochiral nucleophiles to generate chiral propargylamines with two adjacent stereogenic centres are few^11^,^12^,^13^. Interestingly, these limited reports provide *anti*-configured propargylamine Mannich products^11^,^12^,^13^, and a *syn*-selective catalytic asymmetric Mannich reaction of C-alkynyl imines remains unattainable, despite the high synthetic utility. Thus, we became interested in developing the catalytic asymmetric additions of carbon nucleophiles to C-alkynyl imines to target *syn*-configured chiral propargylamines and pharmaceutically and synthetically important chiral propargylamines, such as β-alkynyl-β-amino acids, which cannot be prepared by the frequently employed alkynylation of imines.

β-Amino acids are key structural elements of peptides, peptidomimetics, pharmaceuticals and natural products^16^. β-Amino acids are also essential building blocks for the synthesis of pharmaceutical targets, natural products and peptidic materials with unique structural properties. Among the various β-amino acids, β-alkynyl-β-amino acids represent a particularly intriguing subclass of compounds. It is now recognized that β-ethynyl-substituted amino acids can not only remarkably change the biological properties of some natural amino acids, but are also key intermediates of certain designed drugs, such as Xemilofiban and SC-54701, which are platelet aggregation inhibitors that can prevent ischaemia, heart attacks and other major adverse cardiac events^17^,^18^,^19^,^20^. Very few methods exist, however, for the catalytic enantioselective synthesis of chiral β-alkynyl-β-amino acids and derivatives^10^. In 2005, Snapper and Hoveyda^10^ reported an elegant approach to aryl-protected chiral β-alkynyl-β-amino esters. However, this protocol that utilizes chiral silver Lewis acid catalyst, is restricted to pre-formed imine substrates bearing *N*-aryl substituents with a pendant chelating group for two-point binding to the catalyst. This requirement imposes several practical limitations, including the need for strong oxidative or reductive conditions for product amine deprotection. At the same time, this protocol requires activated silyl enolate as nucleophile^10^. Therefore, the development of new catalytic asymmetric methods for the efficient preparation of β-alkynyl-β-amino acids and derivatives is highly demanded, but remains a significant challenge. In this context, in contrast to chiral silver Lewis acid catalysis approach^10^, we sought to develop a transition metal-free, chiral organobase-catalysed asymmetric approach to Boc (*tert*-butoxycarbonyl)-protected chiral β-alkynyl-β-amino acids and derivatives. In particular, we became interested in the possibility of a direct, transition metal-free, mild chiral organobase-catalysed asymmetric Mannich-type reaction of *in situ* generated *N*-Boc-protected C-alkynyl imines with malonate (thio)esters as nucleophiles, as an efficient approach to Boc-protected chiral β-alkynyl-β-amino acids and derivatives in that the necessity of imine preparation and carbonyl substrate pre-activation in the form of a silyl enolate in a separate operation would be obviated. At the same time, such an approach could also significantly benefit from easy removal and handling of the *N*-Boc protecting group^21^ and advantages of organocatalysis^22^. However, this task has proved to be a formidable challenge.

Herein, we report the realization of such a significant challenge allowing a unified synthesis of three different β-amino acid structural types, β-alkynyl-β-amino acids, β-alkenyl-β-amino acids^23^ and β-alkyl-β-amino acids, with the D and L configurations, as well as the achievement of a highly *syn*-selective catalytic asymmetric Mannich reaction of C-alkynyl imines providing *syn*-propargylamines with two adjacent stereogenic centres via the development of a synergistic catalytic activation strategy.

## Results

### Synthesis of chiral Boc-protected β-alkynyl β-amino acids

We initially explored the classic method for *in situ* generation of *N*-Boc-protected aryl and alkyl imines from the corresponding *N*-Boc amino sulfone precursor^24^ in our proposed organobase catalysed asymmetric Mannich-type of *in situ* generated *N*-Boc-protected C-alkynyl imines to synthesize Boc-protected β-alkynyl-β-amino acids and derivatives. However, we found that traditional methods^24^ were not effective for the generation of the C-alkynyl *N*-Boc amino sulfone precursor.

**Figure 2:**
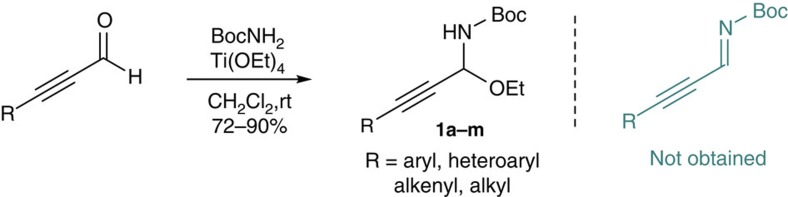
Synthesis of C-alkynyl *N*-Boc-*N*,*O*-acetals 1. Condition: ynal (10 mmol), BocNH_2_ (15 mmol), Ti(OEt)_4_ (15 mmol), dichloromethane (DCM) (80 ml), rt, 36 h; Boc, *t*-butoxycarbonyl.

Next, various reported synthetic methods to *N*-Boc aryl and alkyl imines were examined to access *N*-Boc C-alkynyl imines. However, despite extensive efforts, we still could not obtain *N*-Boc C-alkynyl imines perhaps due to their poor stability. Interestingly, our attempt to prepare *N*-Boc C-alkynyl imine through the condensation of 3-phenylpropiolaldehyde and BocNH_2_ in the presence of titanium ethoxide^25^,^26^,^27^ unexpectedly led to the formation of C-alkynyl *N*-Boc-*N*,O-acetal **1a** (R=Ph; Fig. 2). Furthermore, various C-alkynyl *N*-Boc-*N*,*O*-acetals **1a**–**m** could be readily obtained as an air-, water- and light-stable solid under mild conditions. Our examination of the literature revealed that this class of *N*,O-acetals were not reported previously. Thus, given the difficulty and importance of generating *N*-Boc C-alkynyl imines, we questioned whether these new C-alkynyl *N*-Boc-*N*,*O*-acetals could be used for a direct, chiral base-catalysed asymmetric Mannich-type reaction of *in situ-*generated *N*-Boc C-alkynyl imines with malonate (thio)esters to synthesize Boc-protected β-alkynyl-β-amino acids and derivatives.

The reaction of C-alkynyl *N*,*O*-acetal **1a** (R=Ph) with *S*,*O*-malonate **2a** was initially examined in the presence of achiral bases. We were delighted to discover that in the presence of bases such as NaH, *t*-BuOK and NaHMDS (sodium hexamethyldisilazide), the Mannich-type reaction of **1a** and **2a** proceeded smoothly to give product *rac*-**3a** in high yield (Table 1, entries 1−3).

To investigate the nature of C-alkynyl *N*-Boc-*N*,*O-*acetal as a new imine precursor in base-mediated *in situ* generation of *N*-Boc C-alkynyl imine, the following experiments were conducted. When a mixture of **1a** and CD_3_CD_2_OD was stirred in the presence of NaH, the corresponding deuterium labelling **1a′** formed (see Supplementary Fig. 1). When **1a** was stirred in the presence of NaH and the reaction was monitored by ^1^H-NMR, the imine itself was not observed. These experimental results suggested that elimination of EtOH is reversible and also only a trace amount of C-alkynyl *N*-Boc imine is generated by a base promoter (Supplementary Fig. 1).

The base-mediated protocol is particularly attractive because it provides a rare opportunity to develop the corresponding catalytic asymmetric variant. However, the realization of such a catalytic asymmetric variant is quite challenging as it requires that a chiral base catalyst must be capable of not only catalysing the *in situ* generation of challenging *N*-Boc C-alkynyl imines from C-alkynyl *N*-Boc-*N*,*O*-acetals but also promoting the subsequent asymmetric nucleophilic addition with malonate (thio)esters. In addition, the elimination byproduct EtOH could also have a deleterious effect on the reactivity and/or stereoselectivity by a competing hydrogen bonding. Our examination of the literature revealed no precedents of chiral base-catalysed generation of *N*-Boc imines from *N*-Boc-*N*,*O*-acetals^28^. Despite these challenges, chiral bases, (DHQD)_2_PHAL (hydroquinidine 1,4-phthalazinediyl diether)^29^, quinine and cinchonidine, were preliminarily tested in the model reaction of **1a** and **2a**. Unfortunately, the reaction did not happen (Table 2, entries 1−3).

**Figure 3:**
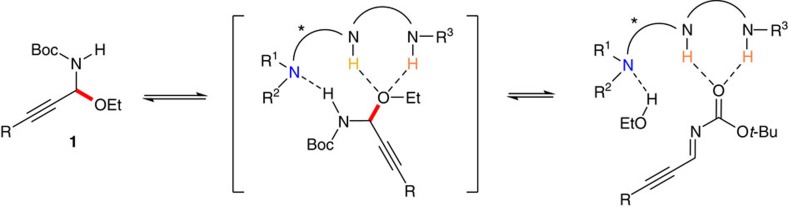
Catalytic activation mode devised in this study. Tertiary amine-catalysed *in situ* generation of *N*-Boc C-alkynyl imines via hydrogen bond activation was shown.

There are several challenges that still needed to be addressed. The basicity of the tertiary amine Brønsted base is weak, thus unlike strong bases such as NaH, *t*-BuOK and NaHMDS, the tertiary amine Brønsted base itself could not deprotonate the proton of BocNH of **1** to generate *N*-Boc C-alkynyl imines with the elimination of EtOH. On the other hand, the cleavage of the C–O bond of *N*,*O*-acetals **1** (the red bond highlighted in Fig. 3) under basic conditions generally is not favoured than under acidic conditions. With these considerations in mind, we hypothesized that the mild non-covalent hydrogen bond could simply promote the cleavage of the C–O bond of *N*,*O*-acetals and also stabilize the resulting unstable *N*-Boc C-alkynyl imines. Thus, a mild chiral bifunctional tertiary amine Brønsted base-catalysed *in situ* generation of challenging *N*-Boc C-alkynyl imines via hydrogen bond activation was devised (Fig. 3). To the best of our knowledge, this activation mode has never been demonstrated, despite its high potential.

To test our hypothesis, several bifunctional tertiary amine/hydrogen bond donor catalysts were tested for the direct catalytic asymmetric Mannich-type of **1a** and **2a**. In the presence of catalyst **A**, developed in our laboratory^30^, the reaction proceeded smoothly to provide the desired product **3a** in good yield (82%) with high enantioselectivity (95.5:4.5 er; Table 2, entry 4). Interestingly, catalyst **B**, a diastereoisomer of the catalyst **A**, gave poor yield and enantiocontrol (entry 5), thus indicating the remarkable influence of tertiary amine Brønsted base moiety. Catalyst **C**^31^,^32^,^33^,^34^ afforded **3a** in 80% yield with 97:3 er (entry 6). Commercially available Takemoto's catalyst **D**^35^ also worked well for this tandem reaction (entry 7), indicating the practicality of this strategy. Furthermore, Rawal's catalyst **F**^36^ bearing a squaramide group was also effective to deliver the product in high efficiency (entry 10). It is noteworthy that only a small excess (1.2 equivalents) of the *S*,*O*-malonate was necessary to obtain these results. Due to easy epimerization at the α-stereogenic centre under the reaction conditions, the product **3a** obtained was an around 1:1 diastereomeric mixture.

To better understand our devised activation mode, tertiary amine **G** that lacks a hydrogen bond donor and thiourea **H** without a Brønsted base functionality were examined. The reaction did not occur and the substrate **1a** was recovered. Furthermore, only a trace amount of **3a** was obtained with **G**+**H** (Supplementary Fig. 2). These results and DFT (density functional theory) calculations (Supplementary Fig. 3) support our working hypothesis shown in Fig. 3 that both the Brønsted base functionality and the hydrogen bond donor are essential for synergistic activation of *N*-Boc-*N*,*O*-acetals in this enantioselective tandem process. The chiral bifunctional tertiary amine/thiourea or tertiary amine/squaramide catalyst played multiple roles—cooperatively activating C-alkynyl *N*-Boc-*N*,*O*-acetal electrophile to generate less stable *N*-Boc C-alkynyl imine and promoting the subsequent enantioselective Mannich-type addition by synergistic activation of C-alkynyl imine electrophile and *S*,*O*-malonate nucleophile. It is noteworthy that while chiral tertiary amine/thioureas and tertiary amine/squaramides have been identified as effective catalysts for numerous transformations^37^,^38^, there is no report of using such catalysts for the generation of imines from *N*,*O*-acetals^28^. Nor is there any report of tertiary amine/thiourea or tertiary amine/squaramide catalysed asymmetric Mannich-type reactions involving C-alkynyl imines or directly from *N*-Boc-*N*,*O*-acetals.

This strategy could be expanded to a wide range of aryl-, vinyl- and alkyl- substituted C-alkynyl *N*-Boc-*N*,*O*-acetals (Table 3). Interestingly, highly electron-deficient, *p*-CF_3_-substituted arylalkynyl substrate did not work even at higher temperature. Fortunately, by using the catalyst **A** developed in our lab^30^, this problematic substrate was found to react well with **2a**, giving the desired Mannich product in good yield and enantioselectivity (entry 8). A heteroaryl-substituted alkynyl substrate was also suitable for this tandem reaction (entry 9). There is no report of a catalytic asymmetric Mannich-type reaction involving heteroarylalkynyl-substituted imines. C-alkynyl *N*,*O*-acetal **1n** protected by the commonly used benzyloxycarbonyl (Cbz) group^21^ instead of Boc group was also applicable to the reaction with *S*,*O*-malonate **2a** (entry 14), thus providing orthogonal sets of easily removable *N*-protecting groups.

**Figure 4:**
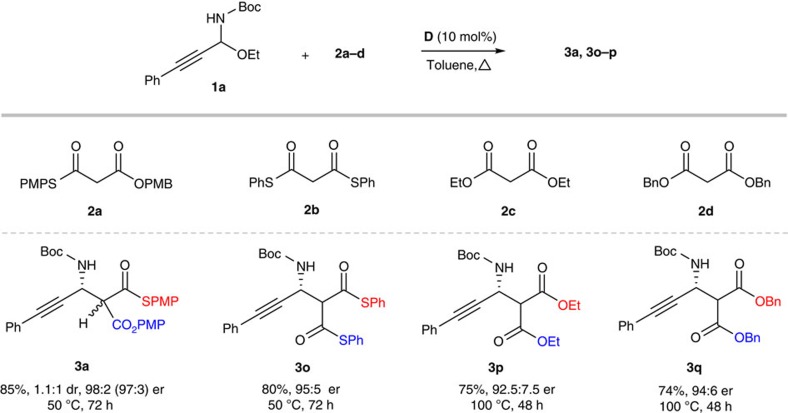
Chiral Brønsted base-catalysed Mannich-type reactions with malonate (thio)esters. Reaction conditions: **1a** (0.1 mmol), **2a**–**d** (0.12 mmol), the catalyst **D** (0.01 mmol, 10 mol %), toluene (1 ml), 50 or 100 °C, 72 h.

**Figure 5:**
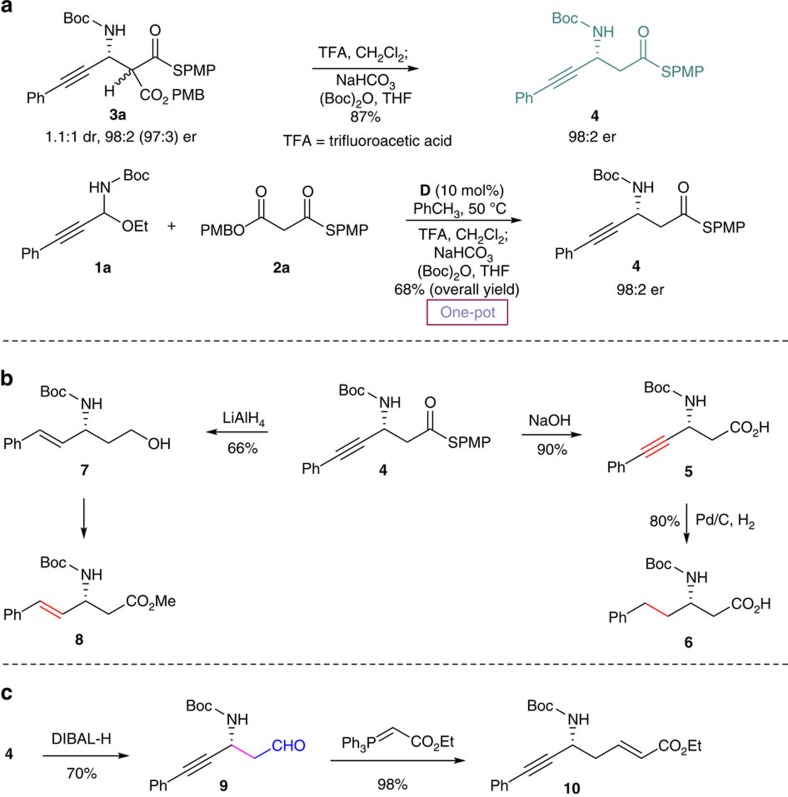
Unified synthesis of β-alkynyl-, alkenyl- and alkyl-β-amino acids and derivatives. (**a**) Decarboxylation of the Mannich adduct leads to the production of β-alkynyl-β-amino thioester **4**. (**b**) Synthesis of β-alkynyl-, alkenyl- and alkyl-β-amino acid derivatives can be accomplished through simple transformations of **4**. (**c**) Facile synthesis of β-alkynyl-β-amino acetaldehyde and δ-amino-α,β-unsaturated ester.

Furthermore, this strategy also proved to be successful for various malonate (thio)esters, *S*,*O*-malonate **2a**, dithiomalonate **2b** and malonates **2c–d** (Fig. 4). The extension of this strategy to different malonate (thio)esters represents an important feature from a synthetic standpoint because it provides orthogonal sets of easily decarboxylative and/or functionalizable groups. It is also worth noting that examples with high stereocontrol at such a high temperature are scarce in chiral tertiary amine/thiourea catalysis. The product was readily converted into Boc-protected β-alkynyl-β-amino acid and derivatives as shown in Fig. 5. Decarboxylation of **3a** gave Boc-protected β-alkynyl-β-amino thioester **4** in high yield (87%) without loss of enantiopurity. Notably, Boc-protected β-alkynyl-β-amino thioester **4** could also be obtained directly from C-alkynyl *N*-Boc-*N*,*O*-acetal electrophile **1a** in a one-pot operation. Importantly, both enantiomers of Boc-protected β-alkynyl-β-amino thioester **4** could be obtained due to easy access of both enantiomers of the Mannich product (see Table 2, entry 8). Hydrolysis of **4** provided Boc-protected β-alkynyl-β-amino acid **5** in high yield. Reduction of Boc-protected β-alkynyl-β-amino thioester **4** with LiAlH_4_ provided 1,3-amino alcohol **7** that could be converted into chiral β-alkenyl β-amino ester **8** in a one-pot operation^39^. Chiral β-alkenyl β-amino ester **8** is the key intermediate of serine–threonine protein phosphatase inhibitors^40^. Our concise catalytic asymmetric Mannich method for the synthesis of β-alkenyl β-amino ester complementes Gani's chiral auxiliary-based conjugate addition approach that requires a lengthy protecting group exchange and a complex procedure for introducing the double bond of **8** (ref. 40). Hydrogenation of the alkyne group delivered β-alkyl-β-amino acid **6**. The absolute configuration was determined to be (*S*) by comparison with the optical rotation ([α]_D_) of the known (*S*)-enantiomer. Reduction of **4** with DIBAL-H (diisobutyl aluminium hydride) provided aldehyde **9**, a hitherto unattainable acetaldehyde Mannich adduct^41^,^42^ derived from the *N*-Boc imine having an alkynyl substituent. Wittig reaction of **9** gave valuable δ-amino-α,β-unsaturated ester **10** in excellent yield^43^.

During the course of our work of catalytic asymmetric synthesis of Boc-protected chiral β-alkynyl-β-amino acids and derivatives via chiral Brønsted base-catalysed asymmetric Mannich-type reaction of *in situ*-generated *N*-Boc C-alkynyl imines from C-alkynyl *N*-Boc-*N*,*O*-acetals with malonate (thio)esters, Maruoka and co-workers reported a related heterogeneous chiral Brønsted acid-catalysed Mannich-type reaction of C-alkynyl *N*-Boc-aminals with reactive acetylacetone and β-ketoesters as nucleophiles^13^,^14^. However, when this method was attempted with malonate (thio)esters **2a**–**d** as nucleophiles, we could not obtain the Mannich products (no reactions occurred). Unlike more acidic 1,3-diketone and β-ketoesters, the inherent difficulty of the direct Mannich-type reaction with malonate (thio)esters is due to their weak acidity (For p*K*a values for 1,3-dicarbonyl compounds, see ref. 44). Thus, our chiral Brønsted base catalysis strategy, enabled by the development of a synergistic catalytic activation mode, significantly complemented Maruoka's Mannich reactions in terms of catalyst mode of action and the type and scope of the competent enolic nucleophiles, and demonstrated the strategic utility in the synthesis of pharmaceutically and synthetically valuable compounds. Interestingly, we also noted that under the influence of chiral Brønsted base catalysts, C-alkynyl *N*-Boc-aminals were inert and could not eliminate BocNH_2_ to generate *N*-Boc C-alkynyl imines, thus further indicating significant challenge of generation of *N*-Boc C-alkynyl imines by chiral Brønsted base catalysis.

### Catalytic asymmetric approach to *syn*-propargylamines

**Figure 6:**
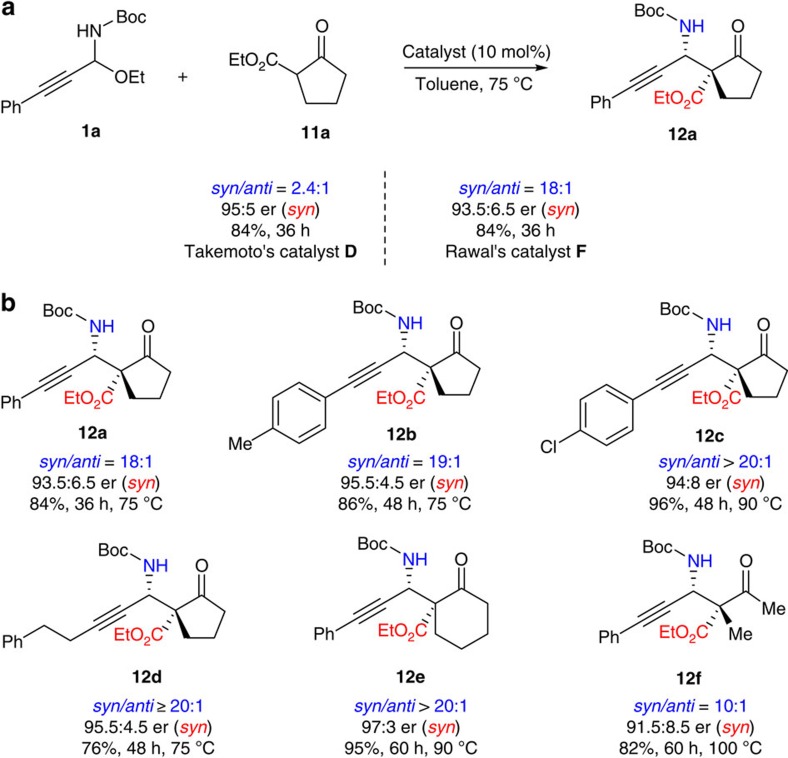
Catalytic asymmetric approach to *syn*-propargylamines. Chiral tertiary amine catalyst bearing a squaramide group leads to high *syn*/*anti* ratio. Reaction conditions: C-alkynyl *N-*Boc*-N,O*-acetal (0.10 mmol), α-substituted β-keto ester (0.2 mmol), the catalyst (0.01 mmol, 10 mol%), toluene (1.0 ml).

We investigated the reaction between C-alkynyl *N-*Boc*-N,O*-acetal **1a** and prochiral nucleophile **11a** in the presence of Takemoto's catalyst **D**. Indeed, our strategy enabled the formation of *syn*-propargylamine **12a**. However, the *syn*/*anti* ratio was quite low (2.4:1 dr; Fig. 6a). After considerable experiments, we were excited to discover that the use of Rawal's catalyst **F** with a squaramide group instead of a thiourea group can lead to the formation of *syn*-propargylamine **12a** in high *syn*/*anti* ratio (18:1 dr) and good enantioselectivity (Fig. 6a). Furthermore, various *syn*-propargylamines could be obtained in high stereocontrol (Fig. 6b).

Encouraged by an unexpected *syn*-diastereoselectivity, we further examined the *syn*-selective reaction with low catalyst loading (Table 4). When the catalyst loading was decreased from 10 mol% to 2 mol%, the yield and stereoselectivity (diastereo- and enantioselectivity) were not affected (entry 2 versus entry 1). This approach also features the lowest catalyst loading reported to date for catalytic asymmetric synthesis of propargylamines with two adjacent stereocenters from C-alkynyl imines^11^,^12^,^13^.

To gain a further insight into the chiral Brønsted base-catalysed asymmetric Mannich-type reaction of *in situ*-generated *N*-Boc-protected C-alkynyl imines from *N*-Boc-*N*,*O*-acetals, control experments were conducted. Replacement of the alkynyl group for the aryl group in the *N*-Boc-*N*,*O*-acetal substrates led to a remarkable loss of enantioselectivity (Supplementary Fig. 4). These results indicated that the alkynyl substituent in the *N*-Boc-*N*,*O*-acetal substrates is necessary for obtaining high stereoselectivity. Although to elucidate the specific reason is premature at this stage, low enantioselectivity in the catalytic asymmetric Mannich-type reaction of the aryl-substituted *N*-Boc-*N*,*O*-acetal is possibly due to strong background reaction.

## Discussion

In summary, we have developed a transition metal-free, organocatalytic asymmetric approach to pharmaceutically and synthetically important Boc-protected chiral β-alkynyl-β-amino acids via a mild chiral Brønsted base-catalysed asymmetric Mannich-type reaction of *in situ*-generated *N*-Boc-protected C-alkynyl imines with less-acidic malonate (thio)esters as nucleophile. High efficiency and practicality have been further demonstrated by a one-pot access to Boc-protected chiral β-alkynyl-β-amino thioesters and the use of a commercially available chiral organocatalyst. Notably, this methodology is highly flexible and could also be applicable to the catalytic asymmetric synthesis of biologically significant β-alkenyl-β-amino acids that are difficult to prepare by asymmetric catalysis, as well as β-alkyl-β-amino acids. Furthermore, both the D and L configurations of these β-amino acids could be accessible via this methodology.

Despite the significant challenge of *in situ* generation of *N*-Boc-protected C-alkynyl imines^45^ by chiral Brønsted base catalysts and the difficulty in substrate activation and reaction stereocontrol, our unexpected formation of previously unreported C-alkynyl *N*-Boc-*N*,*O*-acetals leading to the development of a synergistic catalytic activation strategy has enabled the final success without the use of any additional additives and co-catalysts. The power and utility of this strategy has been further demonstrated in the context of catalytic asymmetric construction of chiral propargylamines with two adjacent stereocenters, culminating in a highly *syn*-selective catalytic asymmetric Mannich reaction of C-alkynyl imines that provide *syn*-propargylamines. Furthermore, the *syn-*selective process can be performed by using a lower catalyst loading of only 2 mol%. This features the lowest catalyst loading reported to date for catalytic asymmetric synthesis of propargylamines with two adjacent stereocenters from C-alkynyl imines. Further studies to expand the application scope of this strategy are ongoing in our laboratory.

## Methods

### General methods and materials

^1^H-NMR and ^13^C-NMR spectra were recorded at 300, 400 and 500 MHz spectrophotometer. Chemical shifts (δ) are expressed in p.p.m., and *J*-values are given in Hz. The enantiomeric excess was determined by chiral high performance liquid chromatography (HPLC) with *n*-hexane and *i*-propanol as eluents. High resolution mass spectrometry was recorded on a VG Auto Spec-3000 spectrometer. Optical rotations were measured on a JASCO DIP-370 polarimeter. All chemicals and solvents were used as received without further purification unless otherwise stated. Flash column chromatography was performed on silica gel (230–400 mesh). For NMR analysis and HPLC traces of the compounds in this article, see Supplementary Figs 5–73. Characterization of the compounds in this article and DFT calculations see Supplementary Methods.

### General procedure for synthesizing N-Boc-N,O-acetals **1**

To a solution of ynal (10 mmol) in dichloromethane (80 ml) under nitrogen atmosphere was added Ti(OEt)_4_ (4.26 g, 15 mmol) and BocNH_2_ (1.76 g, 15 mmol) at room temperature. The mixture was stirred for 36 h at room temperature. The reaction was quenched by addition of H_2_O. The resulting mixture was extracted with CH_2_Cl_2_, and the combined organic phases were dried over Na_2_SO_4_. The mixture was concentrated at reduced pressure and the residue was purified by flash column chromatography, using AcOEt/hexane as the eluent, to afford C-alkynyl *N*-Boc-*N*,*O*-acetal **1**.

### General procedure for the asymmetric reaction of **1** and **2**

To a solution of **1** (0.10 mmol) and **2** (0.12 mmol) in toluene (1.0 ml), catalyst **D** was added (4.3 mg, 0.01 mmol) at 50 °C. After stirring for 72 h, the mixture was directly purified by silica gel chromatography, using AcOEt/hexane as the eluent, to afford the product **3**.

### Synthesis of β-alkynyl-β-amino thioester **4**

Compound **3a** (1.38 g, 2.40 mmol) was dissolved in CH_2_Cl_2_ (10 ml), and the resulting solution was added trifluoroacetic acid (TFA) (12 ml) and stirred for 3 min at room temperature. After the solvent and TFA was removed under reduced pressure, the mixture was dissolved in tetrahydrofuran (THF) (10 ml), and the resulting solution was added to saturated aqueous NaHCO_3_ (25 ml) and (Boc)_2_O (630 mg, 2.88 mmol) and stirred at room temperature until the starting material disappeared (monitored by thin-layer chromatography (TLC)). The mixture was extracted with CH_2_Cl_2_, dried over Na_2_SO_4_, concentrated and purified by silica gel column chromatography, using PE/EA (10/1–5/1) as the eluent, to afford compound **4** (920 mg, 87% yield) as an yellow solid.

### Synthesis of β-alkynyl-β-amino acid **5**

Compound **4** (44 mg, 0.1 mmol) was dissolved in 2 ml of 2 N NaOH. 10% V/V of methanol was added to ensure a clear solution. The reaction was stirred for 36 h at room temperature until the starting material disappeared (monitored by TLC). The pH of the organic phase was decreased to pH 1 using concentrated HCl. The water phases were washed three times with CH_2_Cl_2_. The combined organic phases were dried over Na_2_SO_4_, concentrated and purified by silica gel column chromatography, using PE/EA (1/3–1/1) as the eluent, to afford compound **5** (26 mg, 90% yield) as a white solid.

### Synthesis of β-amino acid **6**

To a solution of **5** (29 mg, 0.1 mmol) in MeOH (1 ml), Pd/C was added (15 mg, 55 wt%) under argon atmosphere. Then the atmosphere was replaced with hydrogen gas. After stirring for 40 h, the reaction mixture was poured directly onto silica gel column chromatography to afford **6** (24 mg, 80% yield) as a white solid.

### Synthesis of 1,3-amino alcohol **7**

Compound **4** (88 mg, 0.20 mmol) was dissolved in THF (4 ml), and the resulting solution was added to LiAlH_4_ (32 mg, 0.84 mmol) and stirred for 4 h at 45 °C. The mixture was extracted with CH_2_Cl_2_, dried over Na_2_SO_4_, concentrated and purified by silica gel column chromatography, using PE/EA (10/1–4/1) as the eluent, to afford compound **7** (36.5 mg, 66% yield) as a colourless liquid.

### Syntheis of β-amino aldehyde **9**

To a solution of **4** (500 mg, 1.13 mmol) in CH_2_Cl_2_ (10 ml) under nitrogen atmosphere, DIBAL-H was added (1.5 ml, 1.50 mmol) at −78 °C. The mixture was stirred for 1.5 h at −78 °C, after which the saturated potassium sodium tartrate solution (75 ml) and MeOH (3 ml) were added. The mixture was allowed to warm to room temperature, stirred until the starting material disappeared (monitored by TLC). The resulting mixture was extracted with CH_2_Cl_2_, dried over Na_2_SO_4_, concentrated and purified by silica gel column chromatography, using PE/EA (10/1) as the eluent, to afford compound **9** as an yellow solid (241 mg, 78% yield).

### Syntheis of δ-amino-α,β-unsaturated ester **10**

Compound **9** (65 mg, 0.24 mmol) was dissolved in CH_2_Cl_2_ (2 ml), and the resulting solution was added to (tripheny-l,5-phosphanylidene)-acetic acid ethyl ester (250 mg, 0.7 mmol) and stirred at room temperature until the starting material disappeared (monitored by TLC). The mixture was concentrated, and purified by silica gel column chromatography, using PE/EA (8/1–5/1) as the eluent, to afford compound **10** (80 mg, 98% yield) as an yellow solid.

### General procedure for the syn-selective Mannich reaction

To a solution of C-alkynyl *N*-Boc-*N*,*O*-acetal **1** (0.10 mmol) and α-substituted β-keto ester **11** (0.2 mmol) in toluene (1.0 ml), catalyst **F** was added (4.5 mg, 0.01 mmol) at designed temperature. After stirring for 36–60 h, the mixture was directly purified by silica gel chromatography, using AcOEt/hexane as the eluent, to afford the product **12**.

## Additional information

**How to cite this article:** Wang, Y. *et al*. Asymmetric synthesis of *syn*-propargylamines and unsaturated β-amino acids under Brønsted base catalysis. *Nat. Commun.* 6:8544 doi: 10.1038/ncomms9544 (2015).

## Supplementary Material

Supplementary Information Supplementary Figures 1-73, Supplementary Methods and Supplementary References.

## Figures and Tables

**Figure 1:**
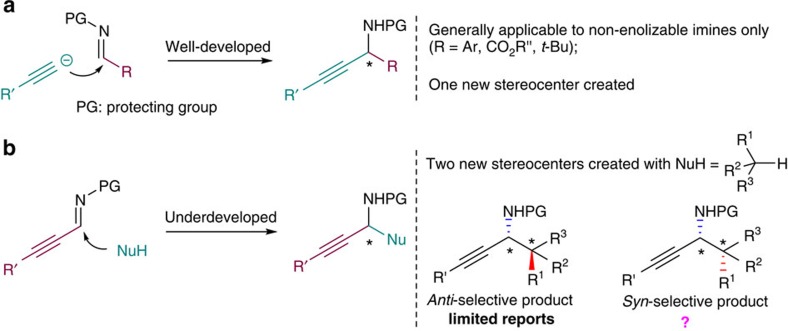
Catalytic asymmetric approaches to propargylamines through the C–C bond formation. (**a**) Alkynylation of imines. (**b**) Addition of carbon nucleophiles to C-alkynyl imines (the inherent flexibility in the structure of the nucleophilic components).

**Table 1:** Base-mediated tandem *N*-Boc C-alkynyl imine generation/Mannich-type reaction.

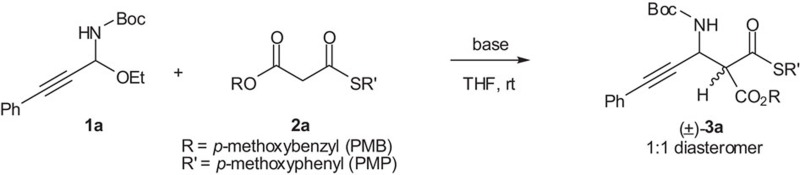		
**Entry**	**Base**	Yield (%)*
1	NaH	84
2	*t*-BuOK	80
3	NaHMDS	82
4	Et_3_N	0

Reaction conditions: **1a** (0.2 mmol), **2a** (0.24 mmol), base (0.4 mmol, 2 equiv), tetrahydrofuran (1 ml), 1 h.

^*^Isolated yield.

**Table 2:** Chiral Brønsted base-catalysed direct Mannich-type reaction of 1a and 2a.

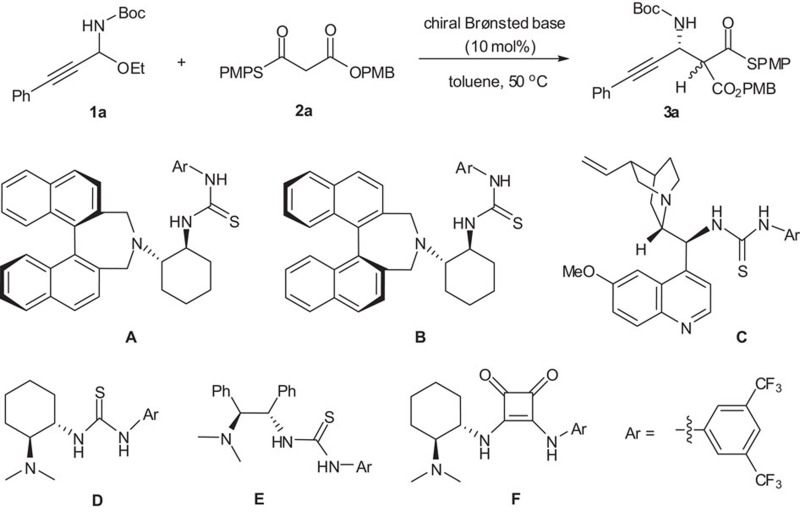				
**Entry**	**Catalyst**	Yield (%)*	***dr***†	***er***‡
1	(DHQD)_2_PHAL	0	—	—
2	quinine	0	—	—
3	cinchonidine	0	—	—
4	**A**	82	1.1:1	95.5:4.5 (95.5:4.5)
5	**B**	48	1.1:1	68.5:31.5 (68:32)
6	**C**	80	1.1:1	97:3 (97:3)
7	**D**	85	1.1:1	98:2 (97:3)
8	*en-***D**	83	1.1:1	2:98 (3:97)
9	**E**	80	1.1:1	95.5:4.5 (95.5:4.5)
10	**F**	86	1.1:1	98:2 (97:3)

Reactions condition: **1a** (0.1 mmol), **2a** (0.12 mmol), chiral base catalyst (0.01 mmol, 10 mol %), toluene (1 ml), 50 °C, 72 h.

^*^Isolated yield.

^†^Determined by ^1^H-NMR.

^‡^Determined by chiral high performance liquid chromatography (HPLC); the er value in the bracket is that of minor isomer.

**Table 3:** Chiral Brønsted base-catalysed Mannich-type reactions with different C-alkynyl *N*-Boc*-N*,*O*-acetals.

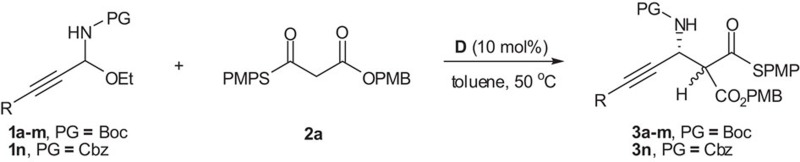					
**Entry**	**R**	**Product**	Yield (%)*	***dr***†	***er***‡
1	C_6_H_5_	**3a**	85	1.1:1	98:2 (97:3)
2	2-Me-C_6_H_4_	**3b**	84	1.1:1	95.5:4. 5 (95.5:4. 5)
3	3-Me-C_6_H_4_	**3c**	81	1.1:1	97.5:2.5 (97:3)
4	4-Me-C_6_H_4_	**3d**	80	1.1:1	98:2 (97:3)
5	4-MeO-C_6_H_4_	**3e**	83	1.1:1	94:6§
6||	4-Br-C_6_H_4_	**3f**	90	1.1:1	96:4 (96:4)
7||	4-Cl-C_6_H_4_	**3g**	81	1.1:1	96.5:3.5§
8¶	4-CF_3_-C_6_H_4_	**3h**	80	1.2:1	91.5:8.5 (90:10)
9	2-thienyl	**3i**	86	1.1:1	98.5:1.5 (98.5:1.5)
10	PhCH=CH	**3j**	87	1.1:1	96.5:3.5 (96:4)
11||	4-Cl-C_6_H_4_CH=CH	**3k**	80	3.0:1	94:6#
12	PhCH_2_CH_2_	**3l**	81	1.1:1	97:3 (97:3)
13	*n*-C_4_H_9_	**3m**	80	1.1:1	93:7 (91:9)
14	C_6_H_5_	**3n**	82	1.1:1	95.5:4.5 (95.5:4.5)

Reactions condition: **1a** (0.1 mmol), **2a** (0.12 mmol), the catalyst **D** (0.01 mmol, 10 mol %), toluene (1 ml), 50 °C, 72 h.

^*^Isolated yield.

^†^Determined by ^1^H-NMR.

^‡^Determined by chiral HPLC; the *er* value in the bracket is that of minor isomer.

^§^Minor isomer is not separated by HPLC.

^||^At 70 °C.

^¶^At 90 °C in the presence of catalyst **A**.

^#^The er value of major isomer.

**Table 4:** Reaction with low catalyst loading.

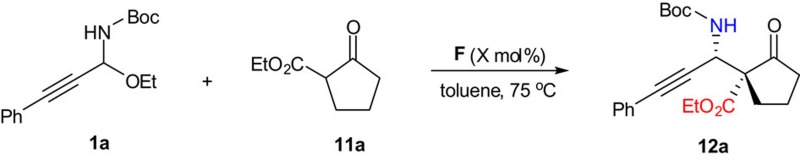					
**Entry**	***X*** **(mol%)**	**Time (h)**	Yield (%)*	***syn/anti***†	***er***‡
1	10	36	84	18:1	93.5:6.5
2	2	60	86	20:1	93:7

Reaction conditions: **1a** (0.10 mmol), **11a** (0.2 mmol), the catalyst **F** (0.01 mmol, 10 mol%), toluene (1.0 ml), 75 °C.

^*^Isolated yield.

^†^Determined by ^1^H-NMR.

^‡^Determined by chiral HPLC.
