# Supplementary material for: Asymmetric synthesis of syn-propargylamines and unsaturated β-amino acids under Brønsted base catalysis
Source: Nat Commun. 2015 Oct 1;6:8544. doi: 10.1038/ncomms9544 (PMC4600747; doi:10.1038/ncomms9544)
Supplement: Supplementary Information — Supplementary Figures 1-73, Supplementary Methods and Supplementary References. [file ncomms9544-s1.pdf]

a

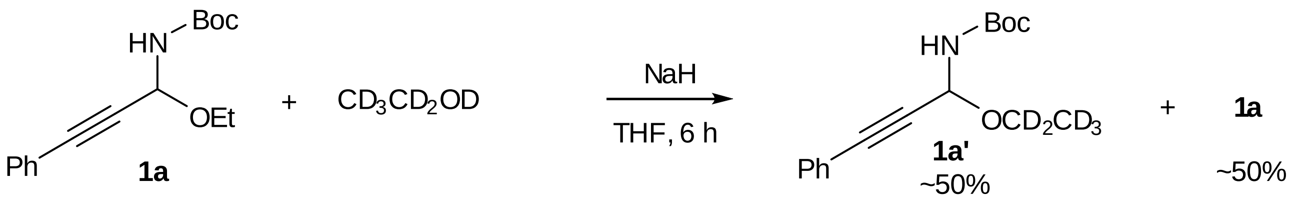

b

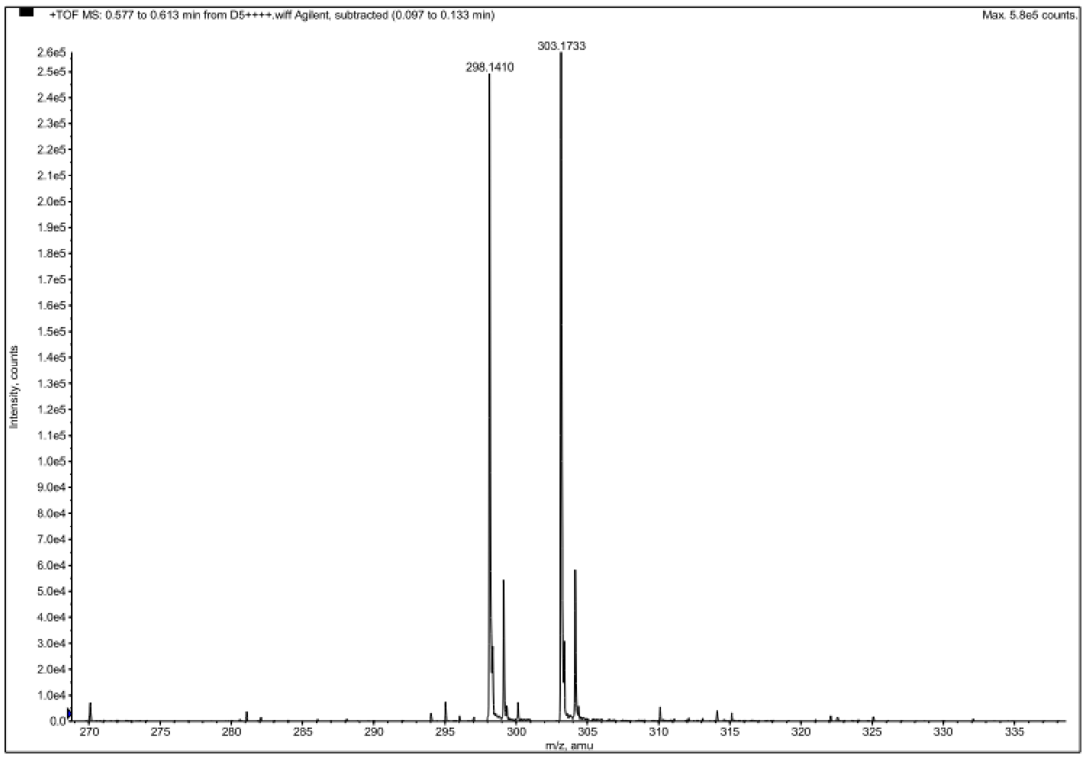

c

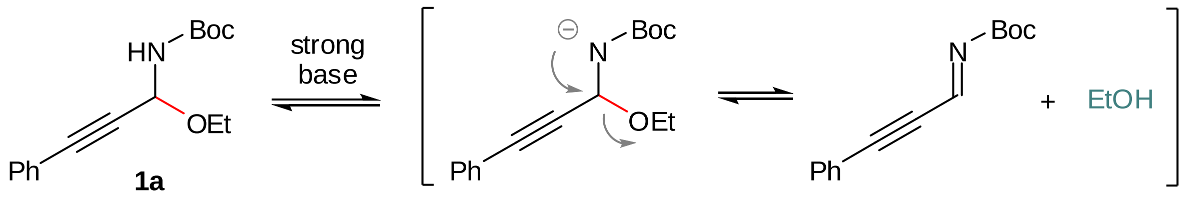

Supplementary Figure 1. Mechanistic study and working model for the base-mediated generation of C-alkynyl *N*-Boc imine. (**a**) Isotopic-labeling experiment. (**b**) HRMS analysis. (**c**) Working model.

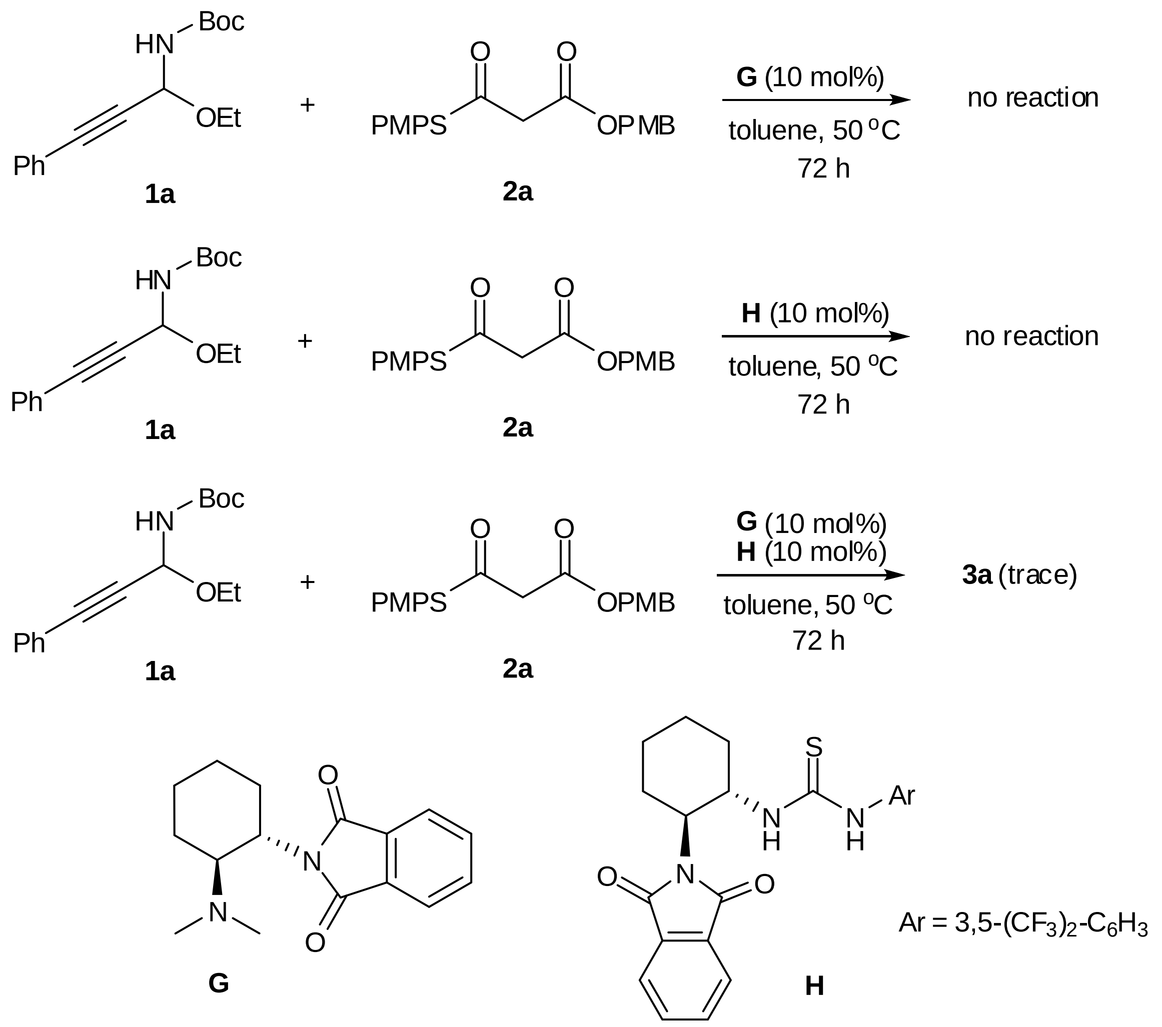

Supplementary Figure 2. Control experiments to investigate the roles of tertiary amine functionality and hydrogen donor in the in situ generation of C-alkynyl *N*-Boc imine.

a

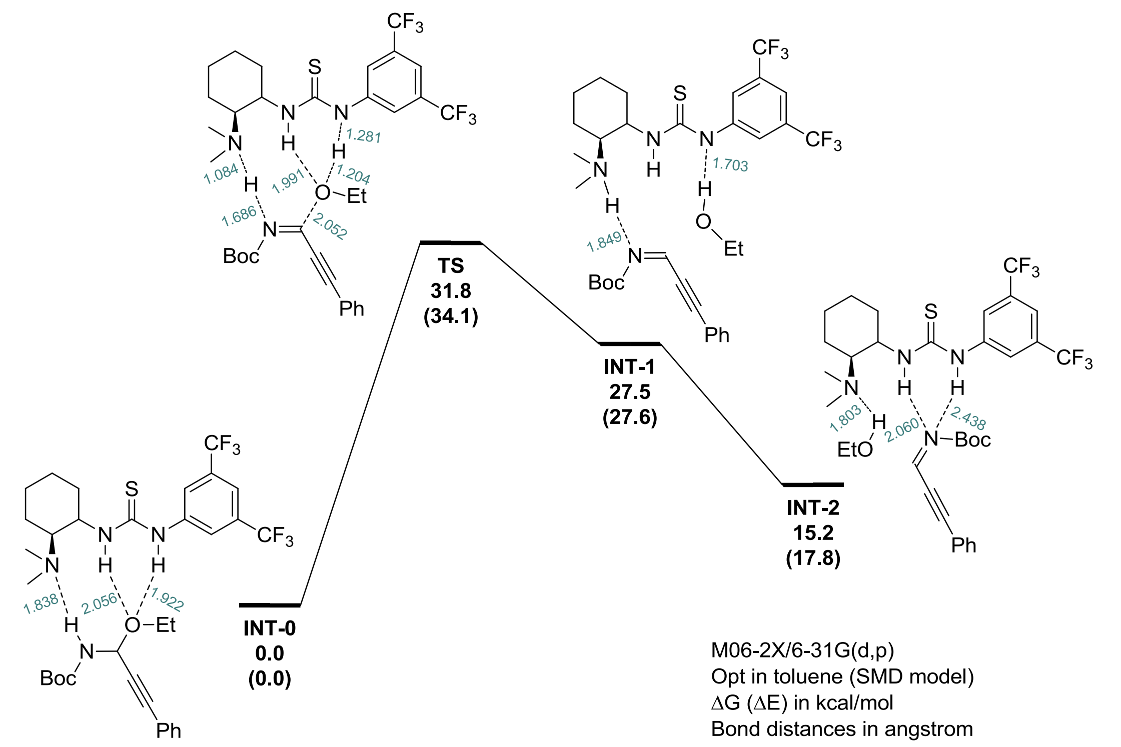

b

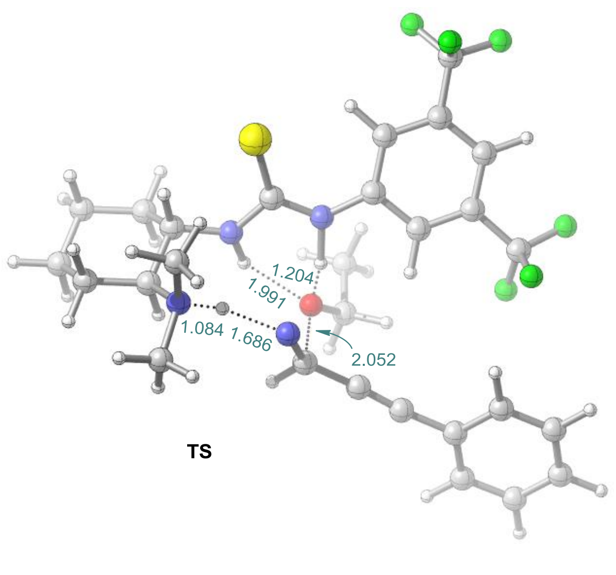

Supplementary Figure 3. Density functional theory (DFT) calculations. (**a**) The potential energy surface for the formation of C-alkynyl *N*-Boc imine intermediate. Δ*G* and Δ*E* (in parentheses) are in kcal/mol. Bond distances are in angstrom. (**b**) The 3D structure of **TS**. The Boc group is omitted for clarity. The bond distances are in angstrom.

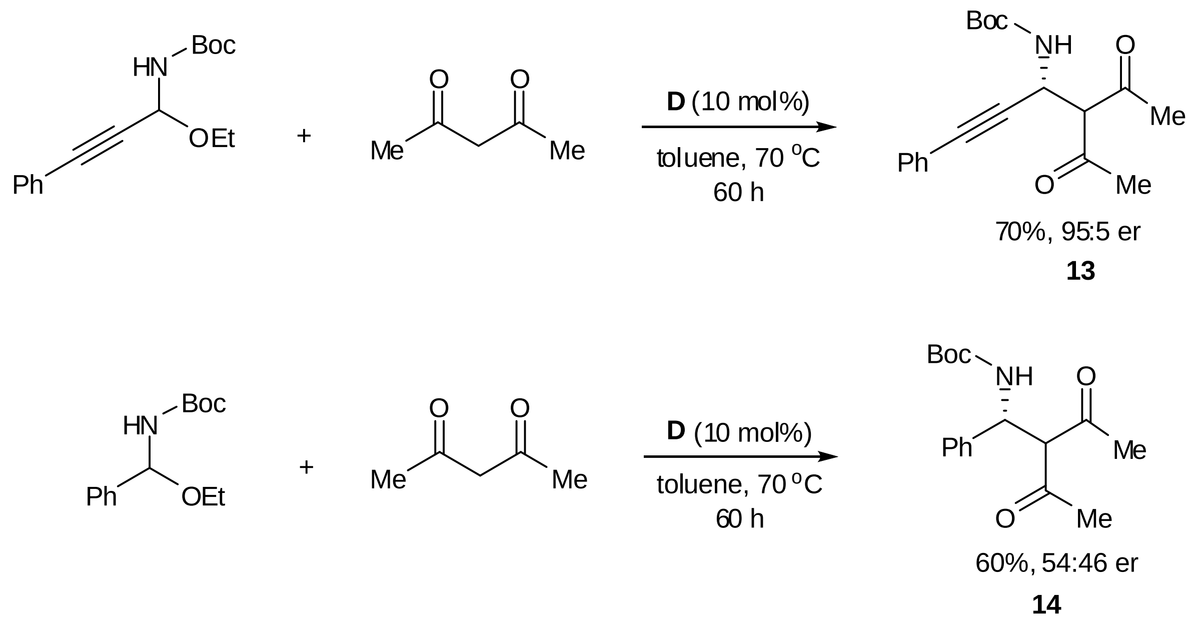

Supplementary Figure 4. Control experiments to investigate the roles of the alkynyl substituent in the catalytic asymmetric Mannich-type reaction of *N*-Boc-*N*,*O*-acetals.

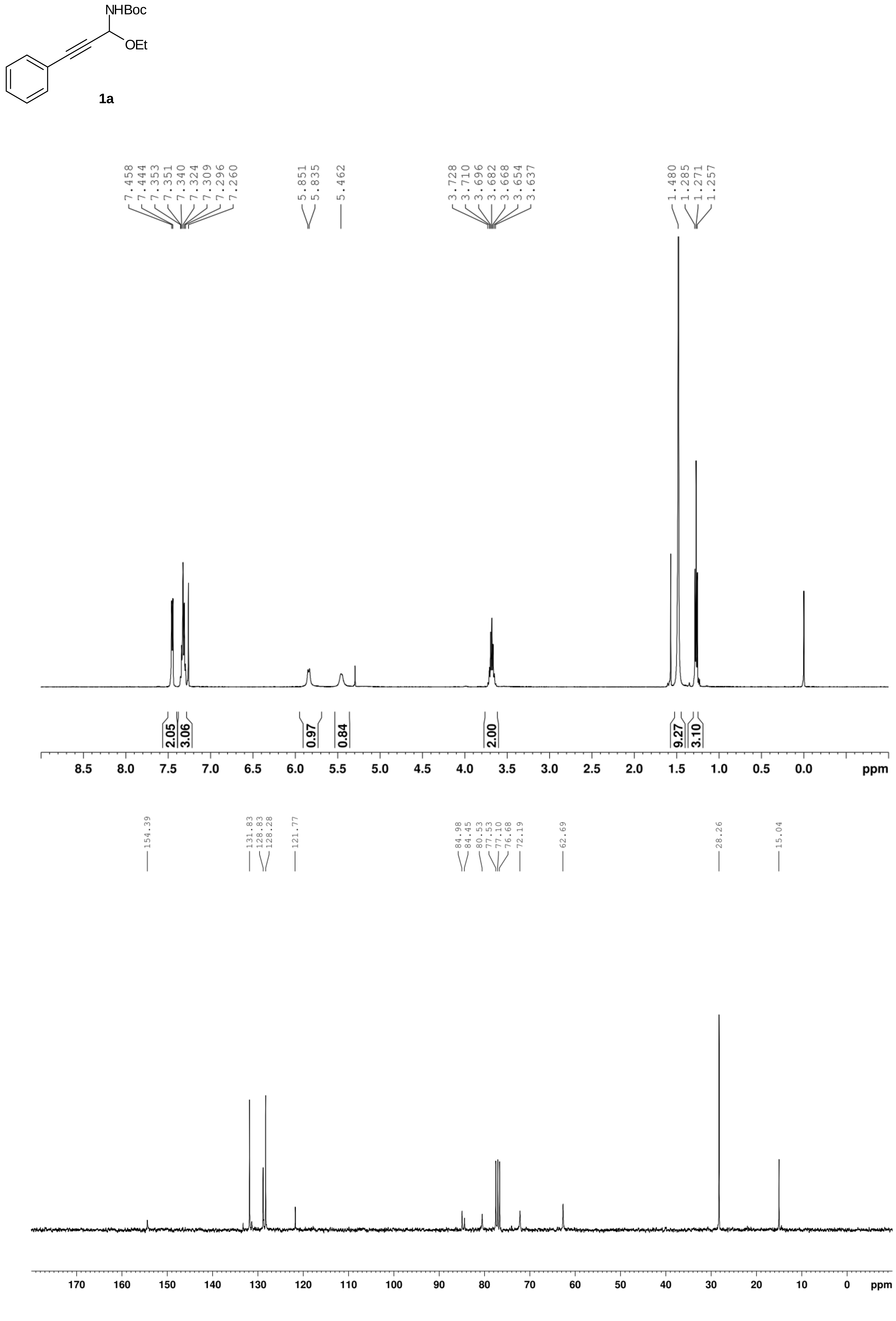

Supplementary Figure 5. ^{1}H and ^{13}C NMR spectra for **1a**.

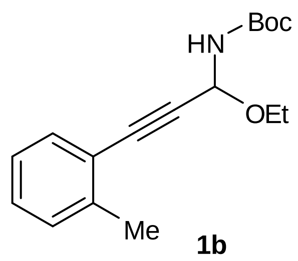

1b

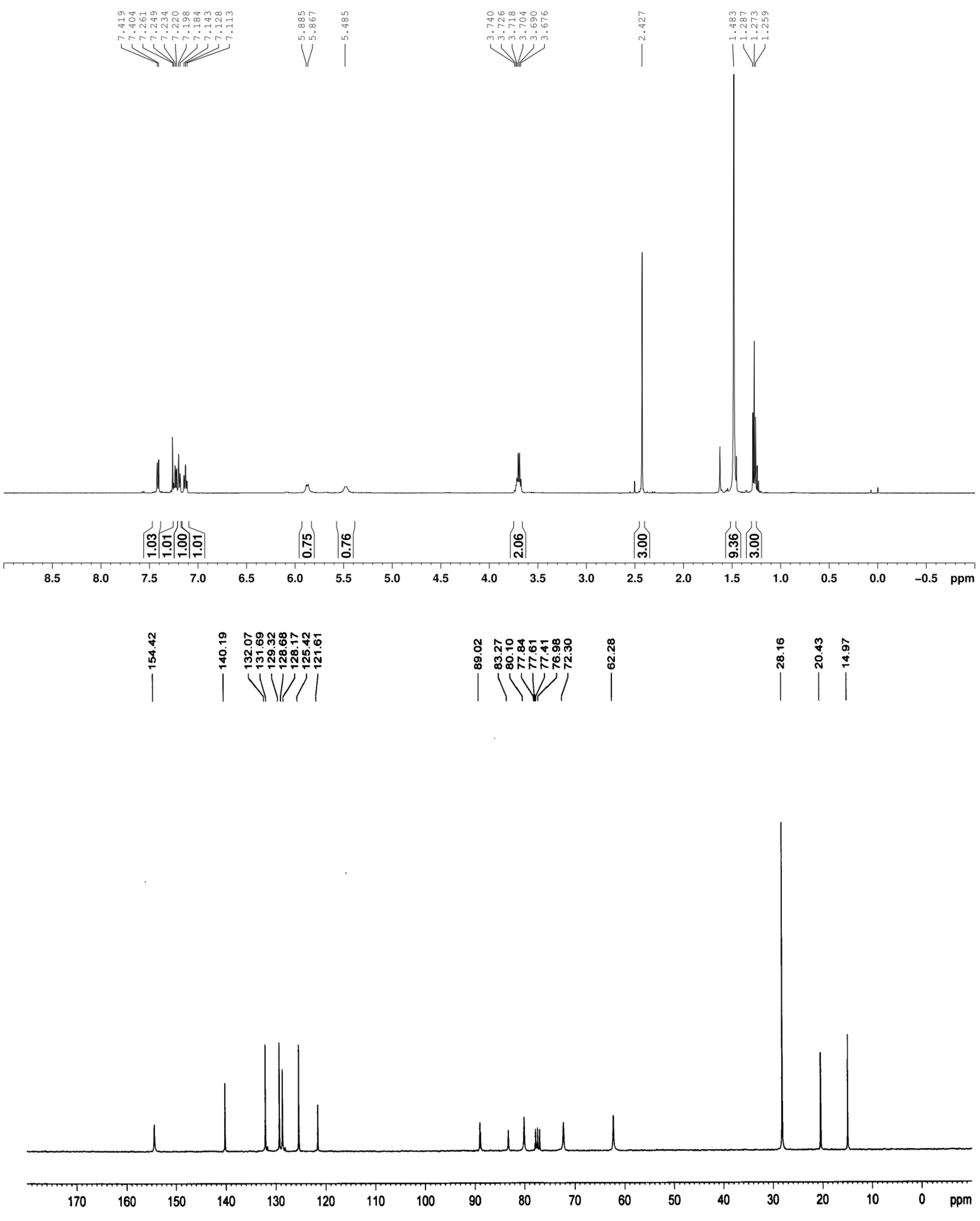

Supplementary Figure 6. ^{1}H and ^{13}C NMR spectra for **1b**.

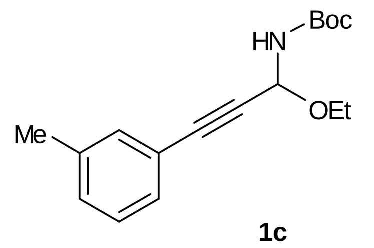

1c

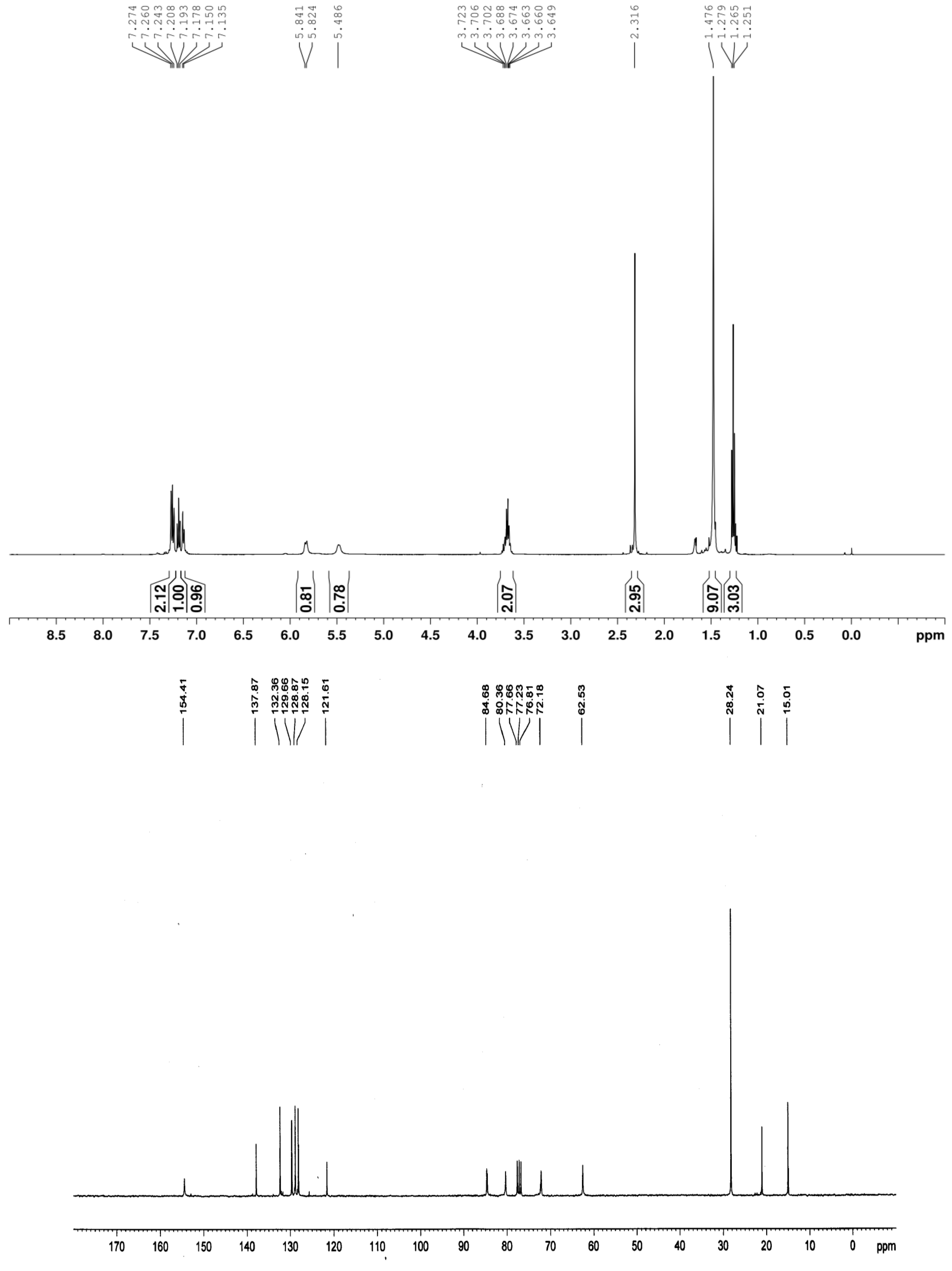

Supplementary Figure 7. ^{1}H and ^{13}C NMR spectra for **1c**.

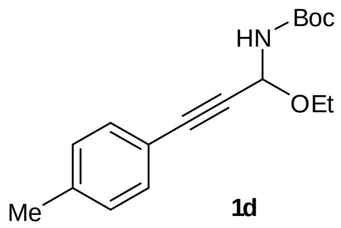

1d

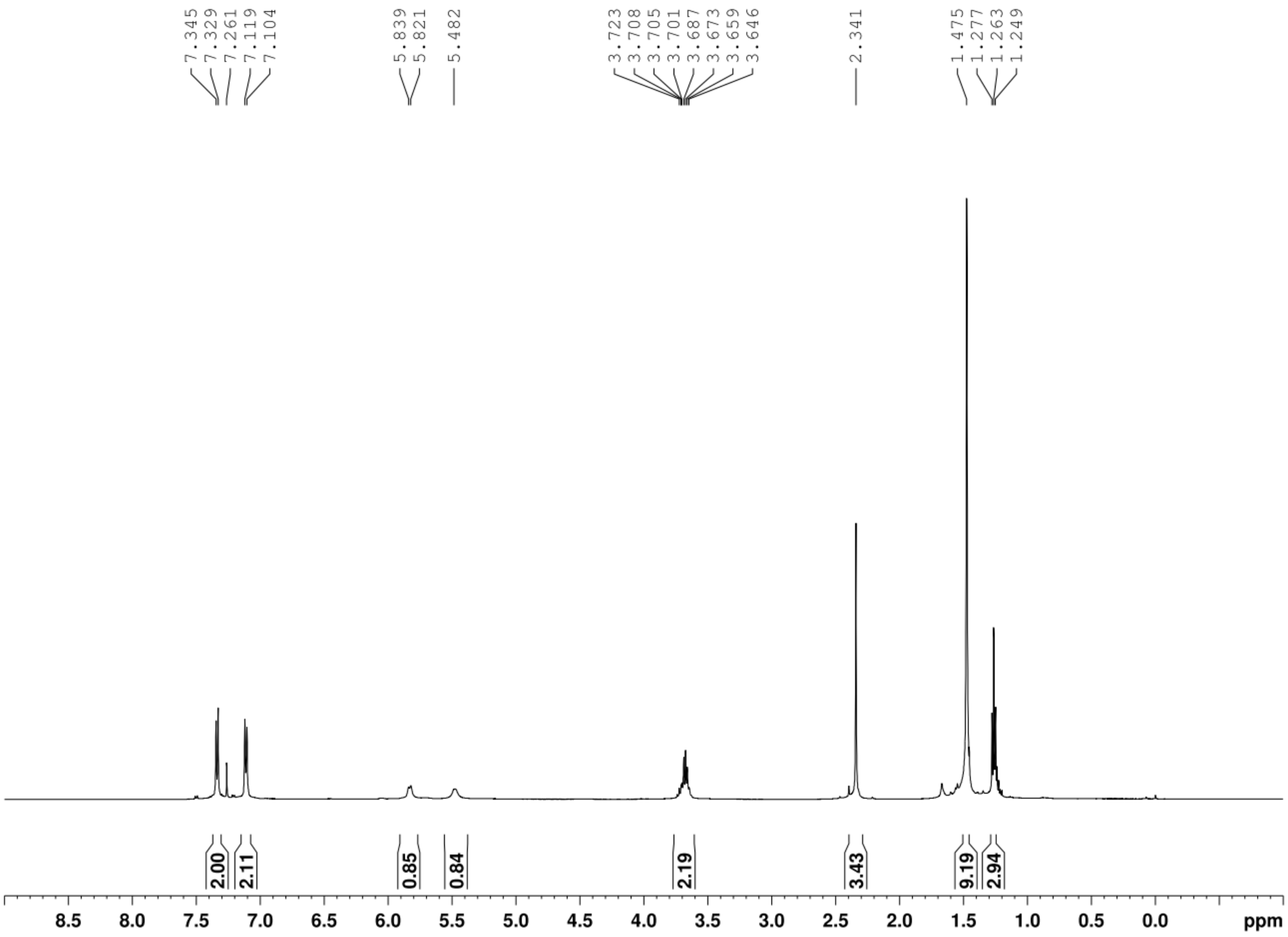

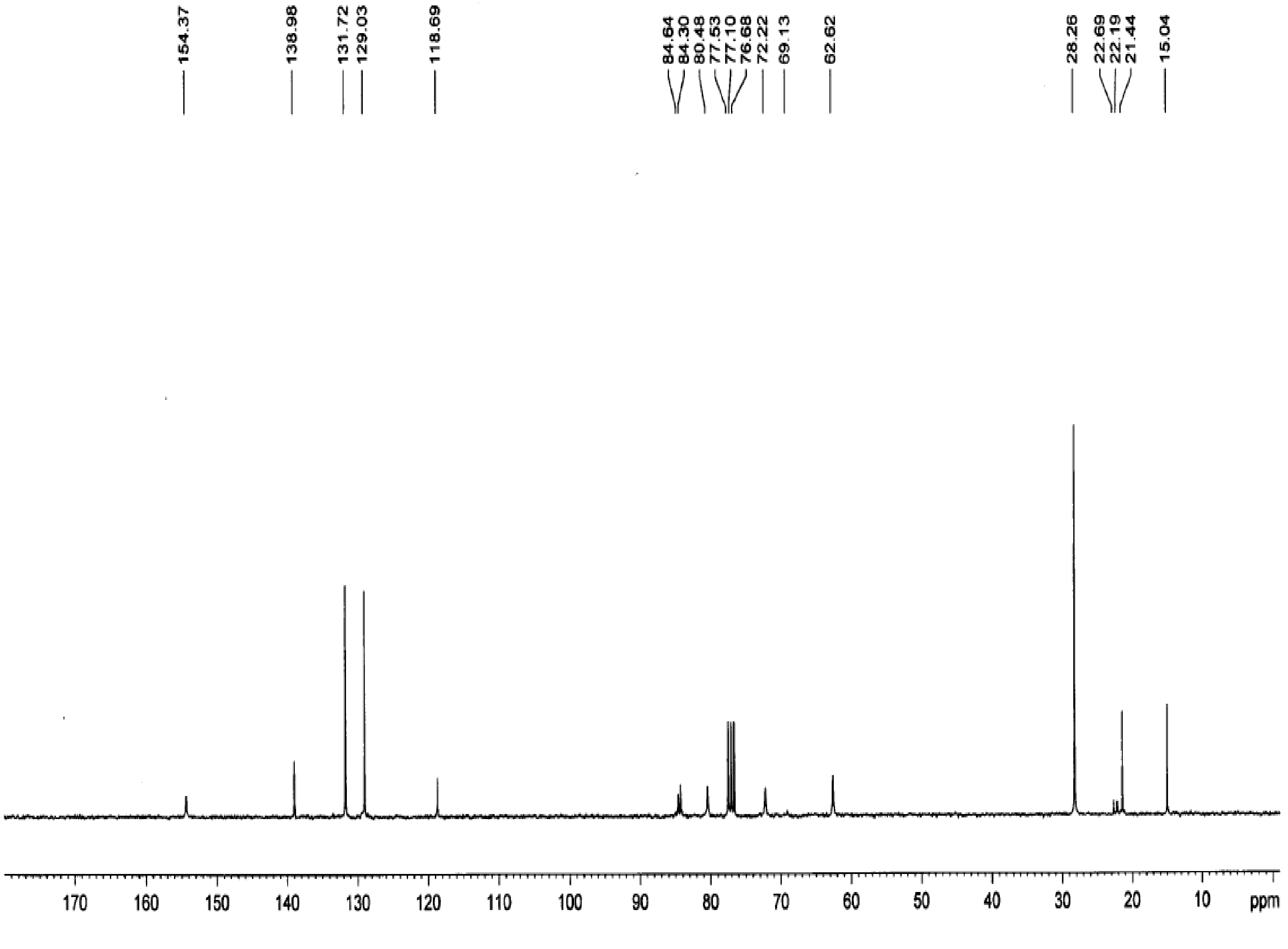

Supplementary Figure 8. ^{1}H and ^{13}C NMR spectra for **1d**.

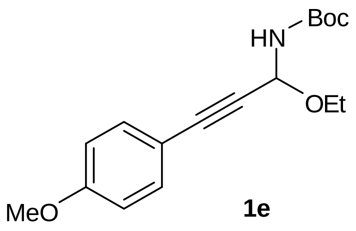

1e

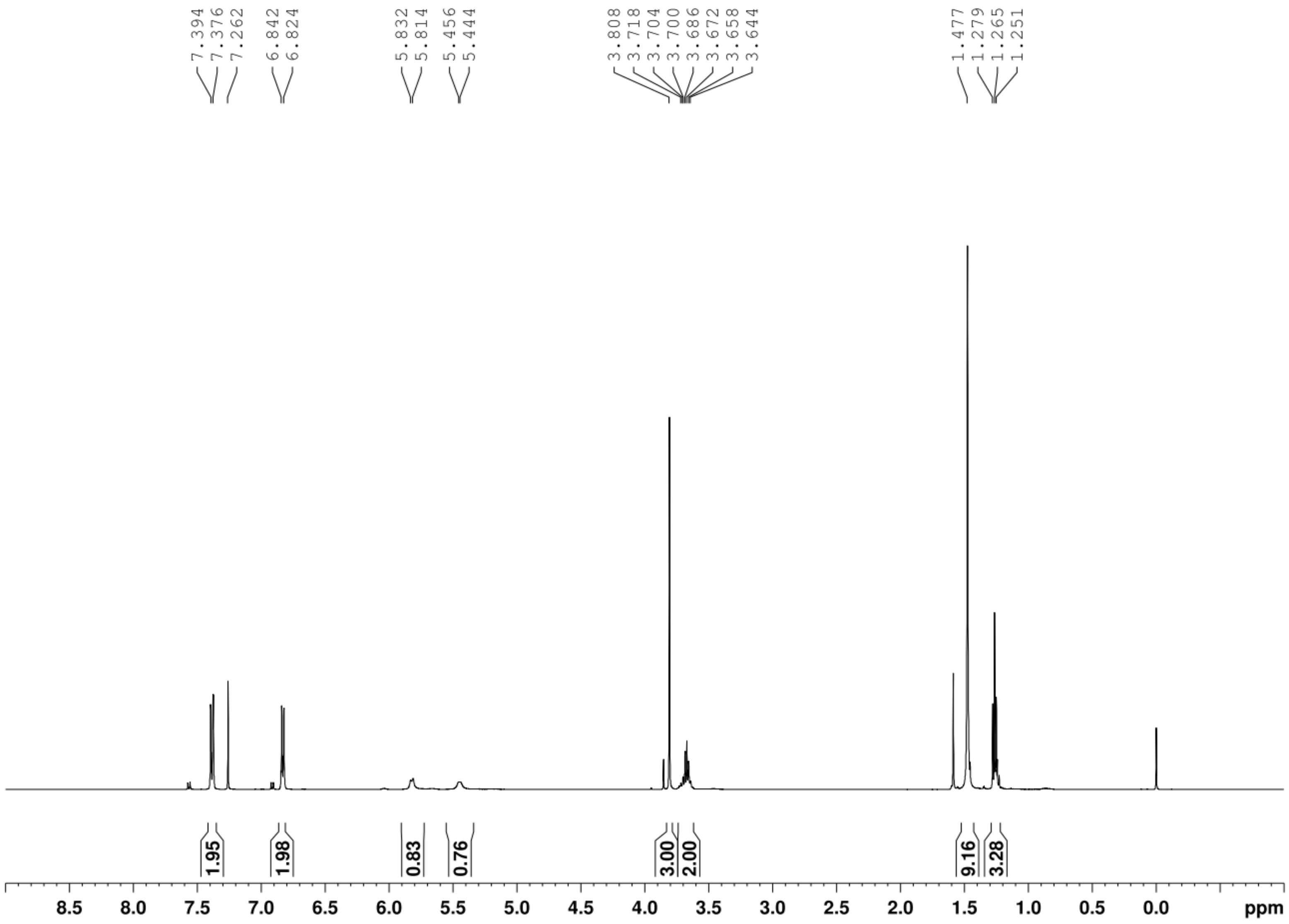

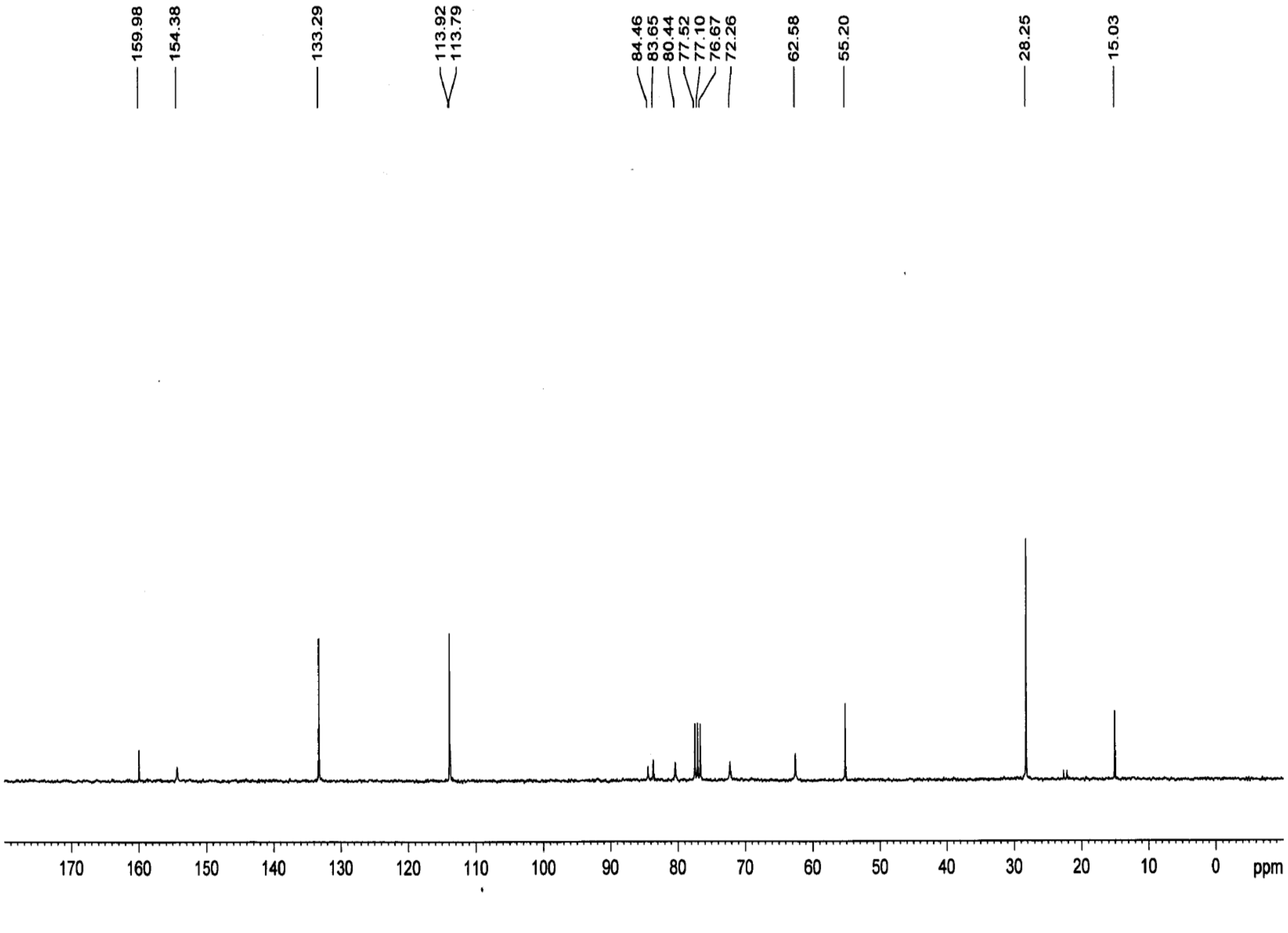

Supplementary Figure 9. ^{1}H and ^{13}C NMR spectra for **1e**.

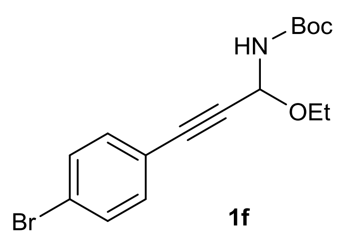

1f

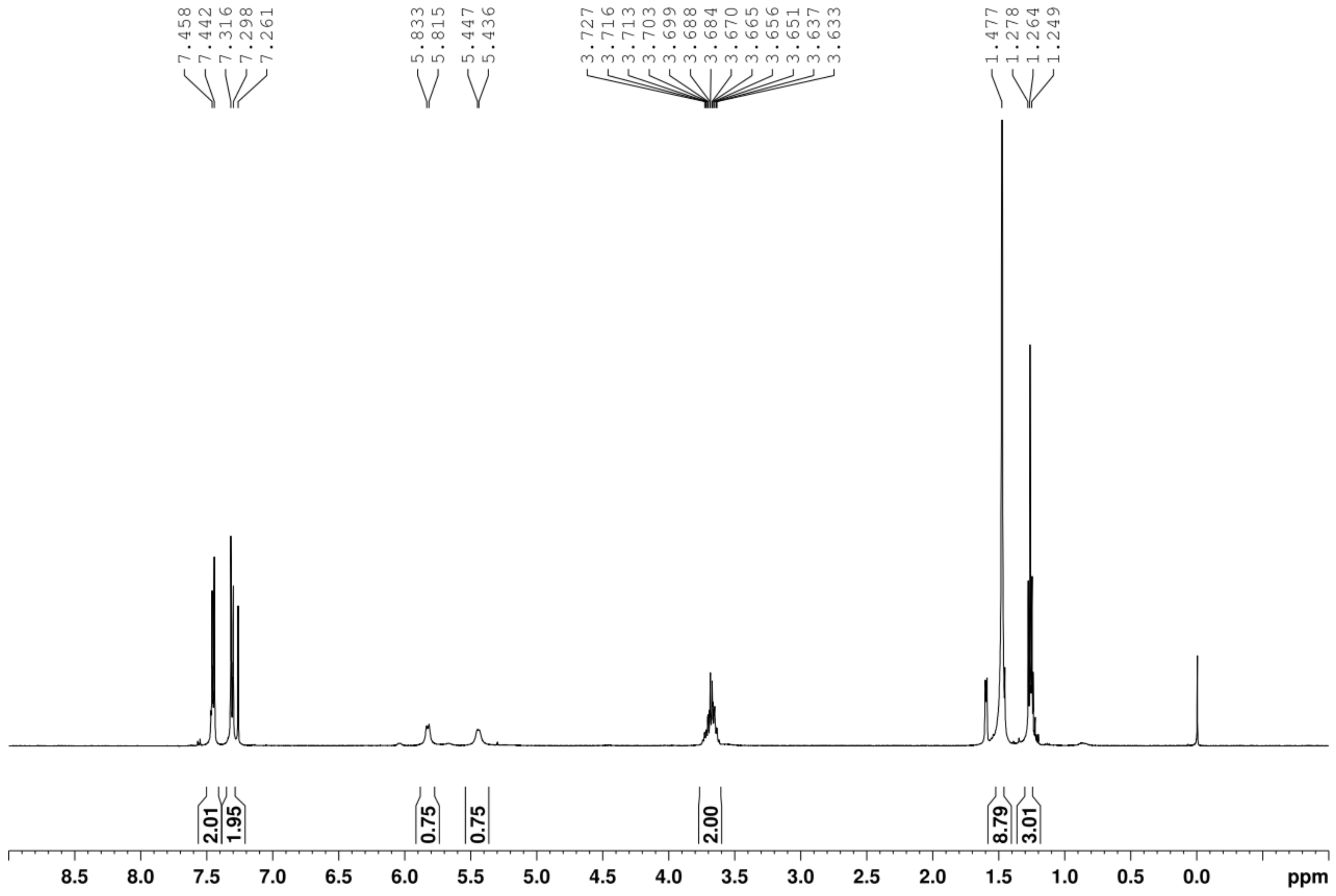

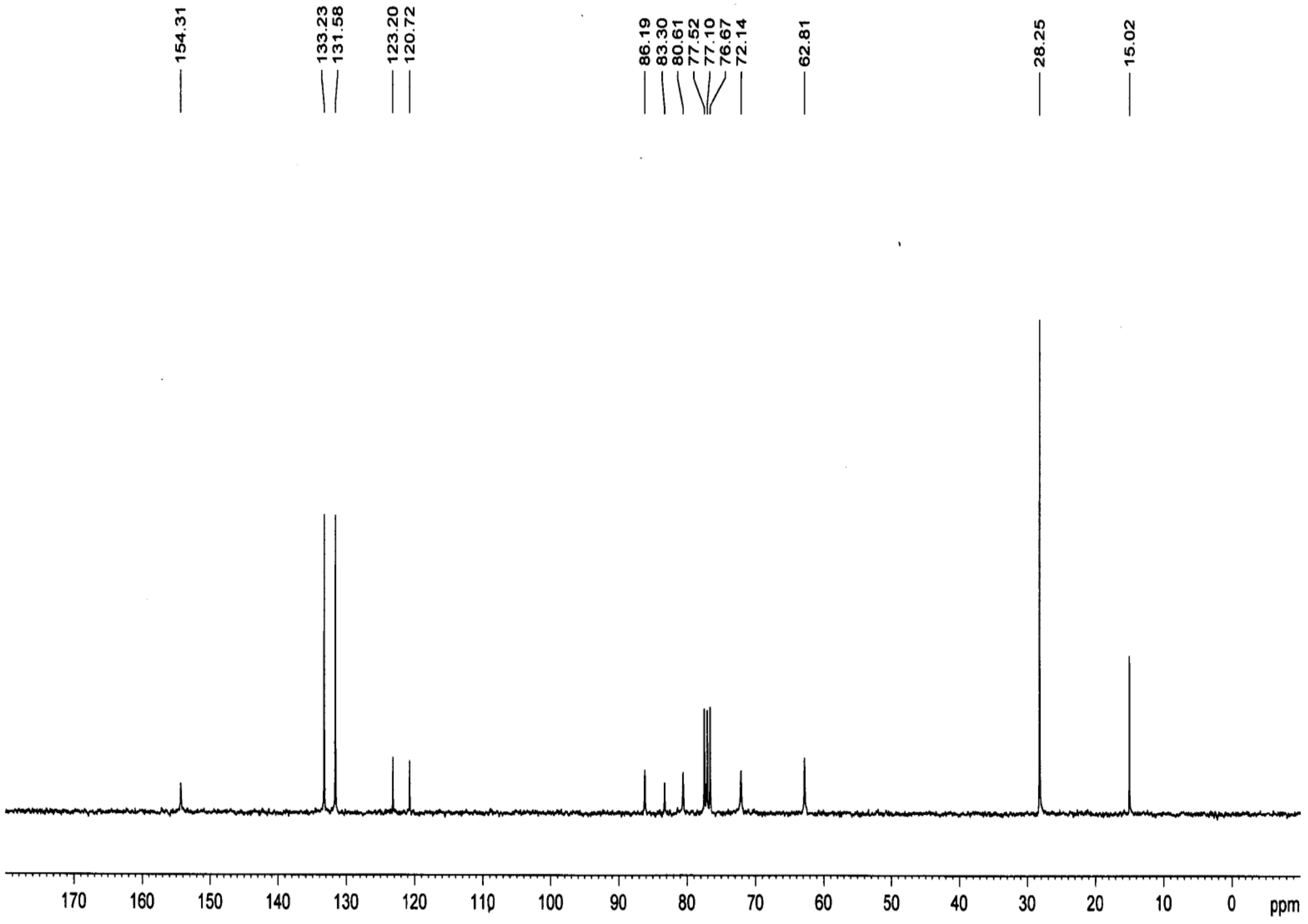

Supplementary Figure 10. ^{1}H and ^{13}C NMR spectra for **1f**.

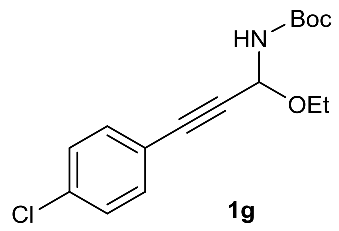

1g

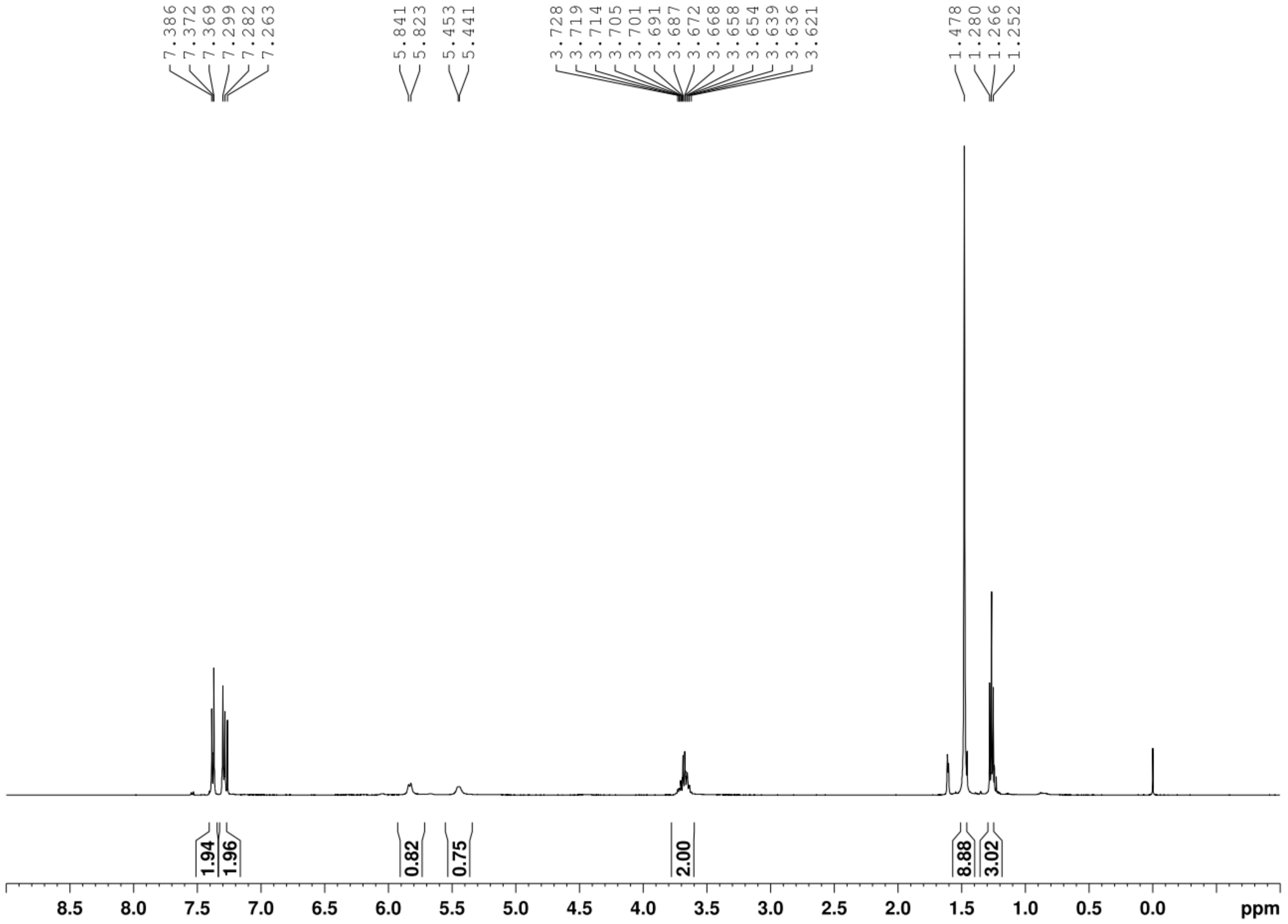

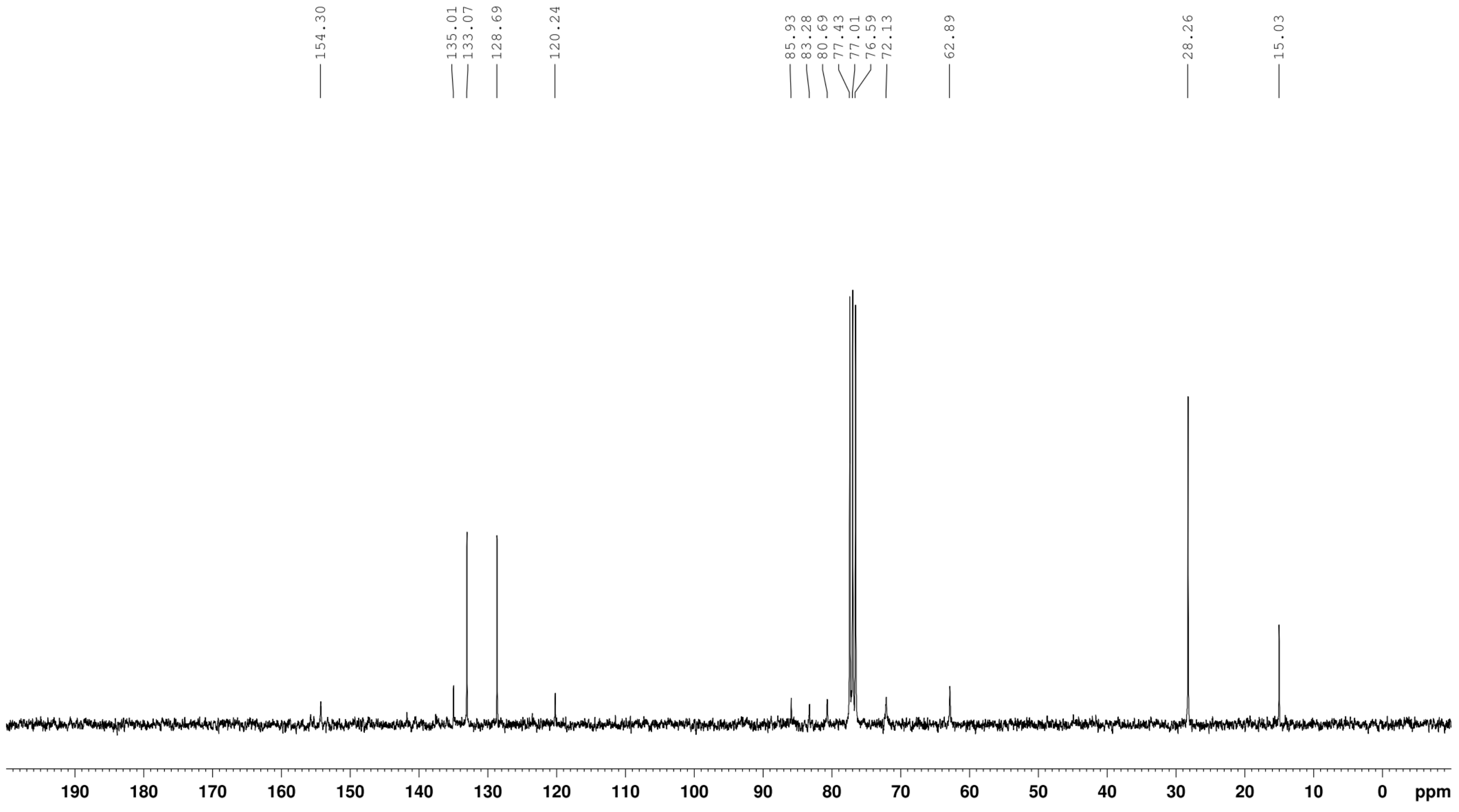

Supplementary Figure 11. ^{1}H and ^{13}C NMR spectra for **1g**.

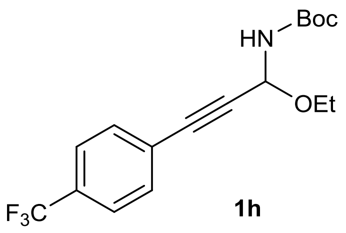

1h

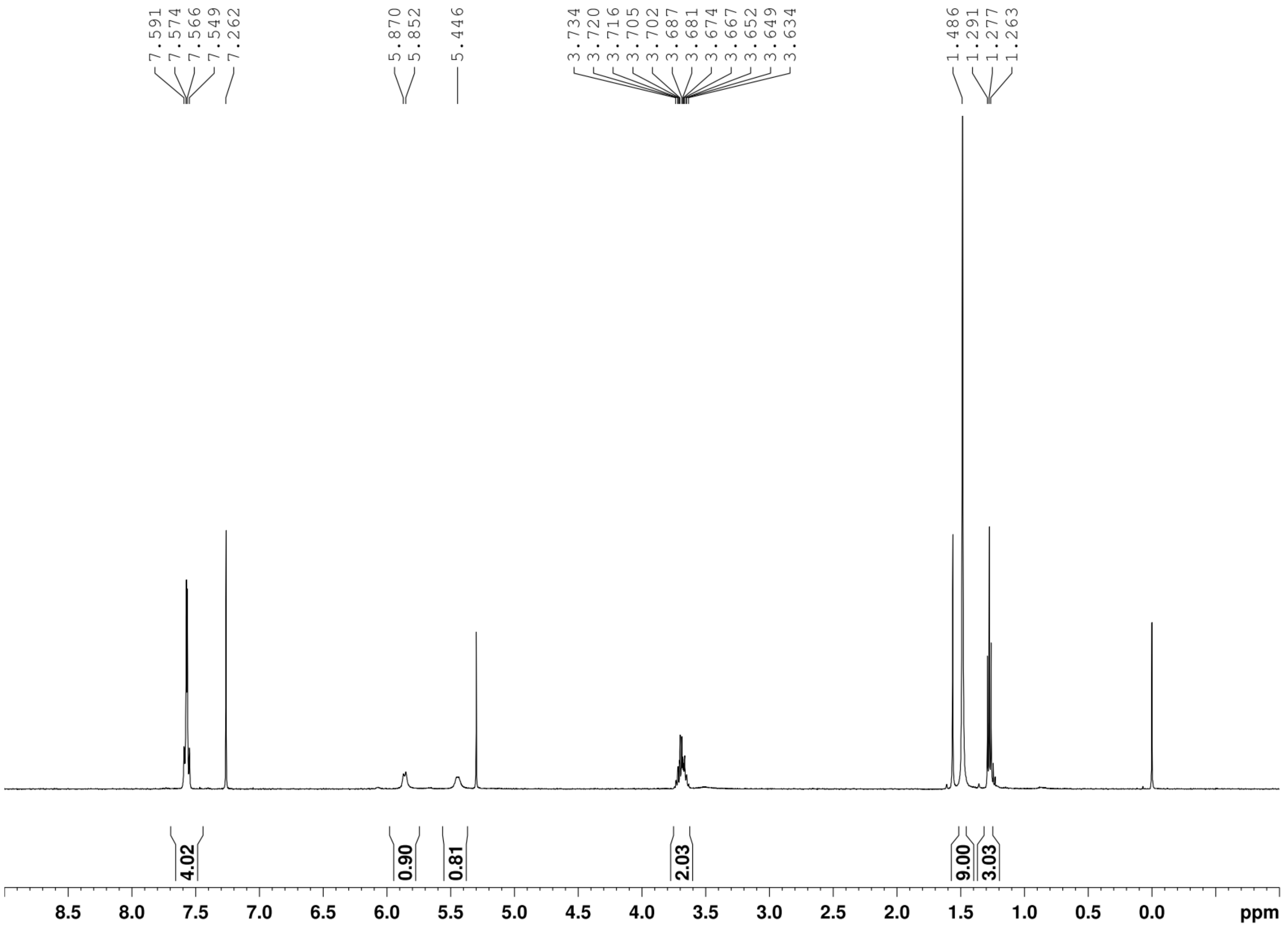

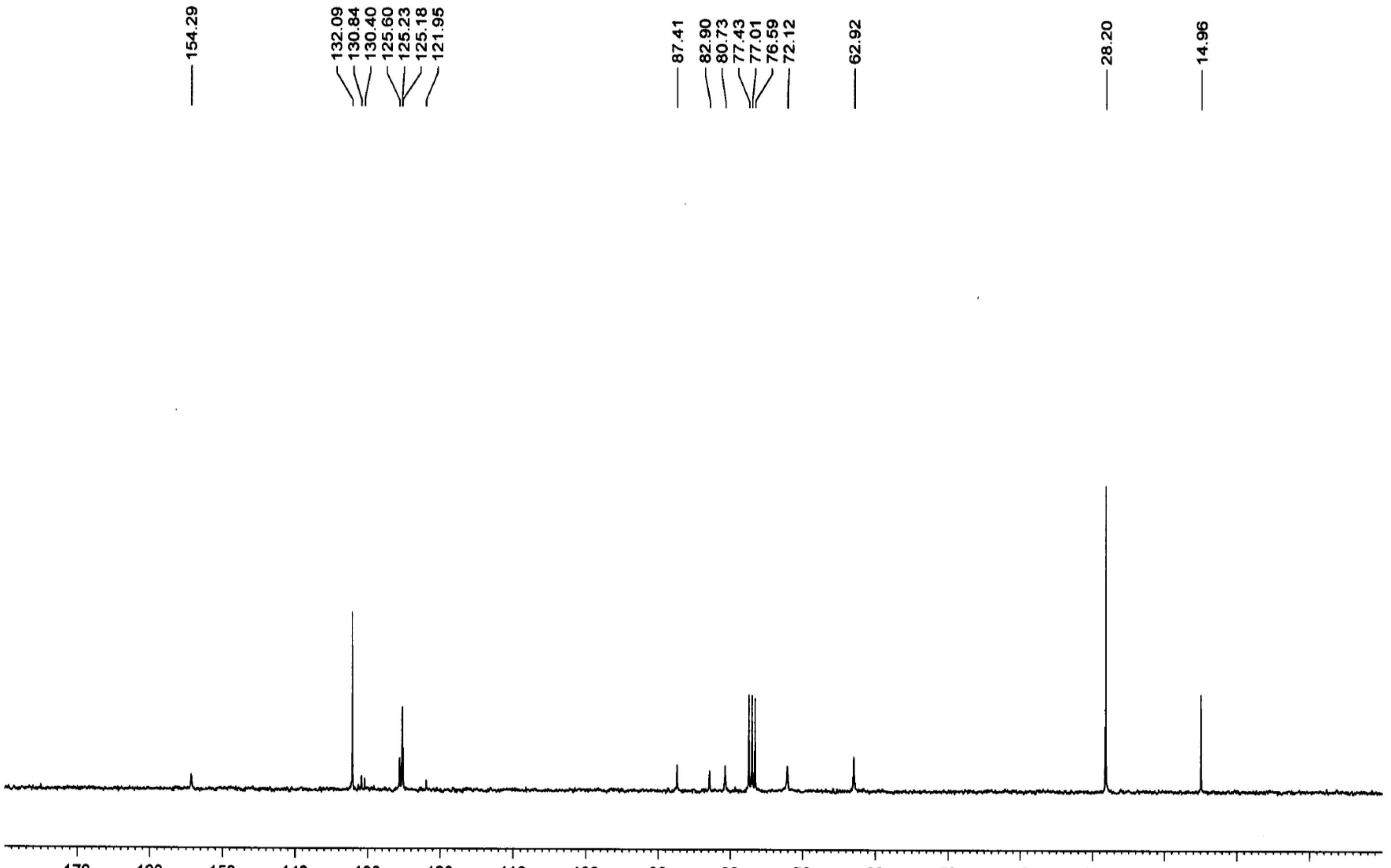

Supplementary Figure 12. ^{1}H and ^{13}C NMR spectra for **1h**.

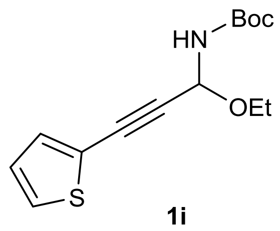

1i

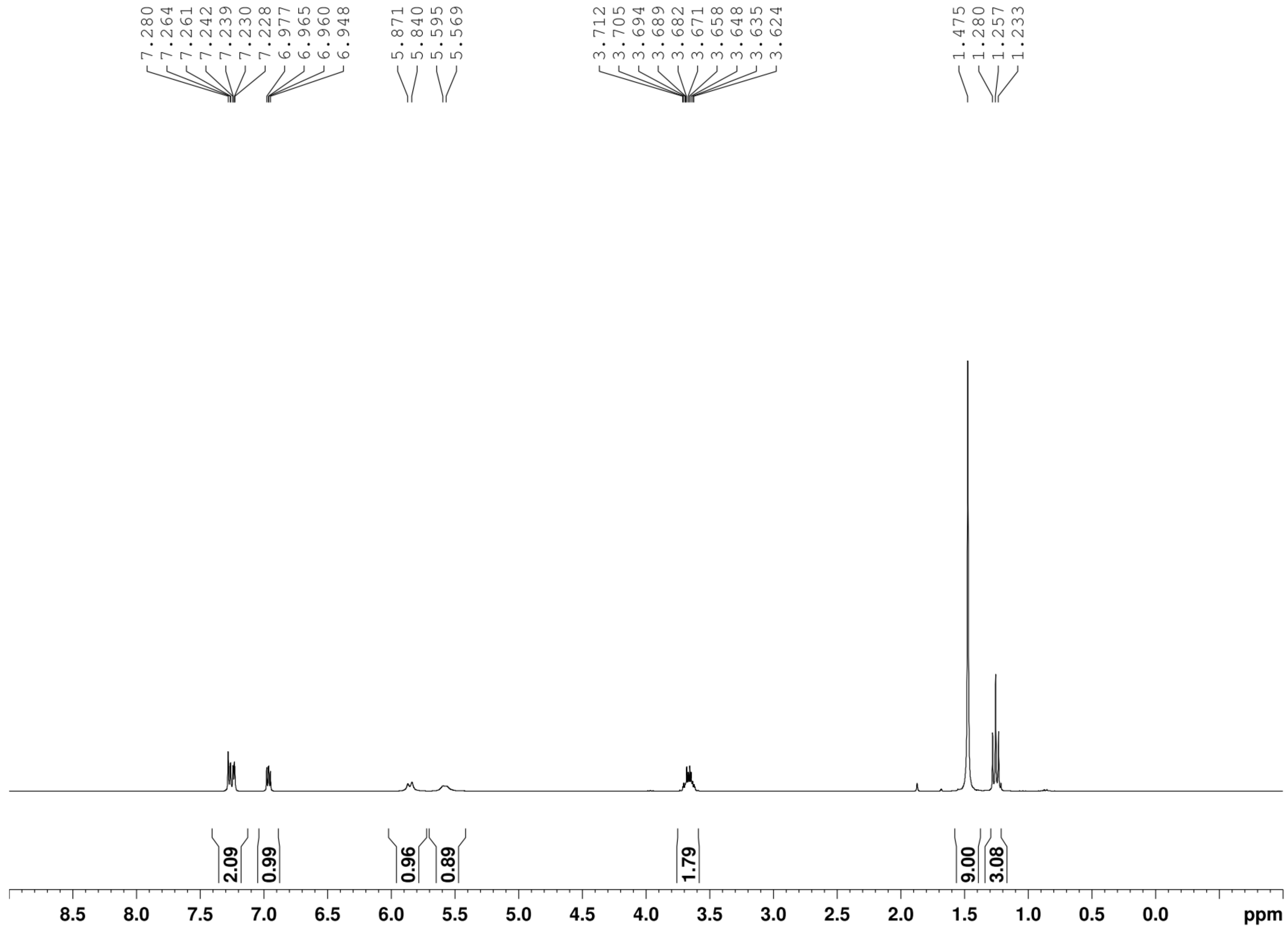

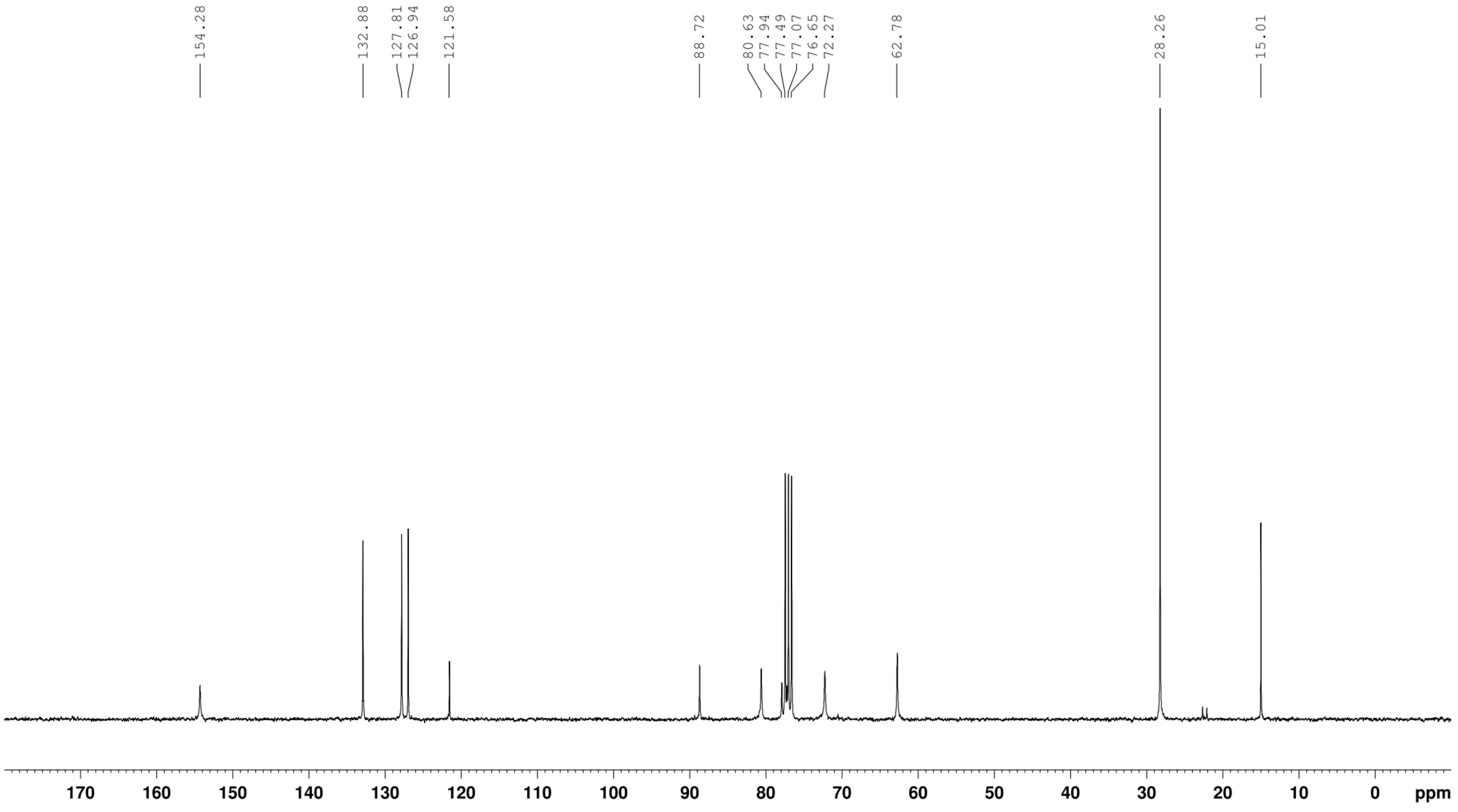

Supplementary Figure 13. ^{1}H and ^{13}C NMR spectra for **1i**.

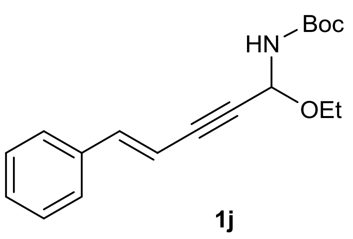

1j

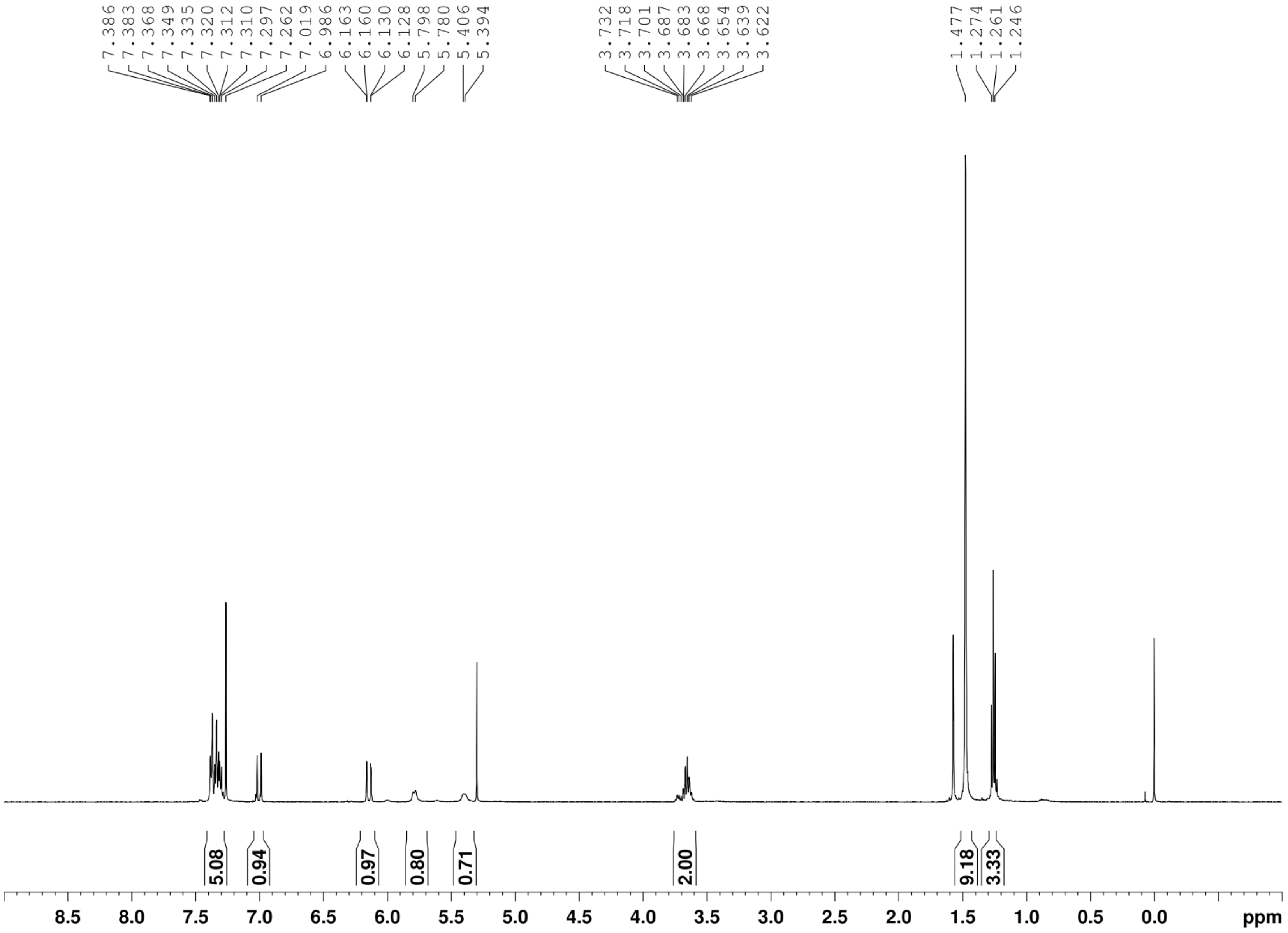

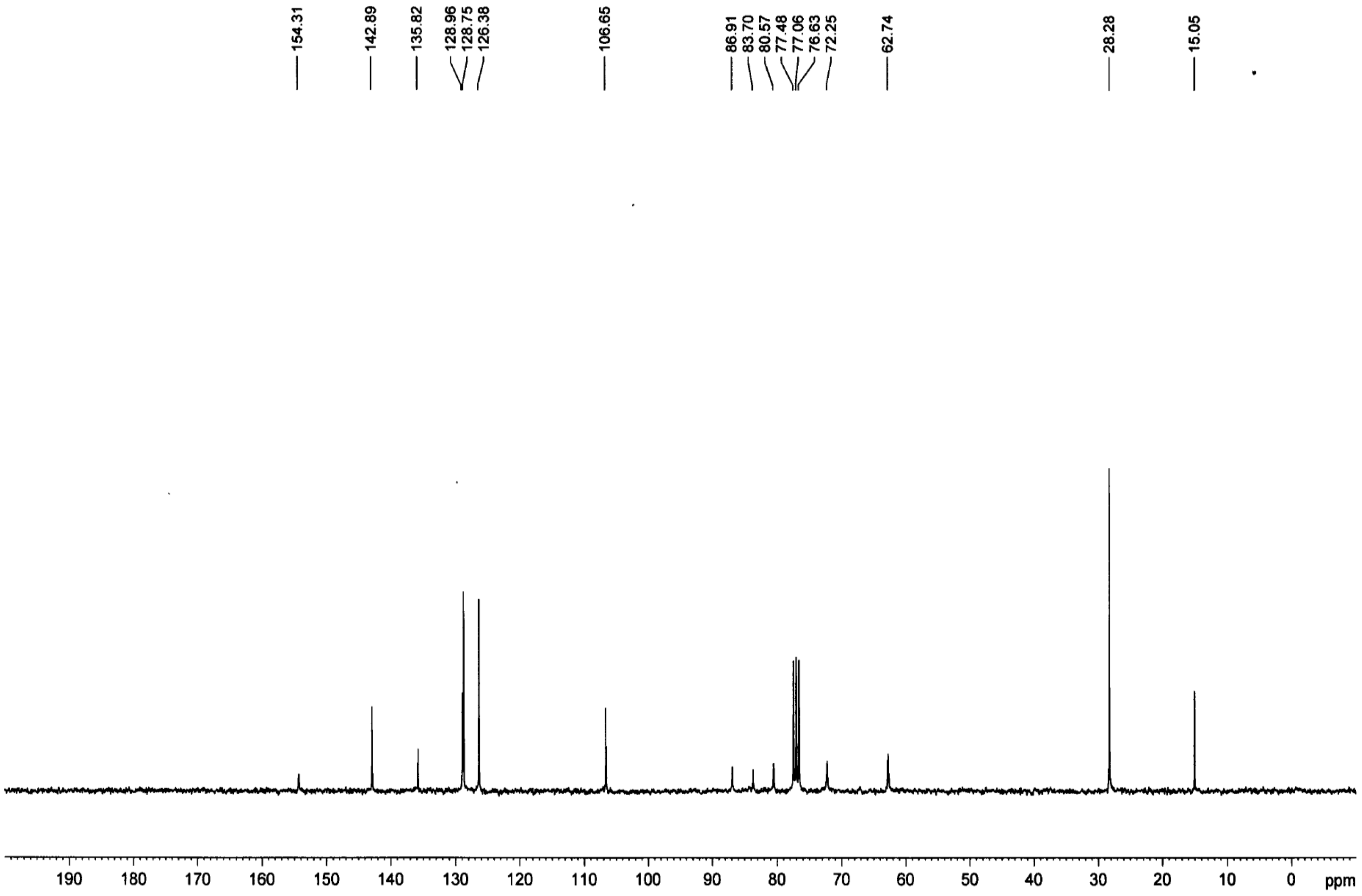

Supplementary Figure 14. ^{1}H and ^{13}C NMR spectra for **1j**.

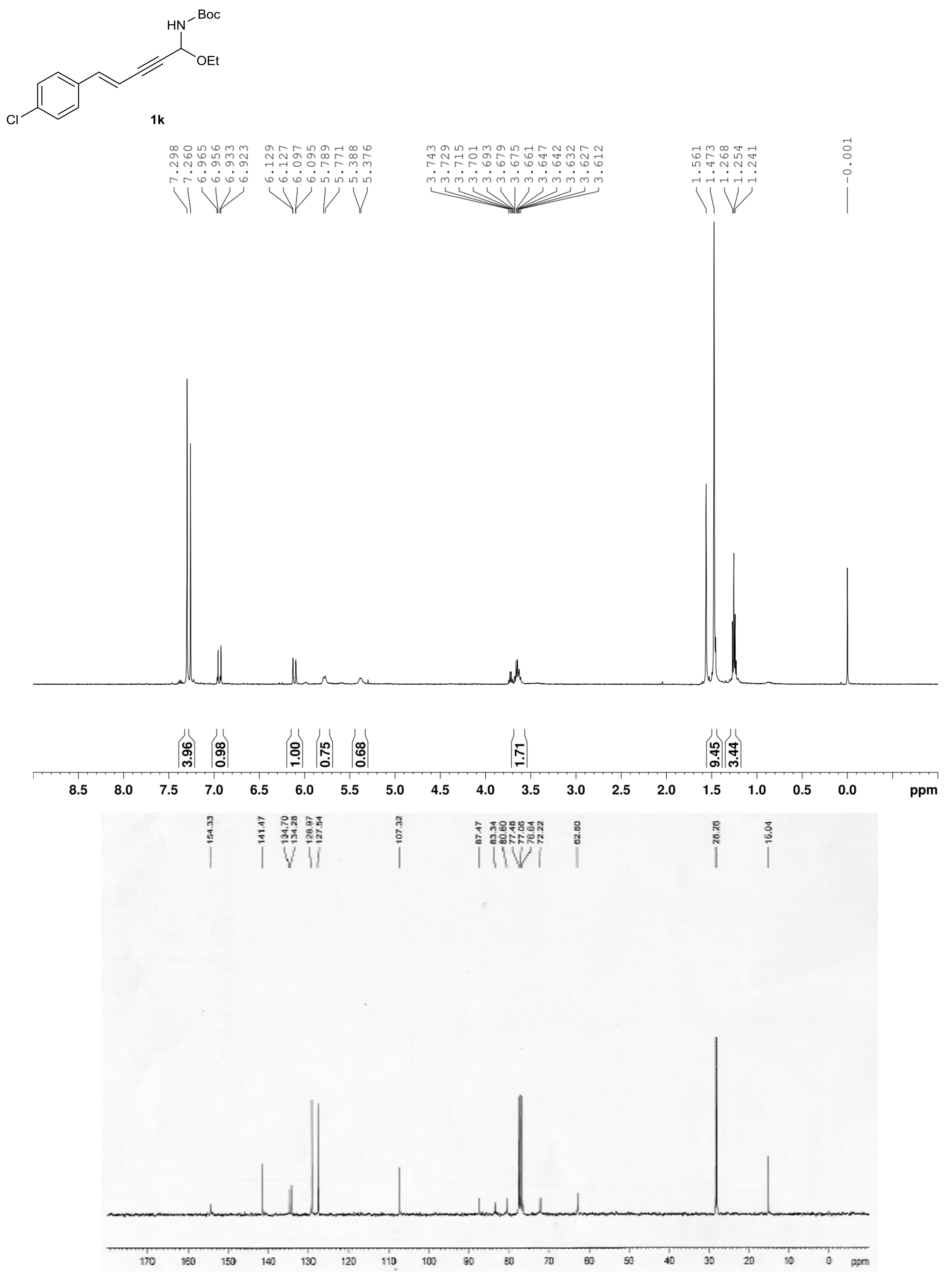

Supplementary Figure 15. ^{1}H and ^{13}C NMR spectra for **1k**.

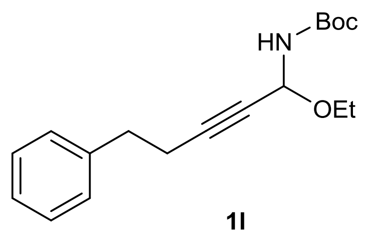

1l

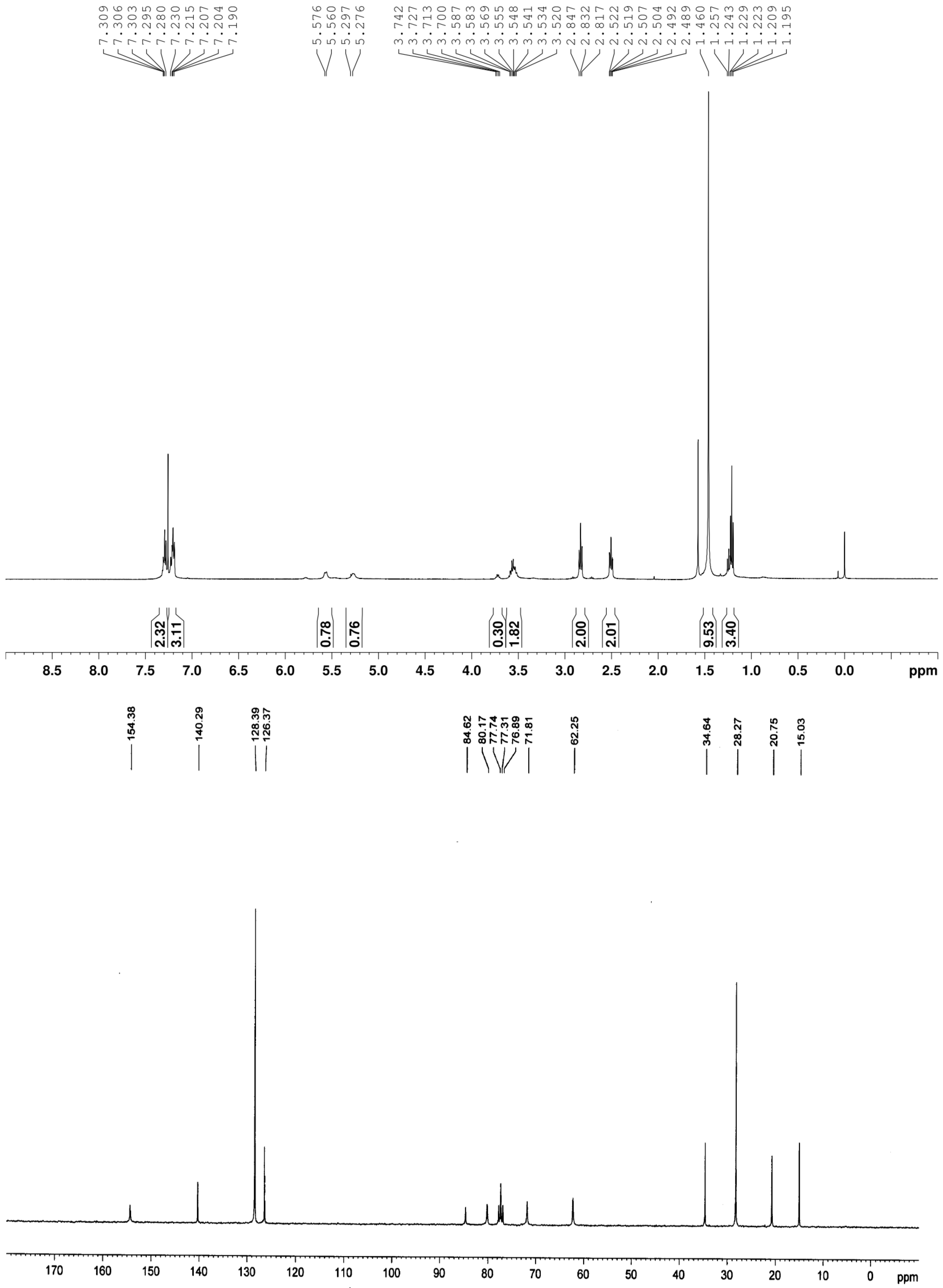

Supplementary Figure 16. ^{1}H and ^{13}C NMR spectra for **1l**.

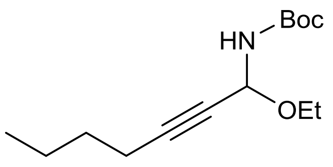

1m

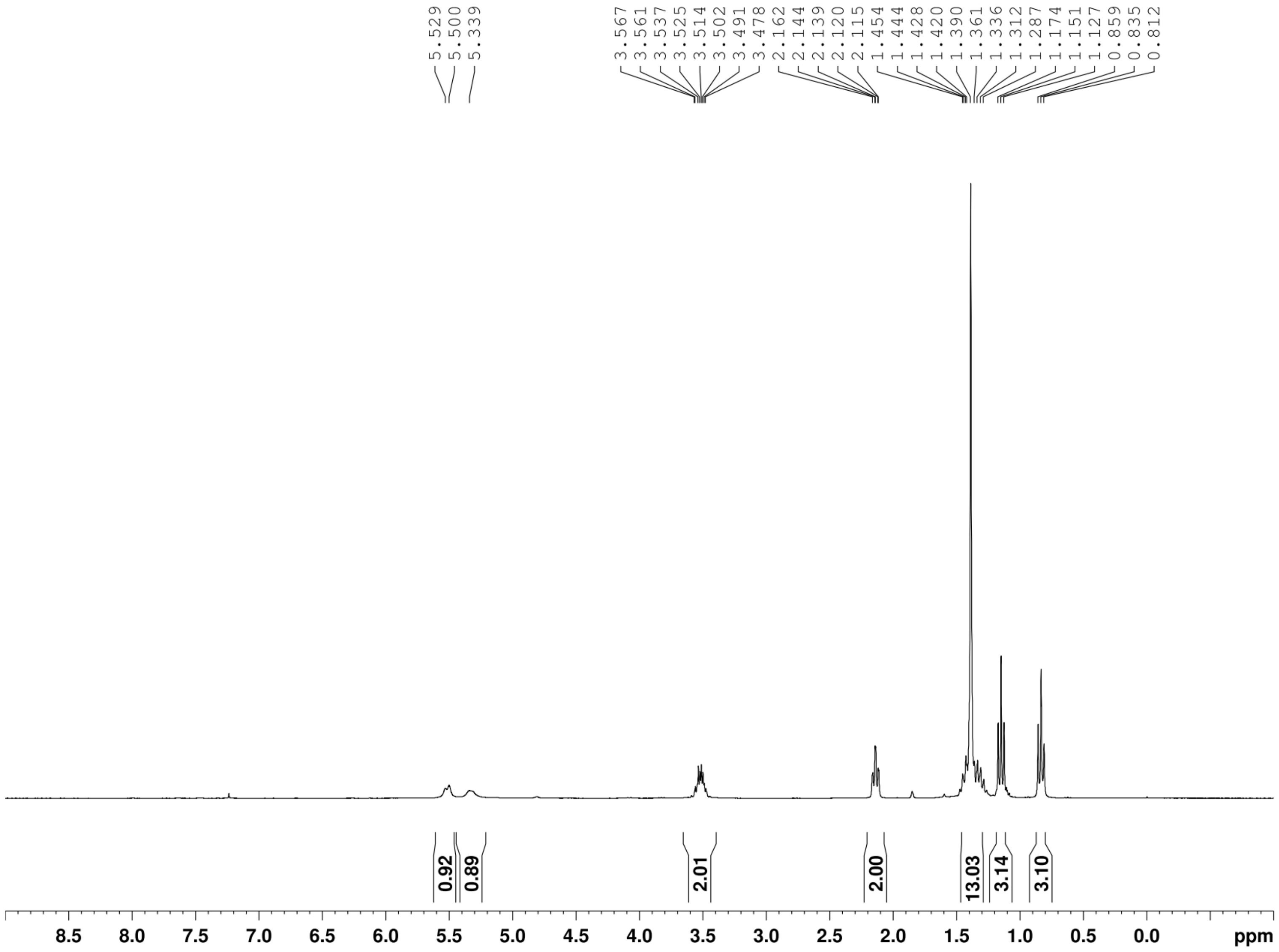

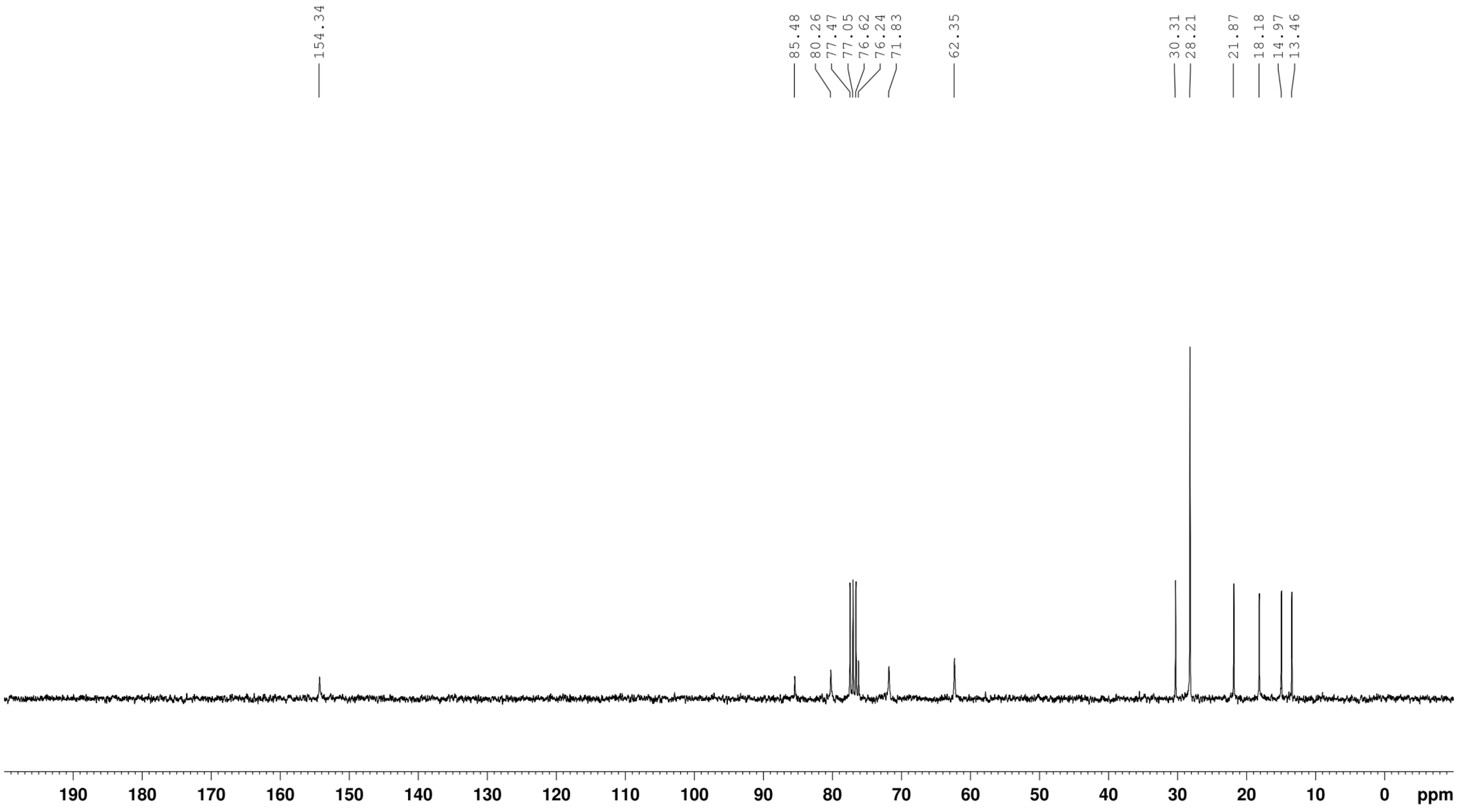

Supplementary Figure 17. ^{1}H and ^{13}C NMR spectra for **1m**.

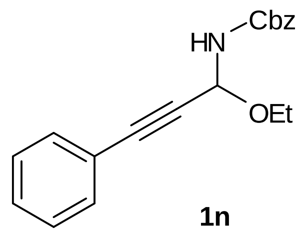

1n

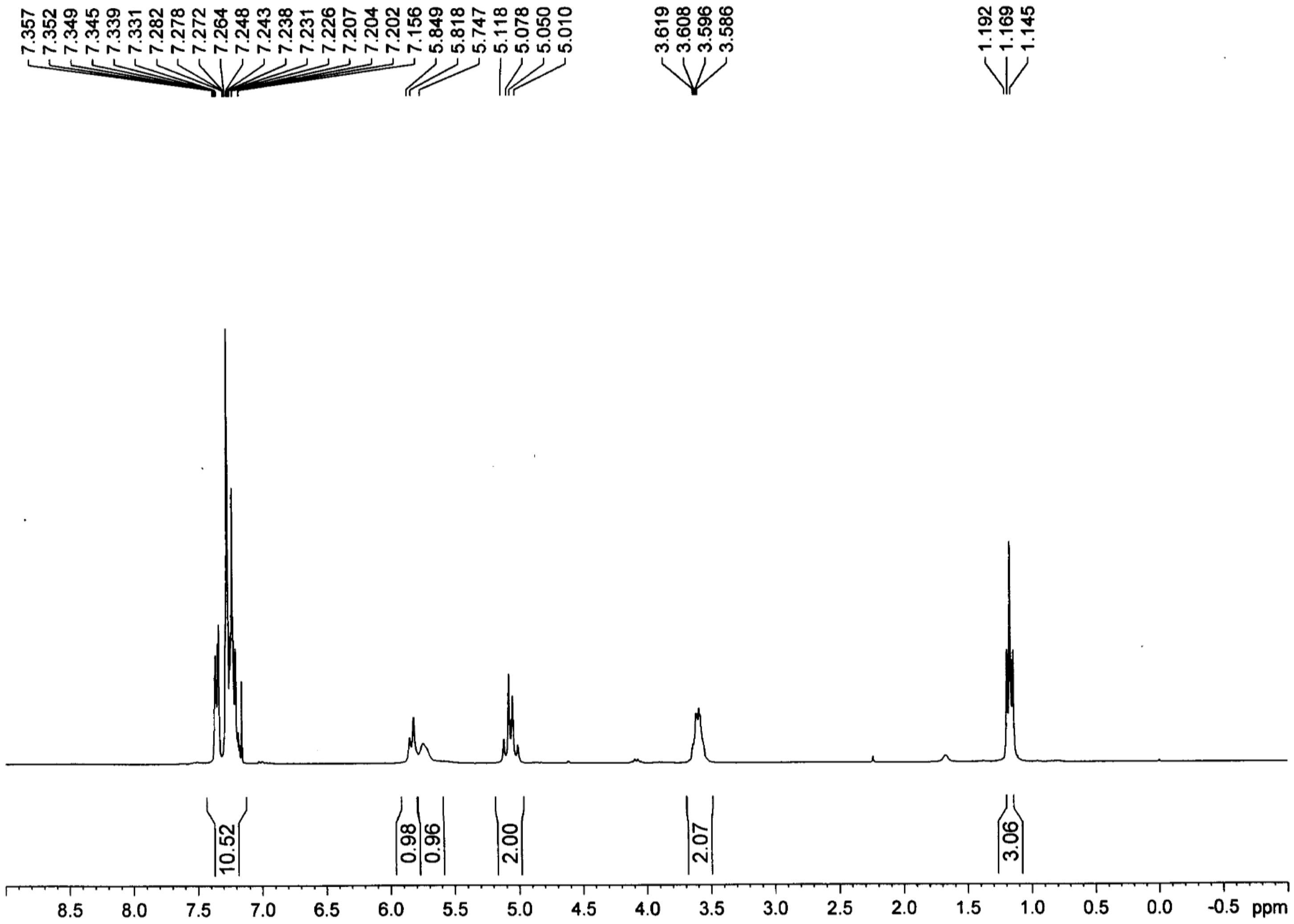

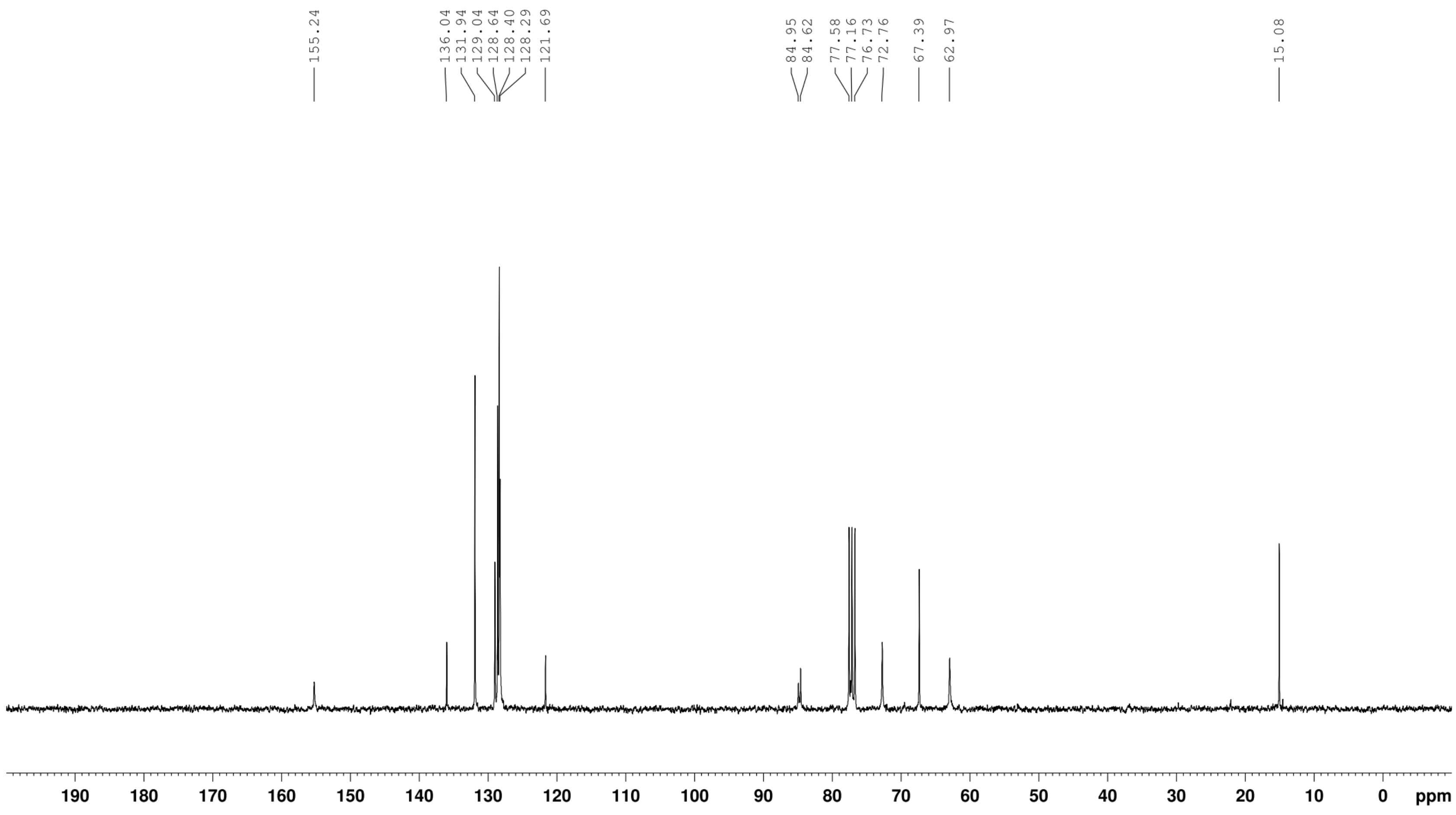

Supplementary Figure 18. ^{1}H and ^{13}C NMR spectra for **1n**.

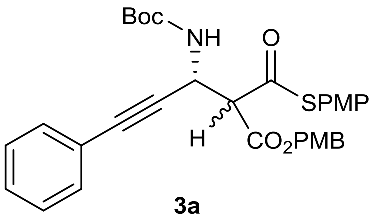

3a

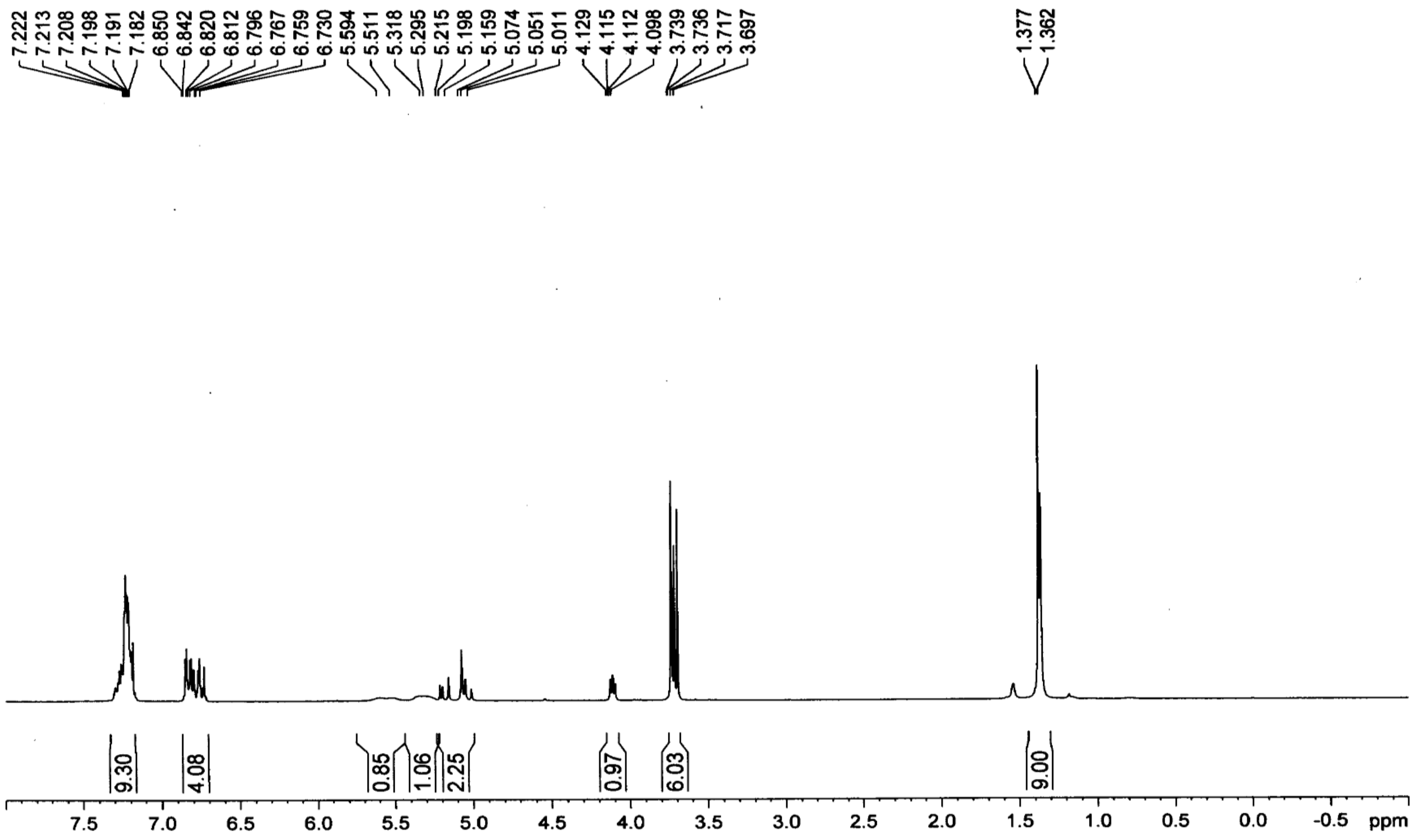

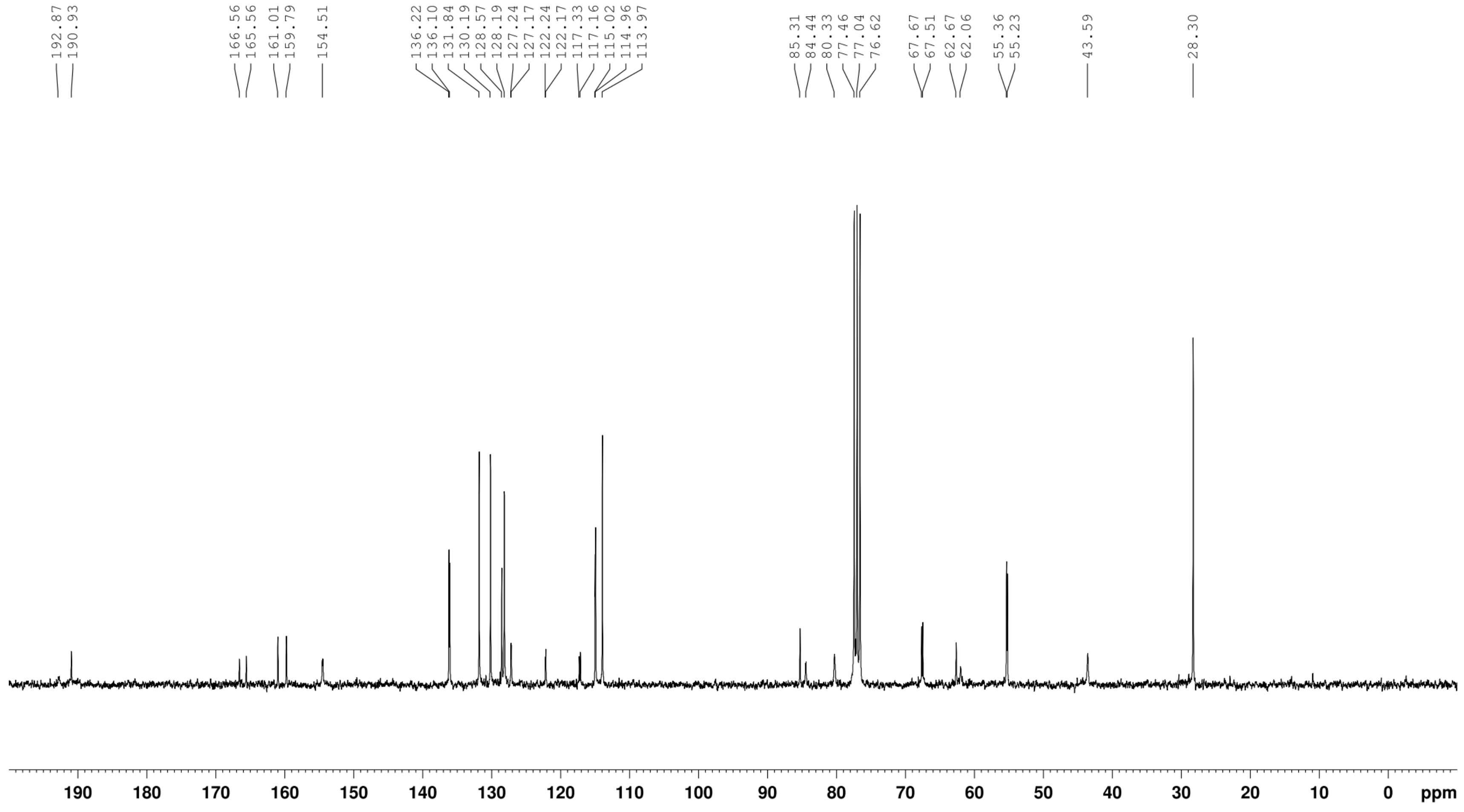

Supplementary Figure 19. ^{1}H and ^{13}C NMR spectra for **3a**.

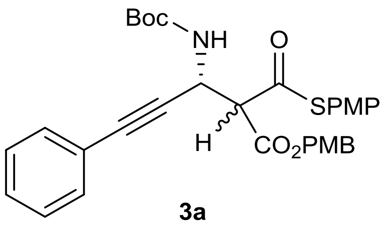

3a

HPLC analysis of **3a**: Daicel CHIRALPAK AD-H, *n*-hexane/*i*-PrOH = 80/20, flow rate = 0.8 mL/min, λ = 254 nm

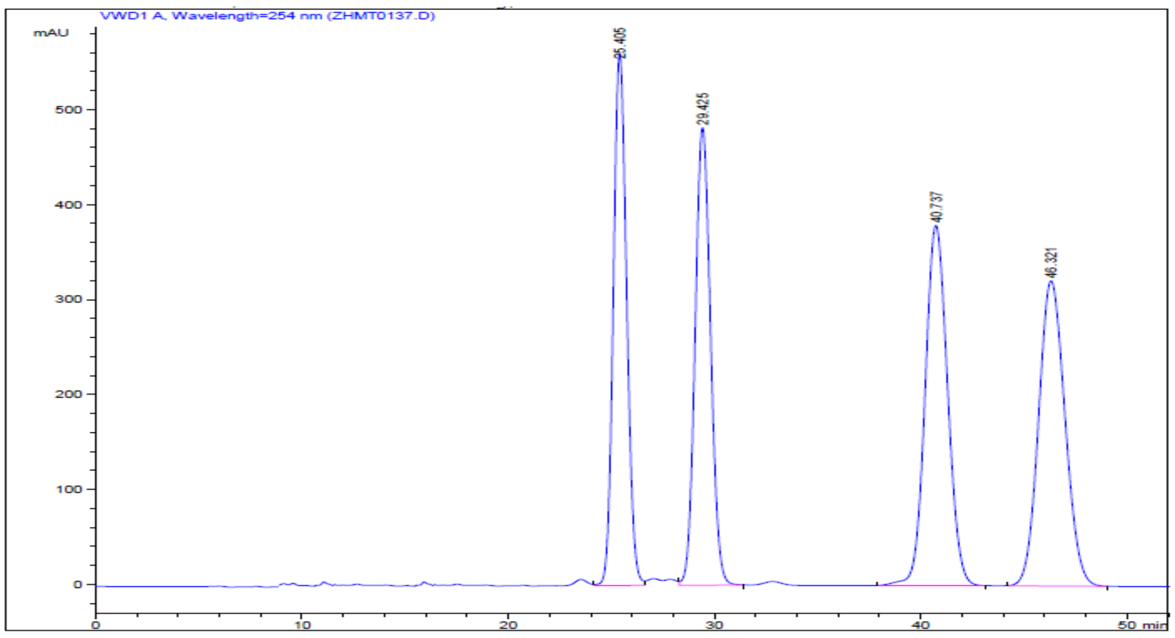

Peak #	RetTime [min]	Type	Width [min]	Area mAU *s	Height [mAU]	Area %
1	25.405	VV	0.7000	2.50805e4	560.15656	23.3541
2	29.425	VB	0.8109	2.50704e4	481.84879	23.3447
3	40.737	BB	1.1877	2.86778e4	379.35965	26.7037
4	46.321	BB	1.3866	2.85638e4	321.29236	26.5975
Totals :				1.07392e5	1742.65735	

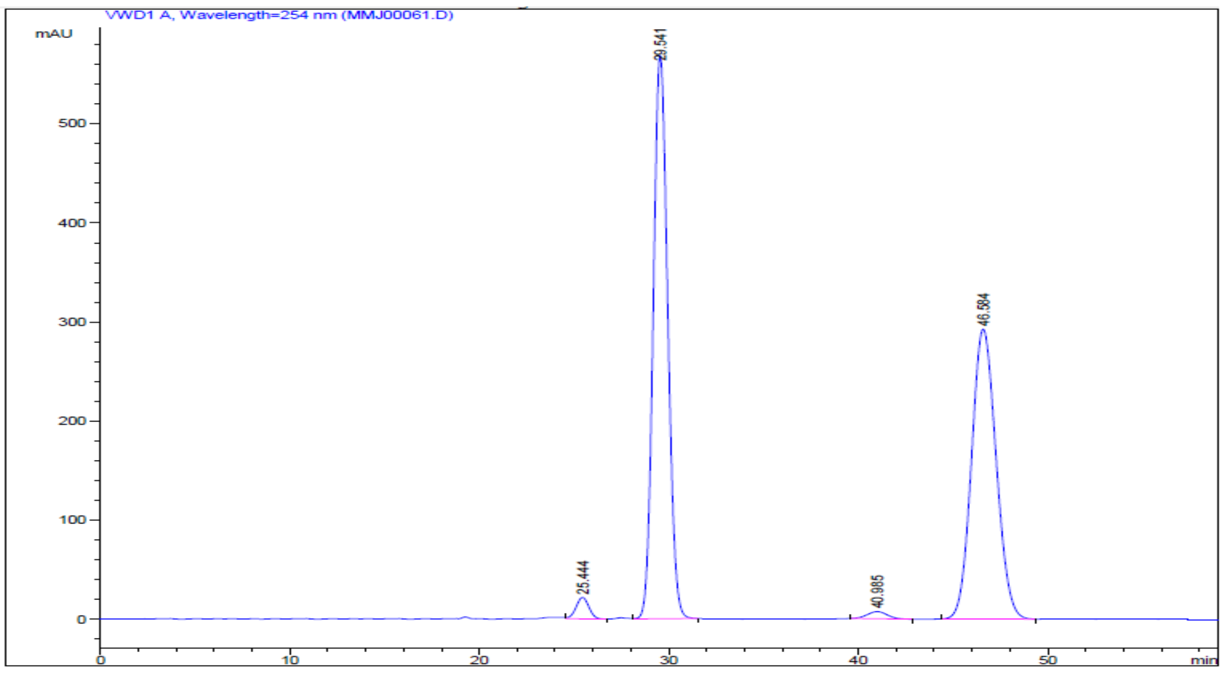

Peak #	RetTime [min]	Type	Width [min]	Area mAU *s	Height [mAU]	Area %
1	25.444	VP	0.7387	1025.64771	21.72257	1.7674
2	29.541	VB	0.8286	3.00810e4	568.31342	51.8352
3	40.985	BP	1.1196	527.06085	7.23243	0.9082
4	46.584	BB	1.4125	2.63983e4	292.52145	45.4892
Totals :				5.80319e4	889.78987	

Supplementary Figure 20. HPLC spectra for **3a**.

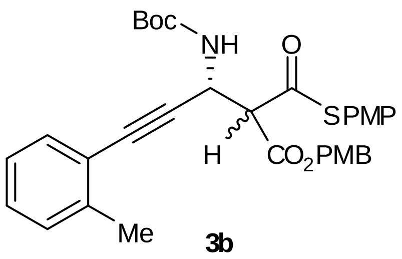

3b

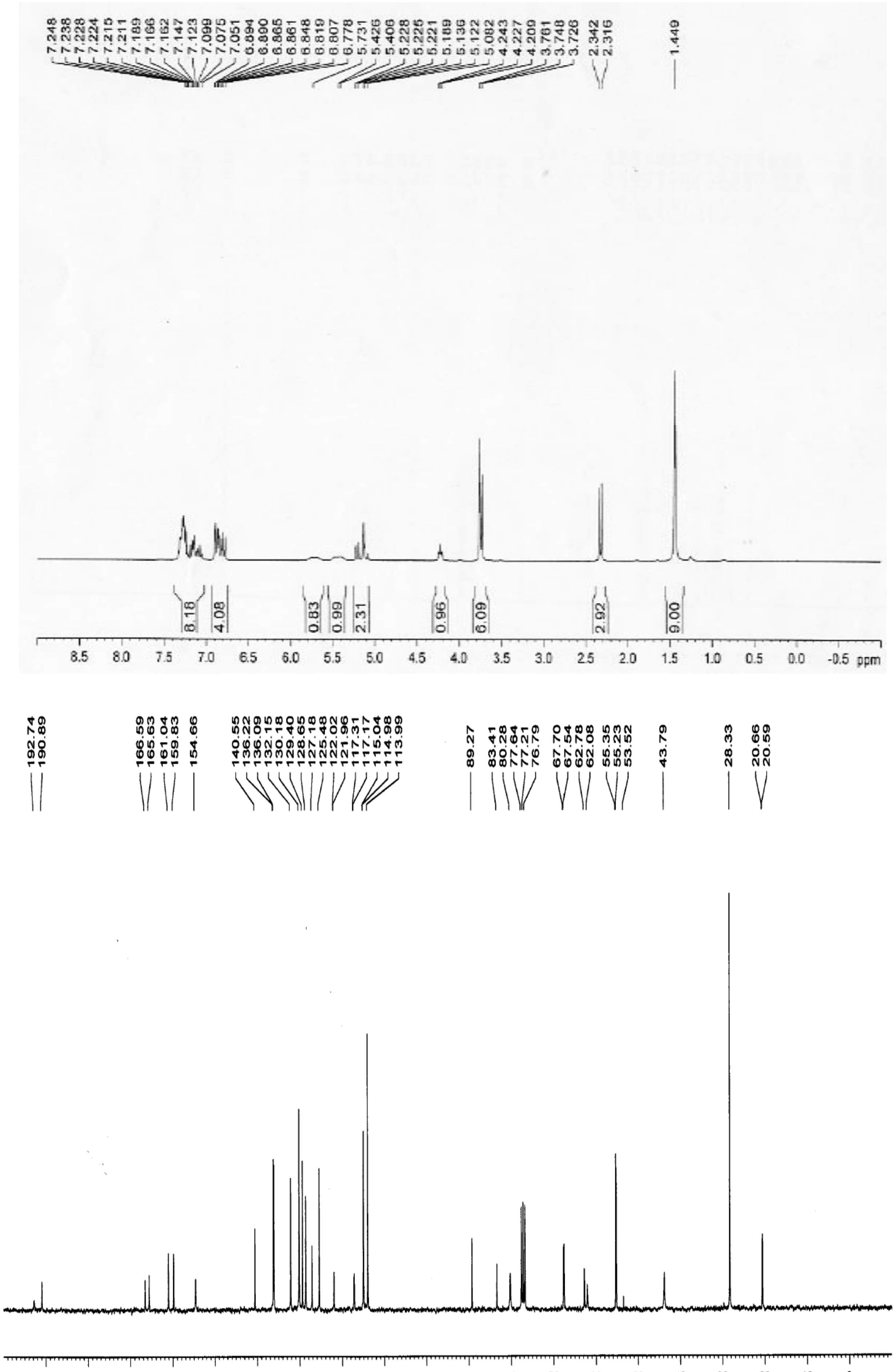

Supplementary Figure 21. ^{1}H and ^{13}C NMR spectra for **3b**.

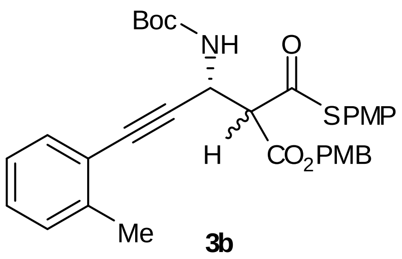

3b

HPLC analysis of **3b**: Daicel CHIRALPAK AD-H, *n*-hexane/*i*-PrOH = 80/20, flow rate = 0.8 mL/min, λ = 254 nm

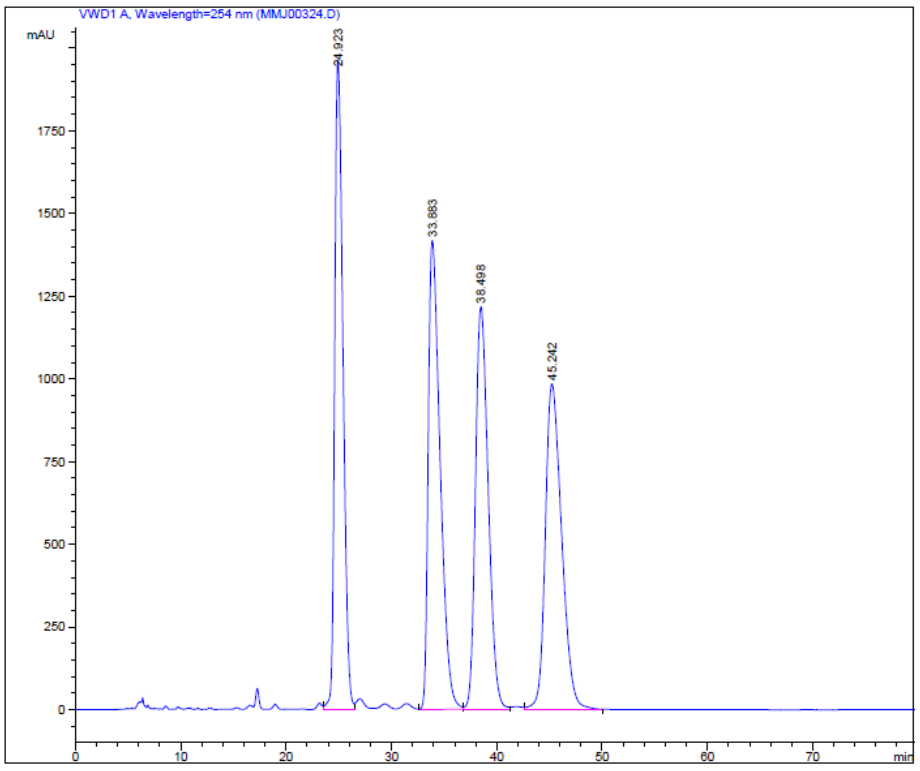

Peak #	RetTime [min]	Type	Width [min]	Area mAU *s	Height [mAU]	Area %
1	24.923	VV	0.8549	1.07055e5	1962.22546	25.1324
2	33.883	VV	1.1933	1.10672e5	1417.54651	25.9814
3	38.498	VB	1.3202	1.02498e5	1217.18359	24.0626
4	45.242	VB	1.6731	1.05740e5	983.91809	24.8236
Totals :				4.25965e5	5580.87366	

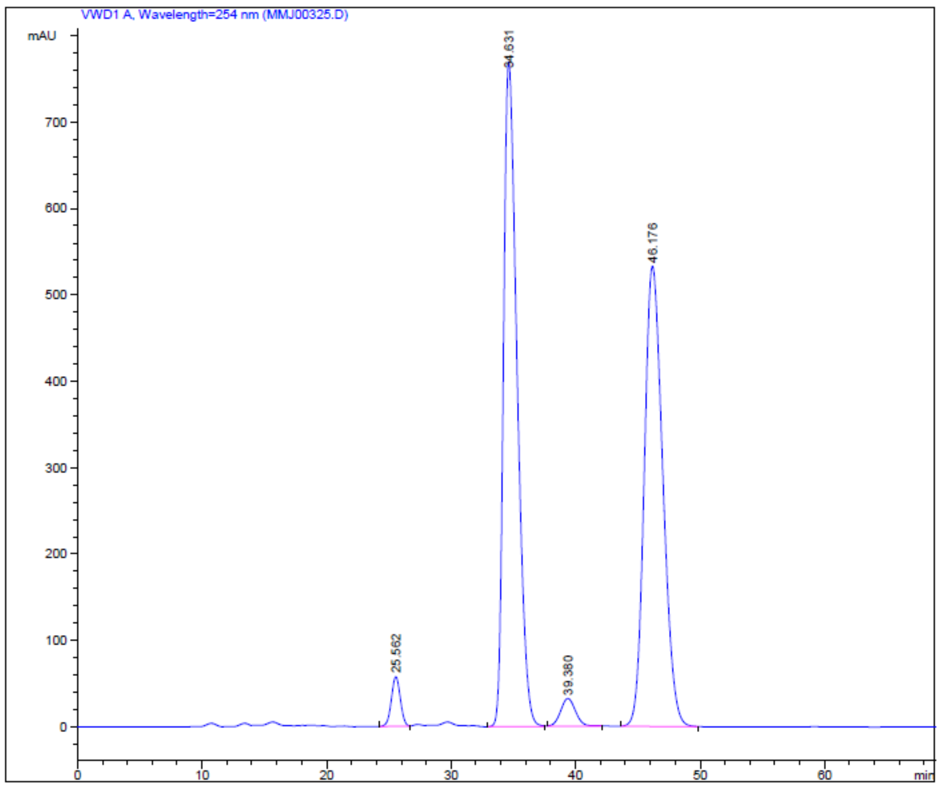

Peak #	RetTime [min]	Type	Width [min]	Area mAU *s	Height [mAU]	Area %
1	25.562	BV	0.7822	2894.33569	57.52042	2.4350
2	34.631	PB	1.1663	5.80953e4	769.41449	48.8752
3	39.380	BB	1.2881	2693.17041	32.17680	2.2657
4	46.176	BB	1.6097	5.51818e4	532.98816	46.4241
Totals :				1.18865e5	1392.09986	

Supplementary Figure 22. HPLC spectra for **3b**.

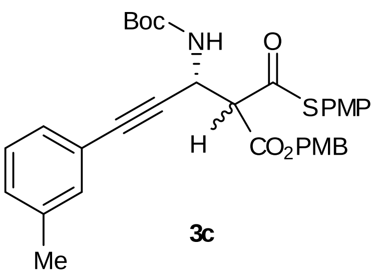

3c

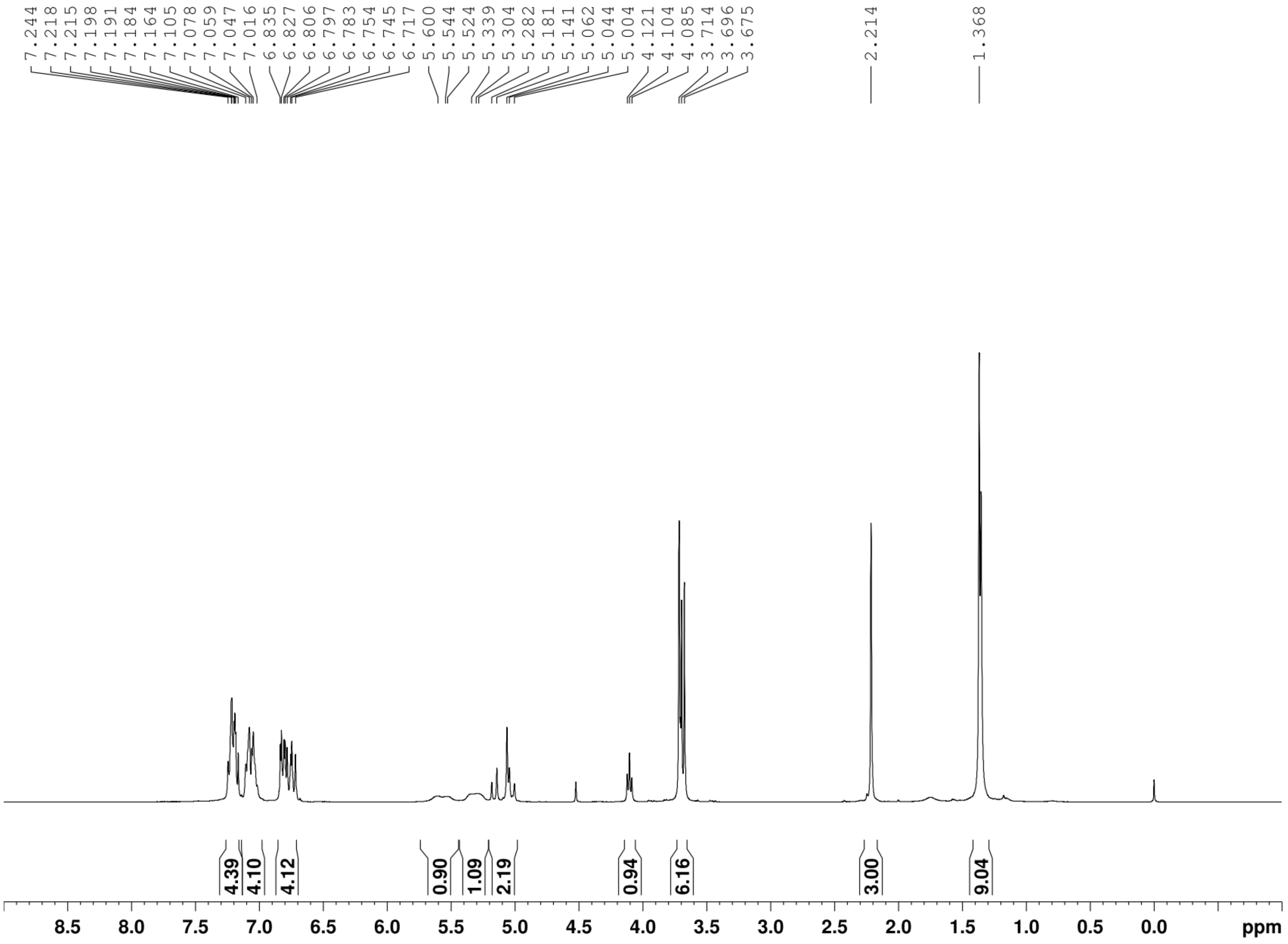

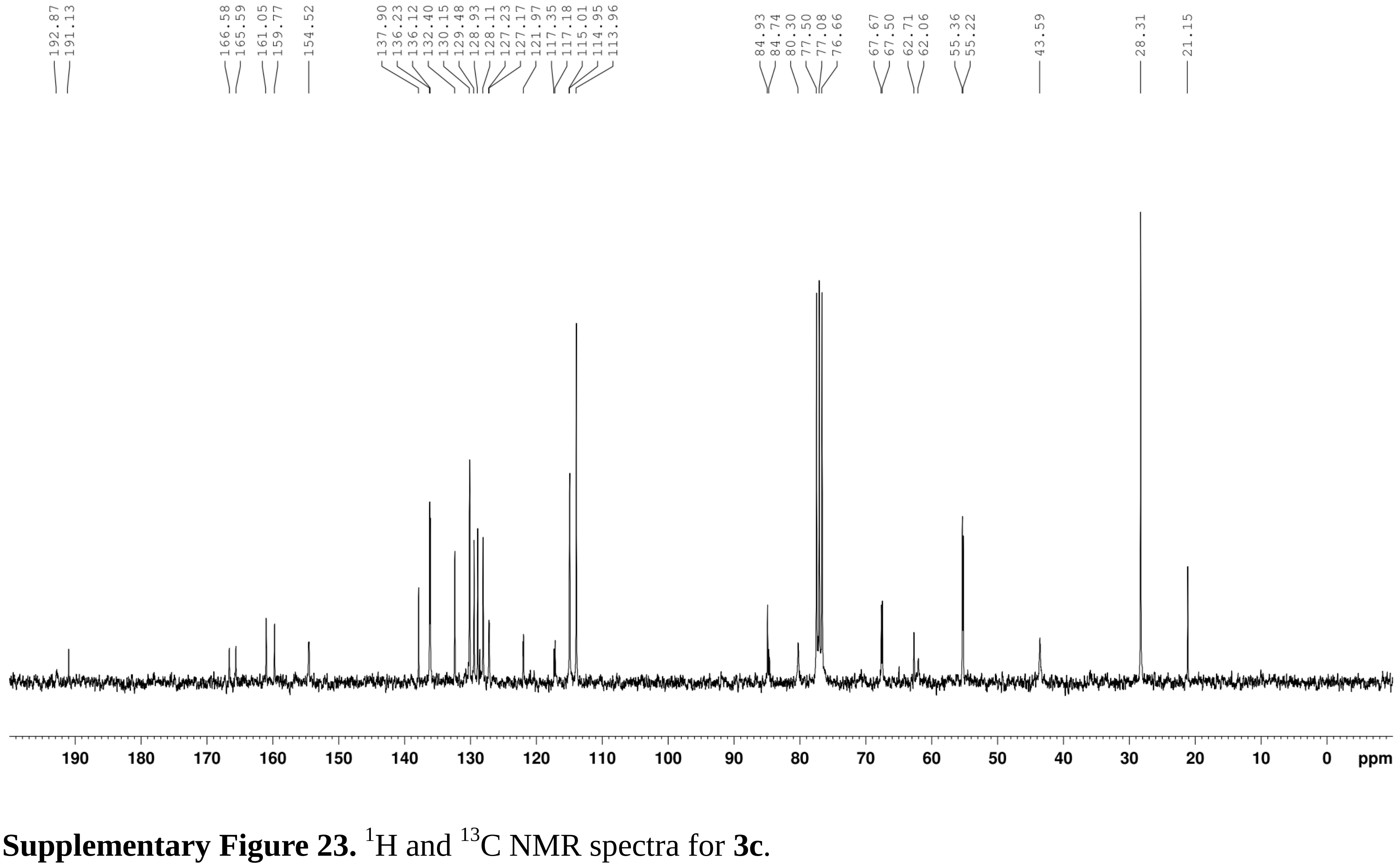

Supplementary Figure 23. ^{1}H and ^{13}C NMR spectra for **3c**.

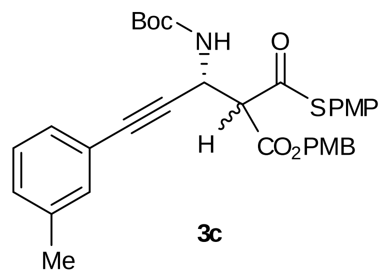

3c

HPLC analysis of **3c**: Daicel CHIRALPAK AD-H, *n*-hexane/*i*-PrOH = 80/20, flow rate = 0.8 mL/min, λ = 254 nm

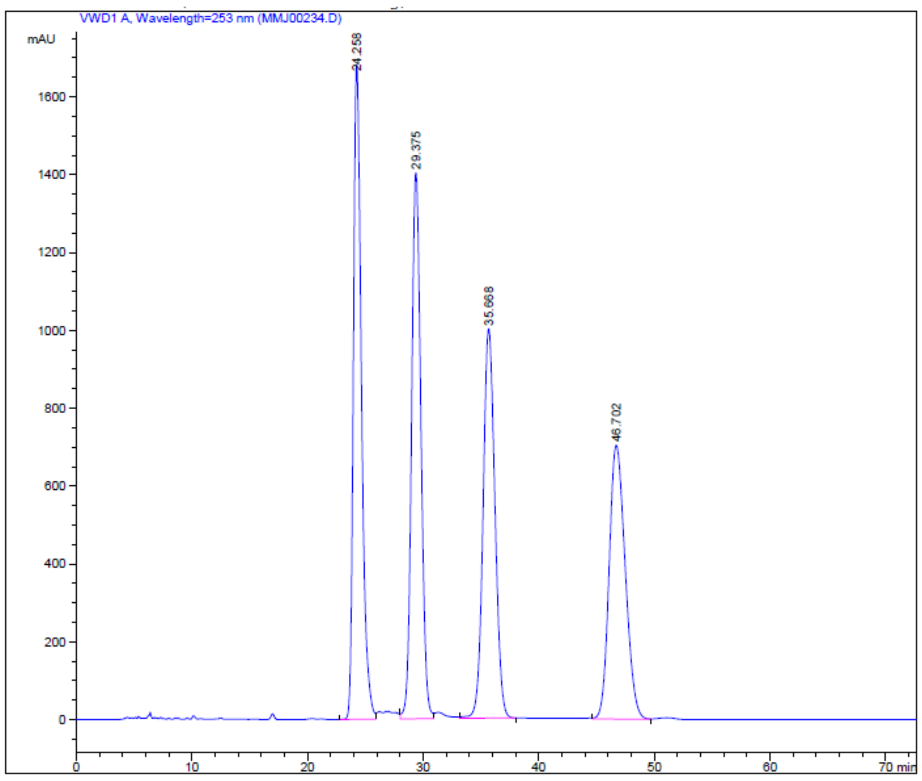

Peak #	RetTime [min]	Type	Width [min]	Area mAU *s	Height [mAU]	Area %
1	24.258	BV	0.7453	8.13743e4	1681.04492	27.1872
2	29.375	VV	0.8777	7.88918e4	1402.26599	26.3578
3	35.668	BB	1.1022	7.11354e4	999.95496	23.7664
4	46.702	VB	1.5123	6.79097e4	703.32019	22.6887
Totals :				2.99311e5	4786.58606	

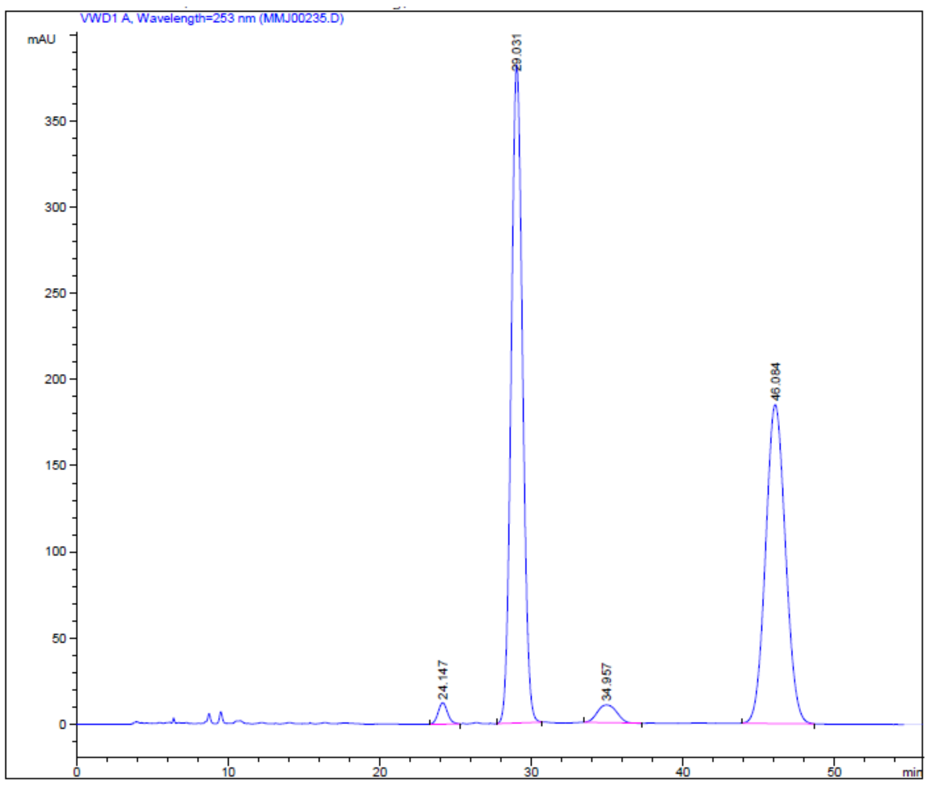

Peak #	RetTime [min]	Type	Width [min]	Area mAU *s	Height [mAU]	Area %
1	24.147	BB	0.6686	540.57410	12.40329	1.4155
2	29.031	BB	0.8127	1.98759e4	381.79446	52.0444
3	34.957	BP	1.2669	907.22607	10.28033	2.3755
4	46.084	BB	1.4269	1.68665e4	185.12766	44.1646
Totals :				3.81902e4	589.60573	

Supplementary Figure 24. HPLC spectra for **3c**.

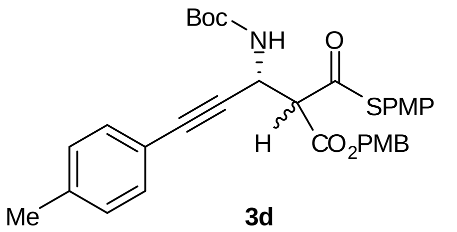

3d

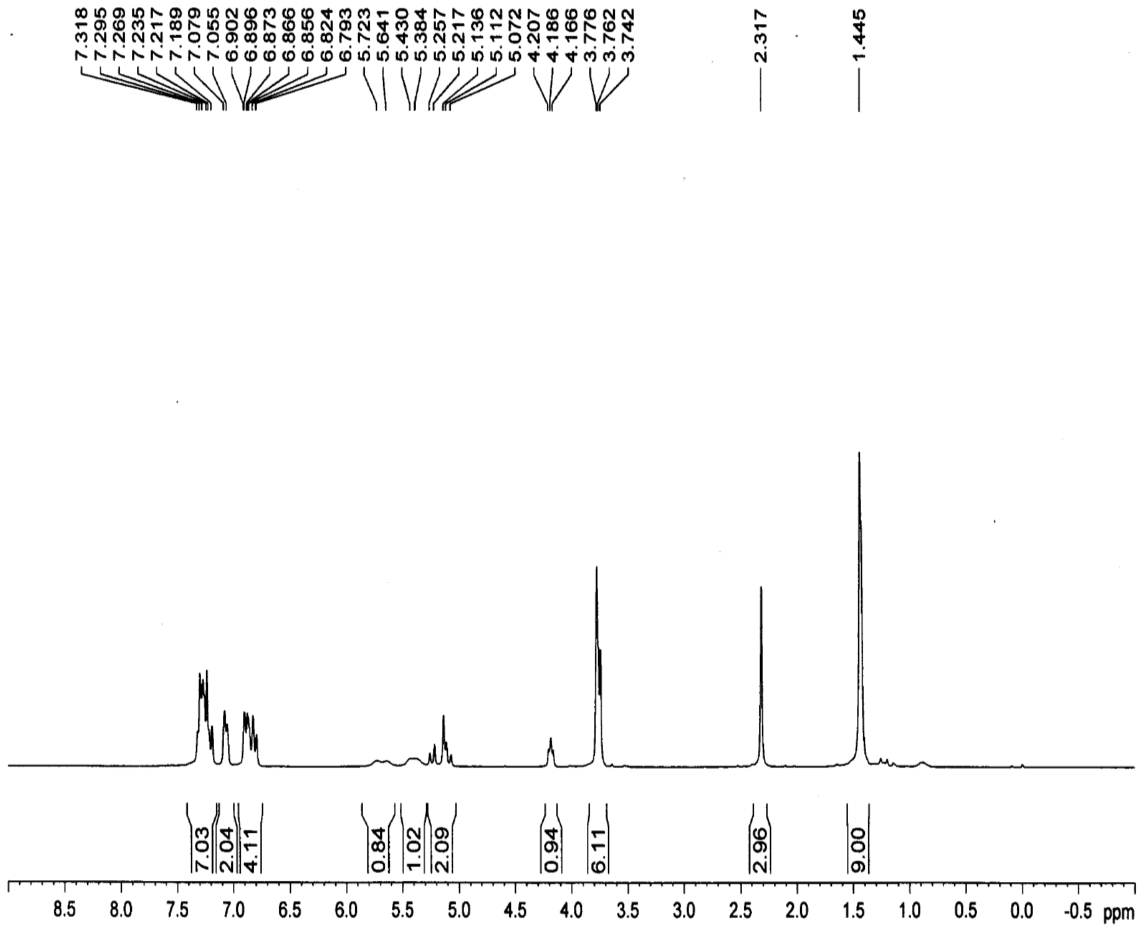

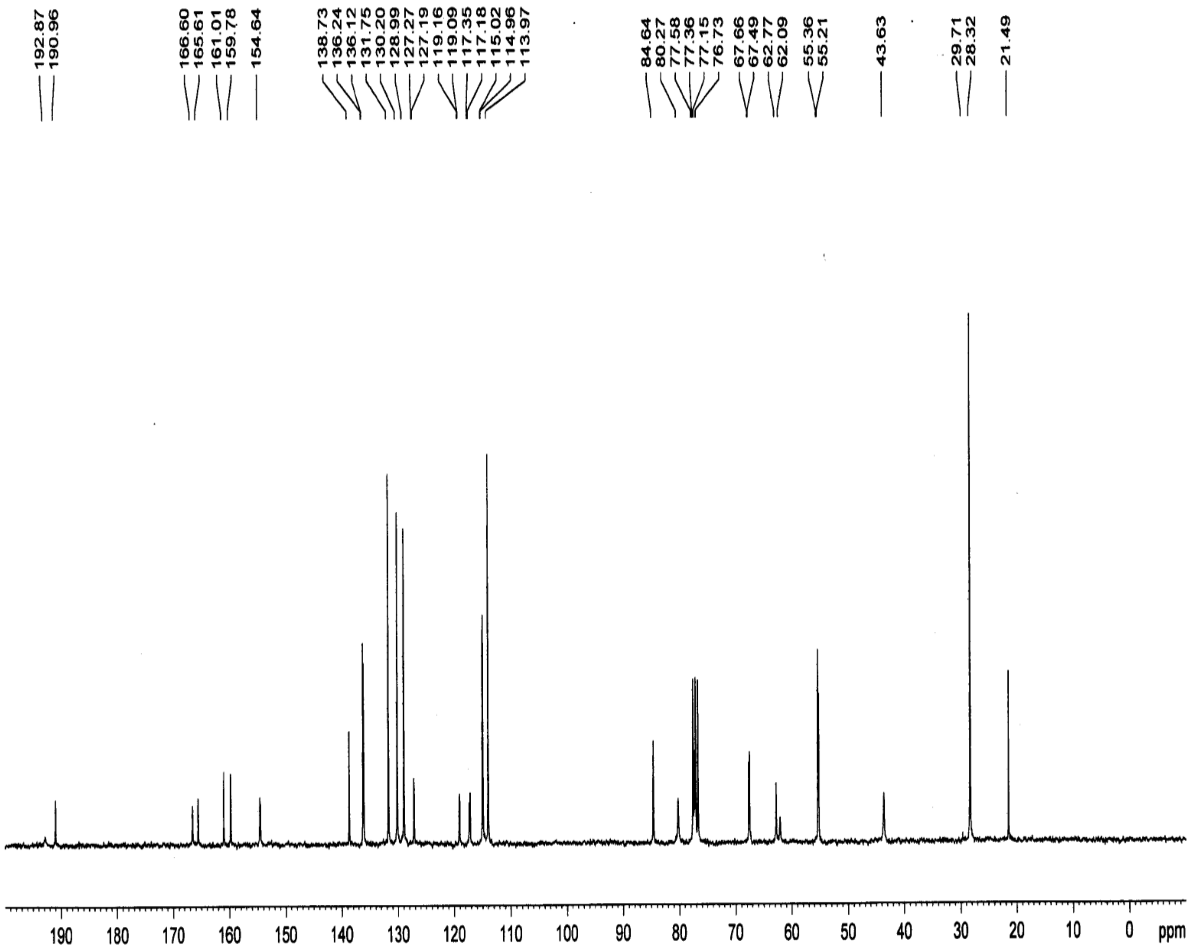

Supplementary Figure 25. ^{1}H and ^{13}C NMR spectra for **3d**.

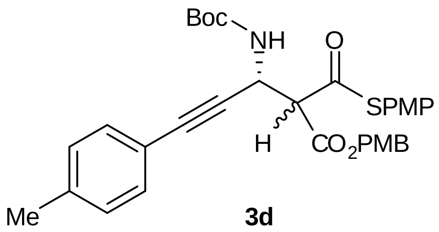

3d

HPLC analysis of **3d**: Daicel CHIRALPAK AD-H, *n*-hexane/*i*-PrOH = 80/20, flow rate = 0.8 mL/min, λ = 254 nm

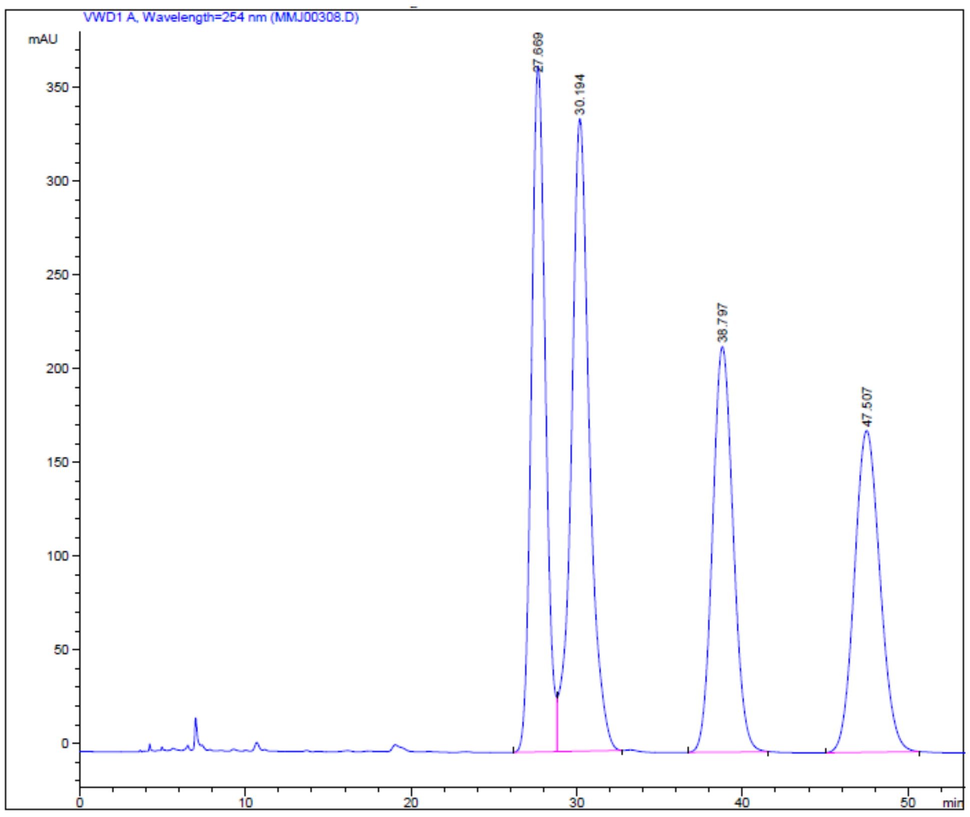

Peak #	RetTime [min]	Type	Width [min]	Area mAU *s	Height [mAU]	Area %
1	27.669	BV	0.9181	2.15700e4	365.76056	25.6636
2	30.194	VB	1.1247	2.54507e4	337.37851	30.2807
3	38.797	BB	1.3401	1.86558e4	216.20853	22.1963
4	47.507	BB	1.6542	1.83727e4	171.55006	21.8595
Totals :				8.40491e4	1090.89766	

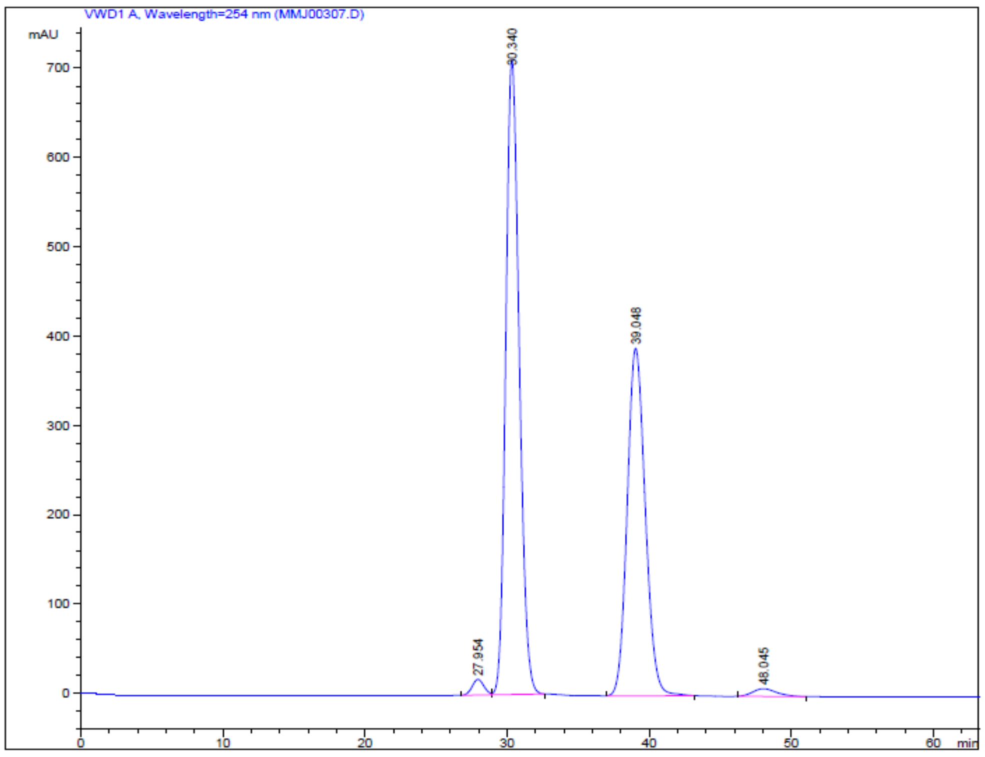

Peak #	RetTime [min]	Type	Width [min]	Area mAU *s	Height [mAU]	Area %
1	27.954	BV	0.8953	1009.95721	17.55847	1.2143
2	30.340	VB	1.0180	4.67824e4	711.06952	56.2478
3	39.048	BB	1.3694	3.43686e4	389.21899	41.3223
4	48.045	BB	1.4228	1011.03925	8.65344	1.2156
Totals :				8.31721e4	1126.50043	

Supplementary Figure 26. HPLC spectra for **3d**.

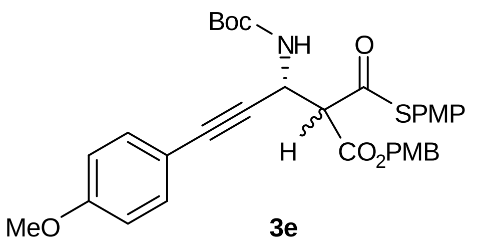

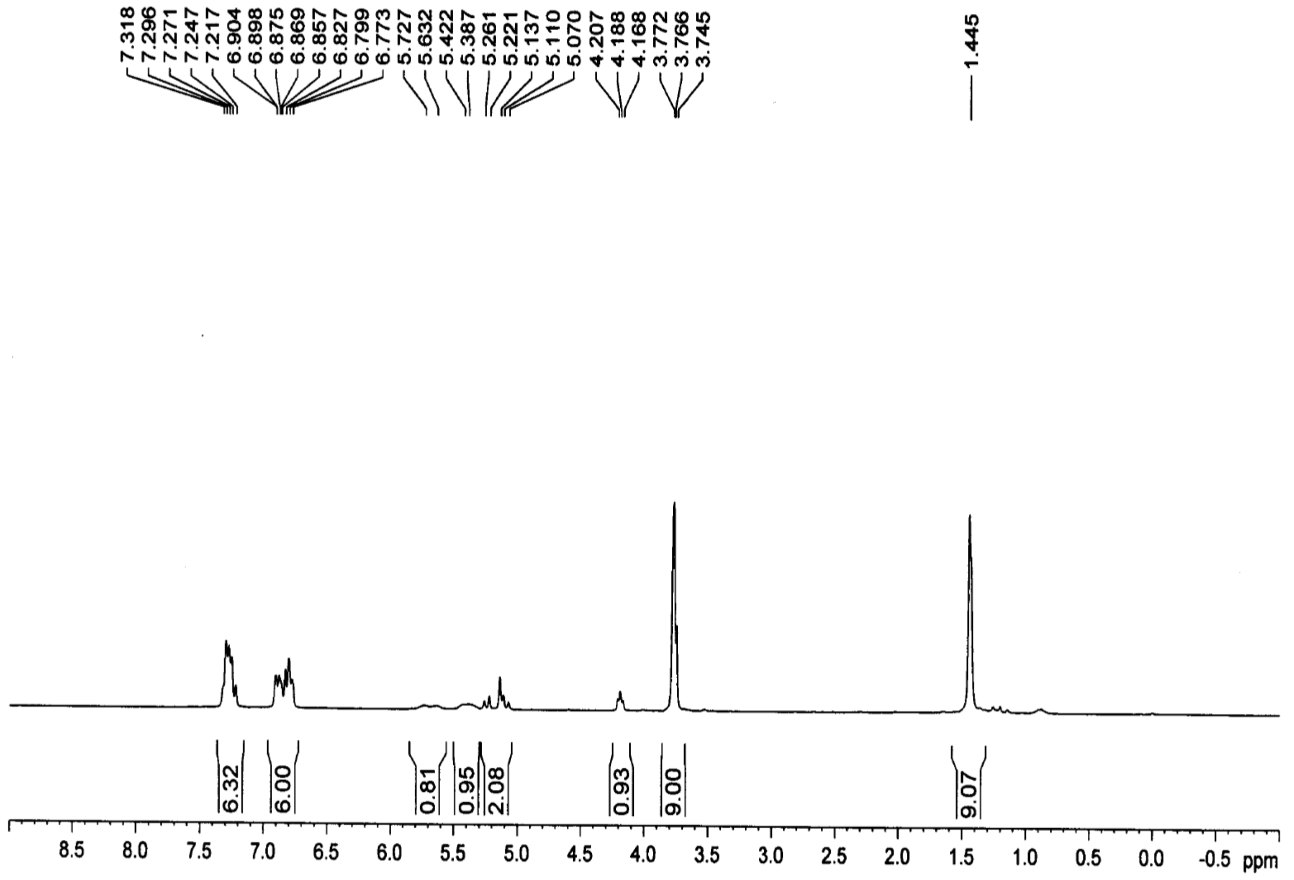

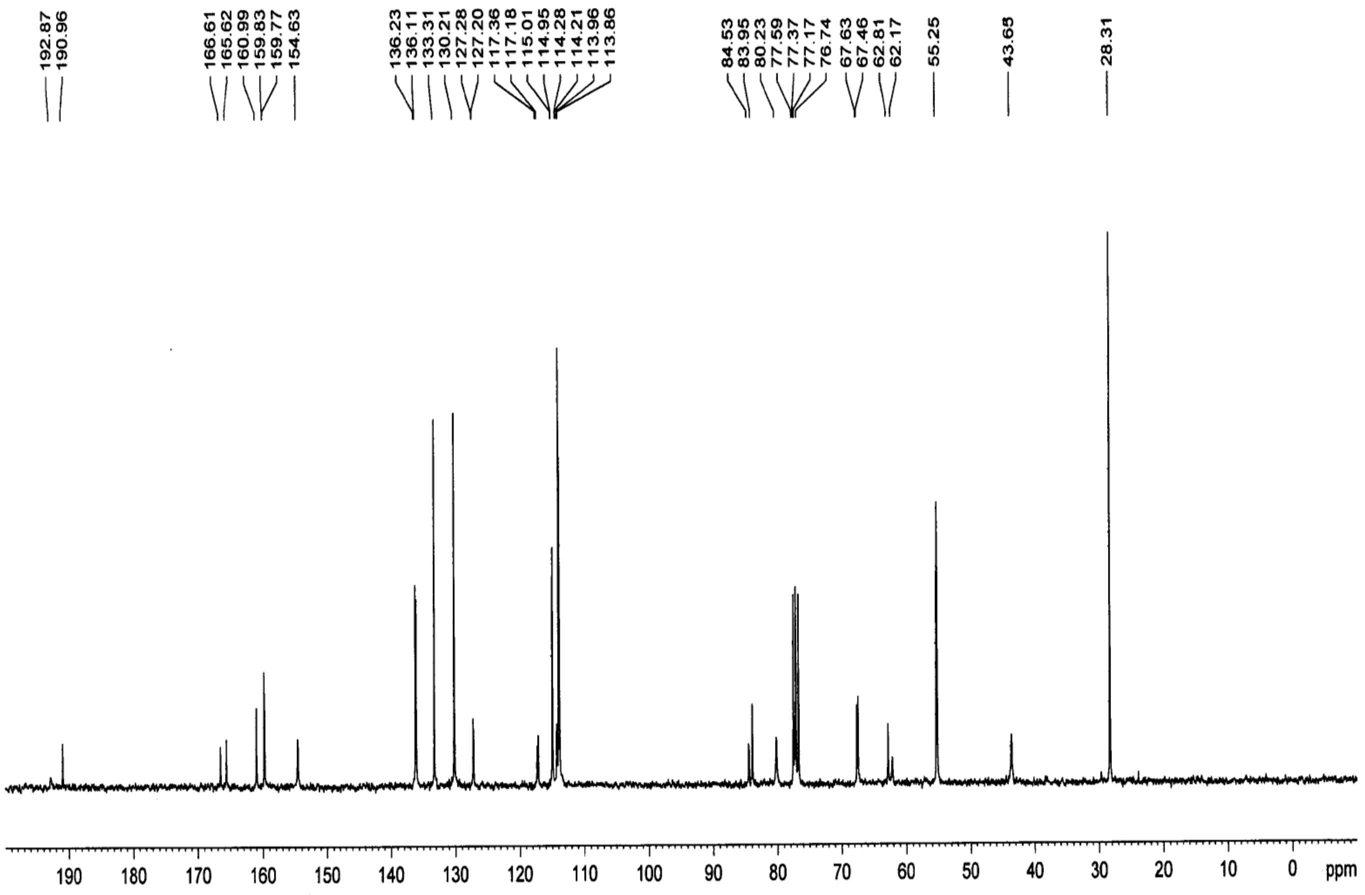

Supplementary Figure 27. ^{1}H and ^{13}C NMR spectra for **3e**.

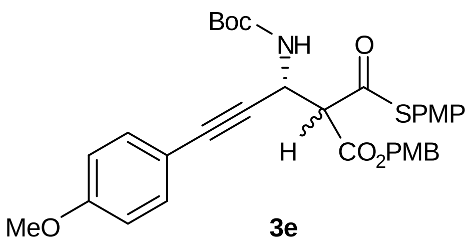

3e

HPLC analysis of **3e**: Daicel CHIRALPAK AD-H, *n*-hexane/*i*-PrOH = 80/20, flow rate = 0.8 mL/min, λ = 254 nm

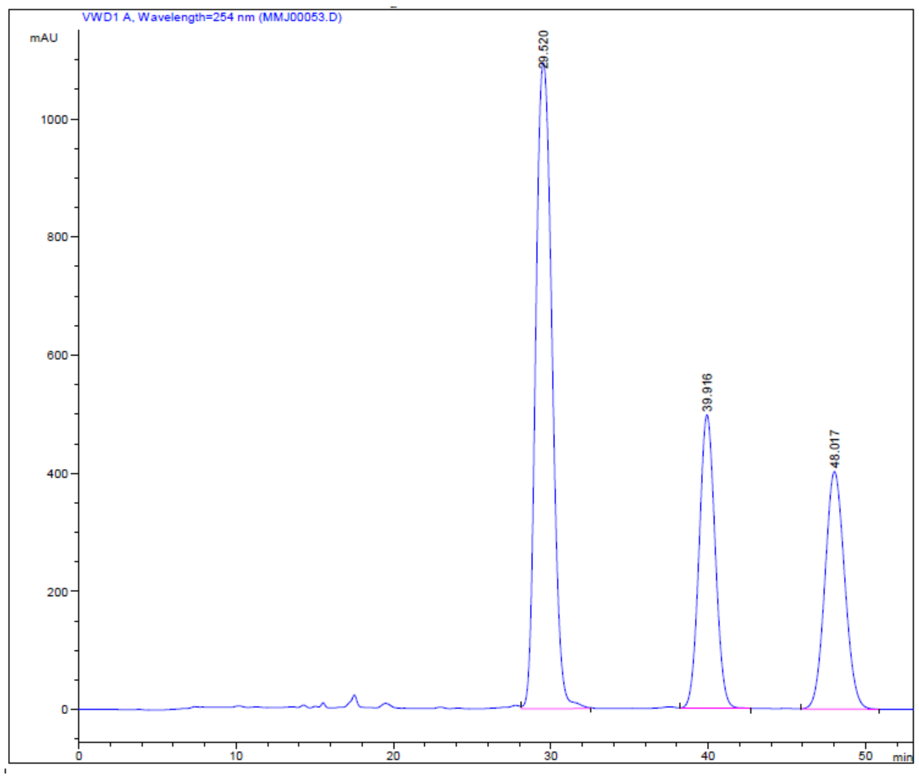

Peak #	RetTime [min]	Type	Width [min]	Area mAU *s	Height [mAU]	Area %
1	29.520	VV	1.1584	7.88401e4	1093.71985	52.8710
2	39.916	VB	1.1002	3.52157e4	497.11157	23.6160
3	48.017	PB	1.3605	3.50621e4	402.27924	23.5130
Totals :				1.49118e5	1993.11066	

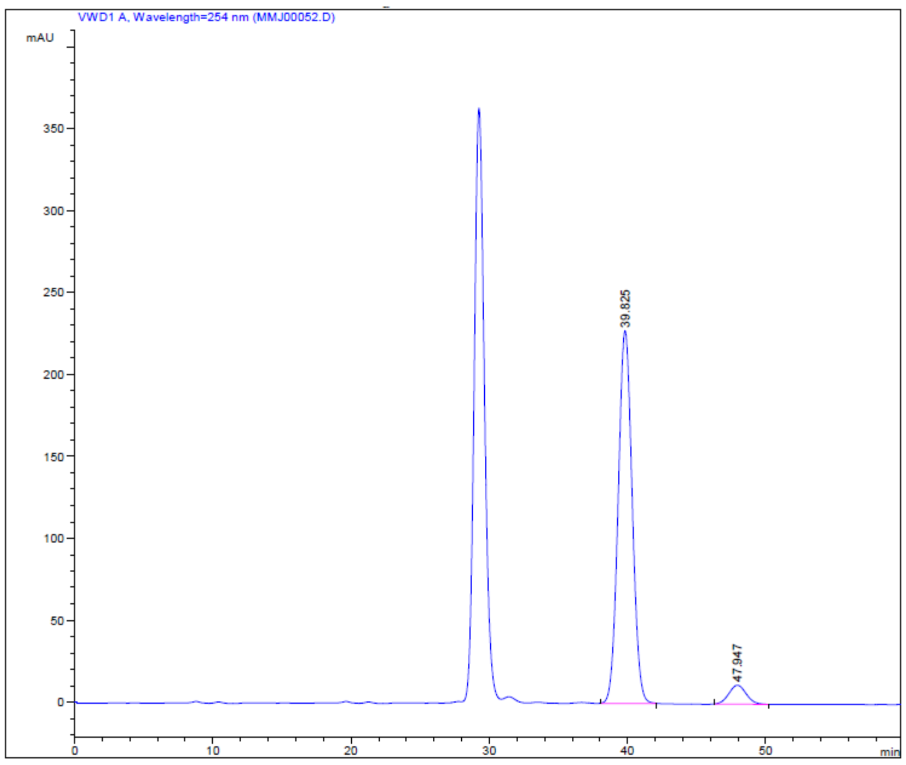

Peak #	RetTime [min]	Type	Width [min]	Area mAU *s	Height [mAU]	Area %
1	39.825	BB	1.0966	1.59902e4	227.52809	94.0186
2	47.947	BB	1.2588	1017.27972	11.60268	5.9814
Totals :				1.70075e4	239.13077	

Supplementary Figure 28. HPLC spectra for **3e**.

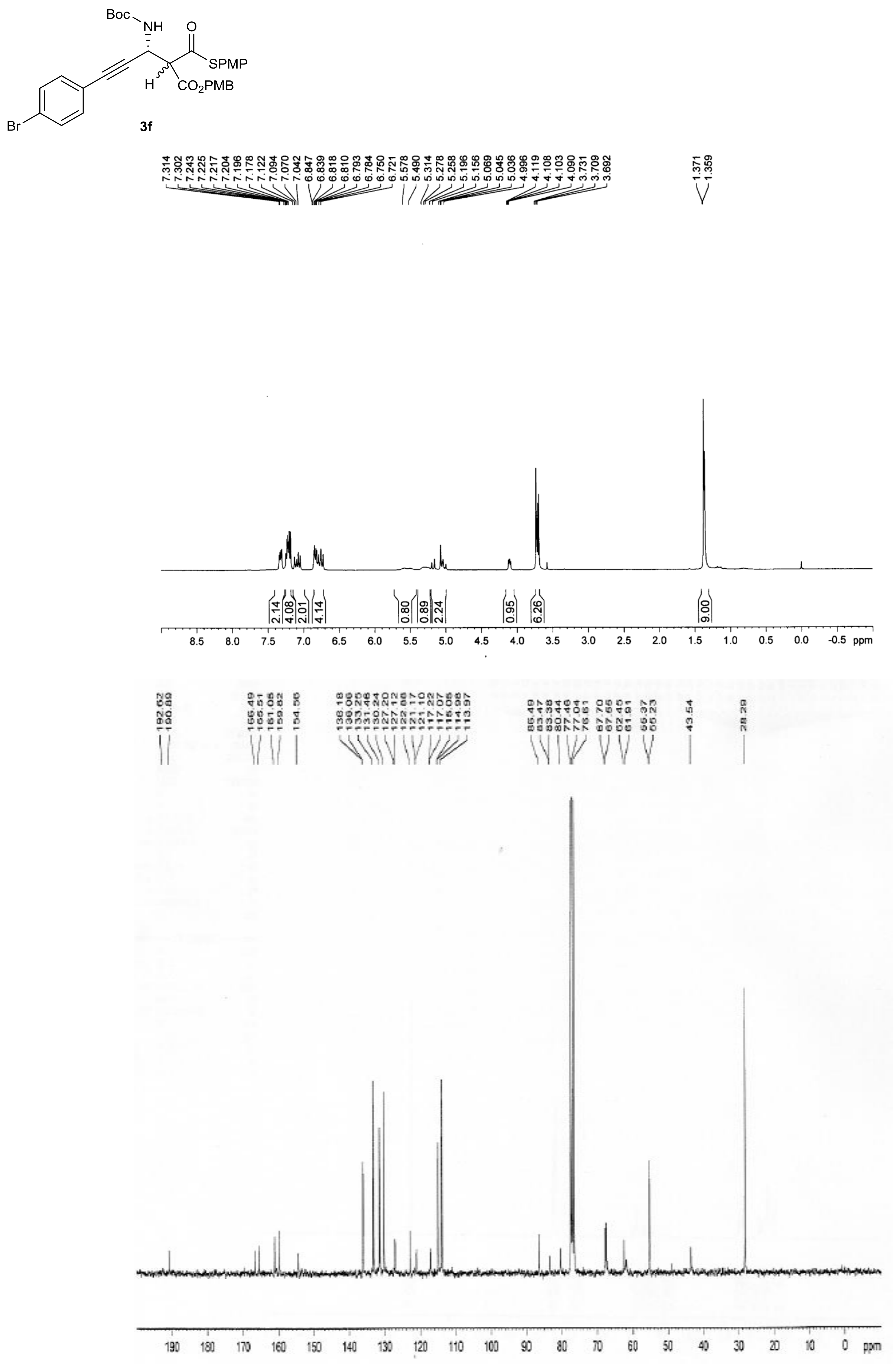

Supplementary Figure 29. ^{1}H and ^{13}C NMR spectra for **3f**.

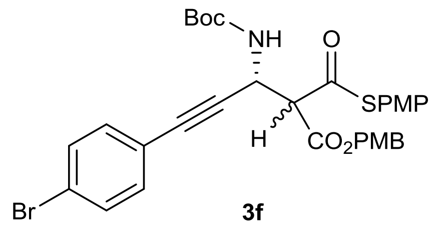

3f

HPLC analysis of **3f**: Daicel CHIRALPAK AD-H, *n*-hexane/*i*-PrOH = 80/20, flow rate = 0.8 mL/min, λ = 254 nm

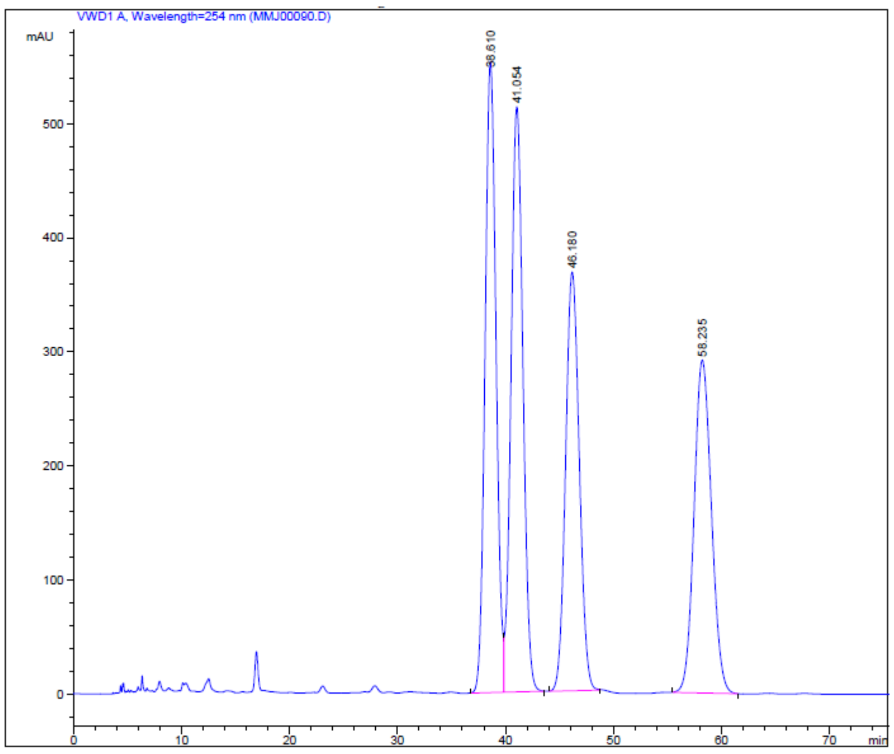

Peak #	RetTime [min]	Type	Width [min]	Area mAU *s	Height [mAU]	Area %
1	38.610	BV	1.1017	3.90946e4	553.76605	27.2645
2	41.054	VB	1.1967	3.93970e4	513.38348	27.4754
3	46.180	BB	1.3871	3.23027e4	367.30963	22.5278
4	58.235	BB	1.7373	3.25959e4	292.09839	22.7323
Totals :				1.43390e5	1726.55756	

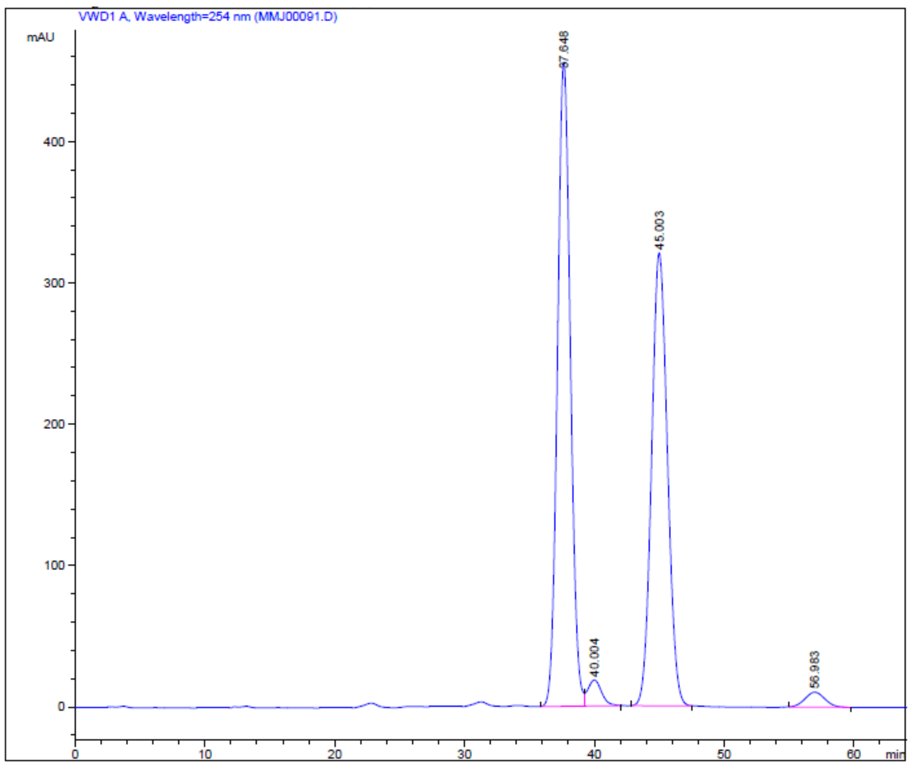

Peak #	RetTime [min]	Type	Width [min]	Area mAU *s	Height [mAU]	Area %
1	37.648	BV	1.0768	3.14661e4	455.45776	51.4309
2	40.004	VB	1.1253	1363.98022	18.43505	2.2294
3	45.003	BB	1.3218	2.72170e4	320.32382	44.4858
4	56.983	BB	1.5630	1134.23645	10.59926	1.8539
Totals :				6.11813e4	804.81590	

Supplementary Figure 30. HPLC spectra for **3f**.

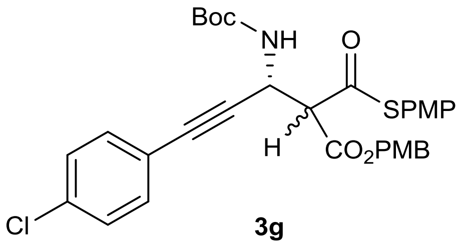

3g

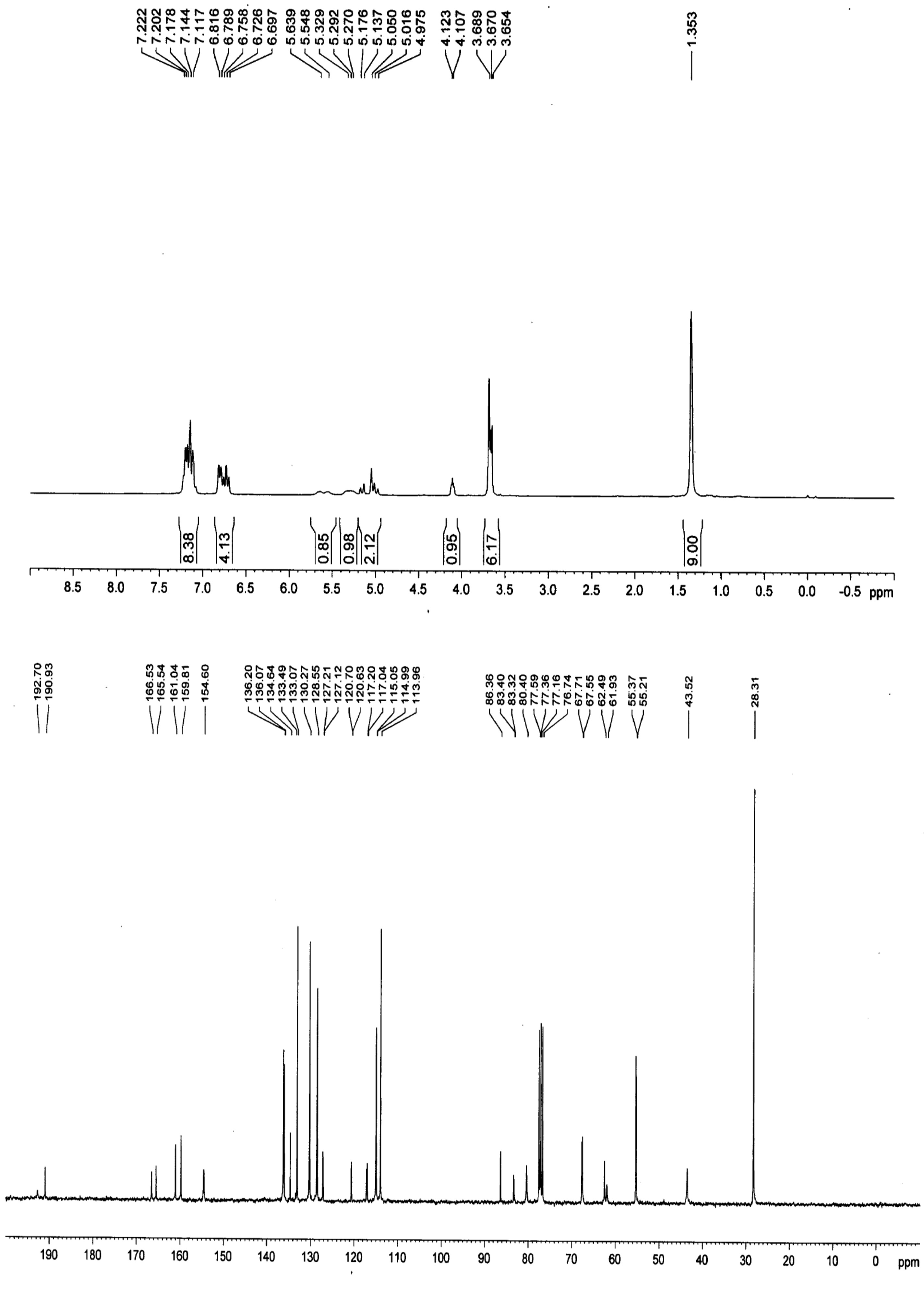

Supplementary Figure 31. ^{1}H and ^{13}C NMR spectra for **3g**.

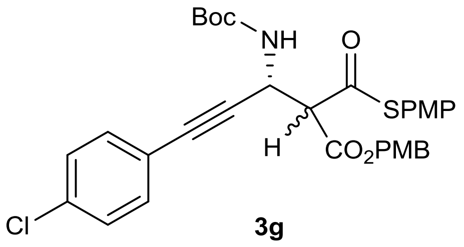

3g

HPLC analysis of **3g**: Daicel CHIRALPAK AD-H, *n*-hexane/*i*-PrOH = 80/20, flow rate = 0.8 mL/min, λ = 254 nm

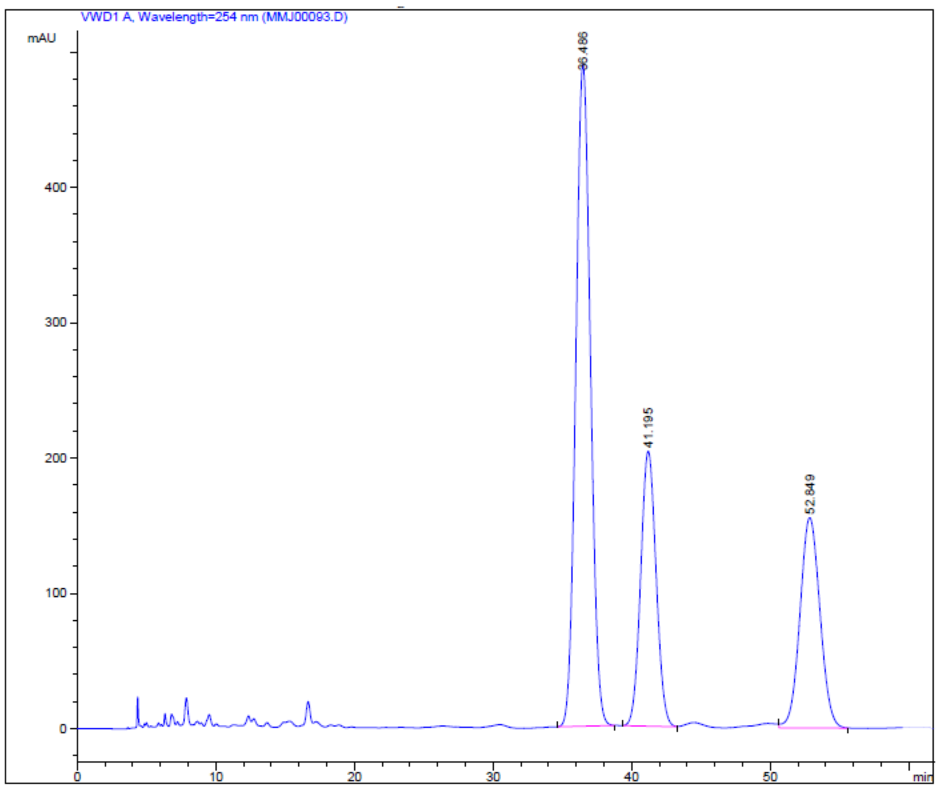

Peak #	RetTime [min]	Type	Width [min]	Area mAU *s	Height [mAU]	Area %
1	36.486	BB	1.1516	3.58225e4	489.03723	53.3478
2	41.195	BB	1.2079	1.57100e4	203.15869	23.3958
3	52.849	VB	1.5702	1.56164e4	155.36324	23.2564
Totals :				6.71489e4	847.55916	

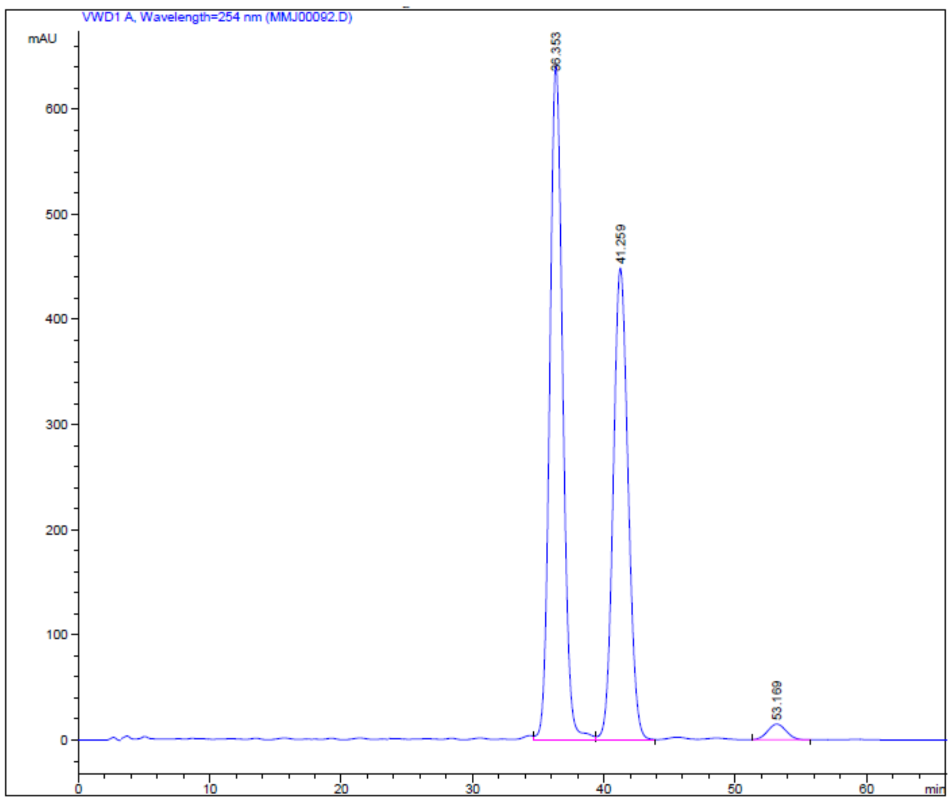

Peak #	RetTime [min]	Type	Width [min]	Area mAU *s	Height [mAU]	Area %
1	36.353	VV	1.0656	4.39963e4	641.11884	54.5371
2	41.259	VB	1.2257	3.52274e4	448.14981	43.6674
3	53.169	BP	1.5120	1448.48657	14.83330	1.7955
Totals :				8.06722e4	1104.10195	

Supplementary Figure 32. HPLC spectra for **3g**.

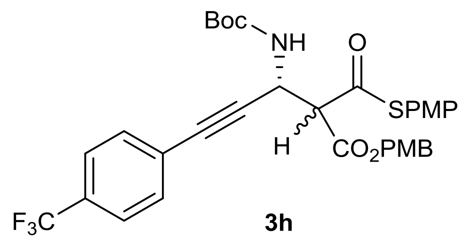

3h

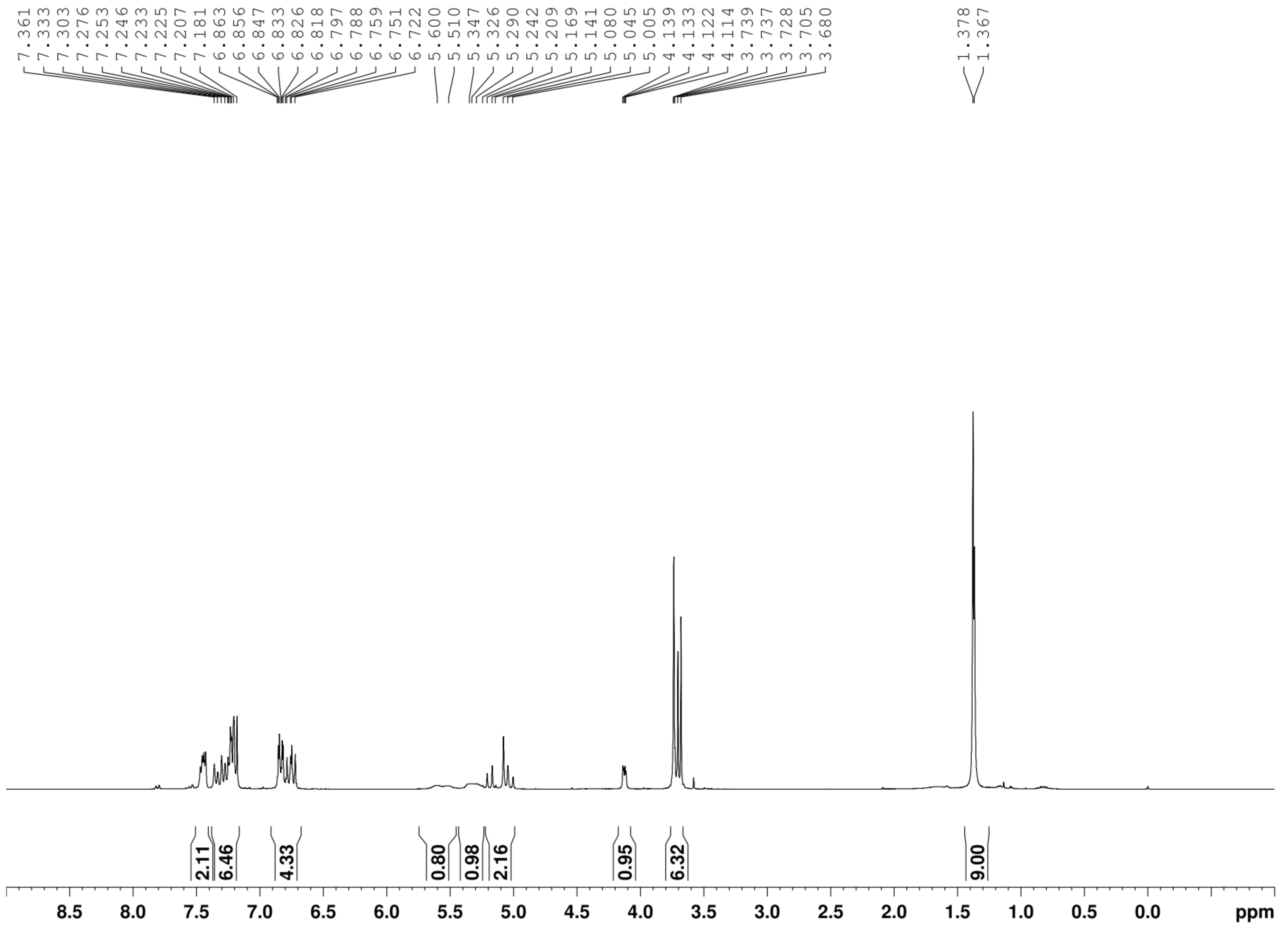

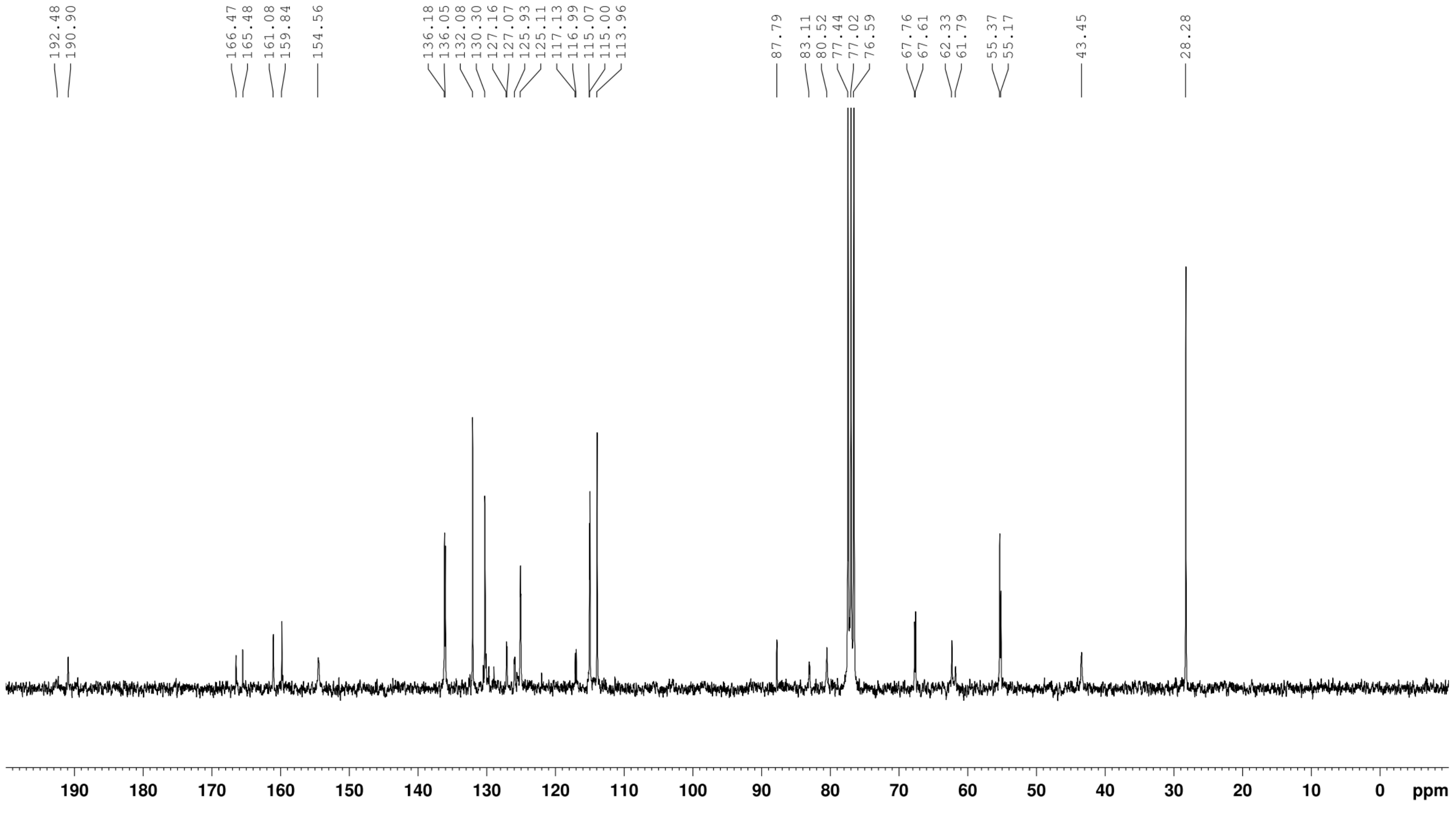

Supplementary Figure 33. ^{1}H and ^{13}C NMR spectra for **3h**.

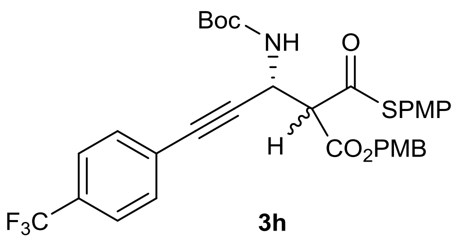

3h

HPLC analysis of **3h**: Daicel CHIRALPAK AD-H, *n*-hexane/*i*-PrOH = 88/12, flow rate = 0.9 mL/min, λ = 254 nm

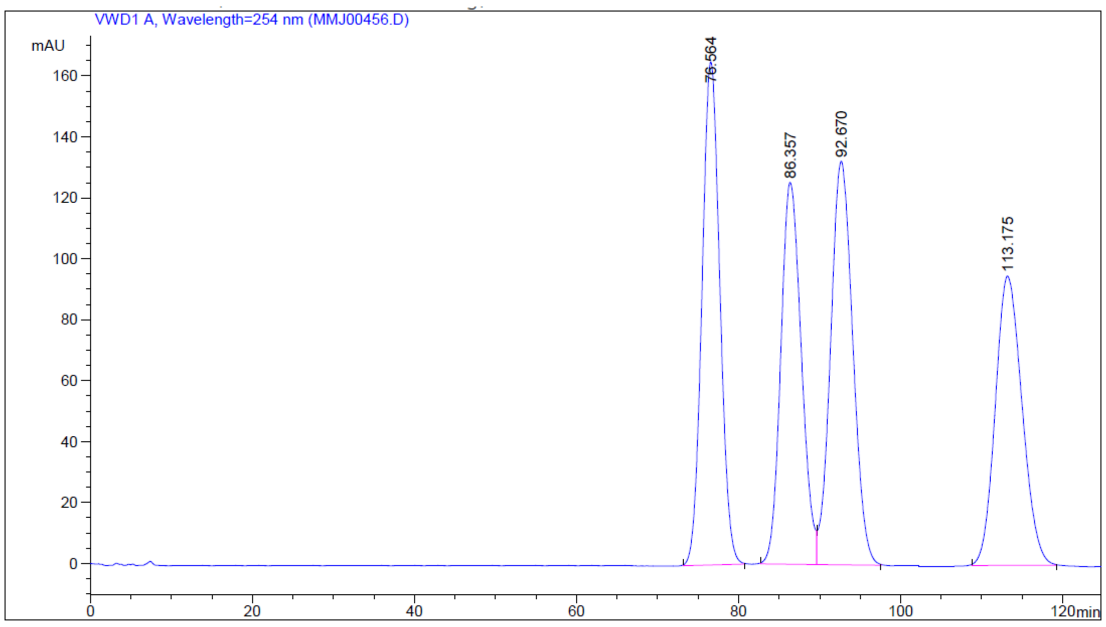

Peak #	RetTime [min]	Type	Width [min]	Area mAU *s	Height [mAU]	Area %
1	76.564	BB	2.3783	2.52498e4	165.10207	26.7430
2	86.357	BV	2.7420	2.20689e4	125.30521	23.3740
3	92.670	VB	2.9416	2.52661e4	132.46732	26.7602
4	113.175	BB	3.2171	2.18318e4	94.99258	23.1228
Totals :				9.44166e4	517.86718	

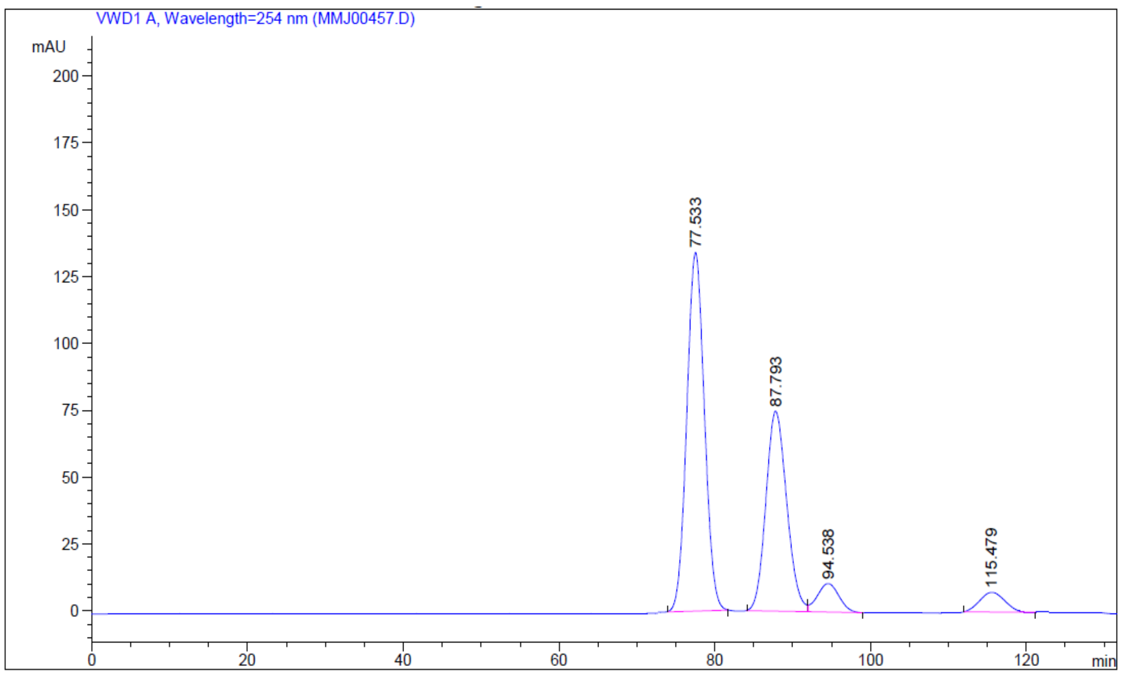

Peak #	RetTime [min]	Type	Width [min]	Area mAU *s	Height [mAU]	Area %
1	77.533	BB	2.4202	2.10790e4	134.13538	54.4558
2	87.793	BV	2.7976	1.39252e4	74.86395	35.9746
3	94.538	VB	2.2761	2077.14941	10.70177	5.3661
4	115.479	BP	2.6260	1627.11426	7.34291	4.2035
Totals :				3.87085e4	227.04401	

Supplementary Figure 34. HPLC spectra for **3h**.

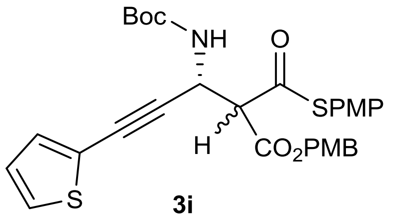

3i

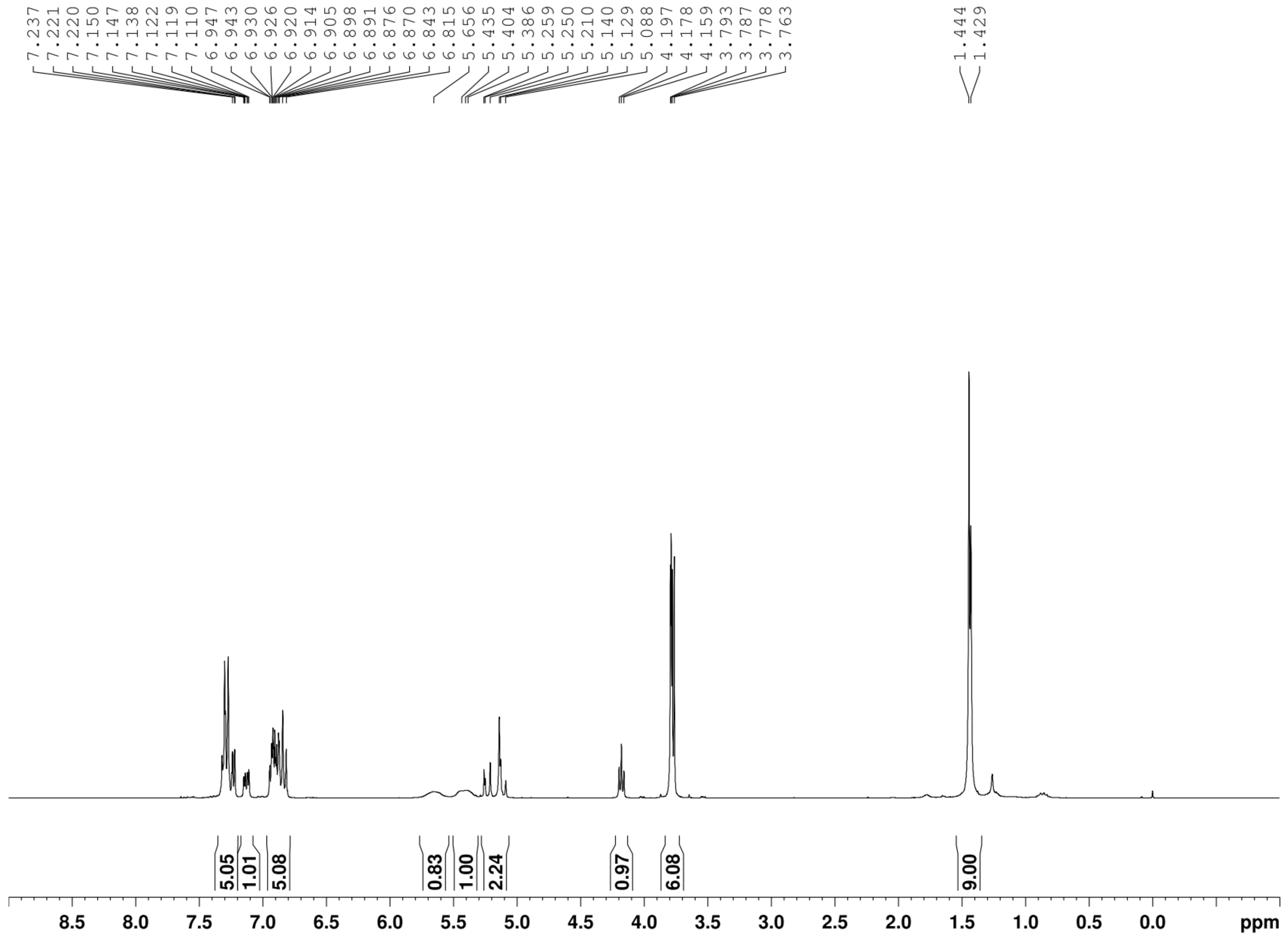

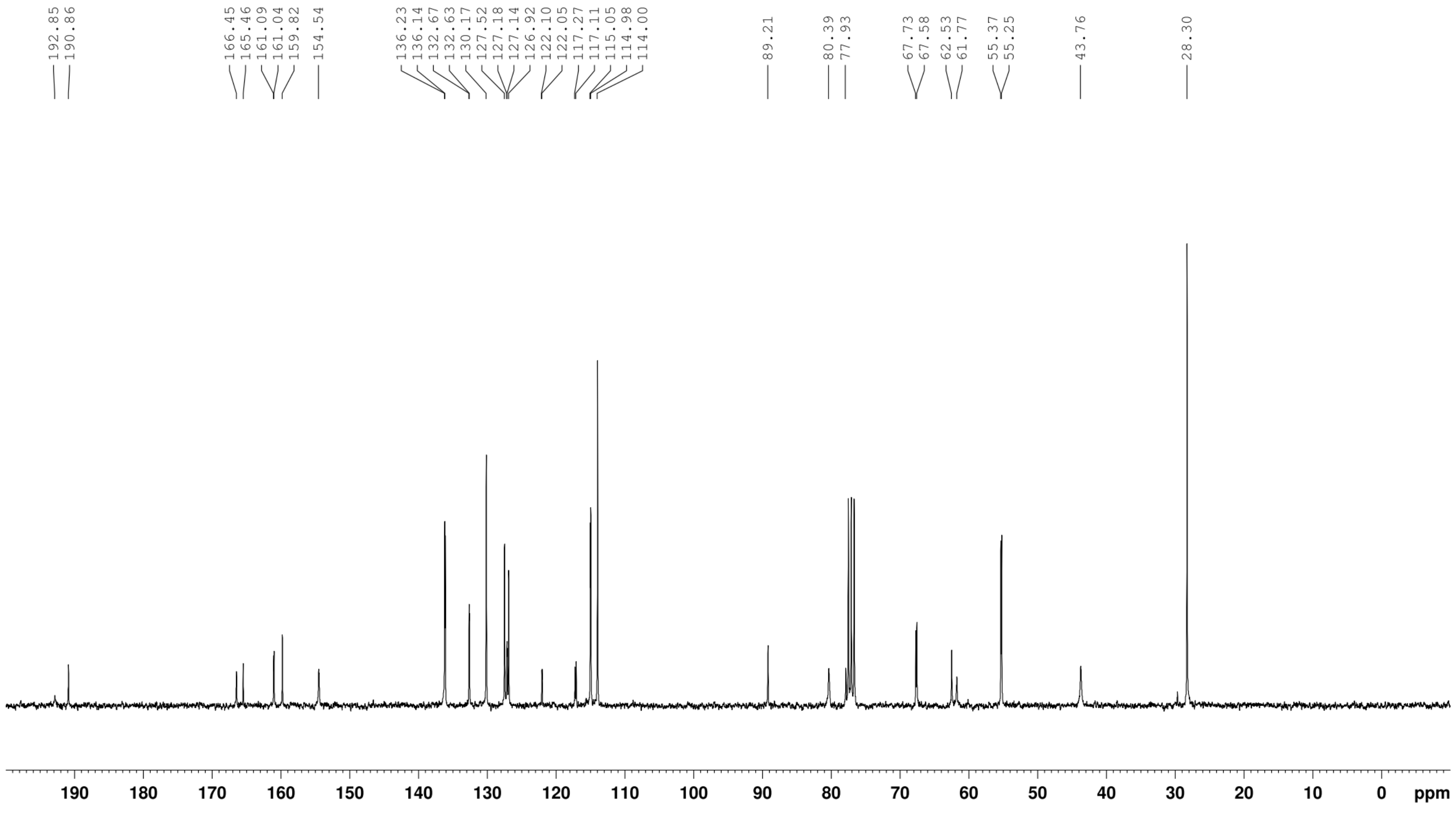

Supplementary Figure 35. ^{1}H and ^{13}C NMR spectra for **3i**.

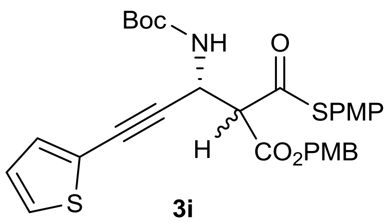

3i

HPLC analysis of **3i**: Daicel CHIRALPAK AD-H, *n*-hexane/*i*-PrOH = 80/20, flow rate = 0.8 mL/min, λ = 254 nm

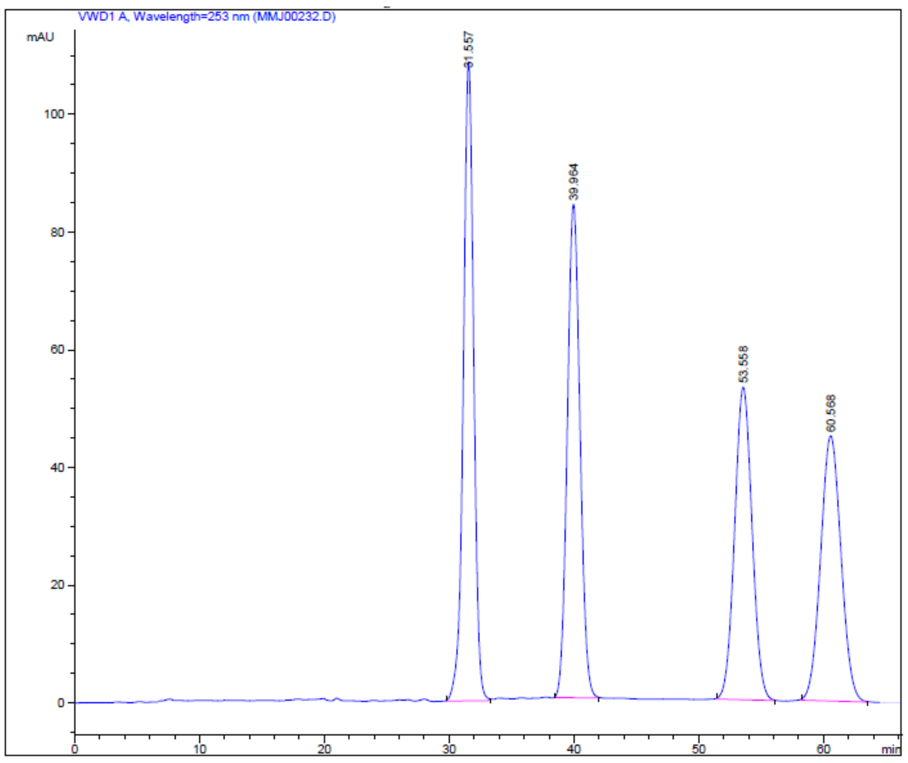

Peak #	RetTime [min]	Type	Width [min]	Area mAU *s	Height [mAU]	Area %
1	31.557	BB	0.8873	6248.35938	108.50401	27.8602
2	39.964	BB	1.1001	5918.04102	83.84307	26.3873
3	53.558	BB	1.5074	5151.42432	53.10250	22.9691
4	60.568	BB	1.7543	5109.75537	45.05020	22.7834
Totals :				2.24276e4	290.49978	

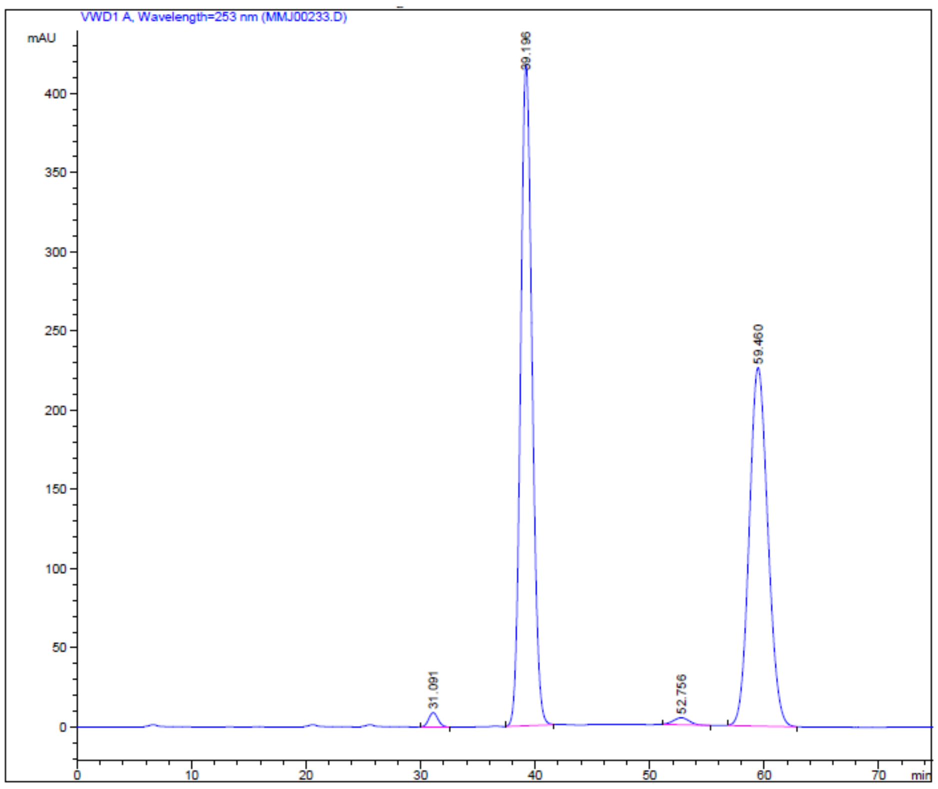

Peak #	RetTime [min]	Type	Width [min]	Area mAU *s	Height [mAU]	Area %
1	31.091	BB	0.8751	507.65750	9.01827	0.9100
2	39.196	PB	1.0914	2.92713e4	417.63440	52.4691
3	52.756	BP	1.4917	420.55402	4.41934	0.7538
4	59.460	BB	1.7561	2.55882e4	226.27953	45.8671
Totals :				5.57878e4	657.35154	

Supplementary Figure 36. HPLC spectra for **3i**.

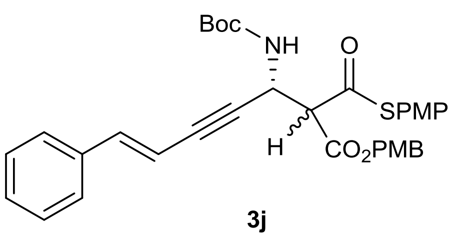

3j

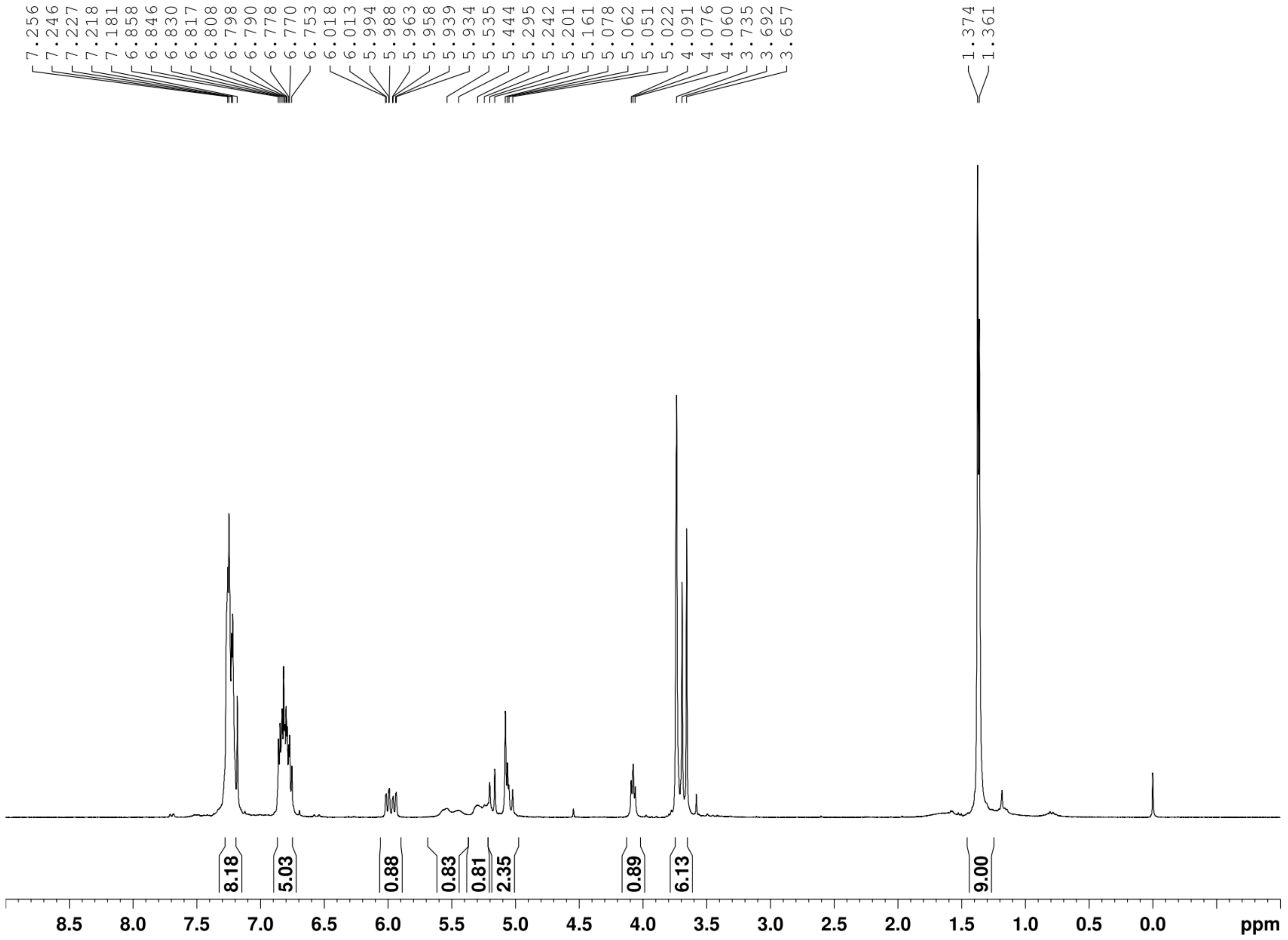

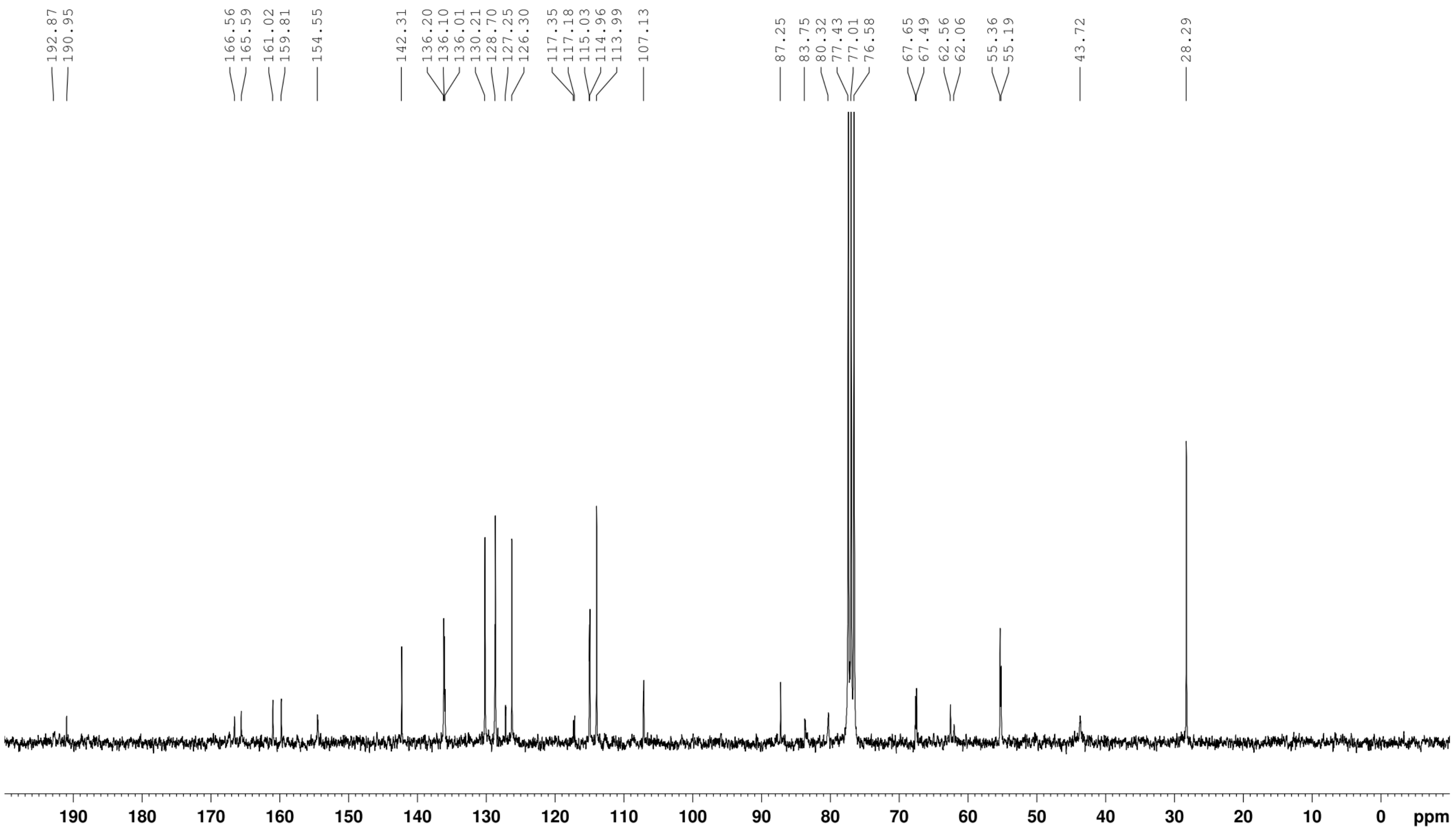

Supplementary Figure 37. ^{1}H and ^{13}C NMR spectra for **3j**.

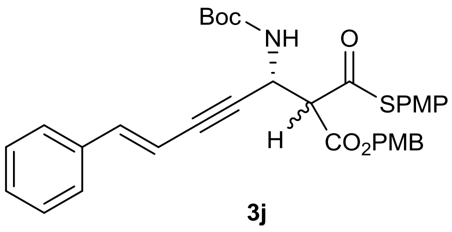

3j

HPLC analysis of **3j**: Daicel CHIRALPAK AD-H, *n*-hexane/*i*-PrOH = 80/20, flow rate = 0.8 mL/min, λ = 254 nm

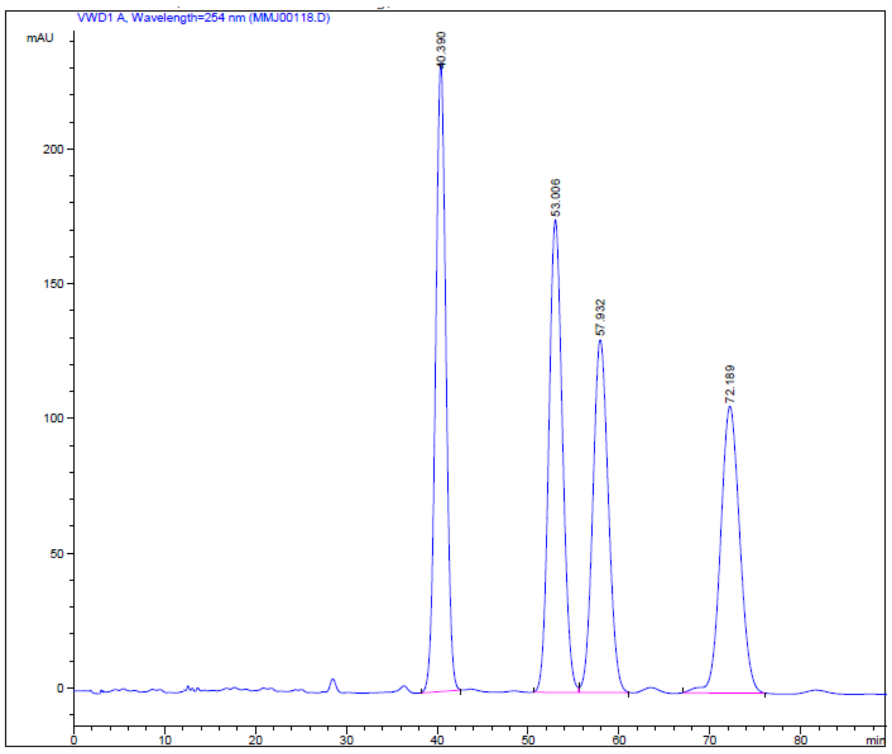

Peak #	RetTime [min]	Type	Width [min]	Area mAU *s	Height [mAU]	Area %
1	40.390	BB	1.2273	1.84624e4	233.35611	27.2244
2	53.006	BV	1.5993	1.82822e4	175.35564	26.9585
3	57.932	VB	1.8296	1.54464e4	130.89331	22.7769
4	72.189	BB	2.2648	1.56249e4	106.41801	23.0402
Totals :				6.78158e4	646.02307	

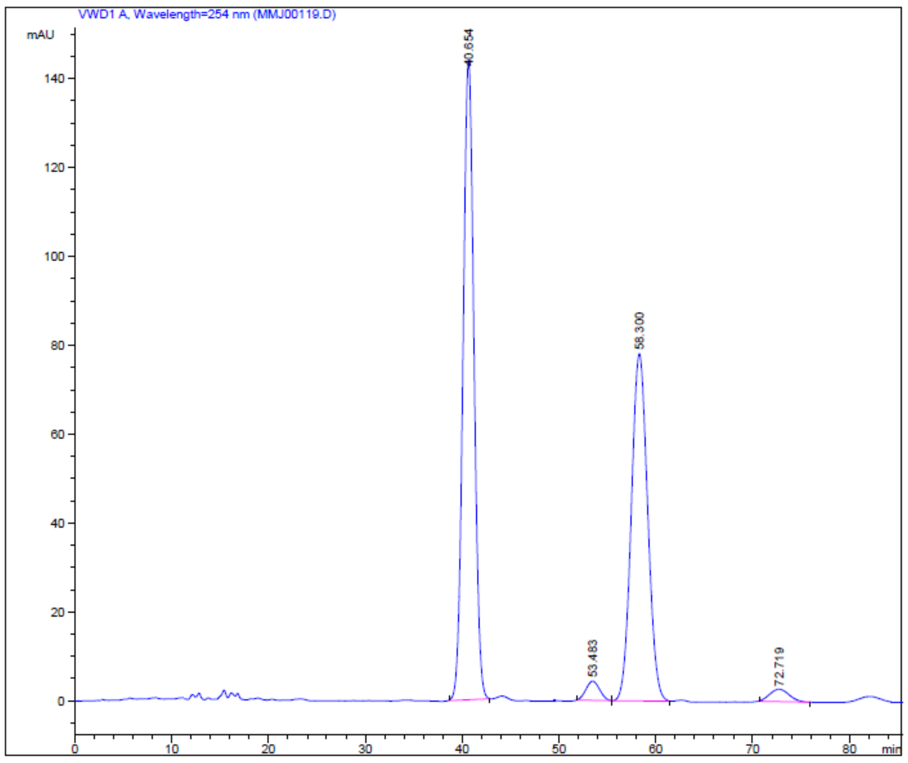

Peak #	RetTime [min]	Type	Width [min]	Area mAU *s	Height [mAU]	Area %
1	40.654	BB	1.2182	1.12601e4	143.97365	53.0699
2	53.483	BP	1.2917	424.18588	4.34013	1.9992
3	58.300	VB	1.8123	9146.12500	78.15310	43.1064
4	72.719	BB	1.6387	387.12222	2.79338	1.8245
Totals :				2.12176e4	229.26026	

Supplementary Figure 38. HPLC spectra for **3j**.

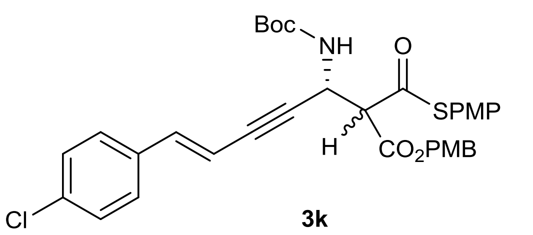

3k

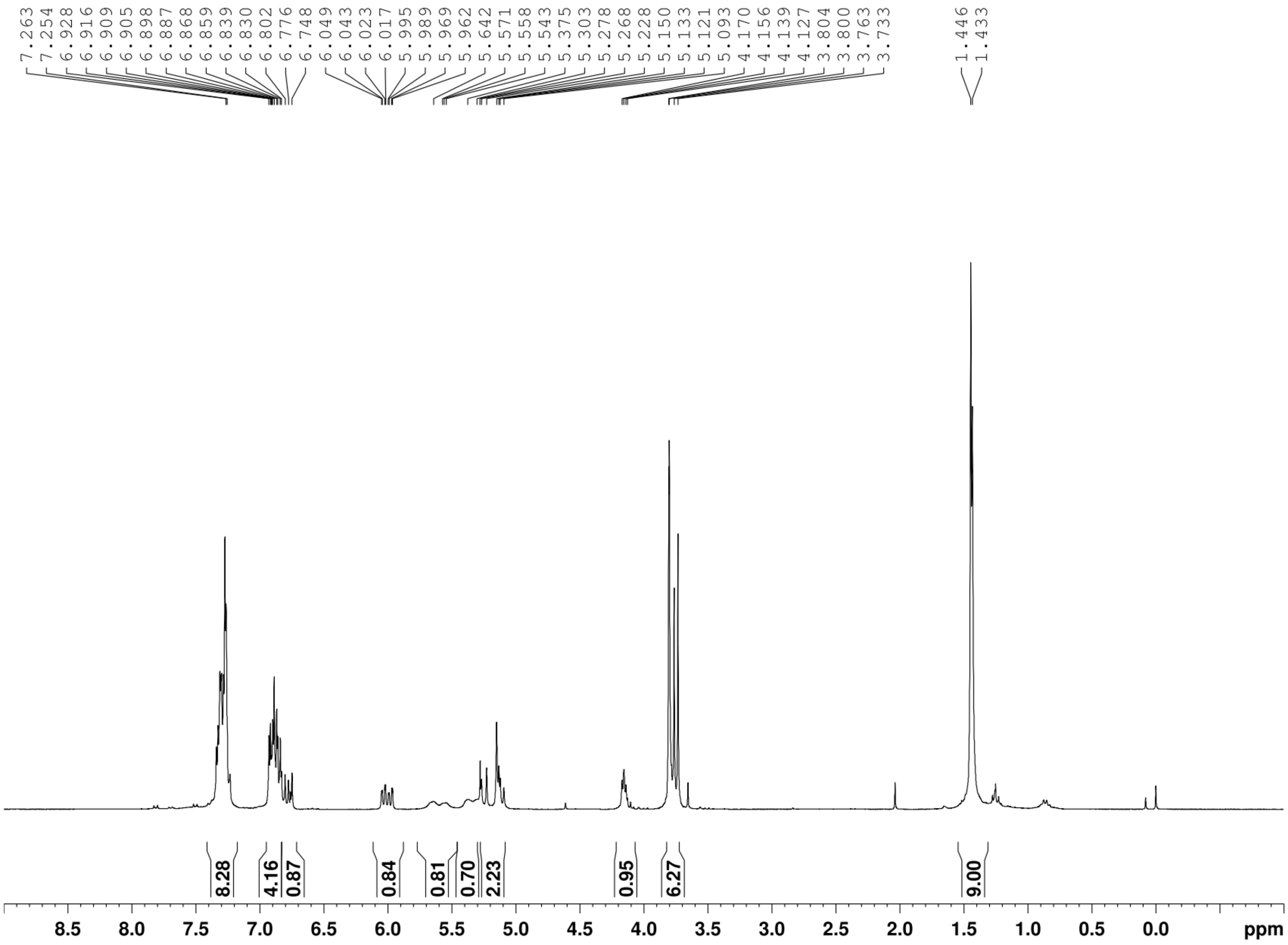

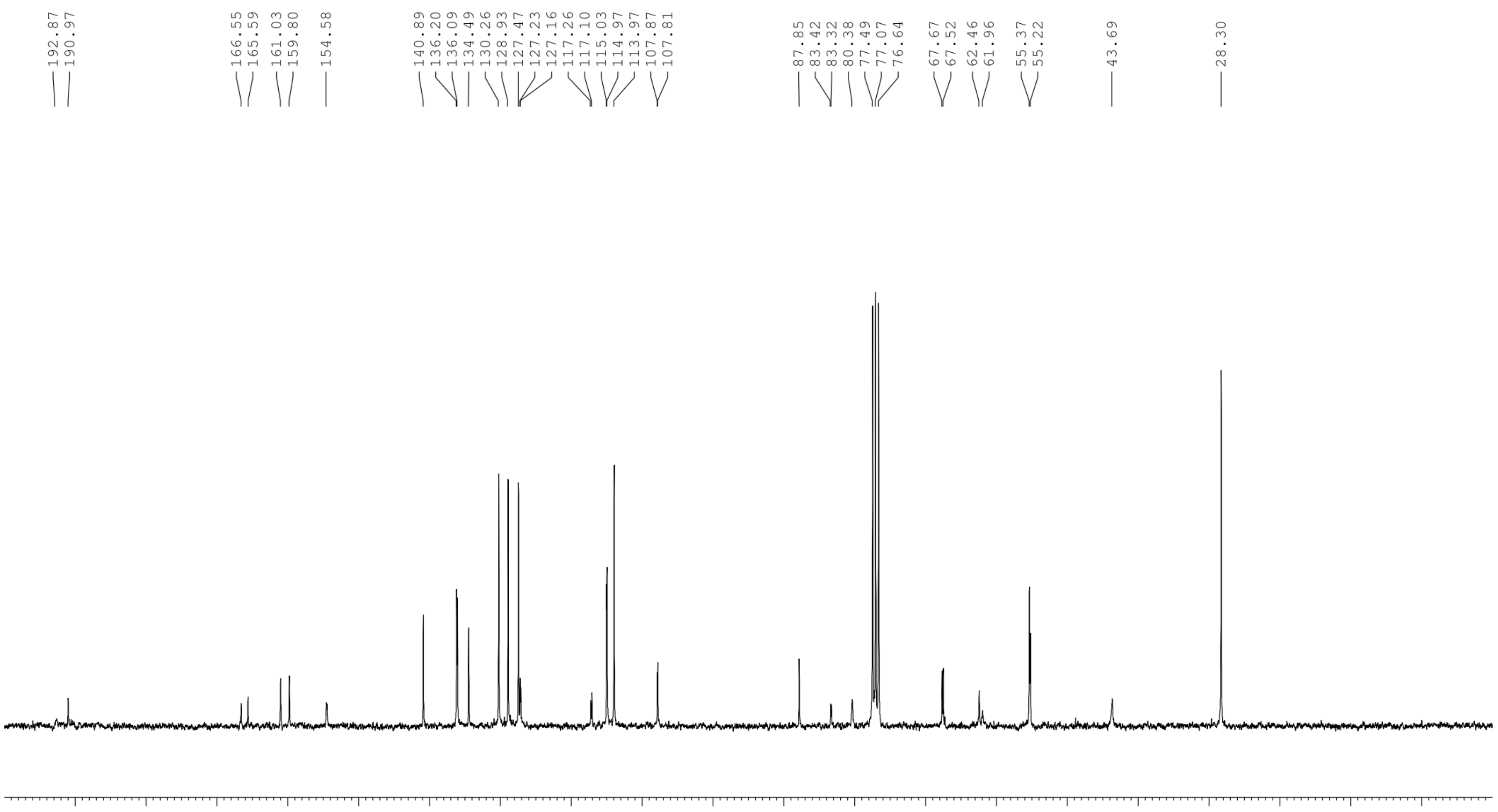

Supplementary Figure 39. ^{1}H and ^{13}C NMR spectra for **3k**.

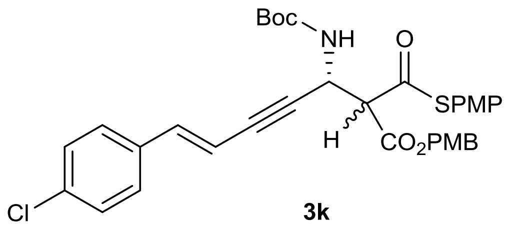

3k

HPLC analysis of **3k**: Daicel CHIRALPAK AD-H, *n*-hexane/*i*-PrOH = 80/20, flow rate = 0.8 mL/min, λ = 254 nm

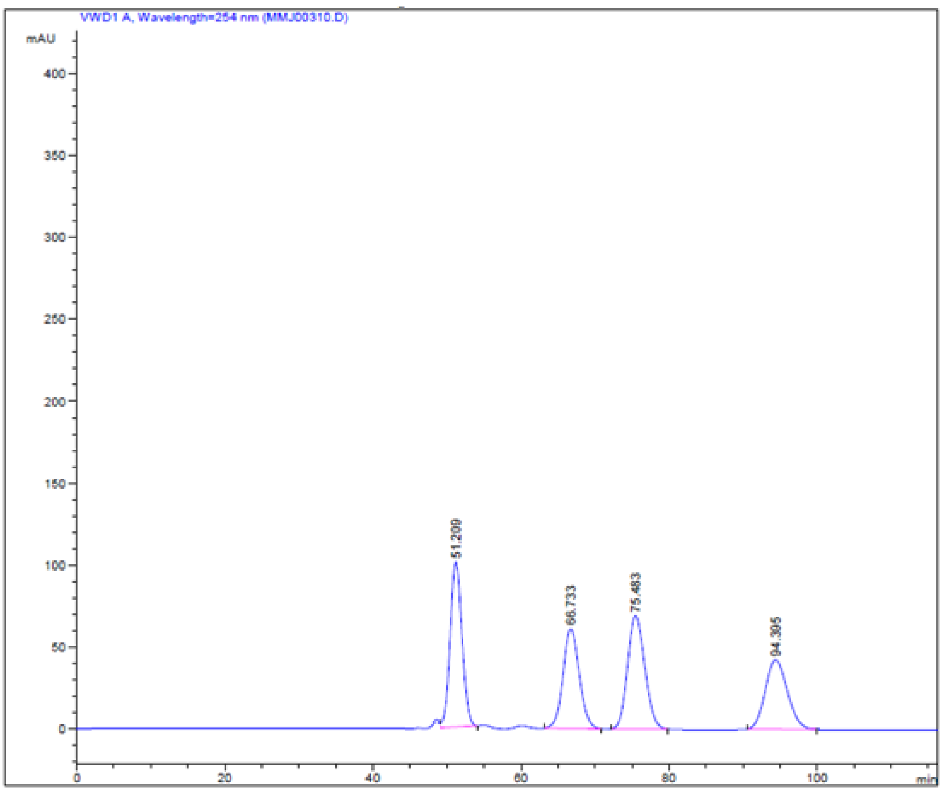

Peak #	RetTime [min]	Type	Width [min]	Area mAU *s	Height [mAU]	Area %
1	51.209	VP	1.7091	1.11451e4	100.47322	27.5212
2	66.733	BB	2.2430	9297.96875	60.74215	22.9600
3	75.483	BB	2.3756	1.12529e4	69.16700	27.7875
4	94.395	BB	2.8518	8800.34082	42.33537	21.7312
Totals :				4.04963e4	272.71775	

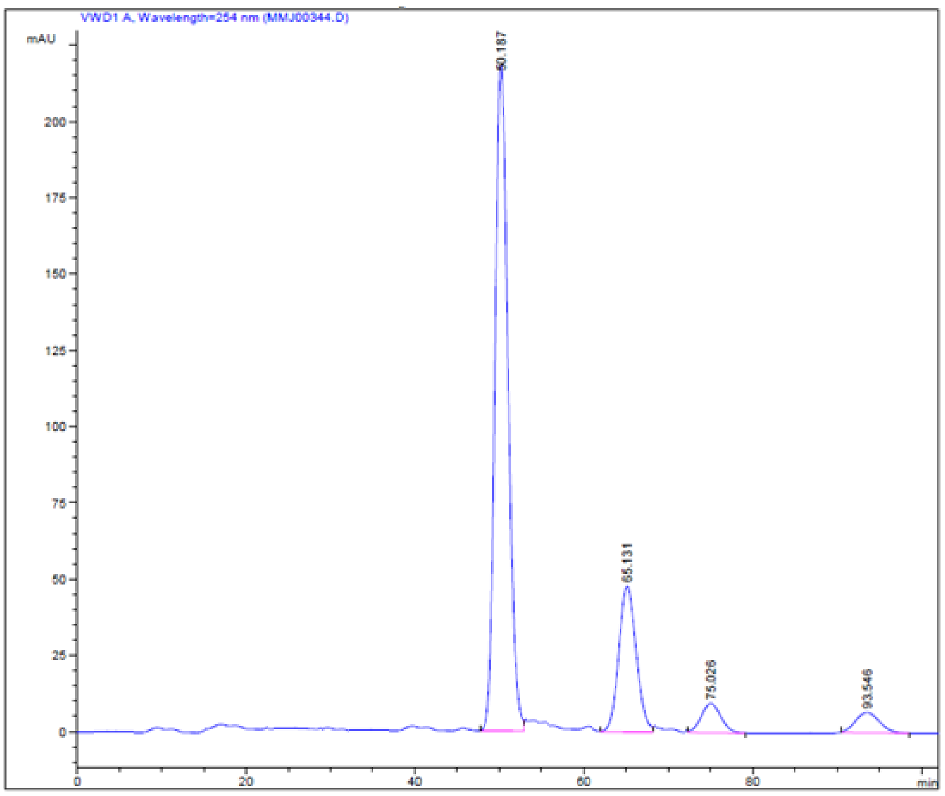

Peak #	RetTime [min]	Type	Width [min]	Area mAU *s	Height [mAU]	Area %
1	50.187	BV	1.6736	2.36486e4	218.45470	70.7513
2	65.131	VV	2.1979	6901.79004	47.94238	20.6486
3	75.026	VB	2.2447	1558.02979	9.63515	4.6613
4	93.546	BB	2.3717	1316.57190	6.67294	3.9389
Totals :				3.34250e4	282.70515	

Supplementary Figure 40. HPLC spectra for **3k**.

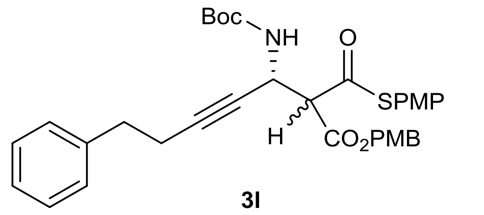

3l

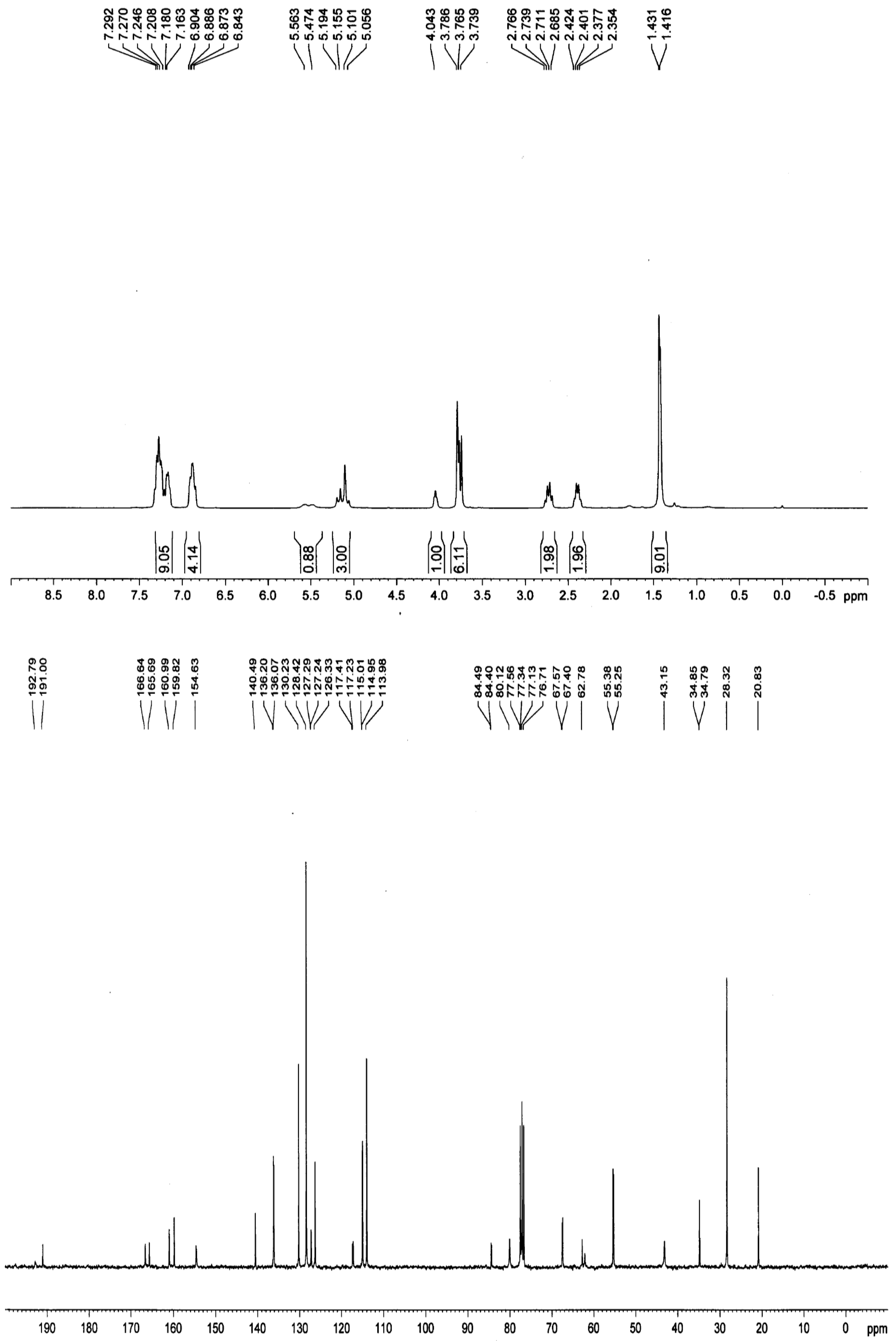

Supplementary Figure 41. ^{1}H and ^{13}C NMR spectra for **3l**.

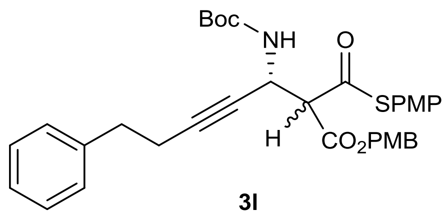

3l

HPLC analysis of **3l**: Daicel CHIRALPAK AD-H, *n*-hexane/*i*-PrOH = 80/20, flow rate = 0.8 mL/min, λ = 254 nm

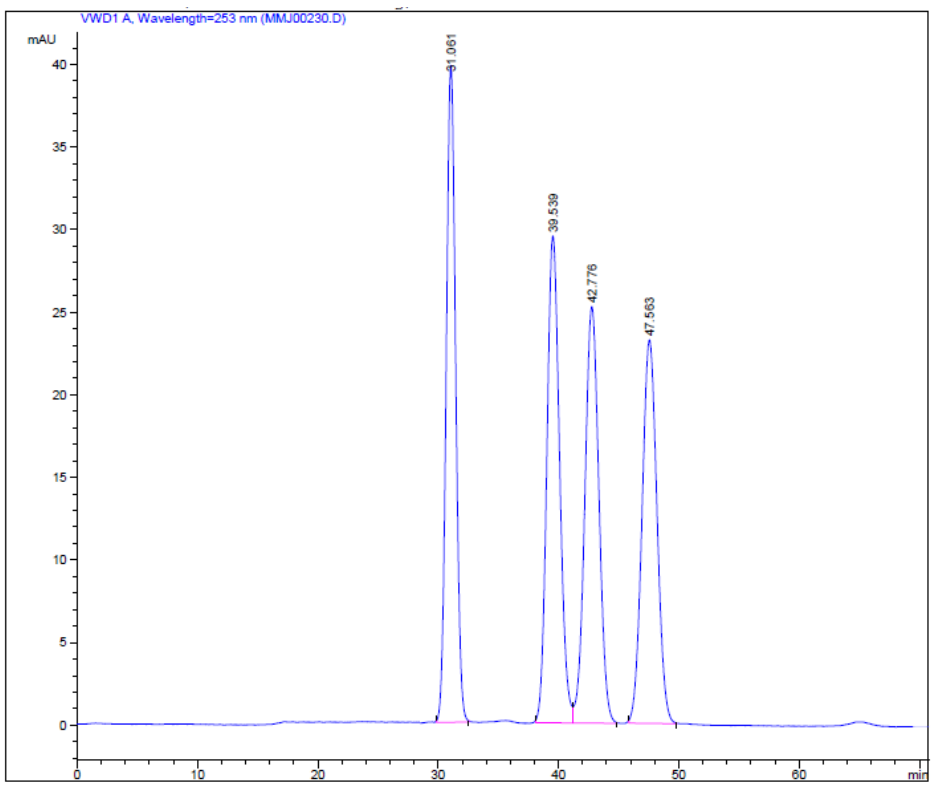

Peak #	RetTime [min]	Type	Width [min]	Area mAU *s	Height [mAU]	Area %
1	31.061	BB	0.8413	2152.54126	39.75747	25.7381
2	39.539	BV	1.1387	2163.75757	29.48341	25.8722
3	42.776	VB	1.2560	2047.55933	25.21530	24.4828
4	47.563	BB	1.3398	1999.39514	23.21319	23.9069
Totals :				8363.25330	117.66937	

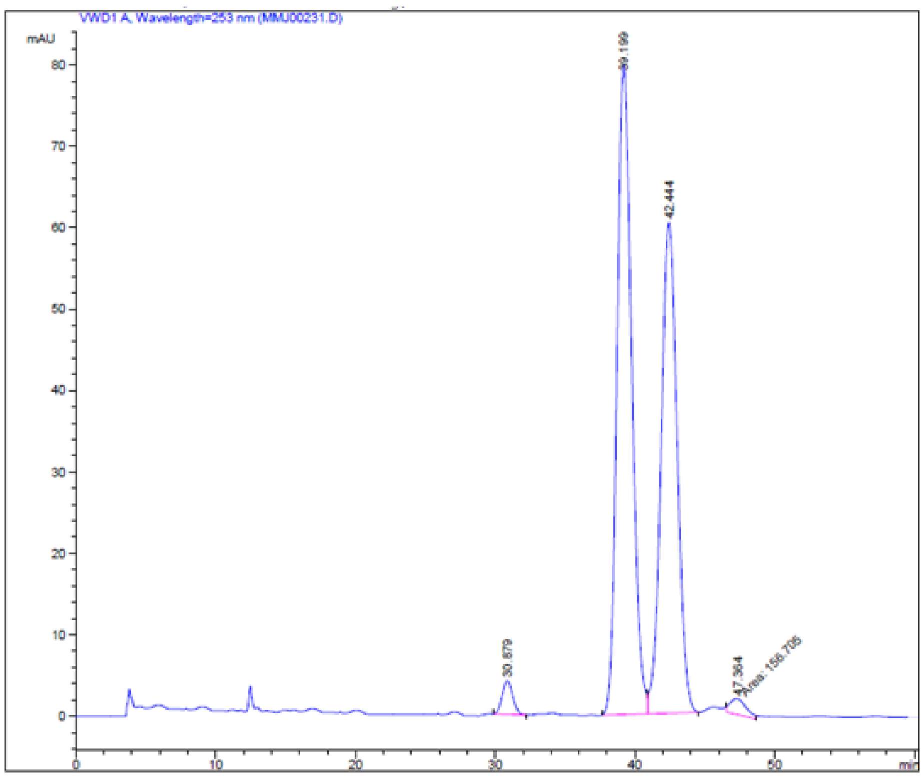

Peak #	RetTime [min]	Type	Width [min]	Area mAU *s	Height [mAU]	Area %
1	30.879	BP	0.7819	216.61737	4.12269	1.9605
2	39.199	BV	1.1336	5828.31055	79.89040	52.7497
3	42.444	VP	1.2381	4847.35791	60.27806	43.8715
4	47.364	MM	1.3328	156.70535	1.95954	1.4183
Totals :				1.10490e4	146.25069	

Supplementary Figure 42. HPLC spectra for **3l**.

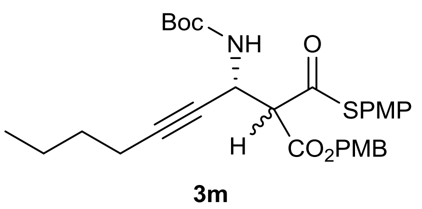

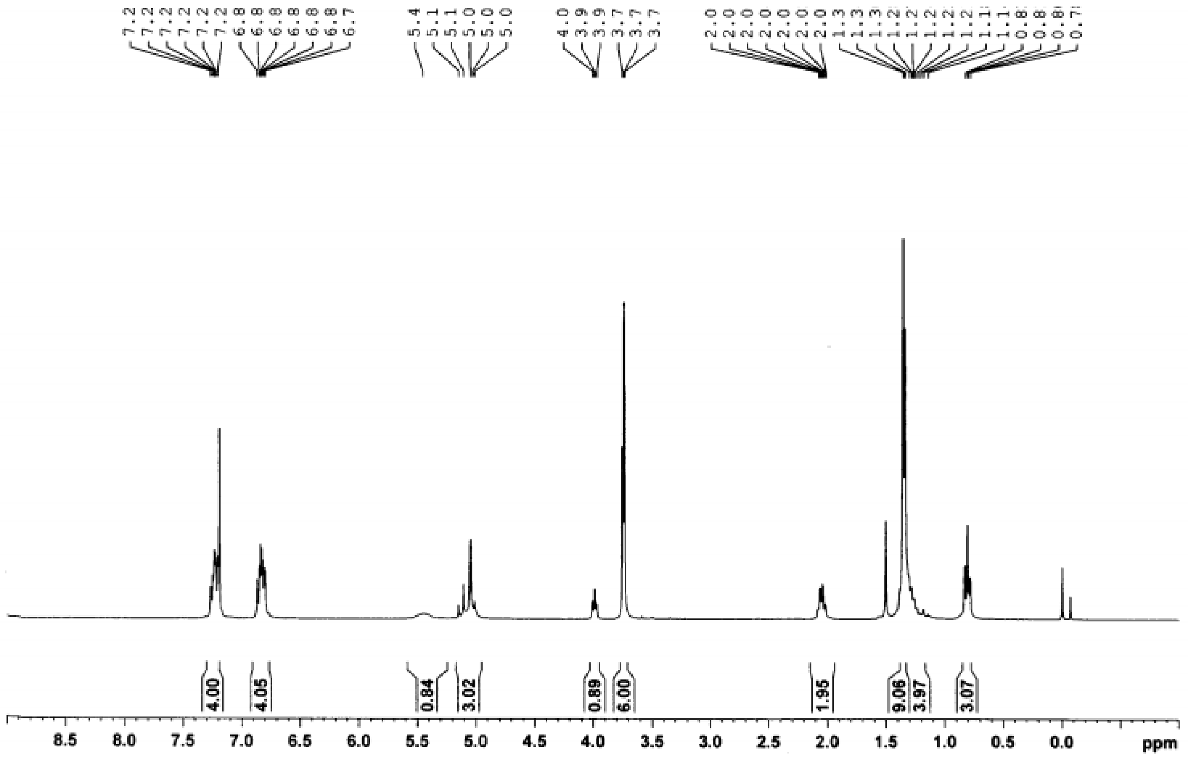

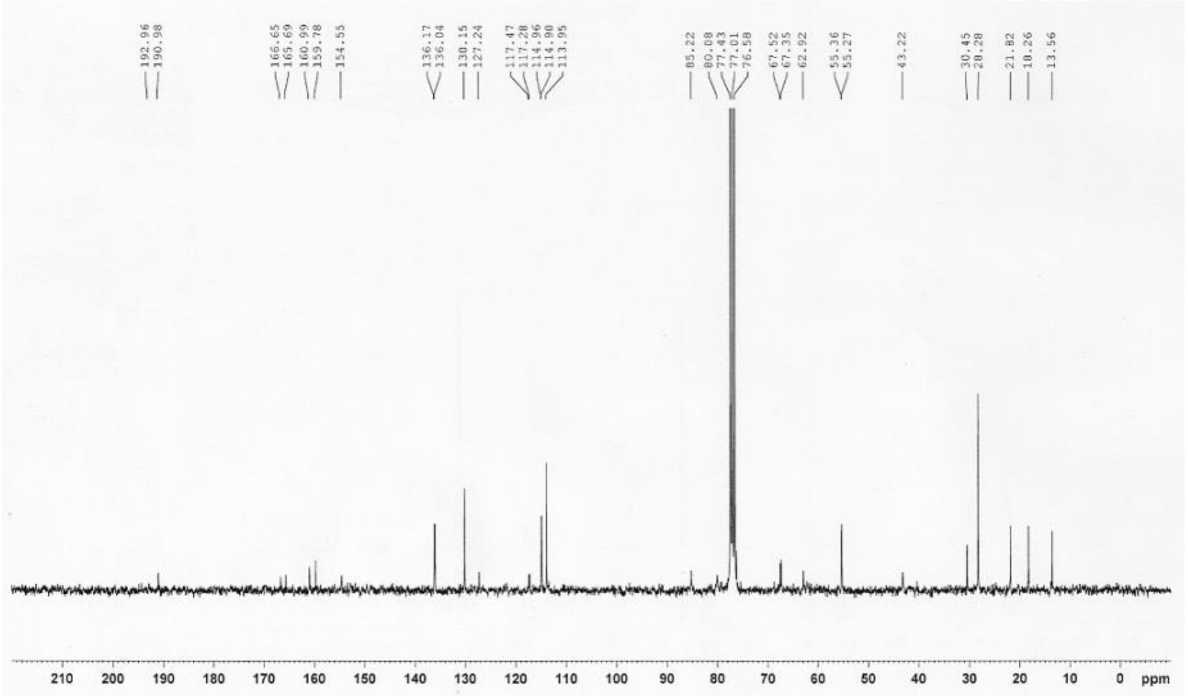

Supplementary Figure 43. ^{1}H and ^{13}C NMR spectra for **3m**.

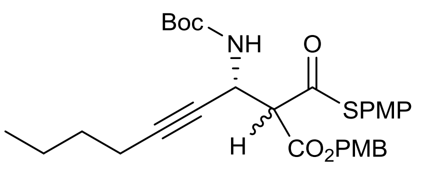

3m

HPLC analysis of **3m**: Daicel CHIRALPAK AD-H, *n*-hexane/*i*-PrOH = 85/15, flow rate = 0.9 mL/min, λ = 254 nm

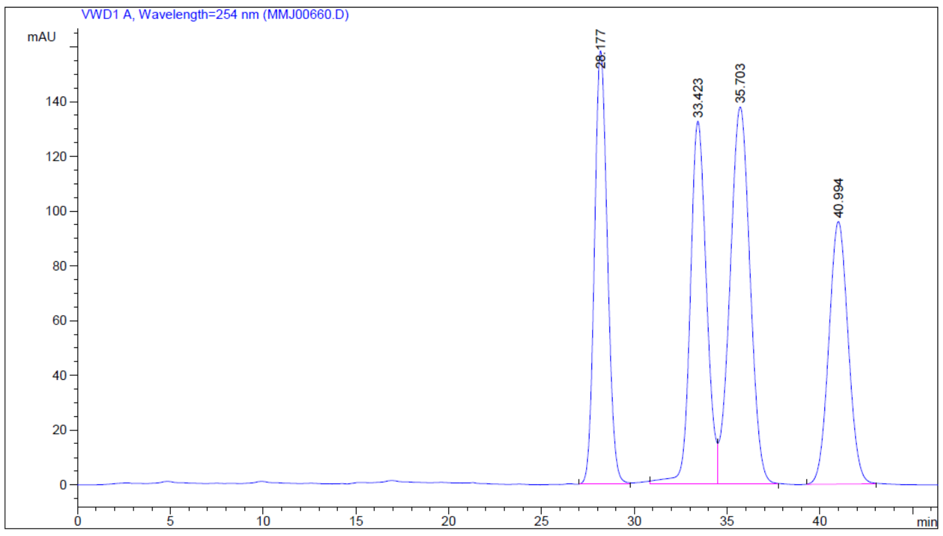

Peak #	RetTime [min]	Type	Width [min]	Area mAU *s	Height [mAU]	Area %
1	28.177	PB	0.7539	7660.54883	158.28680	23.2402
2	33.423	BV	0.9320	8038.63818	132.50882	24.3873
3	35.703	VB	1.1539	1.03219e4	137.73483	31.3142
4	40.994	BB	1.1353	6941.34619	95.93568	21.0583
Totals :				3.29625e4	524.46613	

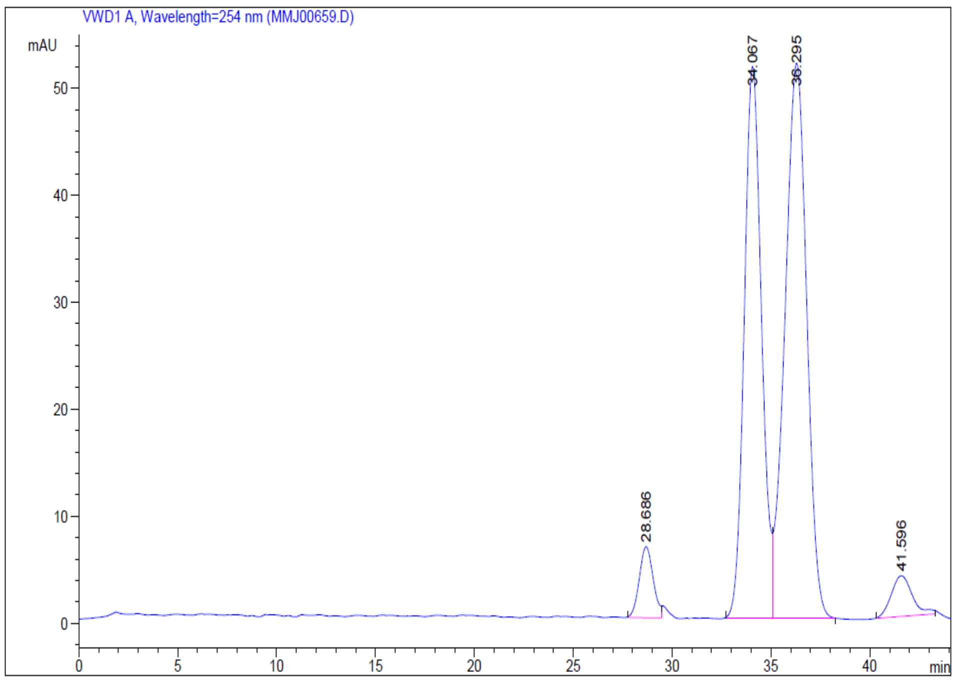

Peak #	RetTime [min]	Type	Width [min]	Area mAU *s	Height [mAU]	Area %
1	28.686	BV	0.7563	322.97583	6.62786	4.1919
2	34.067	BV	0.9274	3122.65967	51.59917	40.5288
3	36.295	VB	1.1851	3976.78931	51.90412	51.6145
4	41.596	BB	1.0400	282.36115	3.80081	3.6648
Totals :				7704.78595	113.93196	

Supplementary Figure 44. HPLC spectra for **3m**.

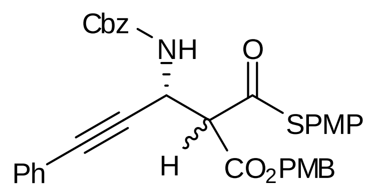

3n

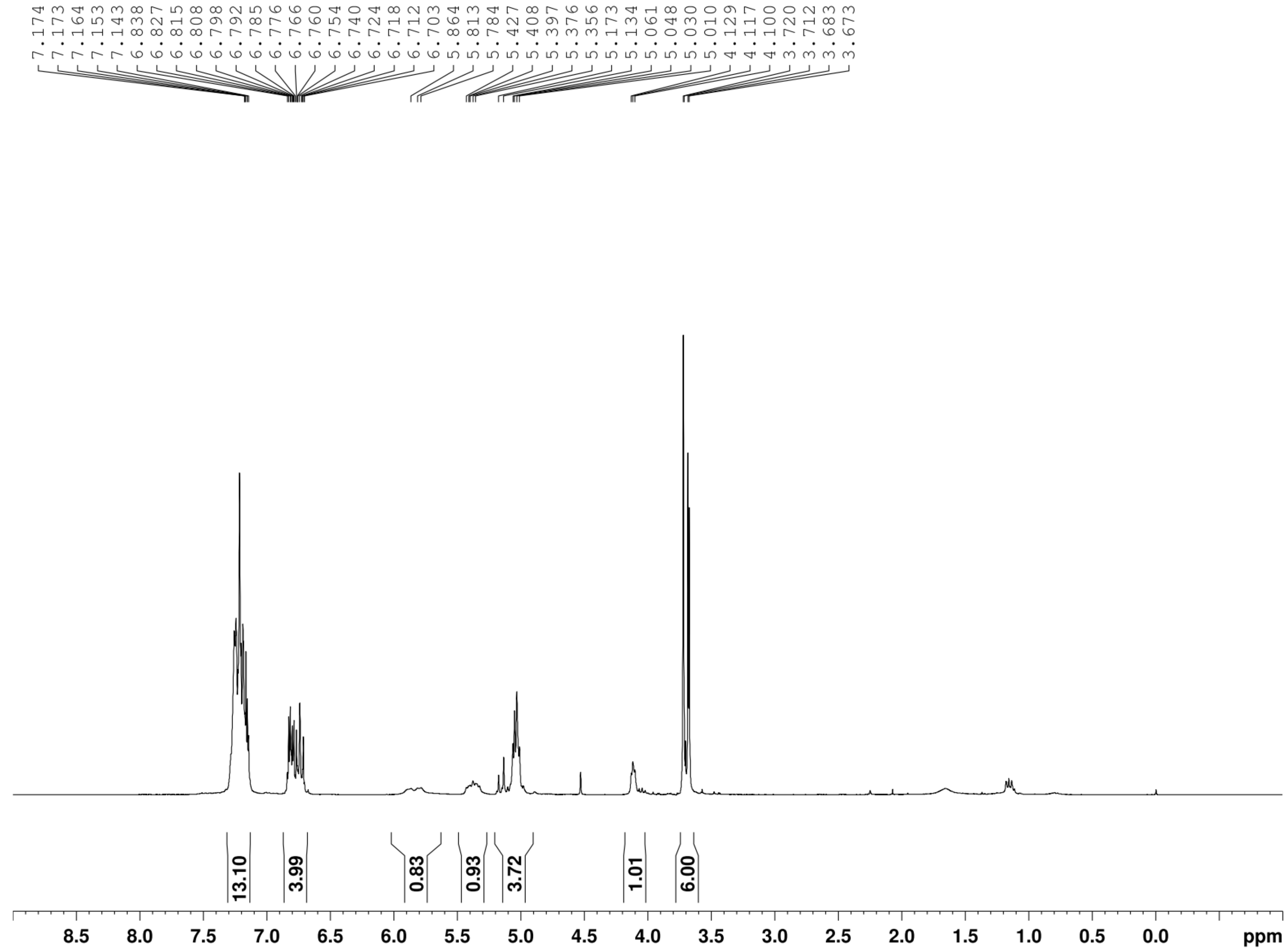

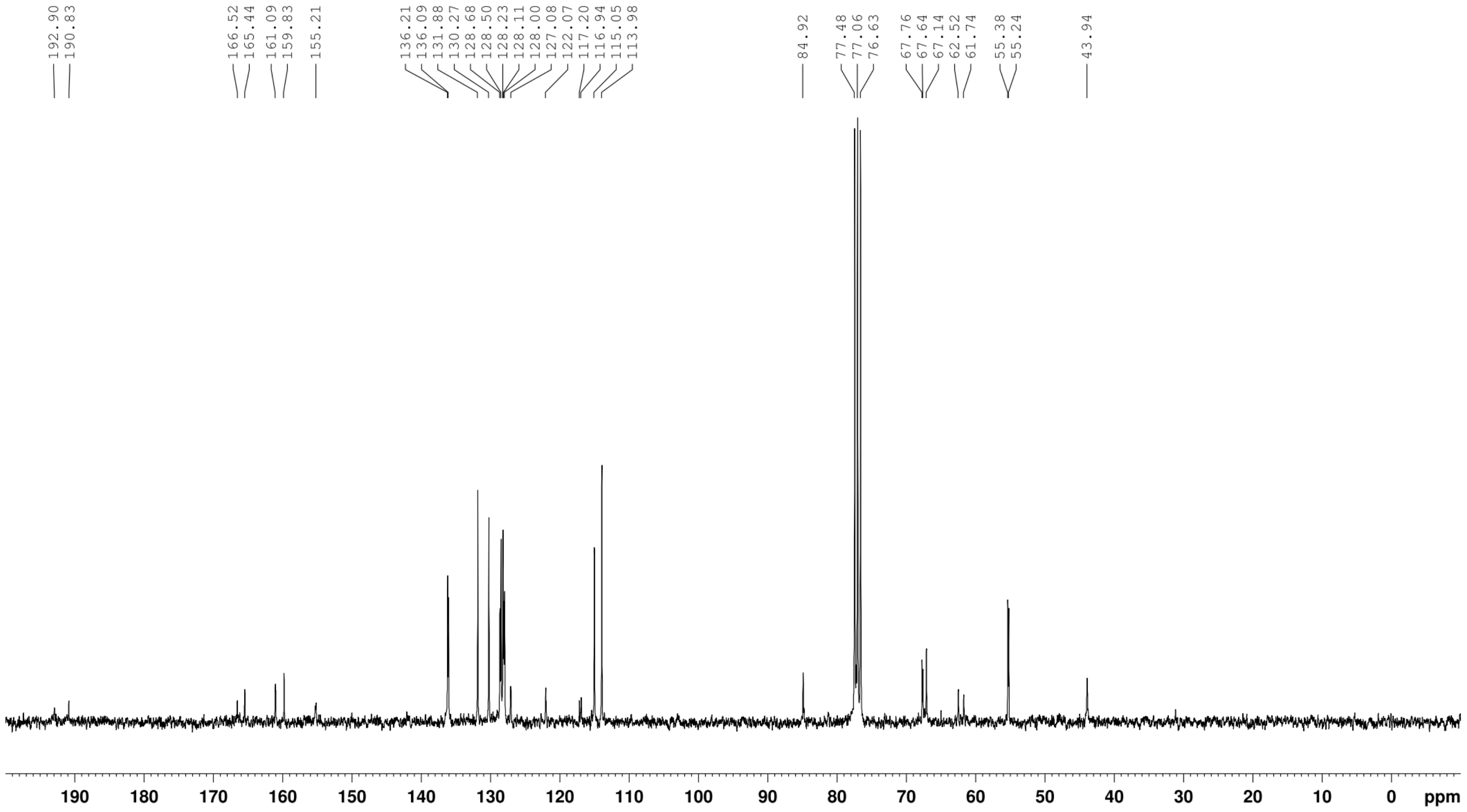

Supplementary Figure 45. ^{1}H and ^{13}C NMR spectra for **3n**.

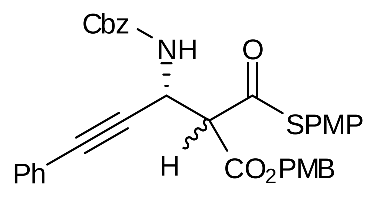

3n

HPLC analysis of **3n**: Daicel CHIRALPAK AD-H, *n*-hexane/*i*-PrOH = 80/20, flow rate = 0.8 mL/min, λ = 254 nm

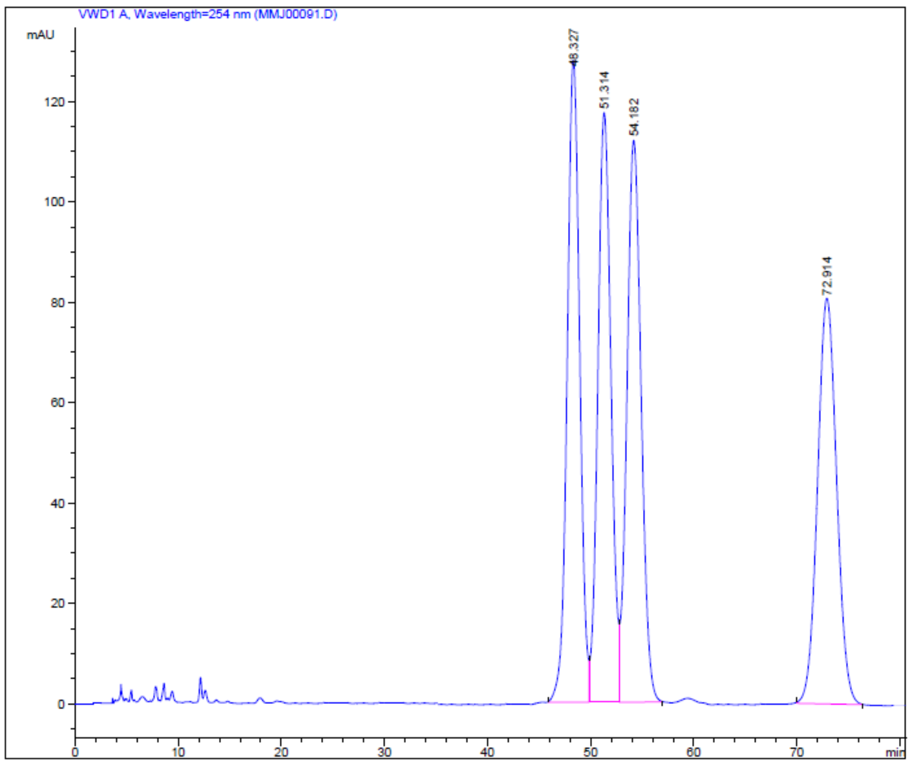

Peak #	RetTime [min]	Type	Width [min]	Area mAU *s	Height [mAU]	Area %
1	48.327	BV	1.3063	1.07773e4	127.89886	25.4766
2	51.314	VV	1.3774	1.04053e4	117.42568	24.5972
3	54.182	VB	1.4803	1.07333e4	111.88306	25.3725
4	72.914	BB	2.0062	1.03869e4	80.81549	24.5536
Totals :				4.23029e4	438.02309	

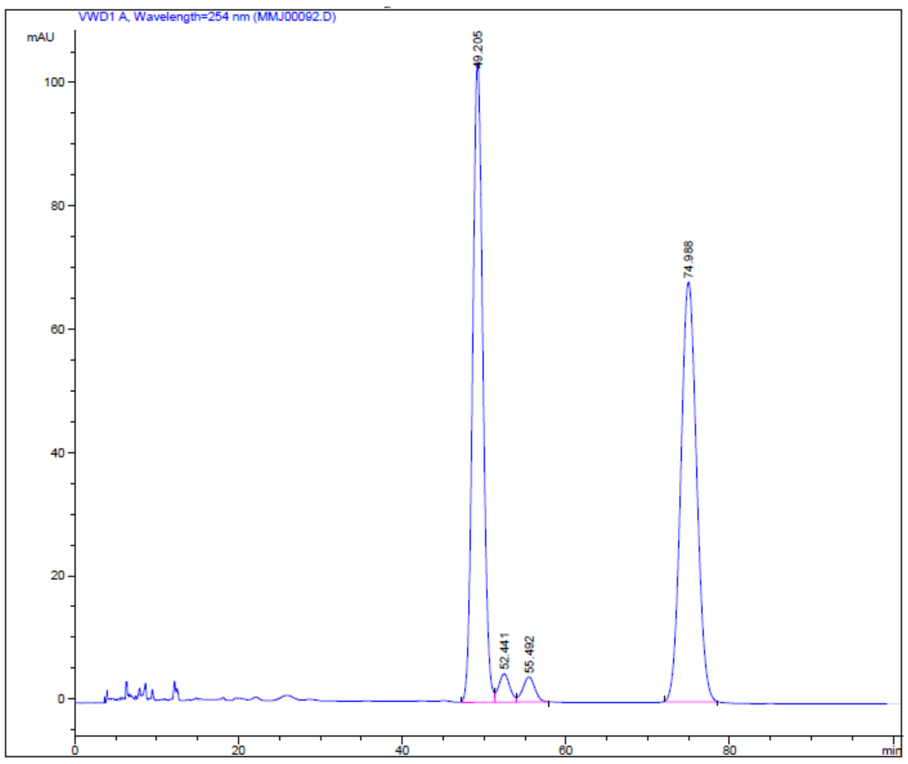

Peak #	RetTime [min]	Type	Width [min]	Area mAU *s	Height [mAU]	Area %
1	49.205	BV	1.3431	8972.11133	103.81974	47.2758
2	52.441	VV	1.1662	431.04239	4.59931	2.2712
3	55.492	VB	1.2810	417.89203	4.06471	2.2020
4	74.988	BB	2.0510	9157.17676	68.15263	48.2510
Totals :				1.89782e4	180.63639	

Supplementary Figure 46. HPLC spectra for **3n**.

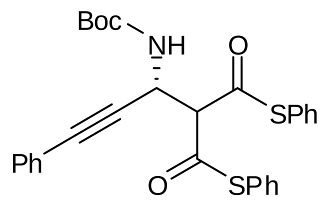

3o

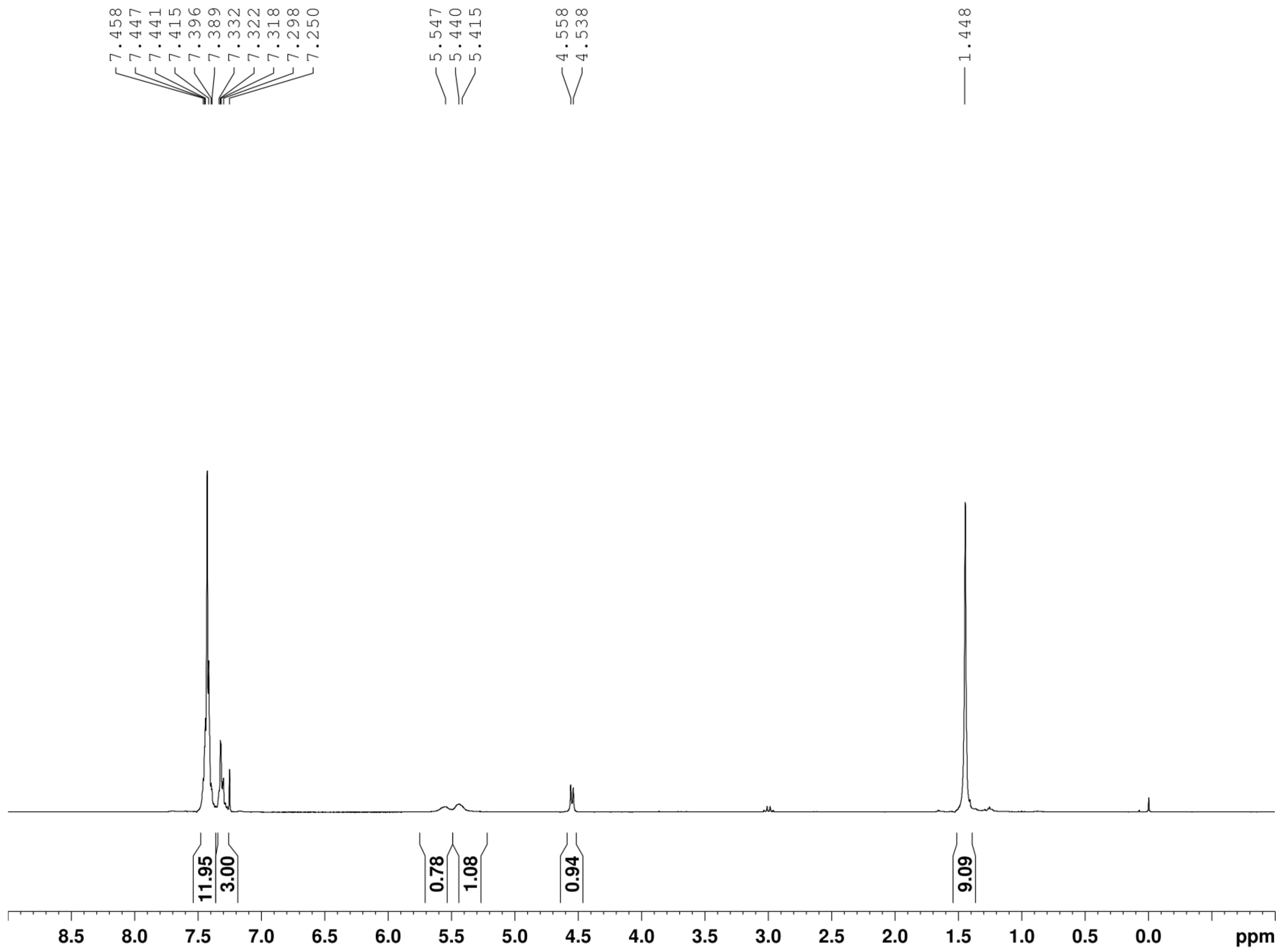

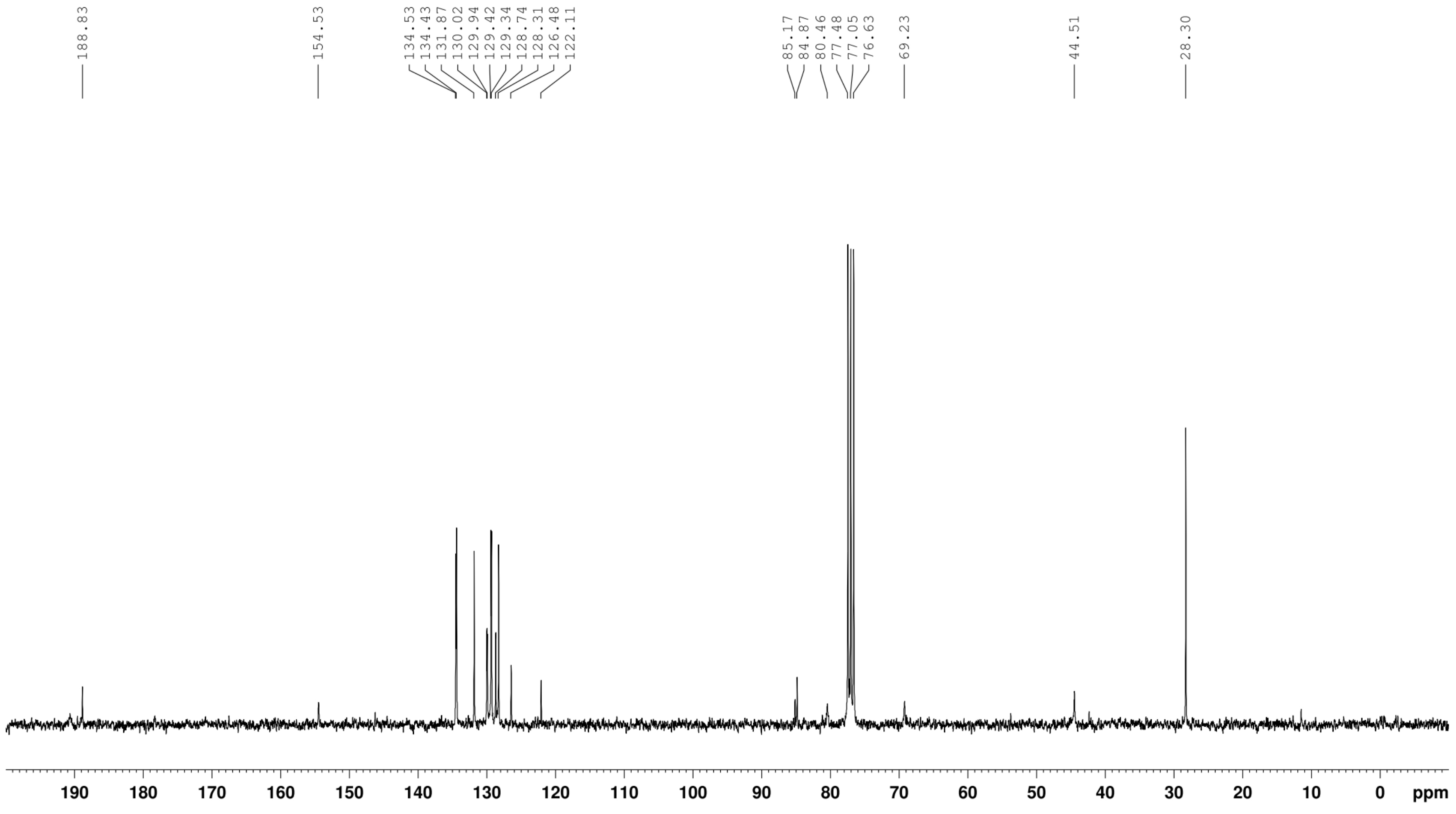

Supplementary Figure 47. ^{1}H and ^{13}C NMR spectra for **3o**.

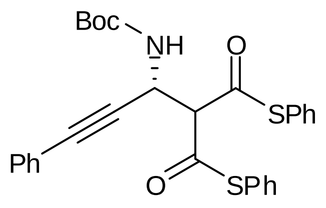

3o

HPLC analysis of **3o**: Daicel CHIRALPAK AS-H, *n*-hexane/*i*-PrOH = 93/7, flow rate = 0.7 mL/min, λ = 254 nm

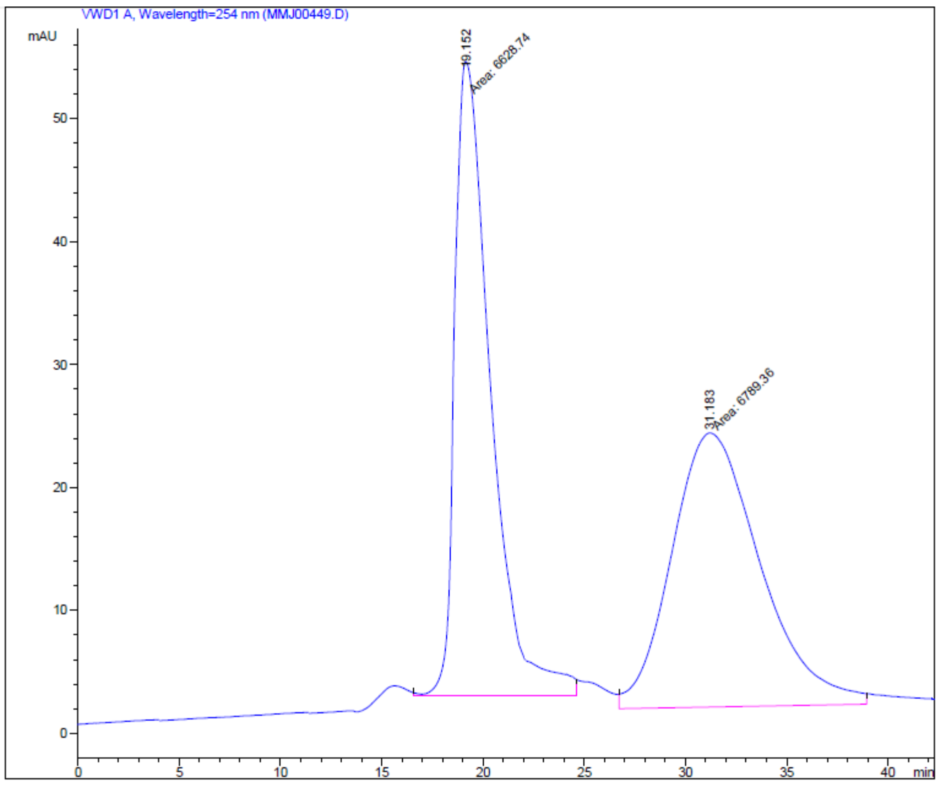

Peak #	RetTime [min]	Type	Width [min]	Area mAU *s	Height [mAU]	Area %
1	19.152	MM	2.1433	6628.74365	51.54685	49.4015
2	31.183	MM	5.0761	6789.36279	22.29206	50.5985
Totals :				1.34181e4	73.83891	

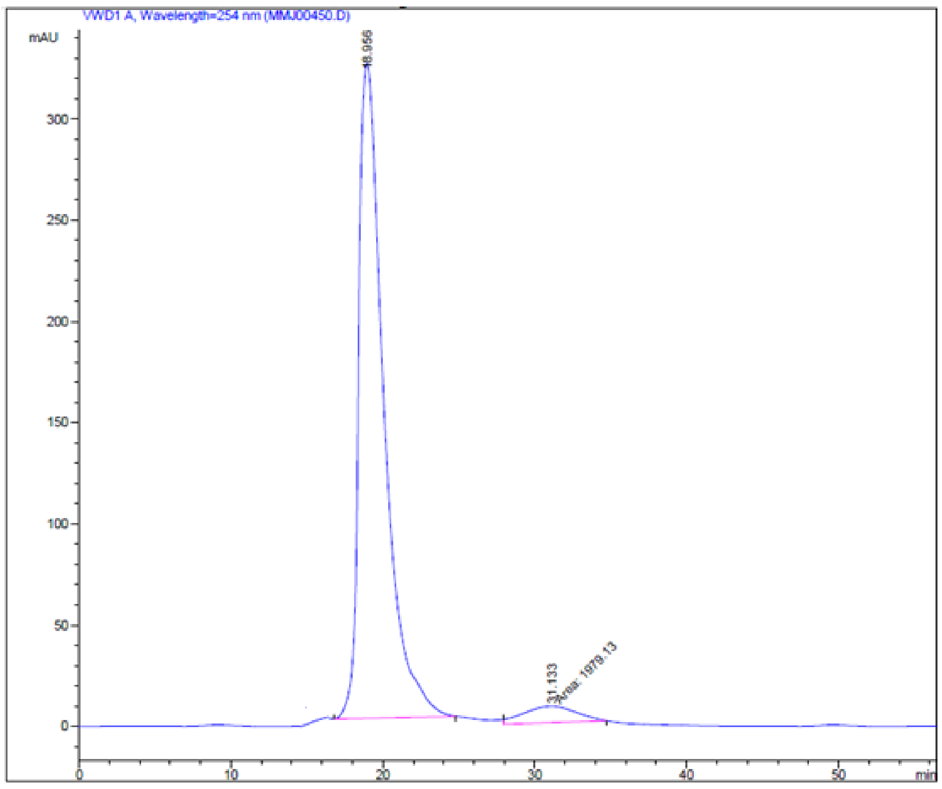

Peak #	RetTime [min]	Type	Width [min]	Area mAU *s	Height [mAU]	Area %
1	18.956	BB	1.6962	3.71298e4	323.75745	94.9394
2	31.133	MM	2.8738	1979.12769	8.07410	5.0606
Totals :				3.91089e4	331.83154	

Supplementary Figure 48. HPLC spectra for **3o**.

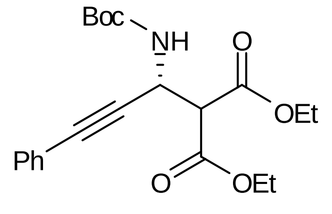

3p

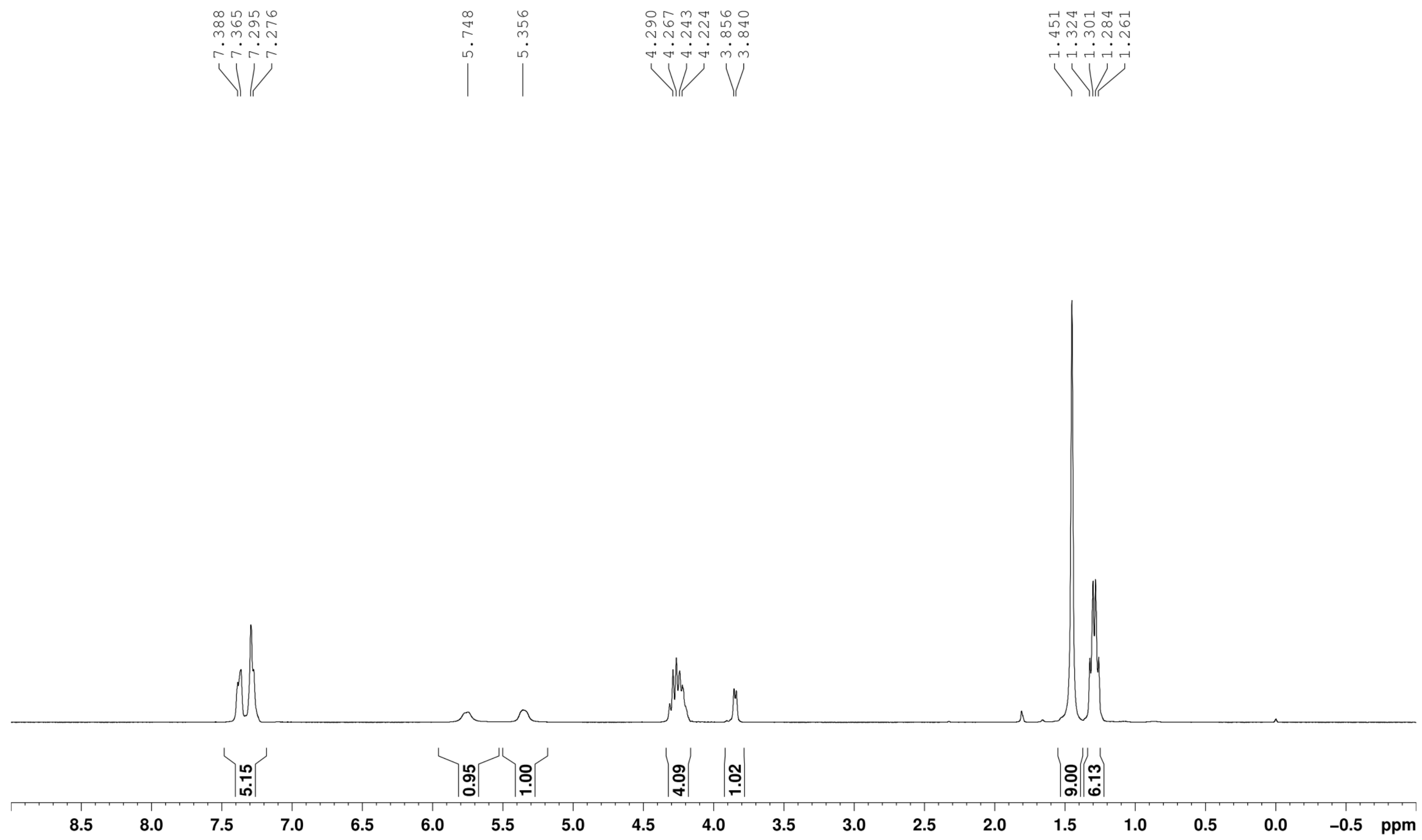

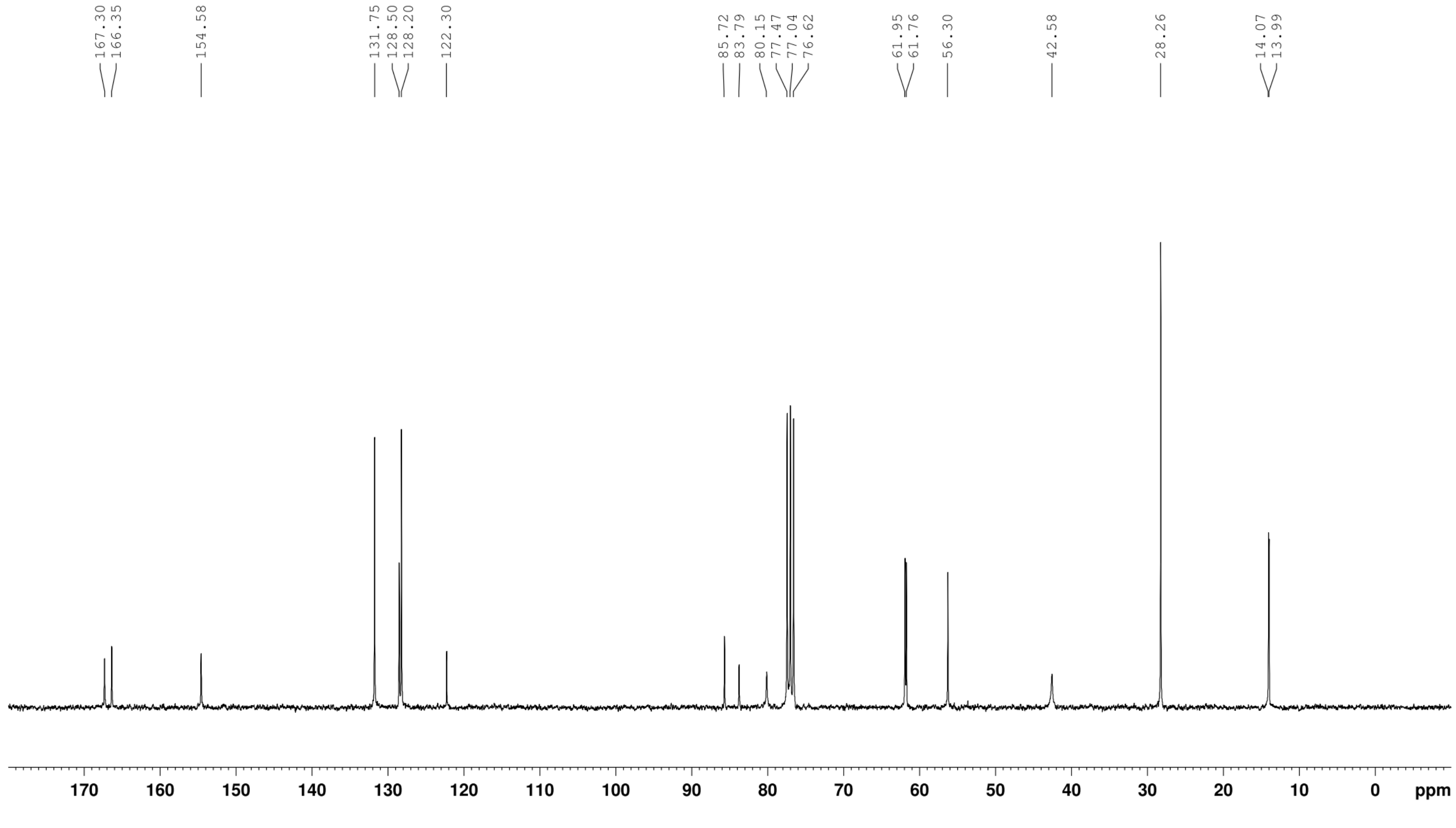

Supplementary Figure 49. ^{1}H and ^{13}C NMR spectra for **3p**.

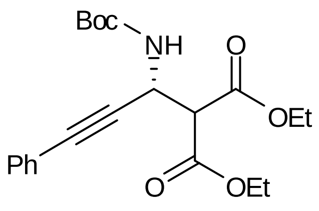

3p

HPLC analysis of **3p**: Daicel CHIRALPAK AS-H, *n*-hexane/*i*-PrOH = 97/3, flow rate = 0.5 mL/min, λ = 254 nm

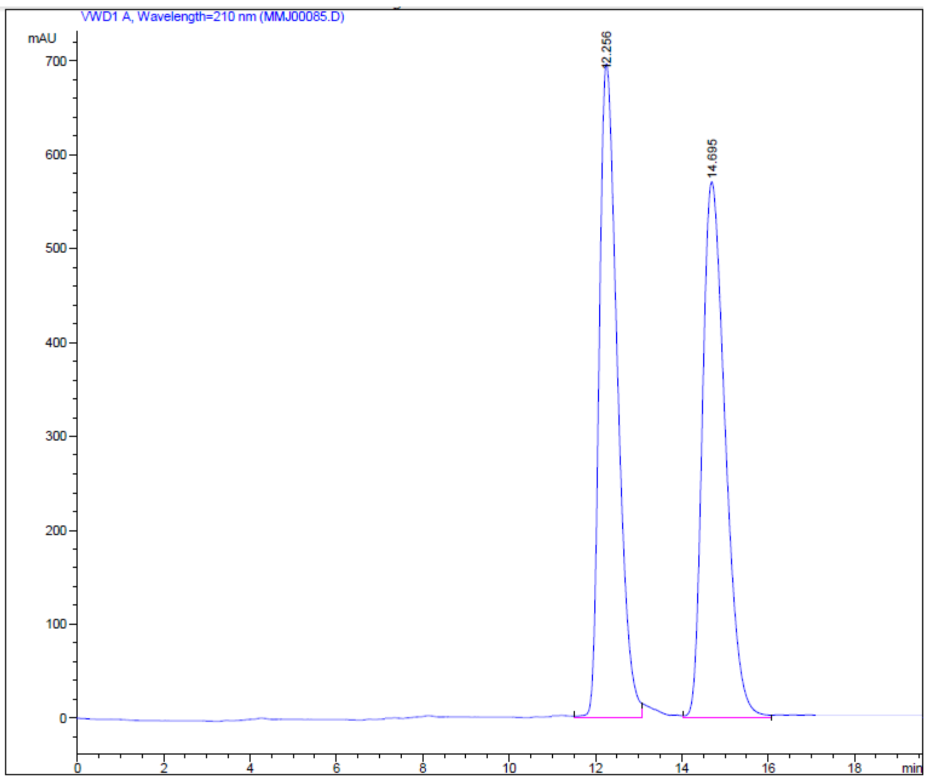

Peak #	RetTime [min]	Type	Width [min]	Area mAU *s	Height [mAU]	Area %
1	12.256	VV	0.4547	2.04764e4	697.24951	49.4499
2	14.695	VV	0.5731	2.09320e4	571.18616	50.5501
Totals :				4.14084e4	1268.43567	

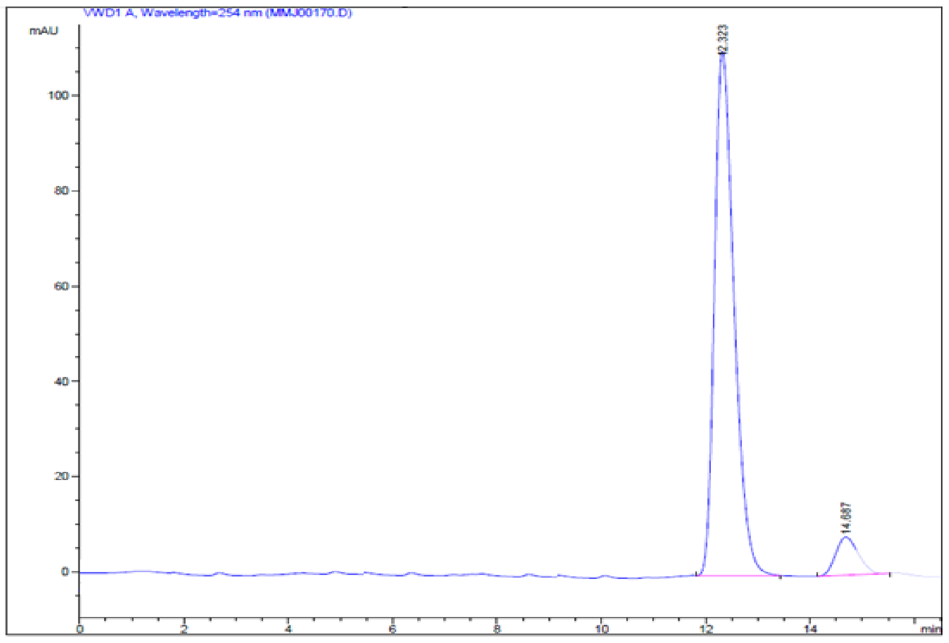

Peak #	RetTime [min]	Type	Width [min]	Area mAU *s	Height [mAU]	Area %
1	12.323	VB	0.3992	2875.02954	110.14690	92.2909
2	14.687	PB	0.4619	240.15135	8.00911	7.7091
Totals :				3115.18089	118.15602	

Supplementary Figure 50. HPLC spectra for **3p**.

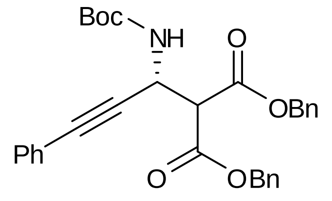

3q

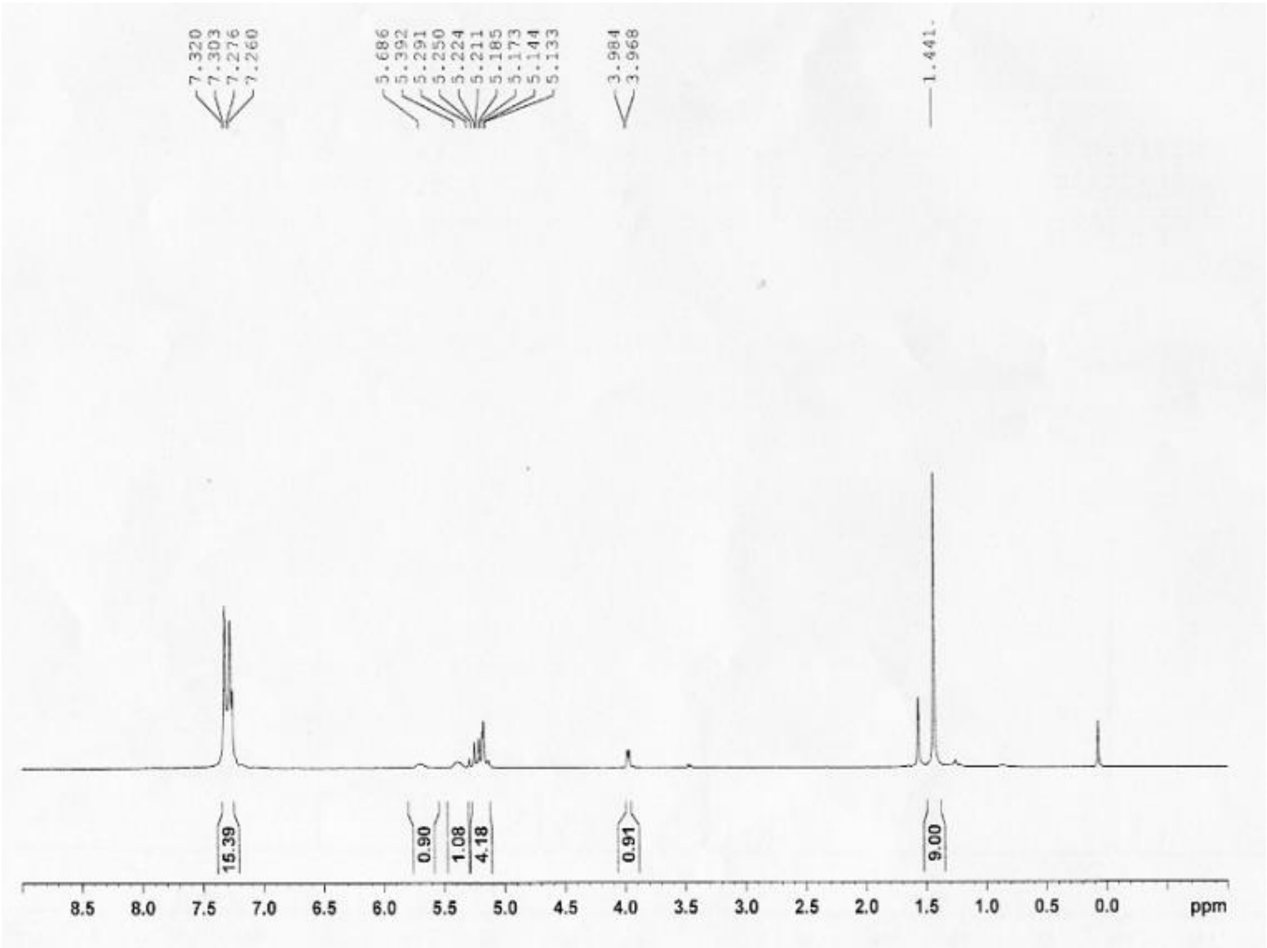

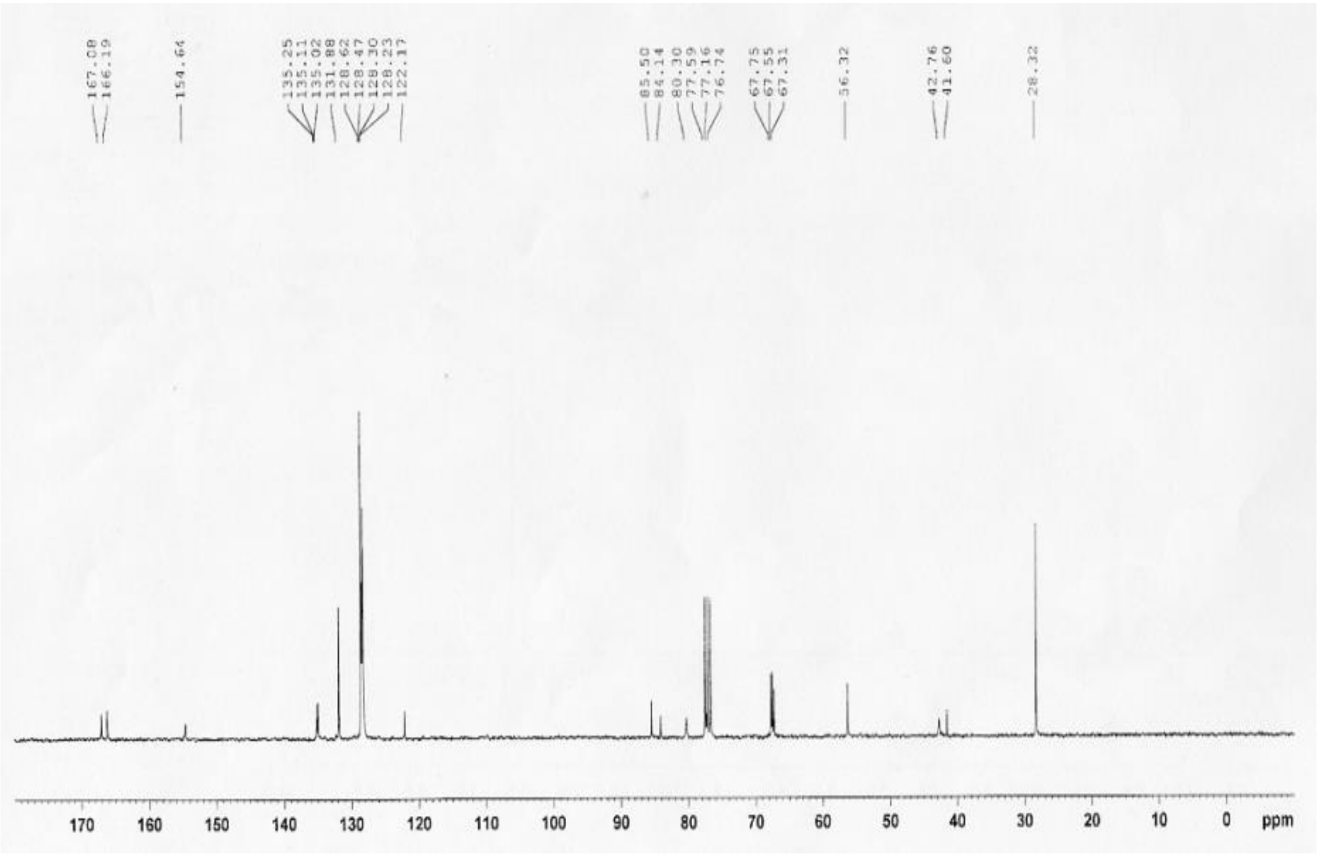

Supplementary Figure 51. ^{1}H and ^{13}C NMR spectra for **3q**.

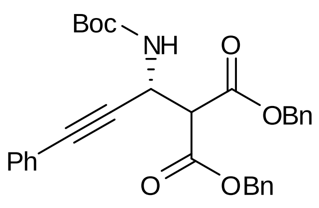

3q

HPLC analysis of **3q**: Daicel CHIRALPAK AS-H, *n*-hexane/*i*-PrOH = 96/4, flow rate = 0.7 mL/min, λ = 254 nm

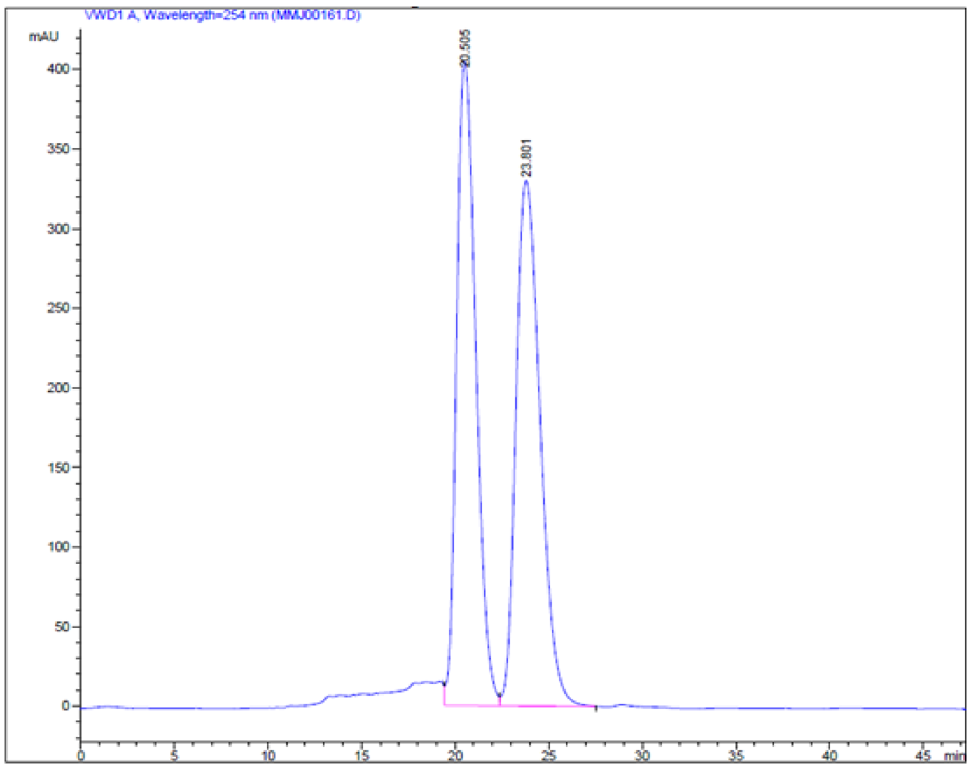

Peak #	RetTime [min]	Type	Width [min]	Area mAU *s	Height [mAU]	Area %
1	20.505	VV	1.1270	2.89862e4	404.59930	49.4052
2	23.801	VB	1.4085	2.96842e4	330.16559	50.5948
Totals :				5.86703e4	734.76489	

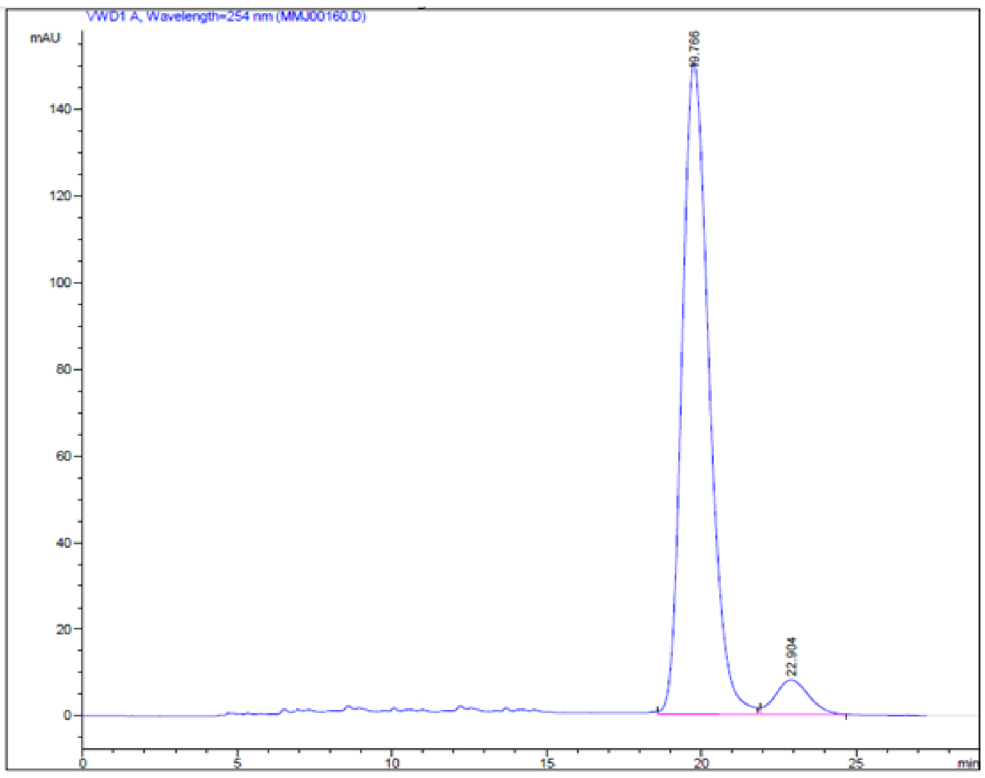

Peak #	RetTime [min]	Type	Width [min]	Area mAU *s	Height [mAU]	Area %
1	19.766	BB	0.9369	9116.44824	150.46167	93.7680
2	22.904	BB	1.0467	605.89386	8.03919	6.2320
Totals :				9722.34210	158.50086	

Supplementary Figure 52. HPLC spectra for **3q**.

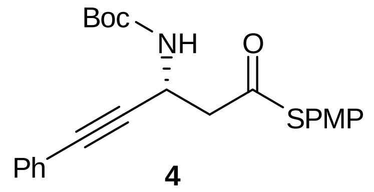

4

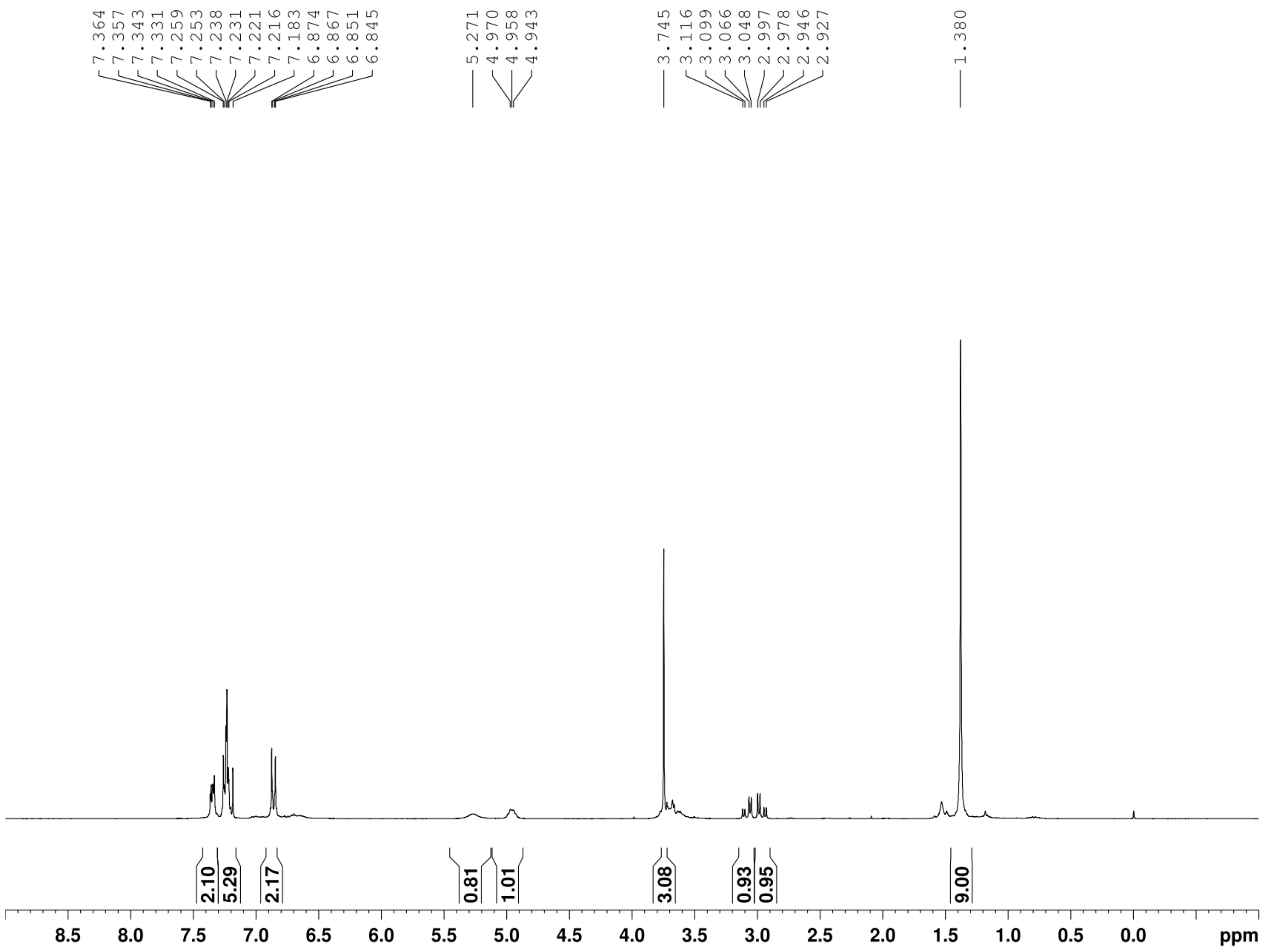

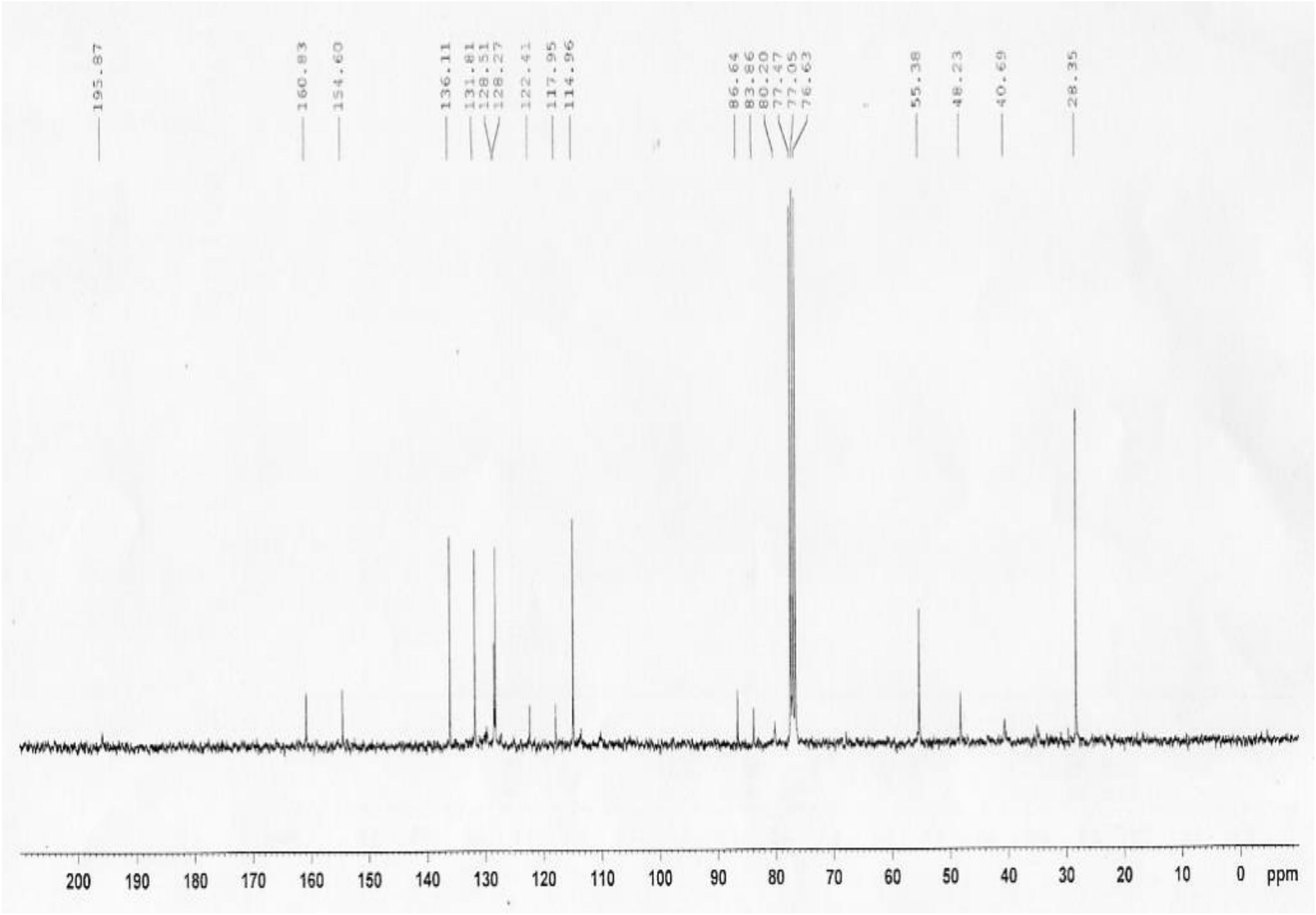

Supplementary Figure 53. ^{1}H and ^{13}C NMR spectra for **4**.

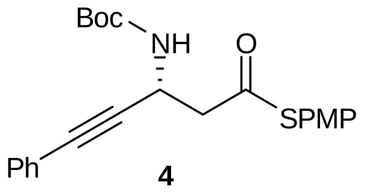

4

HPLC analysis of **4**: Daicel CHIRALPAK AD-H, *n*-hexane/*i*-PrOH = 85/15, flow rate = 0.8 mL/min, λ = 254 nm

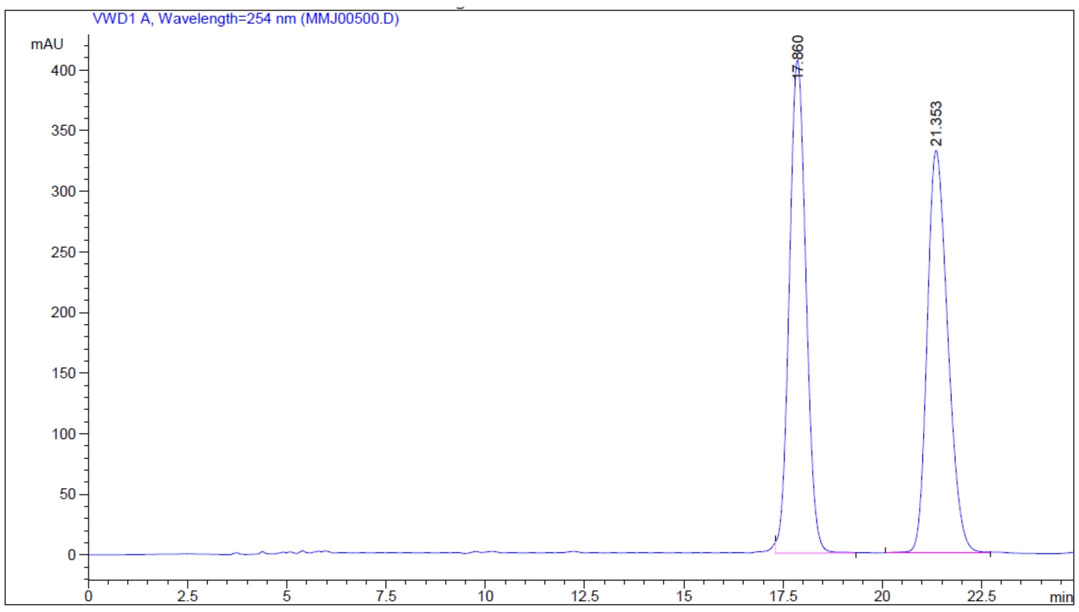

Peak #	RetTime [min]	Type	Width [min]	Area mAU *s	Height [mAU]	Area %
1	17.860	VB	0.4578	1.19025e4	406.87598	49.8403
2	21.353	BB	0.5606	1.19787e4	332.06393	50.1597
Totals :				2.38812e4	738.93991	

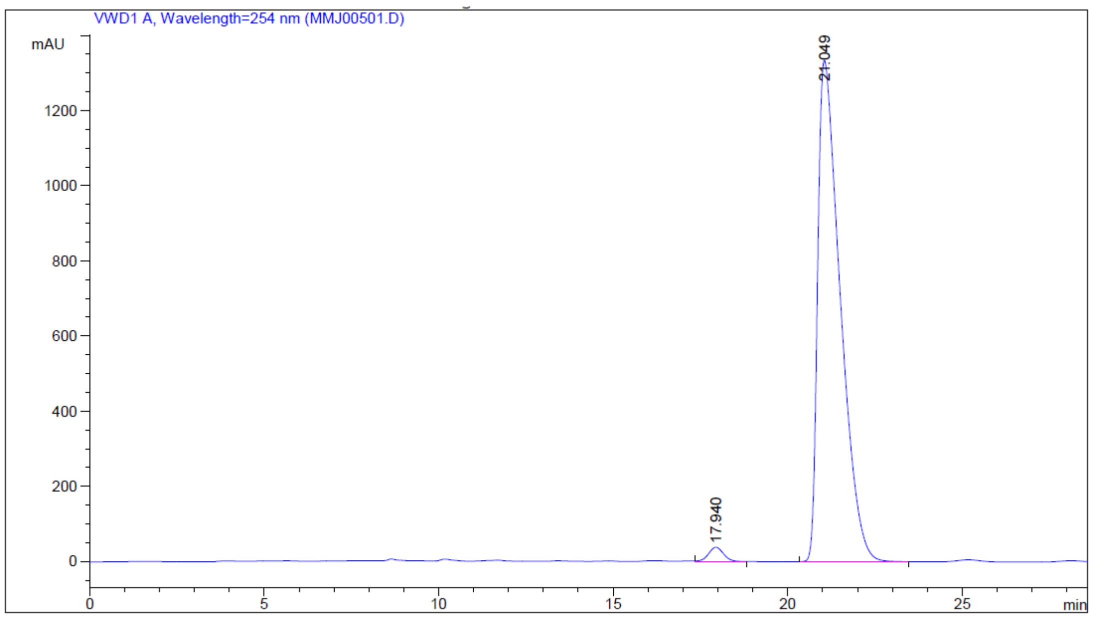

Peak #	RetTime [min]	Type	Width [min]	Area mAU *s	Height [mAU]	Area %
1	17.940	VB	0.4620	1129.79724	37.82521	1.8611
2	21.049	BB	0.6767	5.95764e4	1334.27625	98.1389
Totals :				6.07062e4	1372.10146	

Supplementary Figure 54. HPLC spectra for **4**.

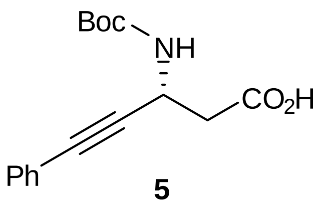

5

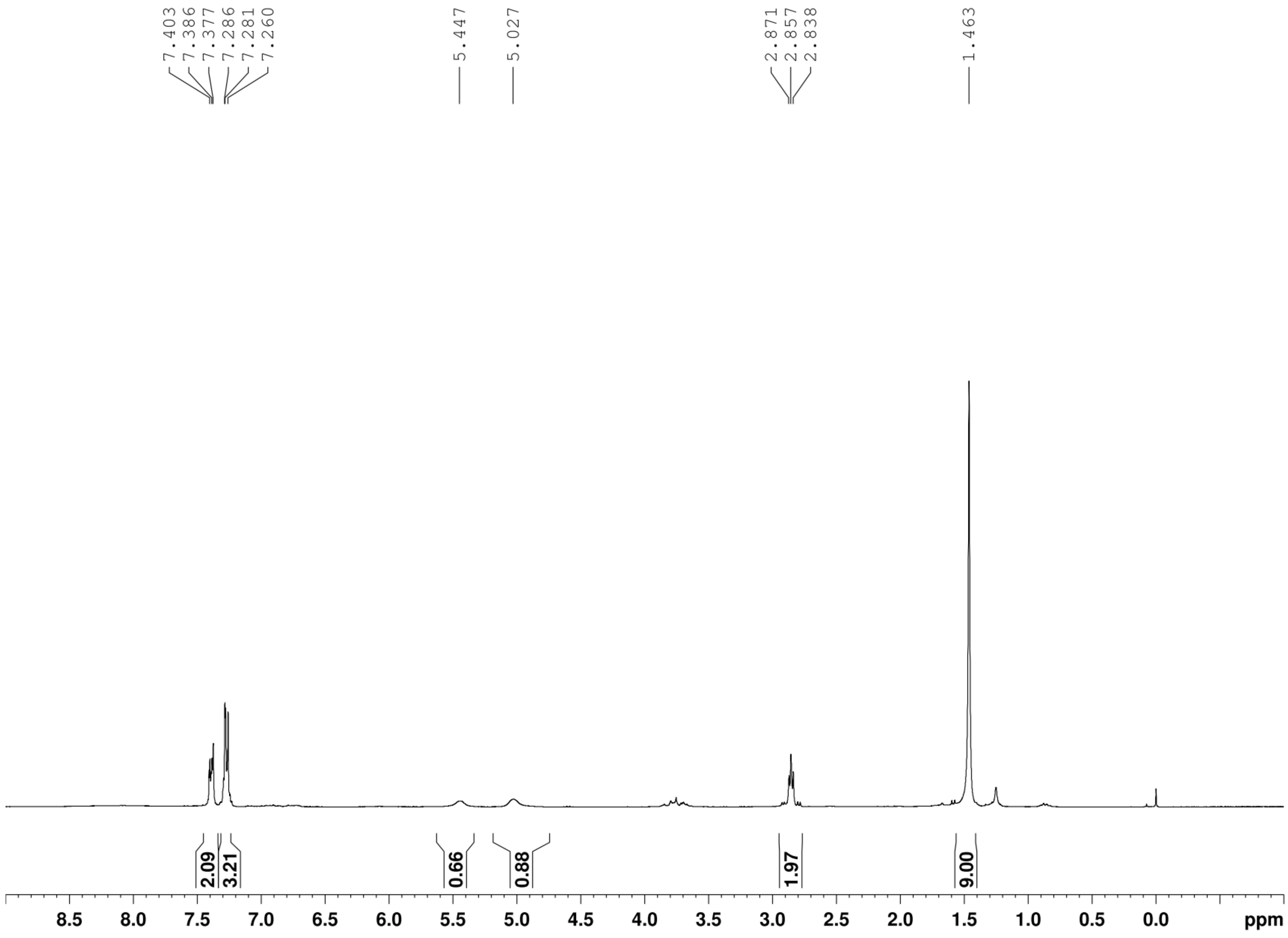

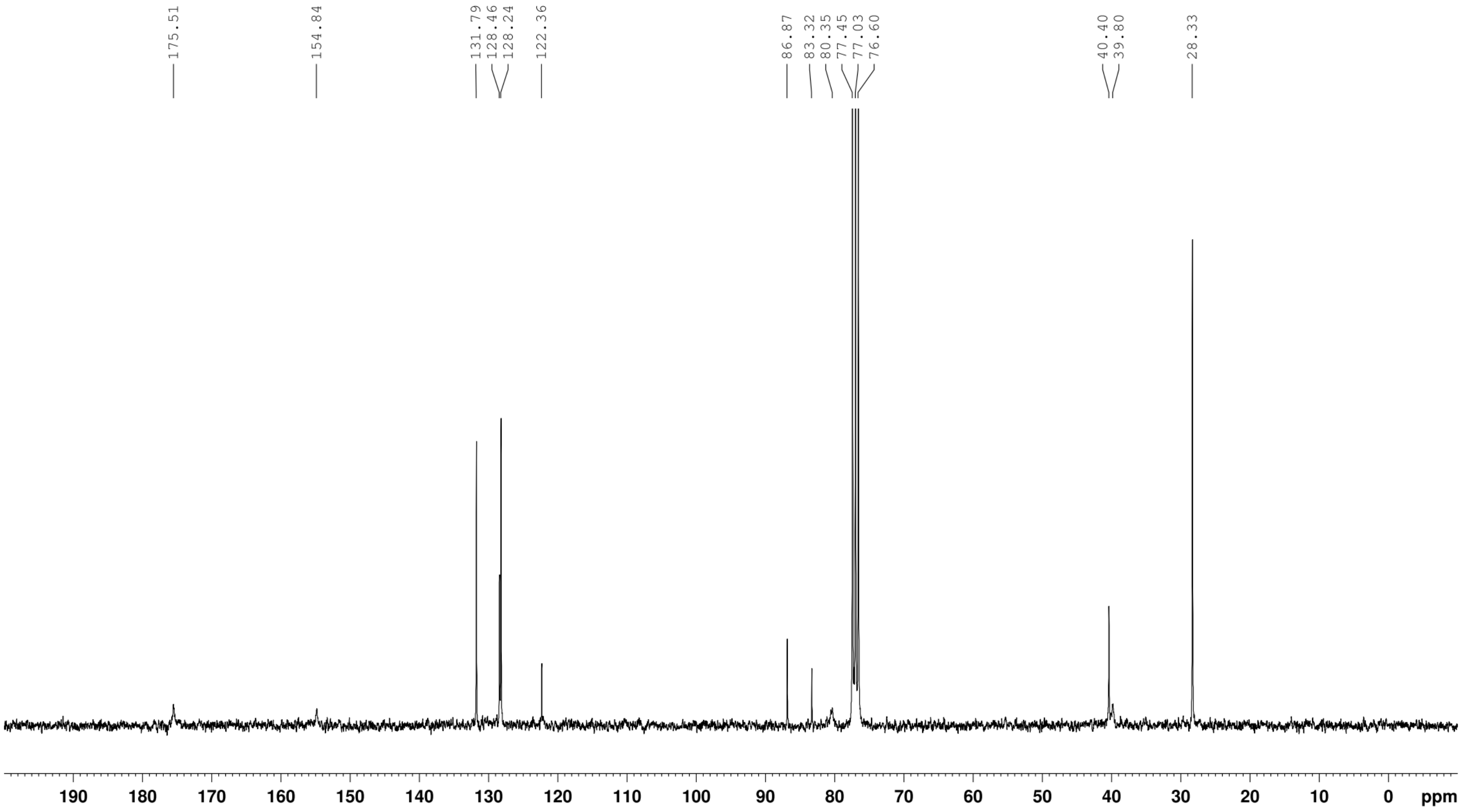

Supplementary Figure 55. ^{1}H and ^{13}C NMR spectra for **5**.

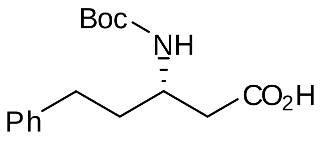

6

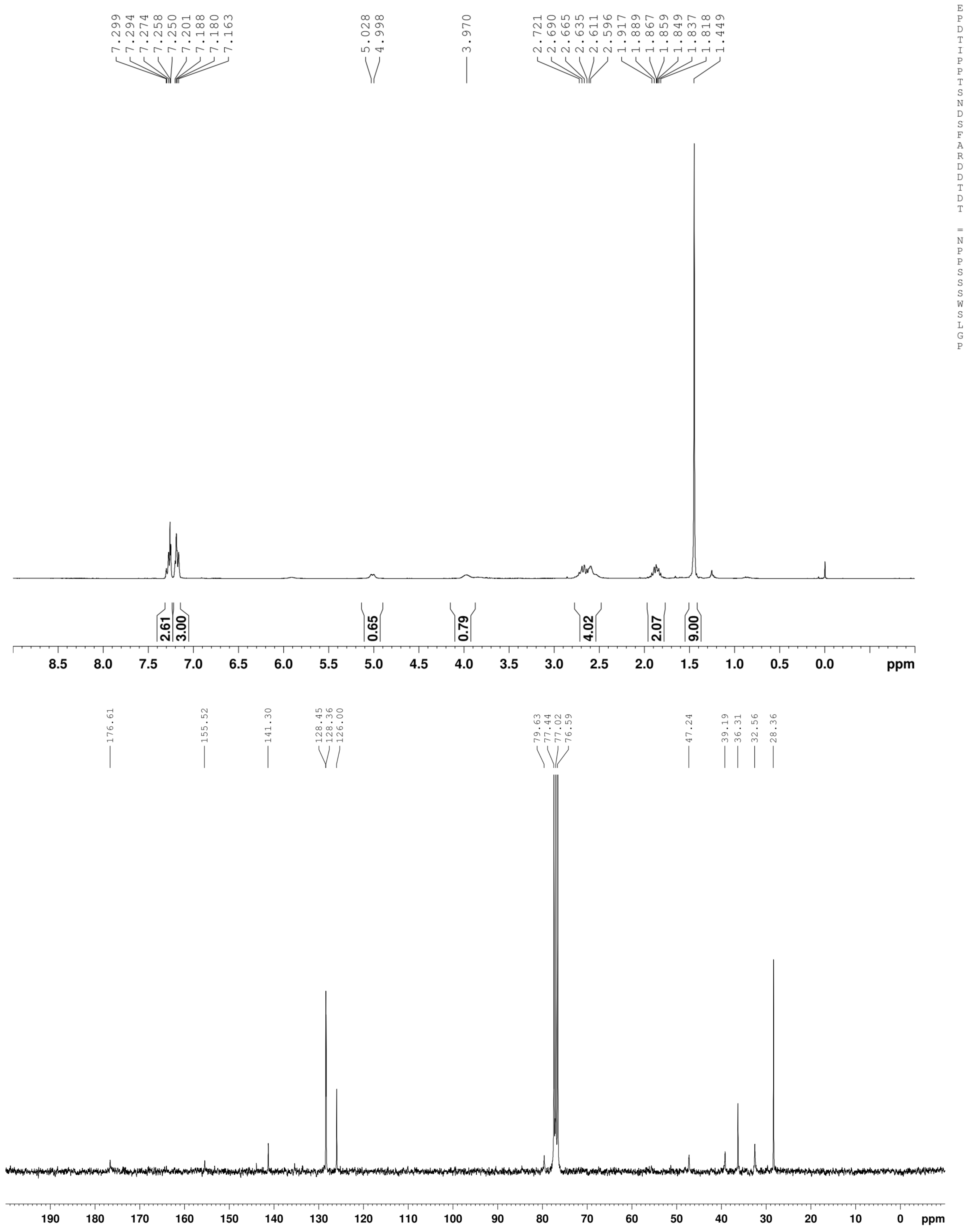

Supplementary Figure 56. ^{1}H and ^{13}C NMR spectra for **6**.

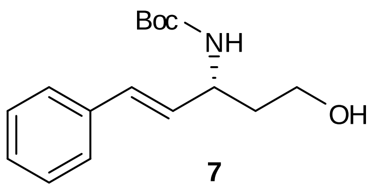

7

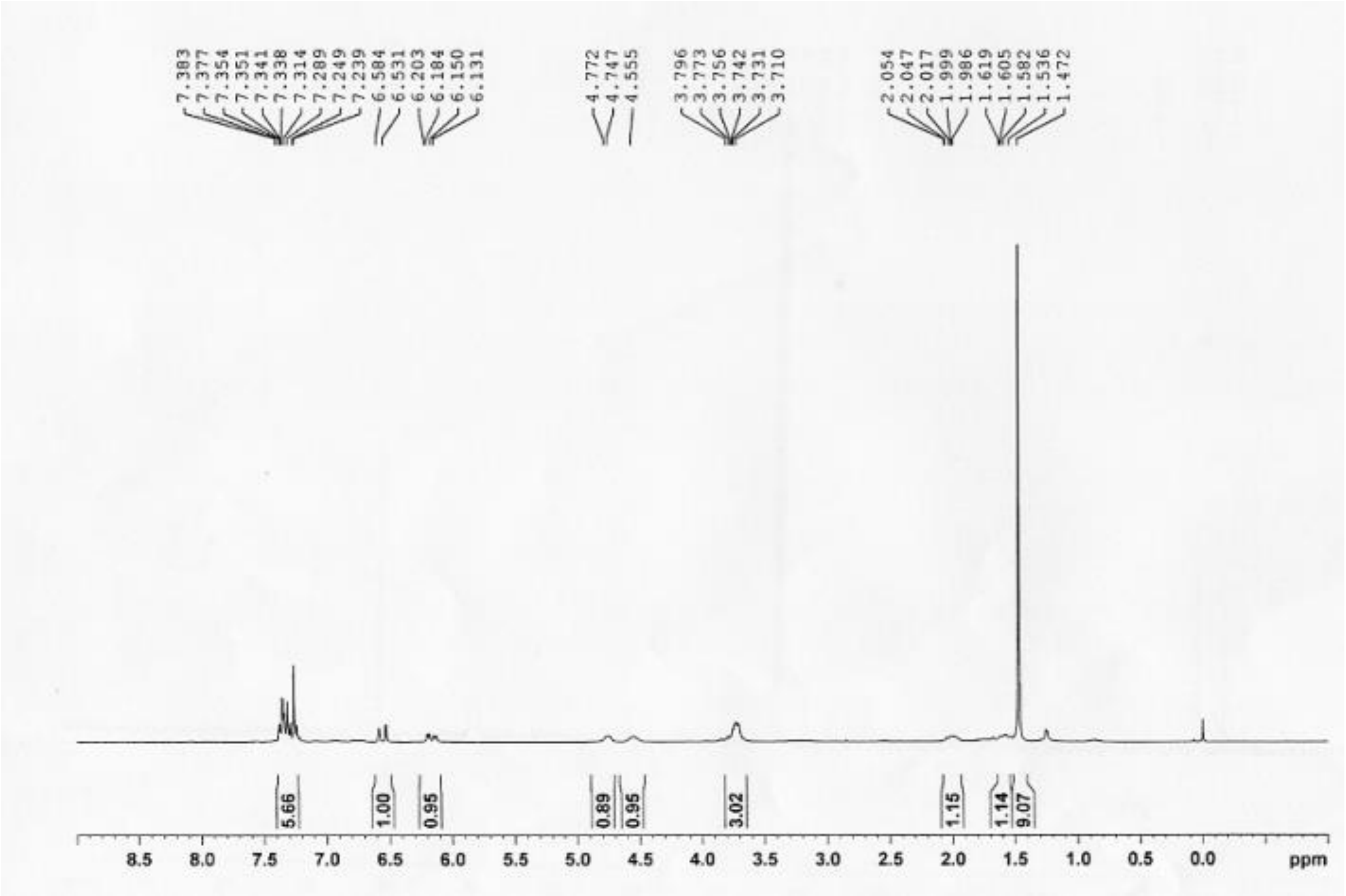

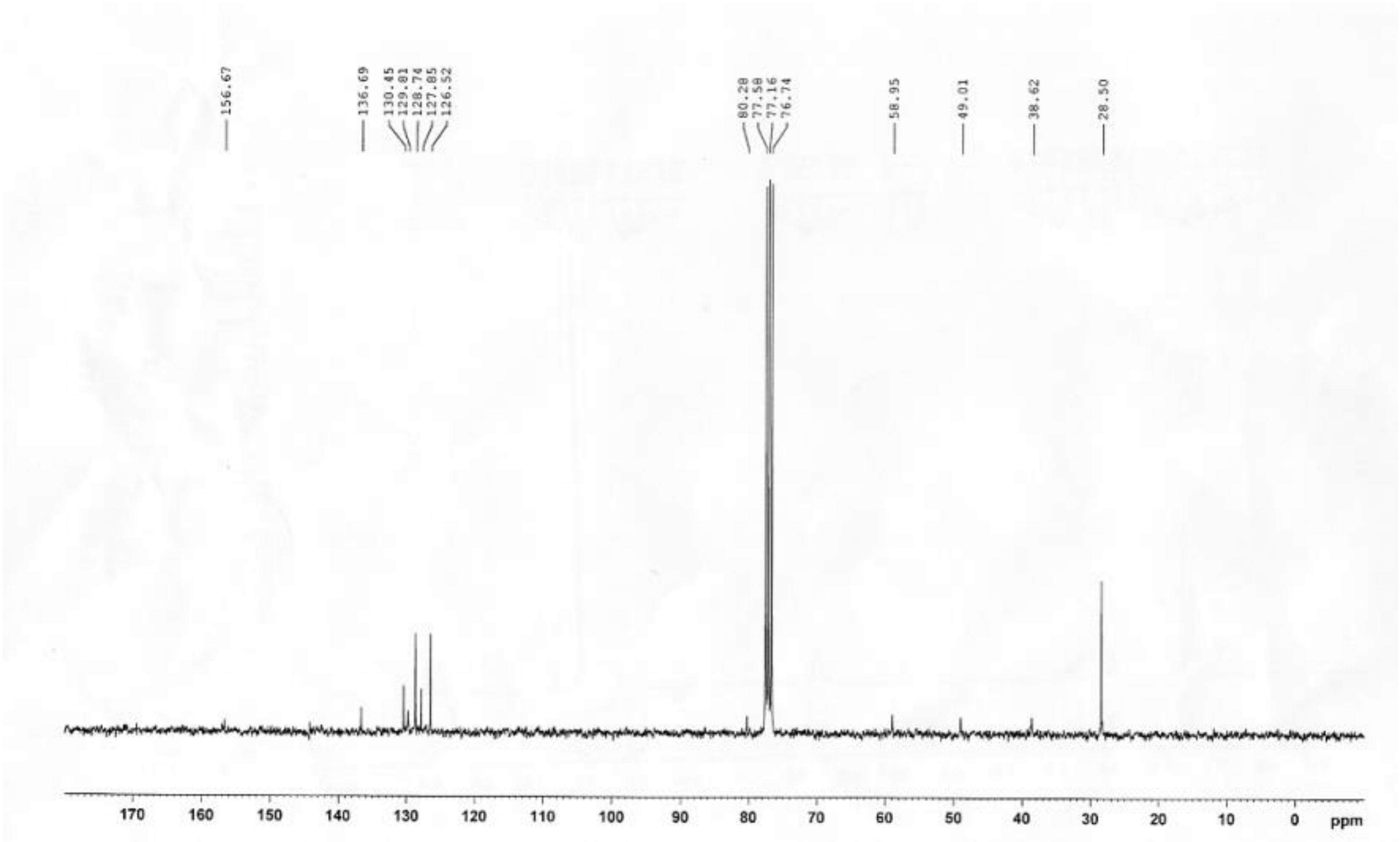

Supplementary Figure 57. ^{1}H and ^{13}C NMR spectra for **7**.

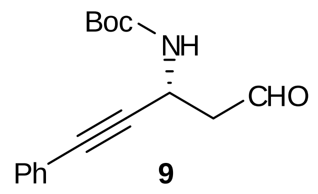

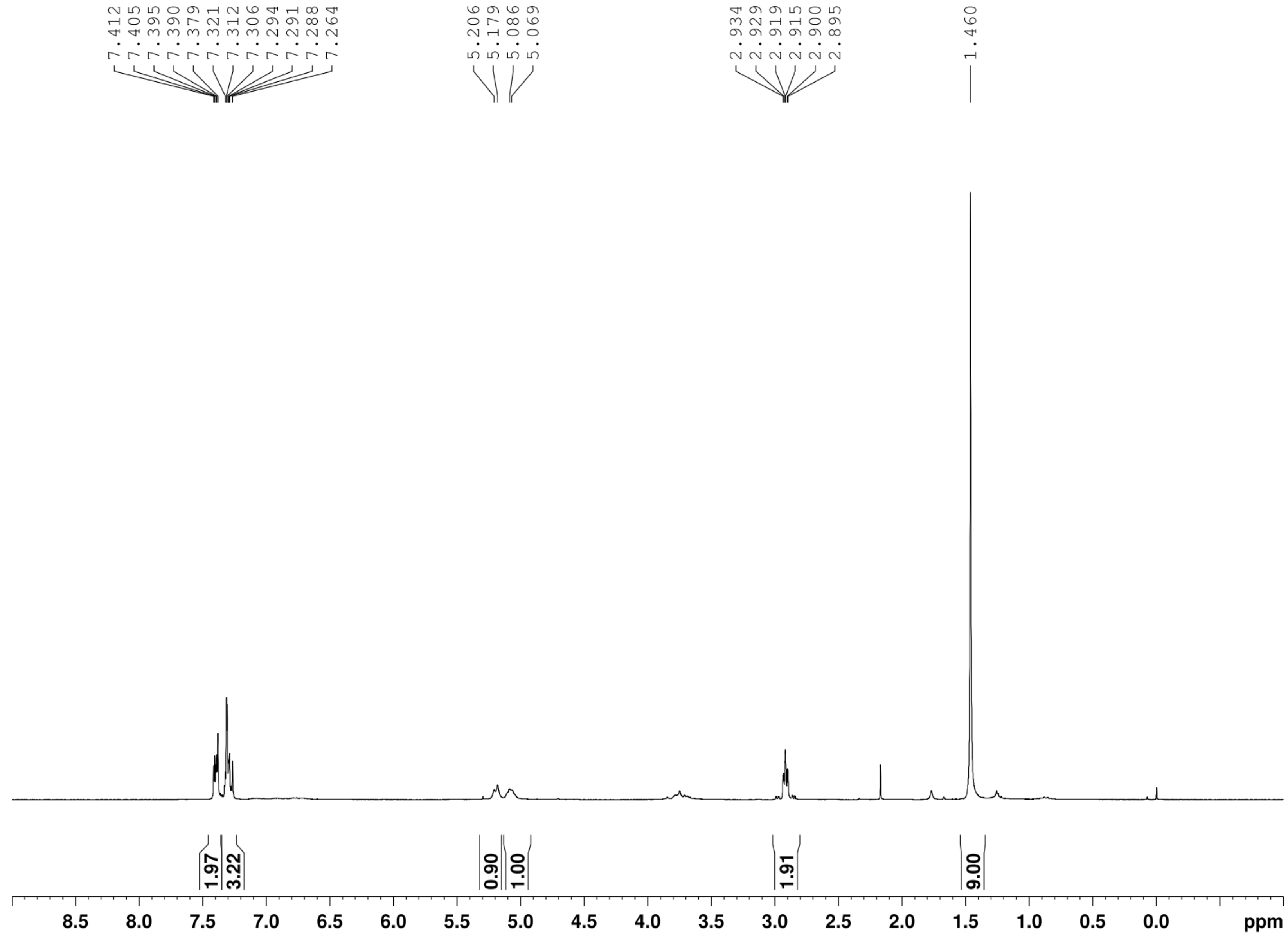

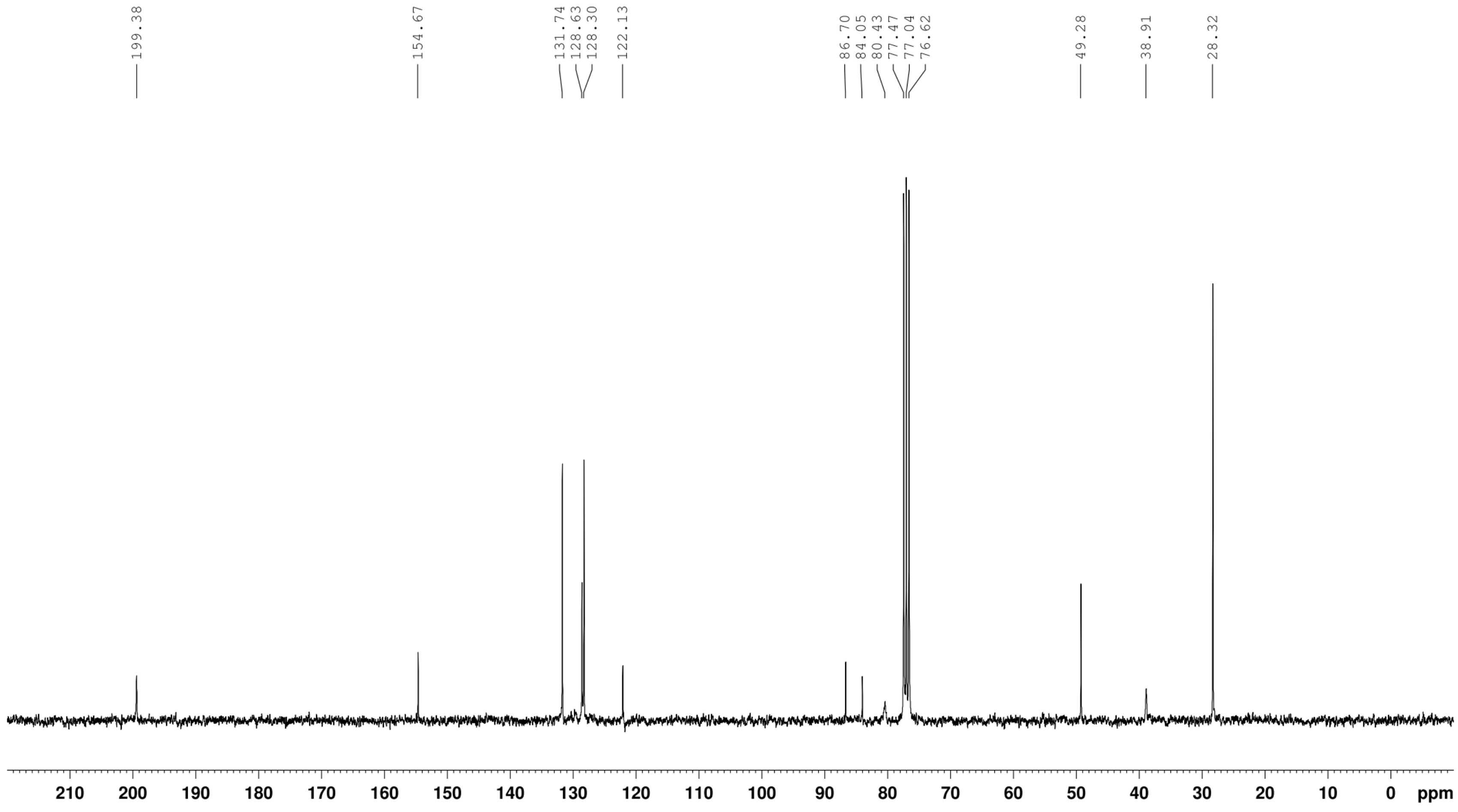

Supplementary Figure 58. ^{1}H and ^{13}C NMR spectra for **9**.

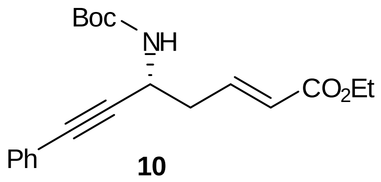

10

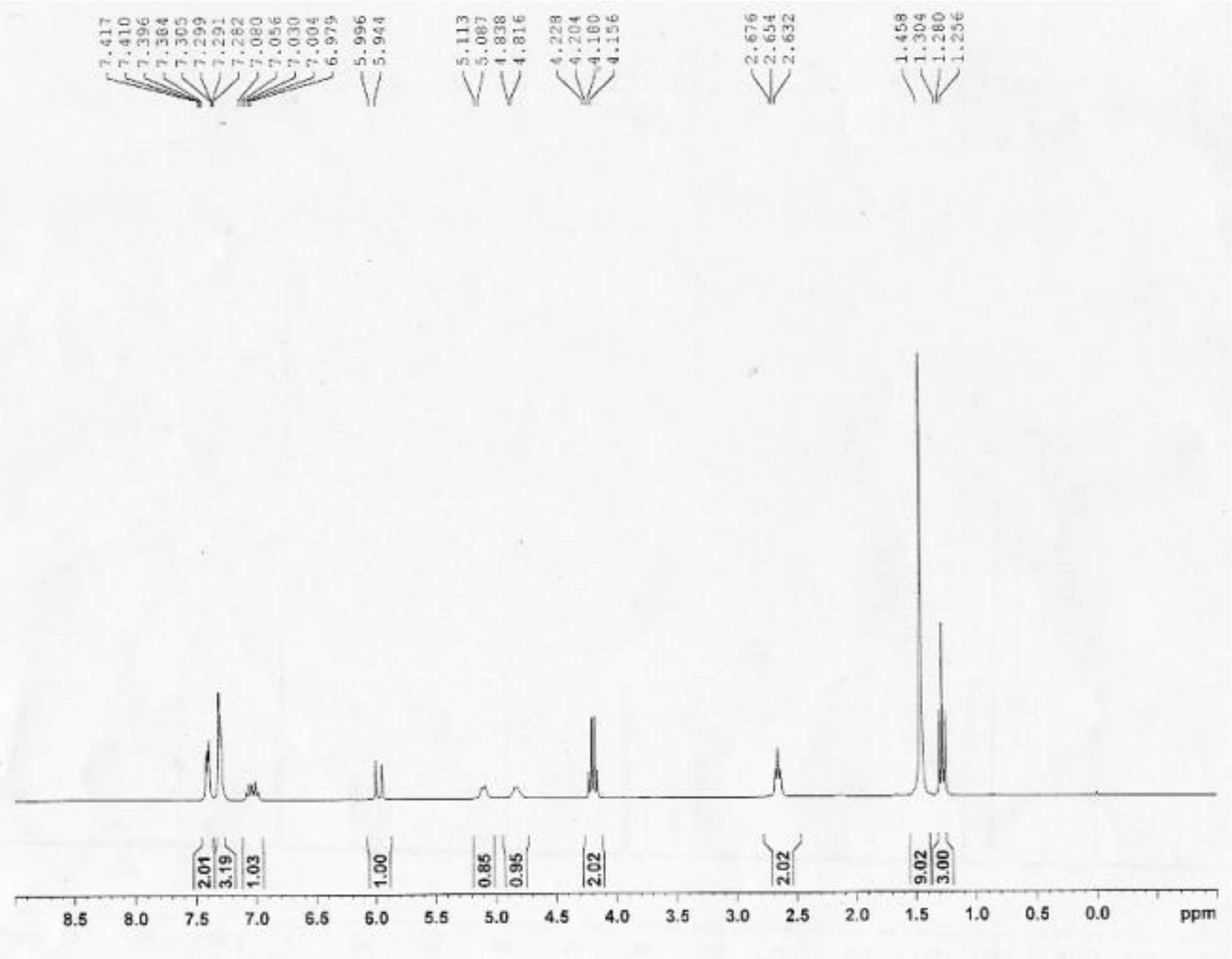

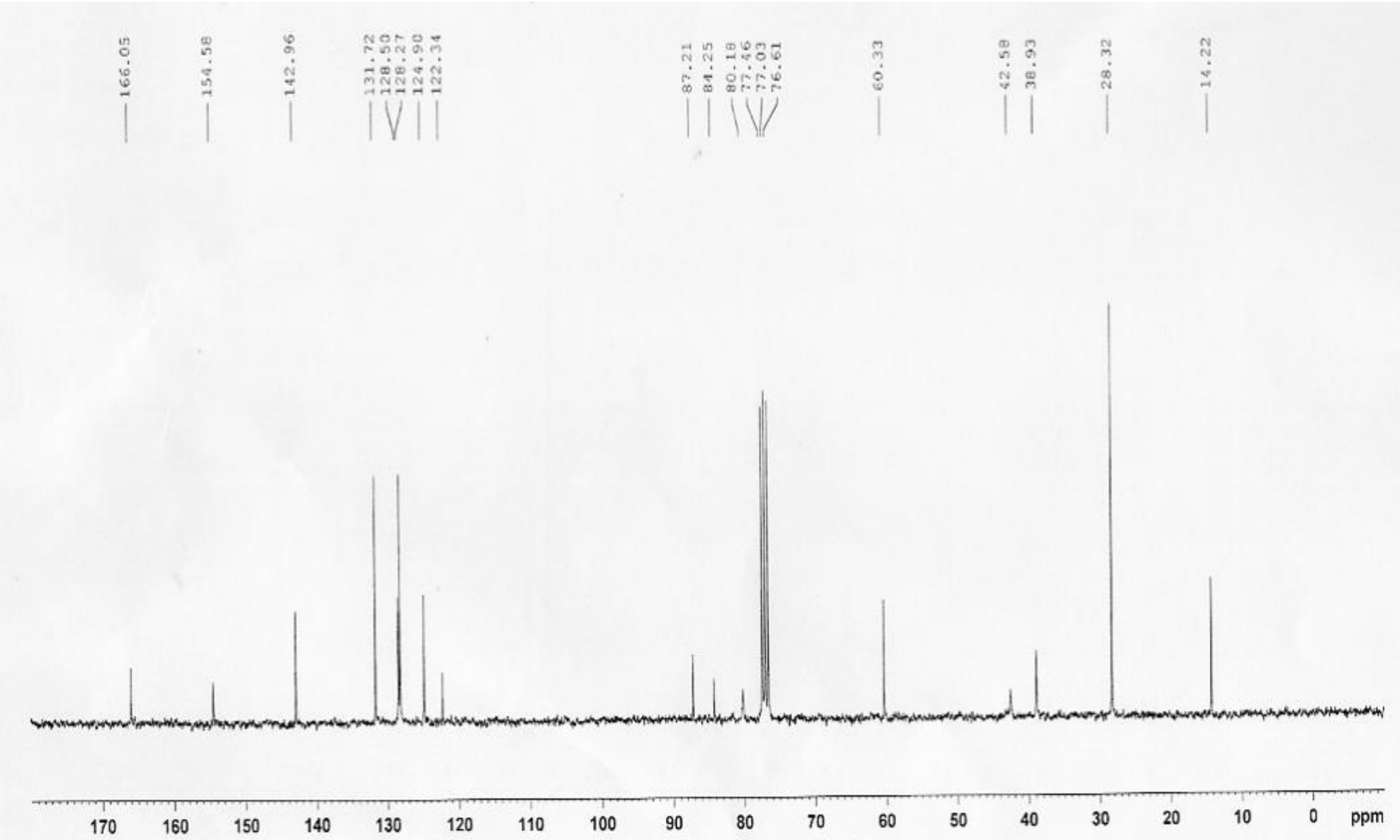

Supplementary Figure 59. ^{1}H and ^{13}C NMR spectra for **10**.

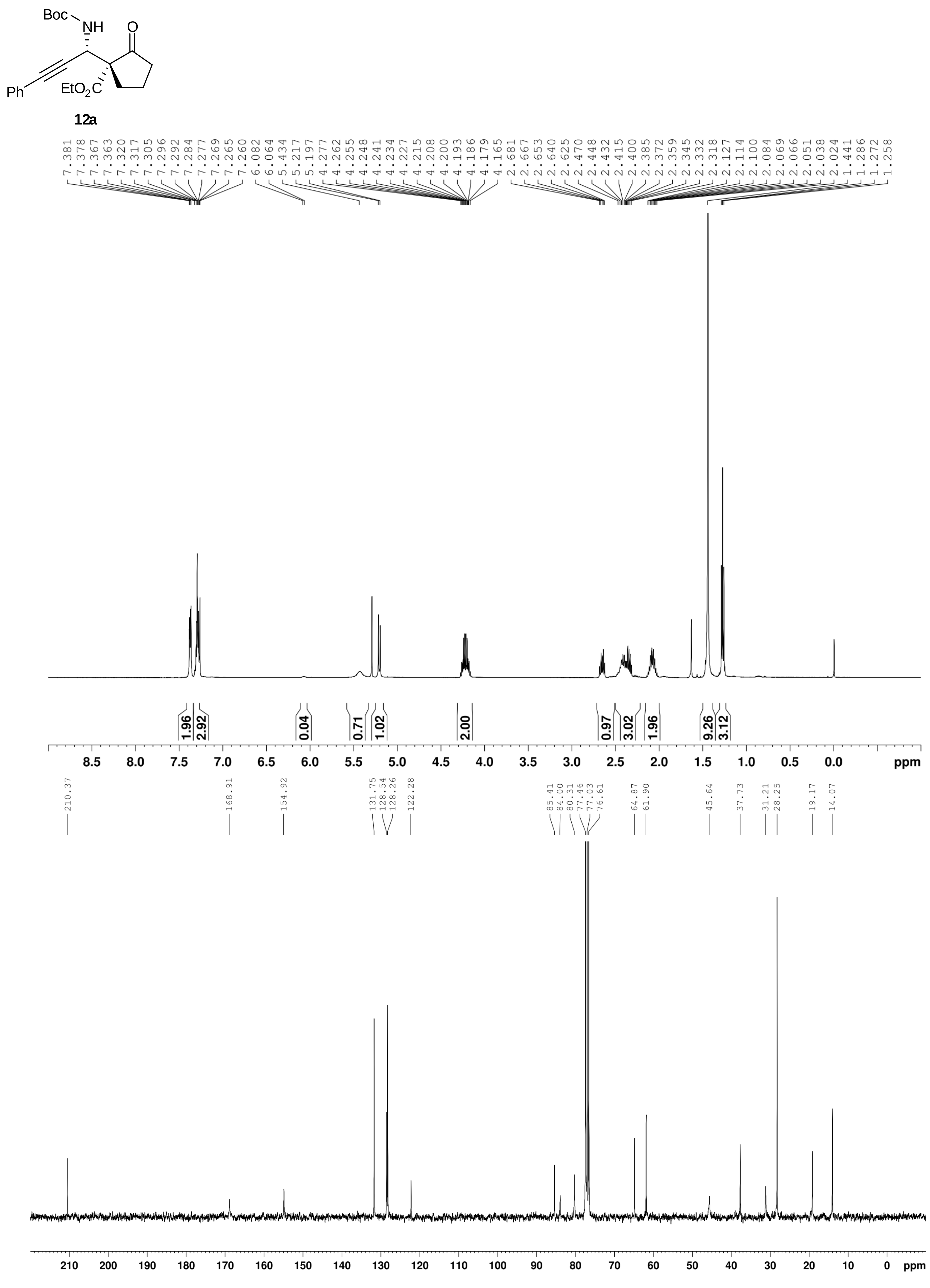

Supplementary Figure 60. ^{1}H and ^{13}C NMR spectra for **12a**.

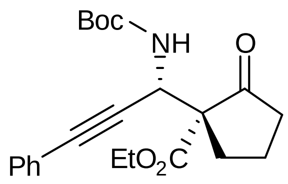

12a

HPLC analysis of **12a**: Daicel CHIRALPAK AD-H, *n*-hexane/*i*-PrOH = 97/3, flow rate = 0.8 mL/min, λ = 254 nm

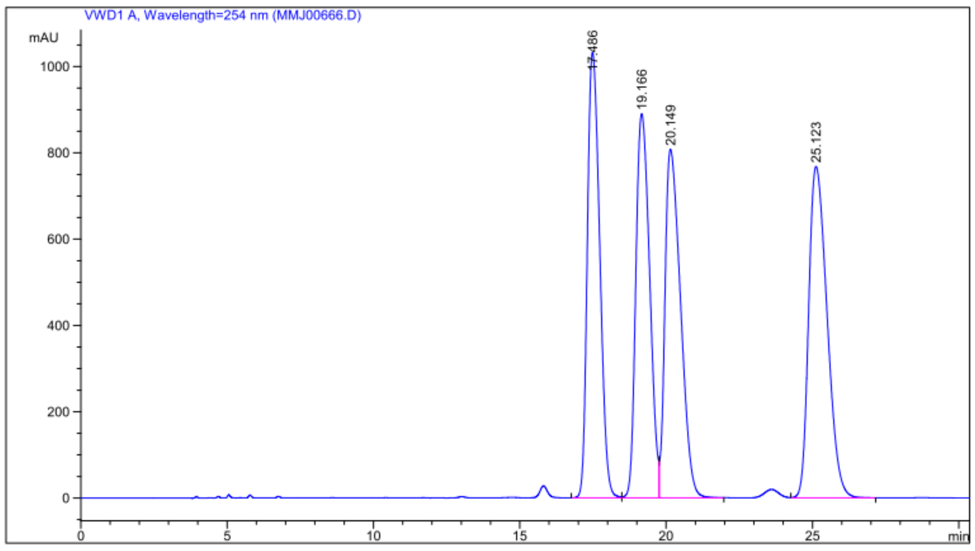

Peak #	RetTime [min]	Type	Width [min]	Area mAU *s	Height [mAU]	Area %
1	17.486	BV	0.4700	3.02638e4	1032.80713	24.6552
2	19.166	VV	0.5341	2.91876e4	889.86157	23.7785
3	20.149	VB	0.5944	2.98599e4	807.64014	24.3261
4	25.123	VB	0.6914	3.34368e4	767.97577	27.2401
Totals :				1.22748e5	3498.28461	

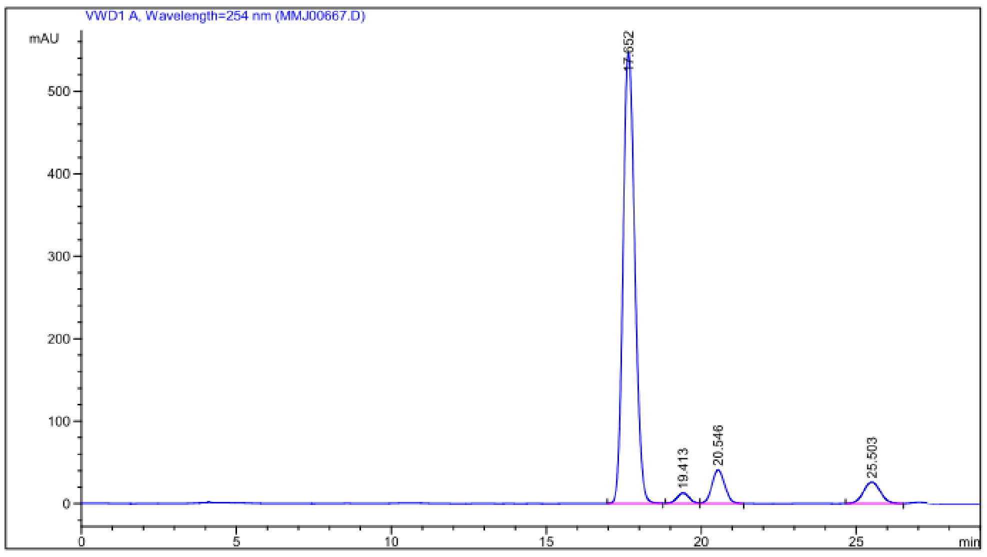

Peak #	RetTime [min]	Type	Width [min]	Area mAU *s	Height [mAU]	Area %
1	17.652	BB	0.4158	1.44228e4	546.17261	85.0194
2	19.413	BV	0.4389	356.87811	12.68463	2.1037
3	20.546	VB	0.4489	1179.56323	41.03613	6.9533
4	25.503	BB	0.6007	1004.89453	26.26292	5.9236
Totals :				1.69642e4	626.15629	

Supplementary Figure 61. HPLC spectra for **12a**.

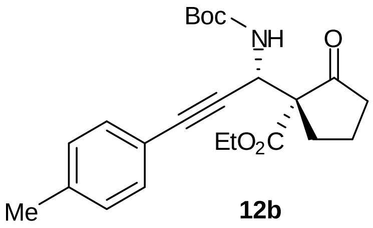

12b

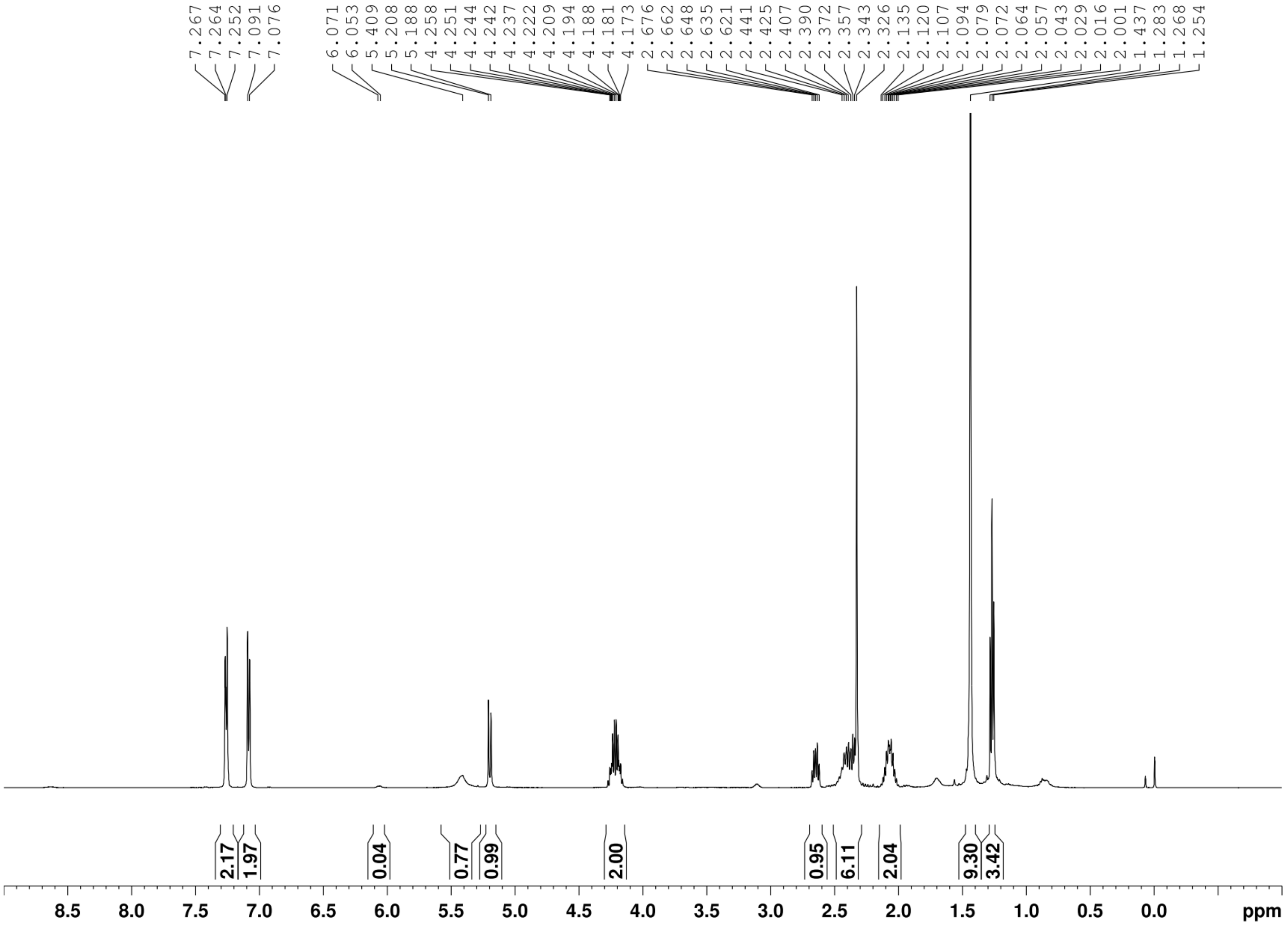

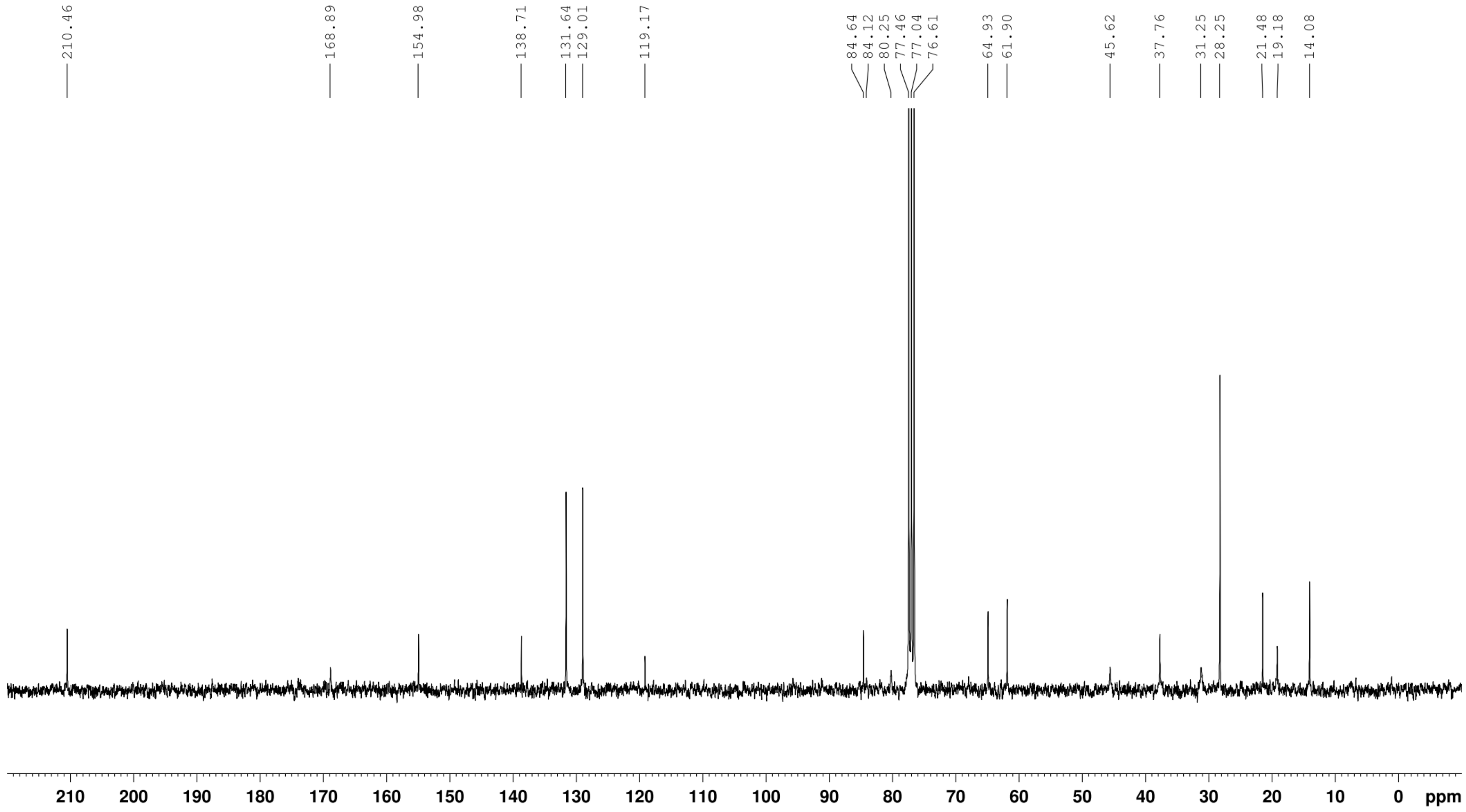

Supplementary Figure 62. ^{1}H and ^{13}C NMR spectra for **12b**.

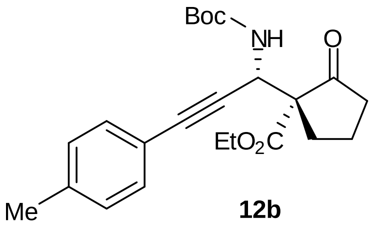

12b

HPLC analysis of **12b**: Daicel CHIRALPAK AD-H, *n*-hexane/*i*-PrOH = 97/3, flow rate = 0.8 mL/min, λ = 254 nm

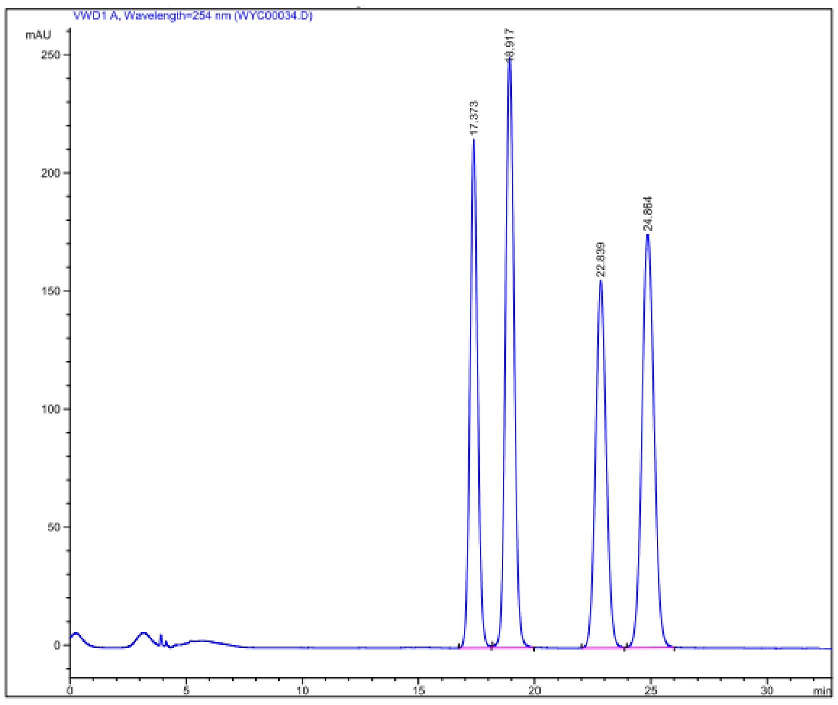

Peak #	RetTime [min]	Type	Width [min]	Area mAU *s	Height [mAU]	Area %
1	17.373	BB	0.3567	4972.94141	215.36708	22.0489
2	18.917	BB	0.3952	6371.79443	249.78671	28.2511
3	22.839	BB	0.4937	4934.50879	155.61226	21.8785
4	24.864	BB	0.5568	6274.92822	174.94356	27.8216

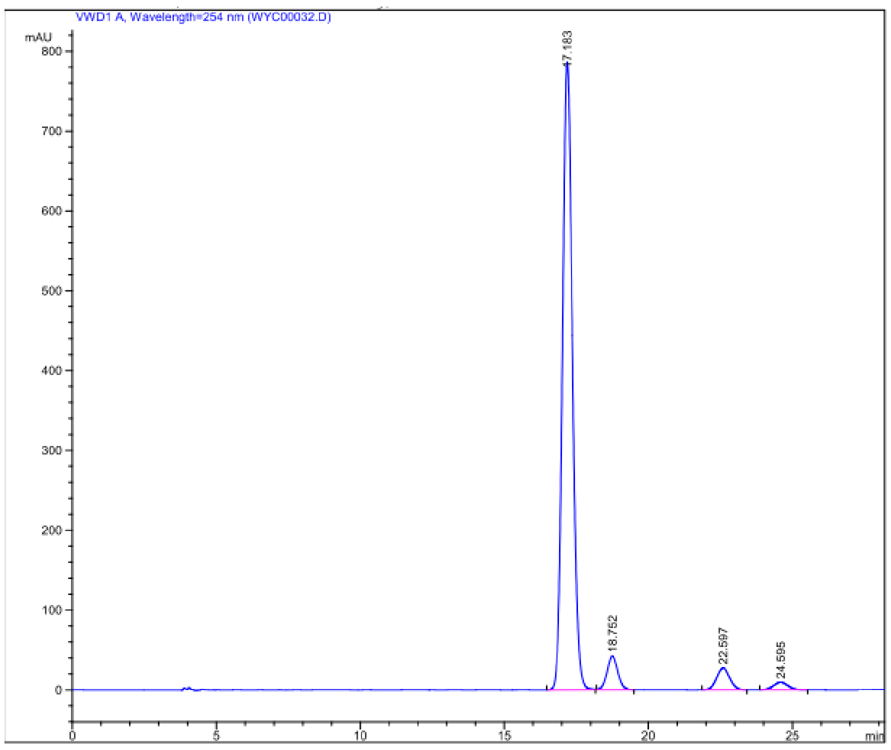

Peak #	RetTime [min]	Type	Width [min]	Area mAU *s	Height [mAU]	Area %
1	17.183	BB	0.3721	1.88079e4	786.70093	89.1755
2	18.752	BB	0.3978	1089.55042	42.34015	5.1660
3	22.597	BB	0.4891	859.43414	27.44145	4.0749
4	24.595	BB	0.5383	333.99329	9.50082	1.5836
Totals :				2.10909e4	865.98334	

Supplementary Figure 63. HPLC spectra for **12b**.

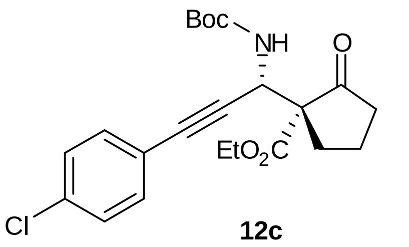

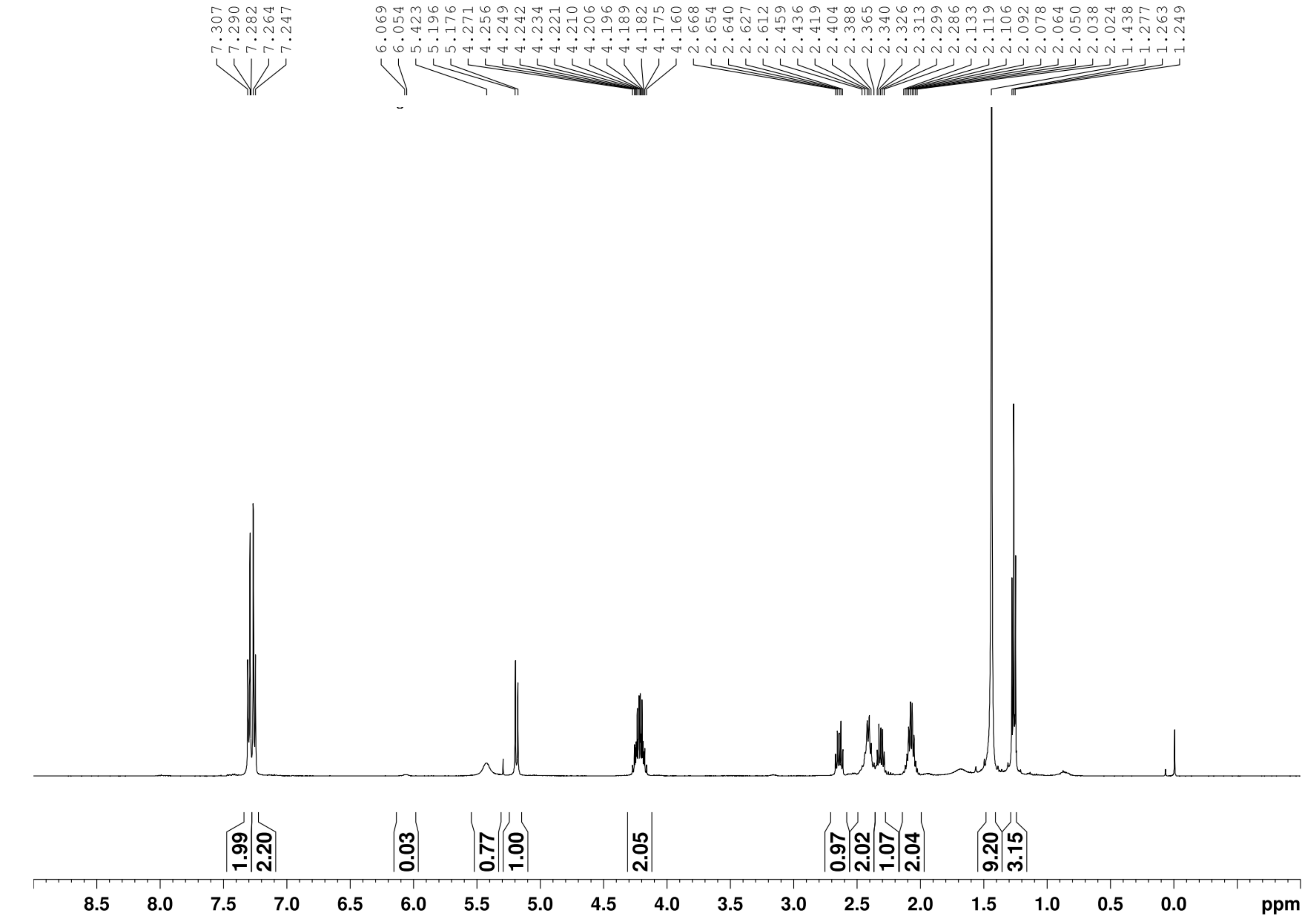

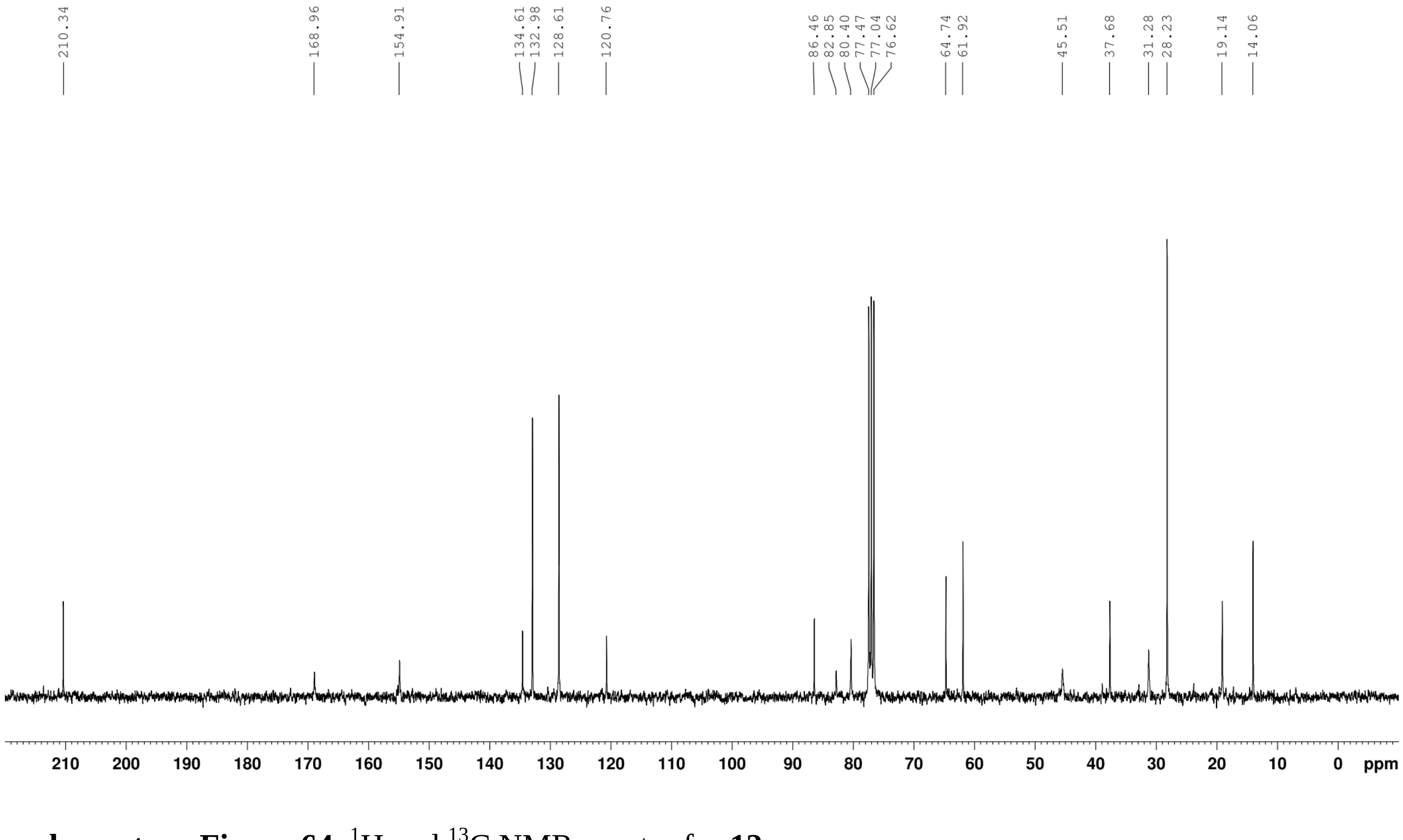

Supplementary Figure 64. ^{1}H and ^{13}C NMR spectra for **12c**.

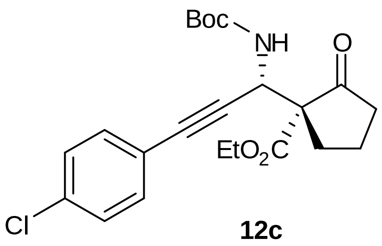

12c

HPLC analysis of **12c**: Daicel CHIRALPAK AD-H, *n*-hexane/*i*-PrOH = 98/2, flow rate = 0.7 mL/min, λ = 254 nm

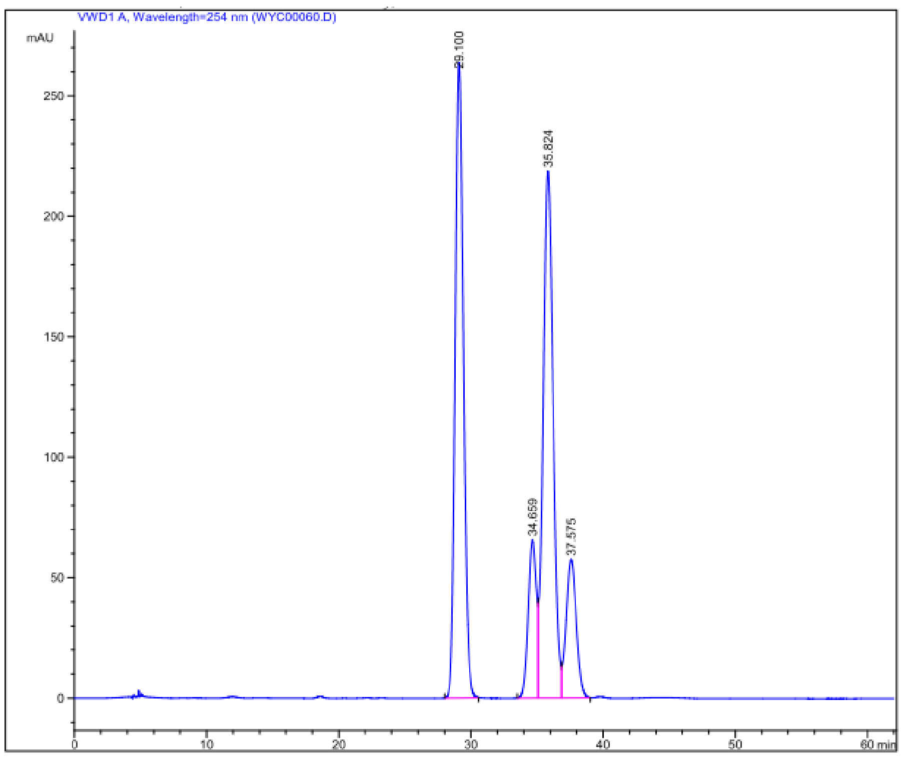

Peak #	RetTime [min]	Type	Width [min]	Area mAU *s	Height [mAU]	Area %
1	29.100	BB	0.6702	1.13689e4	263.80051	39.3181
2	34.659	BV	0.6738	2887.24341	65.76009	9.9852
3	35.824	VV	0.8154	1.15509e4	218.79388	39.9477
4	37.575	VB	0.8382	3108.05957	57.42019	10.7489

Totals : 2.89151e4 605.77466

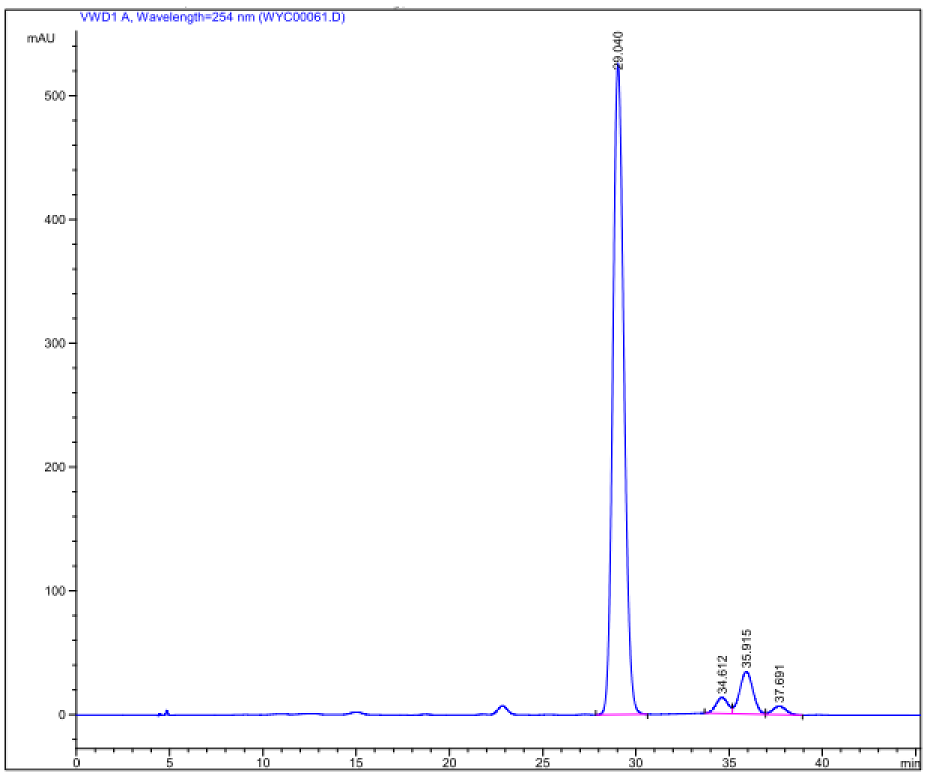

Peak #	RetTime [min]	Type	Width [min]	Area mAU *s	Height [mAU]	Area %
1	29.040	VB	0.6449	2.17201e4	525.92993	89.2206
2	34.612	BV	0.7032	584.08710	12.96578	2.3993
3	35.915	VV	0.7751	1703.33533	34.26451	6.9969
4	37.691	VP	0.7654	336.74741	6.78457	1.3833

Supplementary Figure 65. HPLC spectra for **12c**.

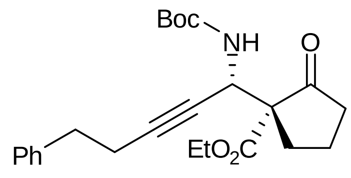

12d

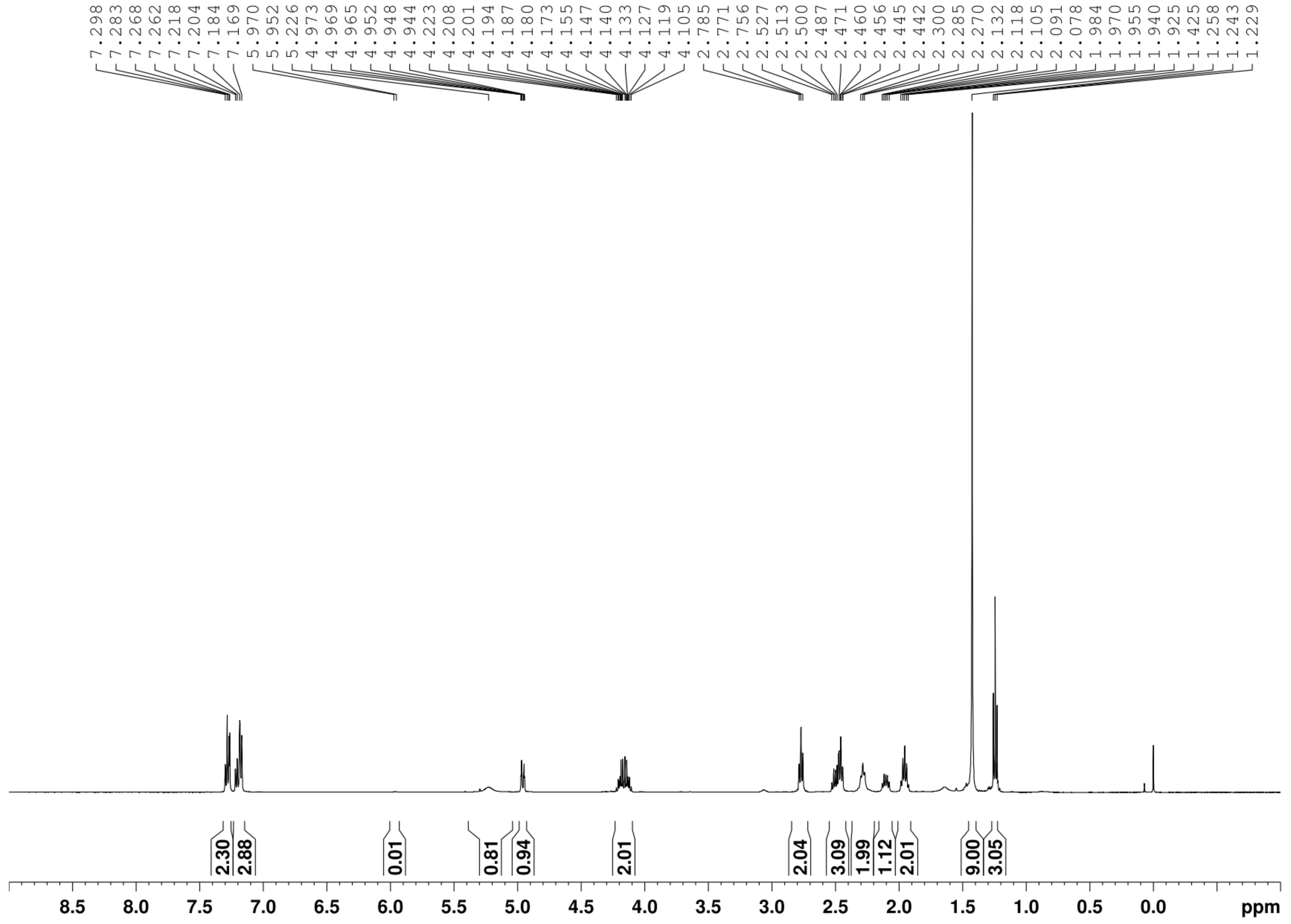

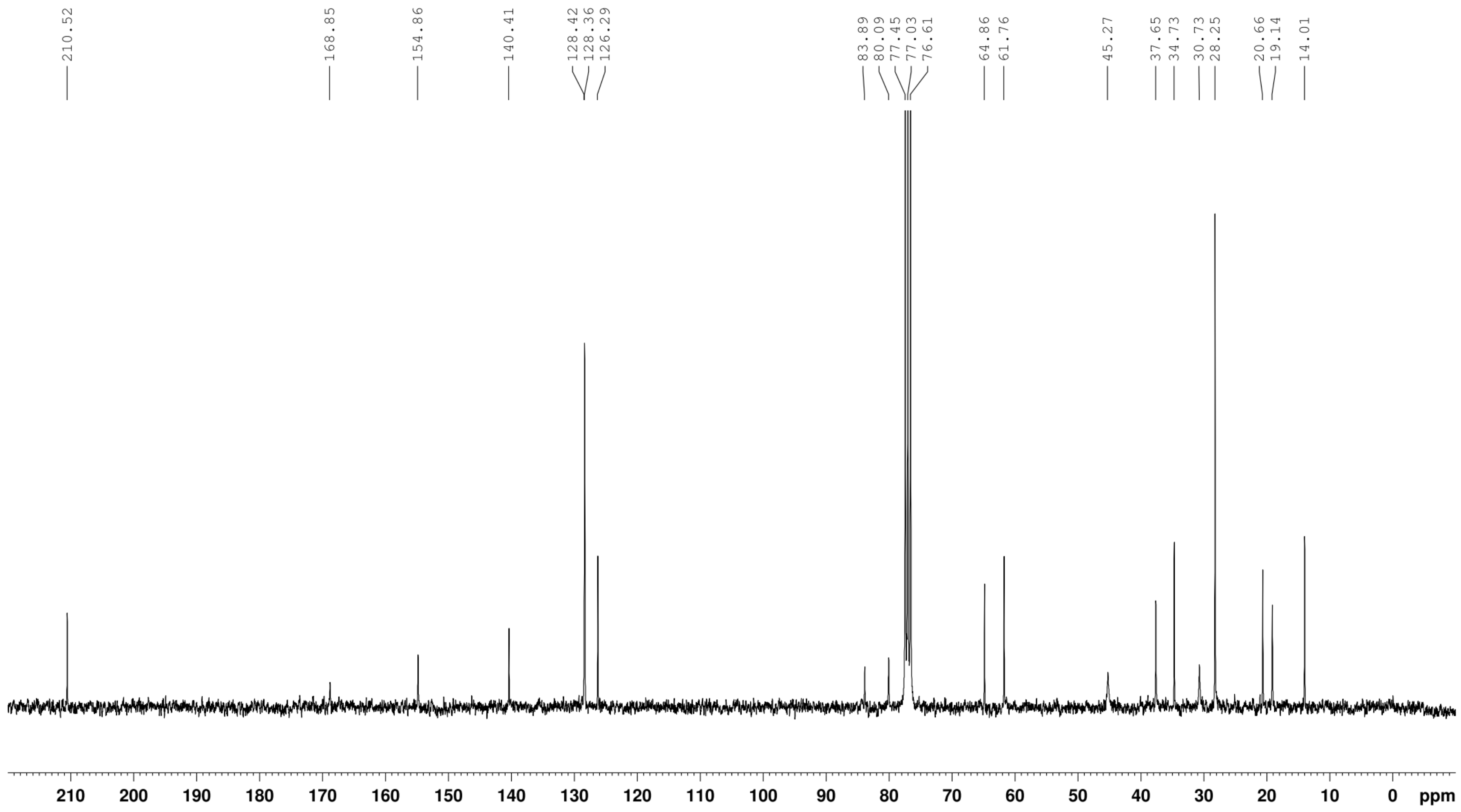

Supplementary Figure 66. ^{1}H and ^{13}C NMR spectra for **12d**.

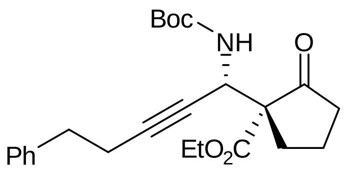

12d

HPLC analysis of **12d**: Daicel CHIRALPAK AD-H, *n*-hexane/*i*-PrOH = 98/2, flow rate = 0.7 mL/min, λ = 254 nm

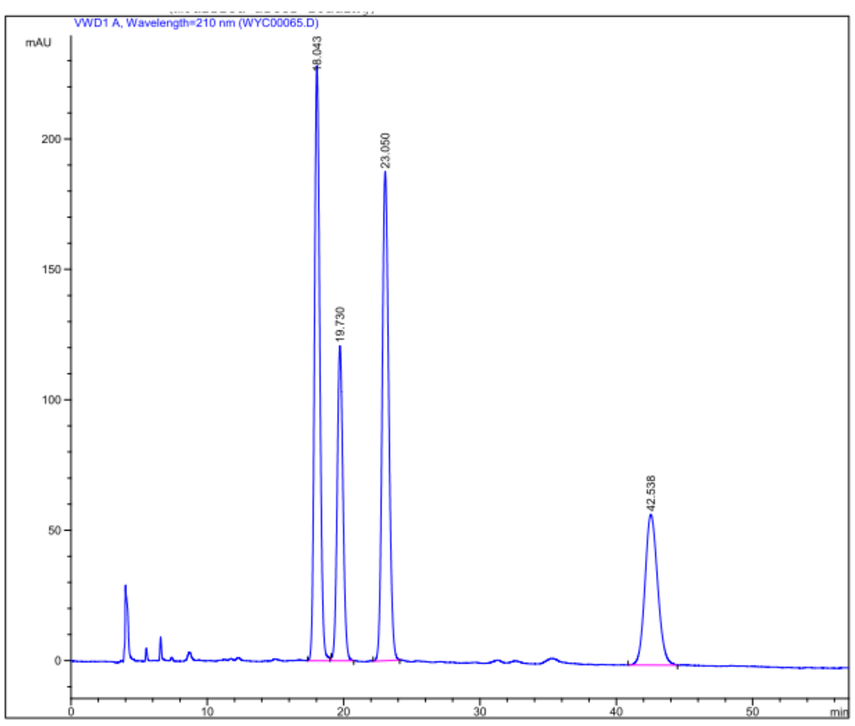

Peak #	RetTime [min]	Type	Width [min]	Area mAU *s	Height [mAU]	Area %
1	18.043	PB	0.4215	6190.63281	228.06282	31.4444
2	19.730	BB	0.4553	3551.35693	120.72469	18.0386
3	23.050	BB	0.5138	6219.85449	187.43349	31.5928
4	42.538	BB	0.9841	3725.73242	57.75306	18.9243
Totals :				1.96876e4	593.97406	

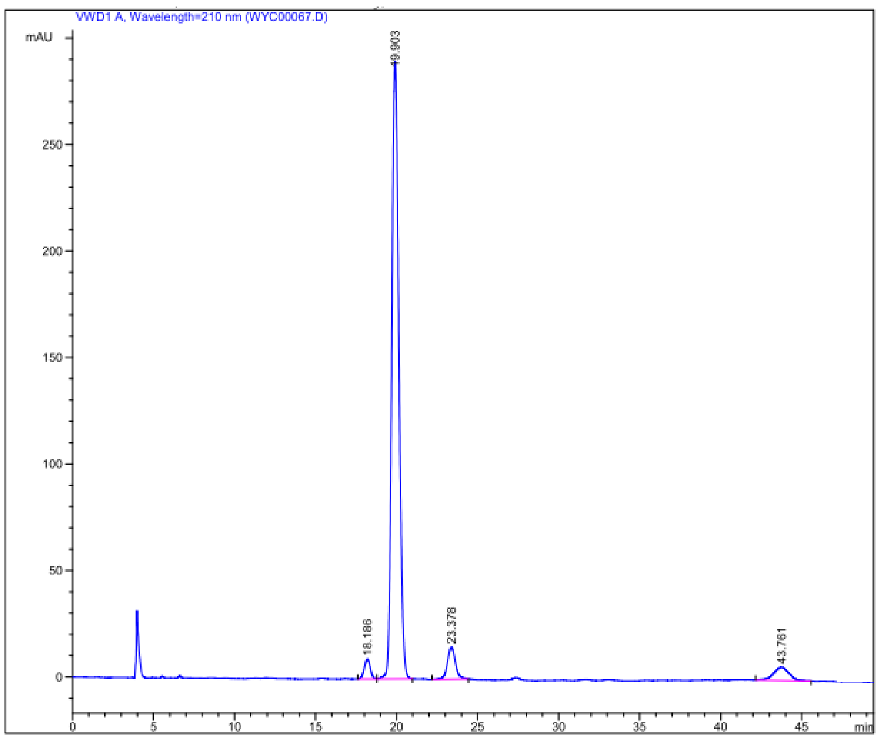

Peak #	RetTime [min]	Type	Width [min]	Area mAU *s	Height [mAU]	Area %
1	18.186	BV	0.4060	242.12903	9.33422	2.4210
2	19.903	VB	0.4758	8824.13477	290.02438	88.2297
3	23.378	PB	0.5170	518.95905	15.06032	5.1889
4	43.761	BB	0.7966	416.09628	6.24203	4.1604
Totals :				1.00013e4	320.66095	

Supplementary Figure 67. HPLC spectra for **12d**.

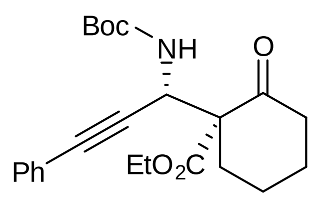

12e

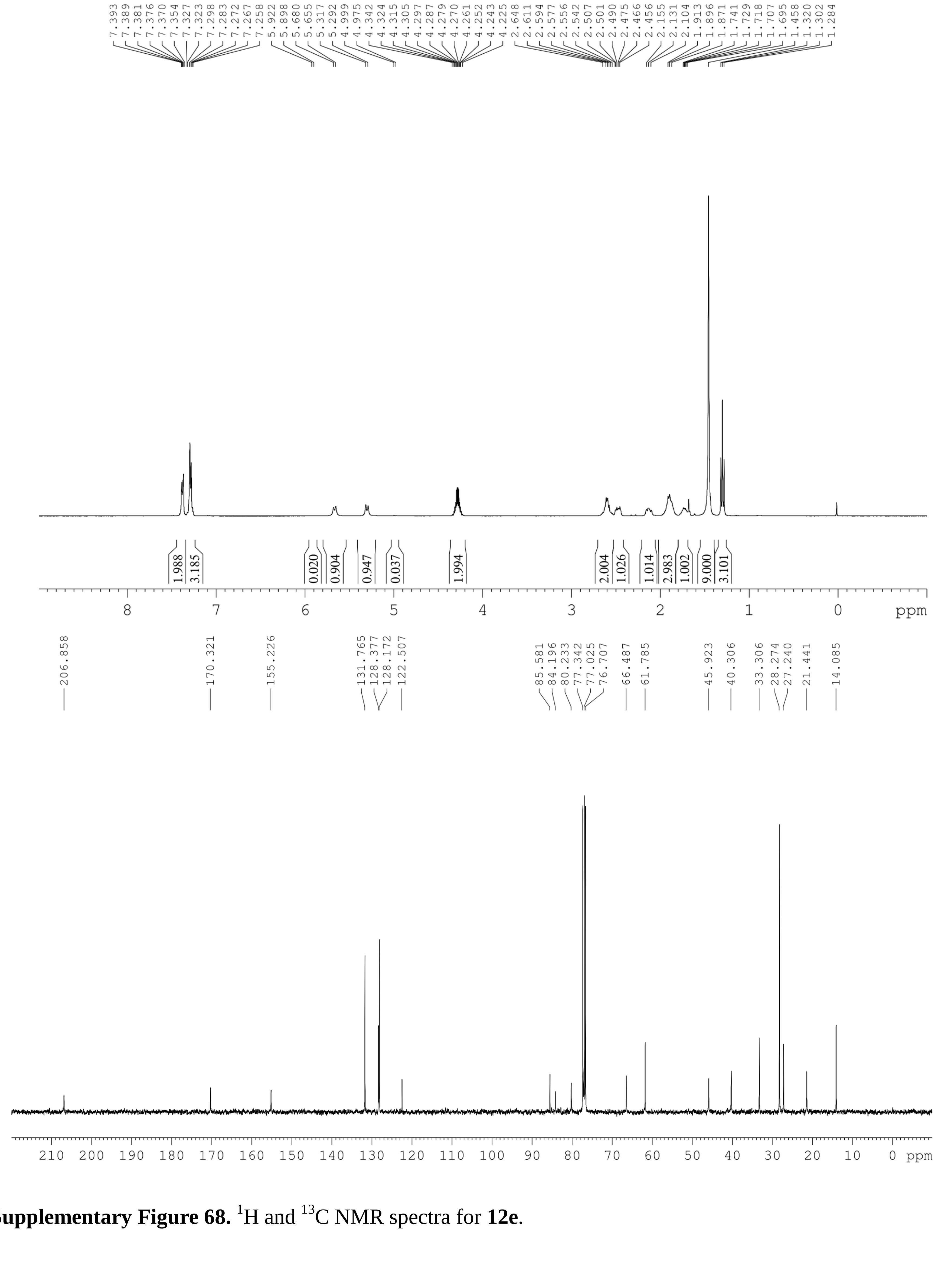

Supplementary Figure 68. ^{1}H and ^{13}C NMR spectra for **12e**.

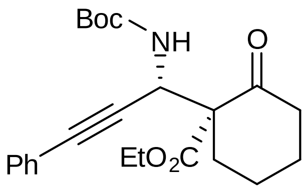

12e

HPLC analysis of **12e**: Daicel CHIRALPAK AD-H, *n*-hexane/*i*-PrOH = 98/2, flow rate = 0.8 mL/min, λ = 254 nm

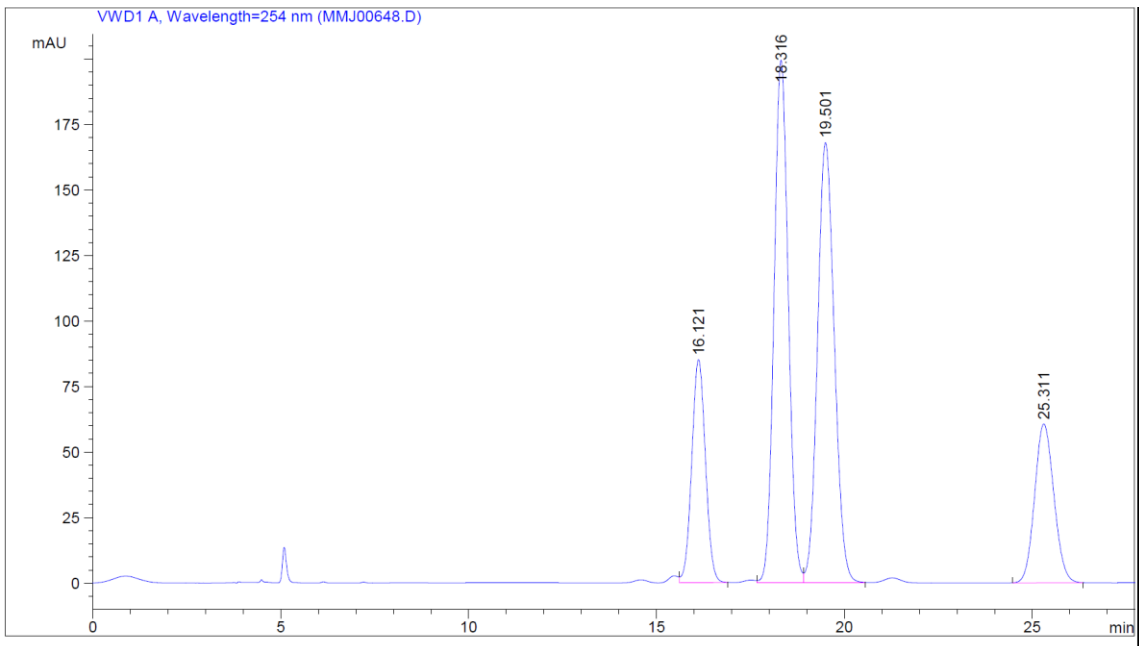

Peak #	RetTime [min]	Type	Width [min]	Area mAU *s	Height [mAU]	Area %
1	16.121	VB	0.3963	2158.44873	85.11113	14.5843
2	18.316	VV	0.4106	5249.28809	199.32089	35.4685
3	19.501	VB	0.4869	5226.65869	167.90503	35.3156
4	25.311	BB	0.5578	2165.45801	60.64437	14.6316

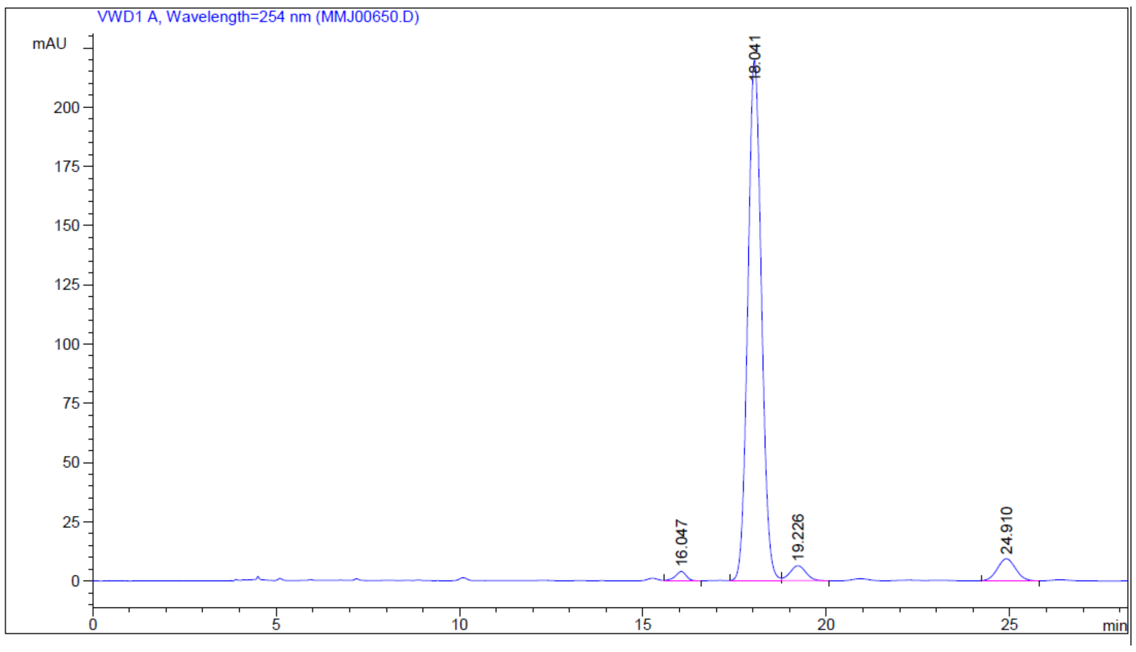

Peak #	RetTime [min]	Type	Width [min]	Area mAU *s	Height [mAU]	Area %
1	16.047	VP	0.3100	80.43448	3.90830	1.2844
2	18.041	BV	0.4031	5668.65527	219.60953	90.5180
3	19.226	VB	0.4668	191.80826	6.30843	3.0628
4	24.910	BB	0.5389	321.56705	9.29703	5.1348

Supplementary Figure 69. HPLC spectra for **12e**.

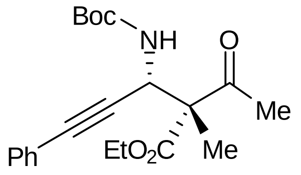

12f

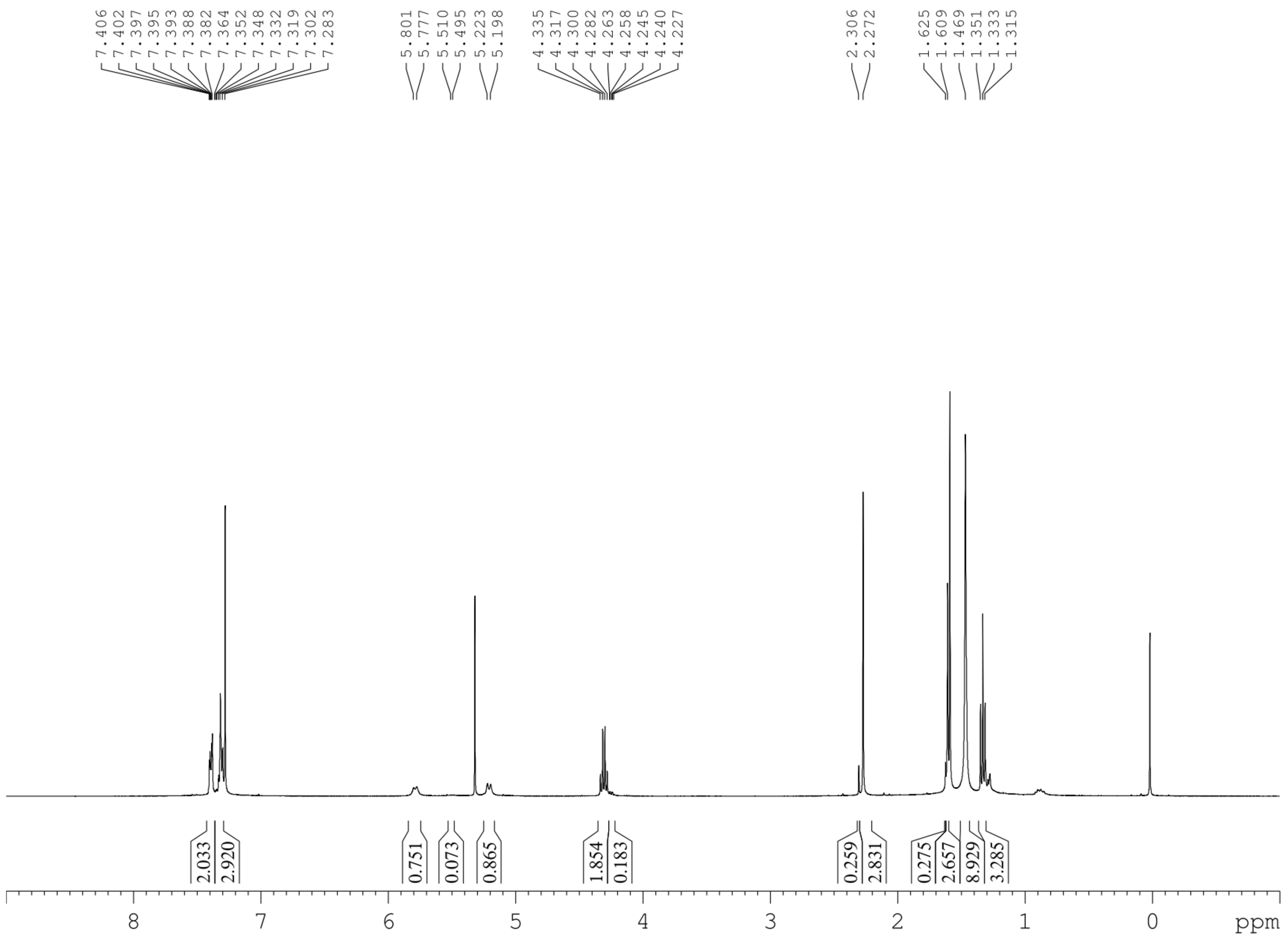

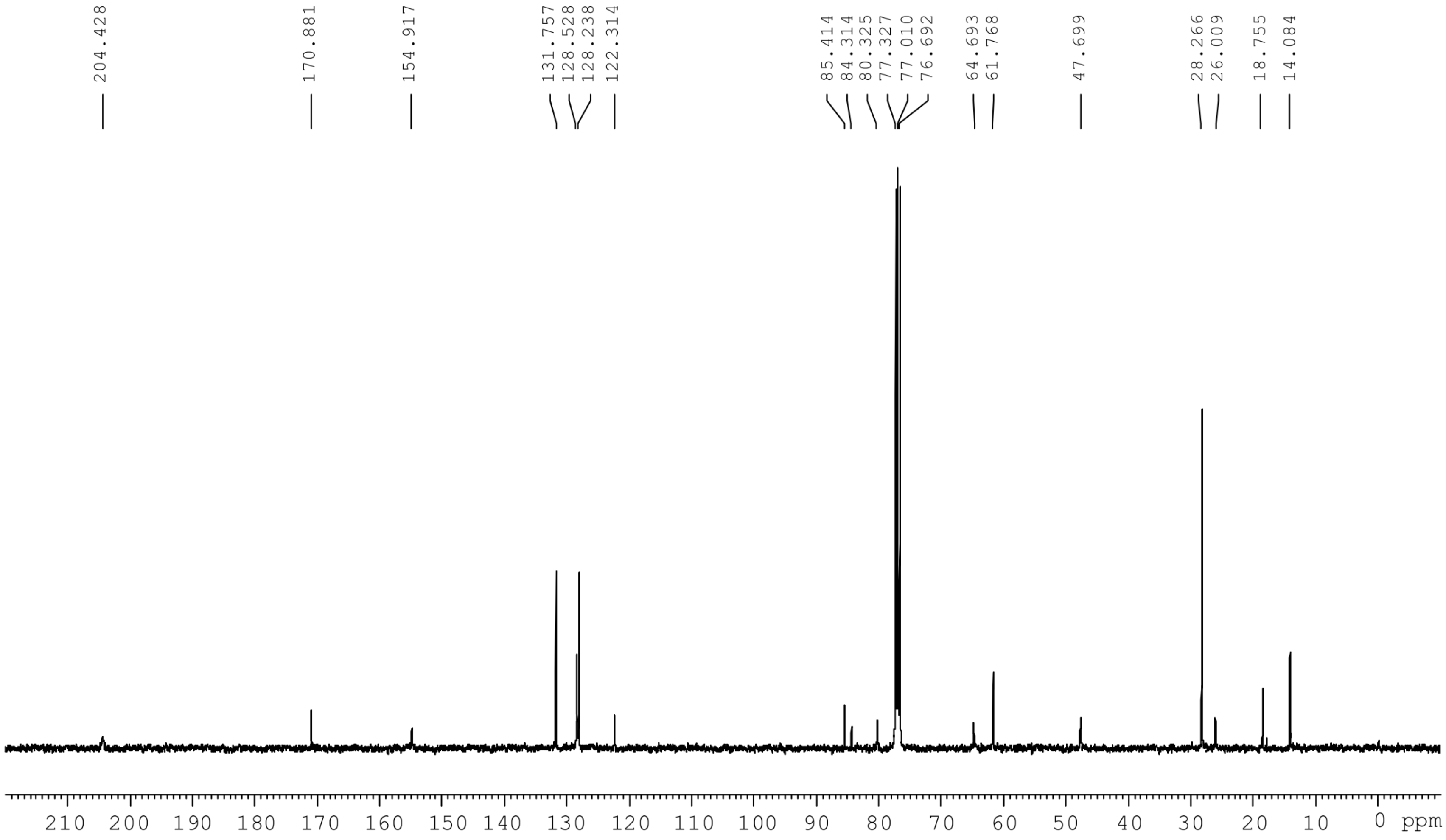

Supplementary Figure 70. ^{1}H and ^{13}C NMR spectra for **12f**.

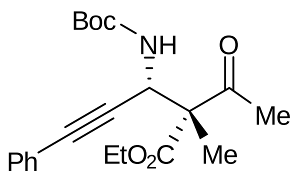

12f

HPLC analysis of **12f**: Daicel CHIRALPAK AD-H, *n*-hexane/*i*-PrOH = 97/3, flow rate = 0.8 mL/min, λ = 210nm

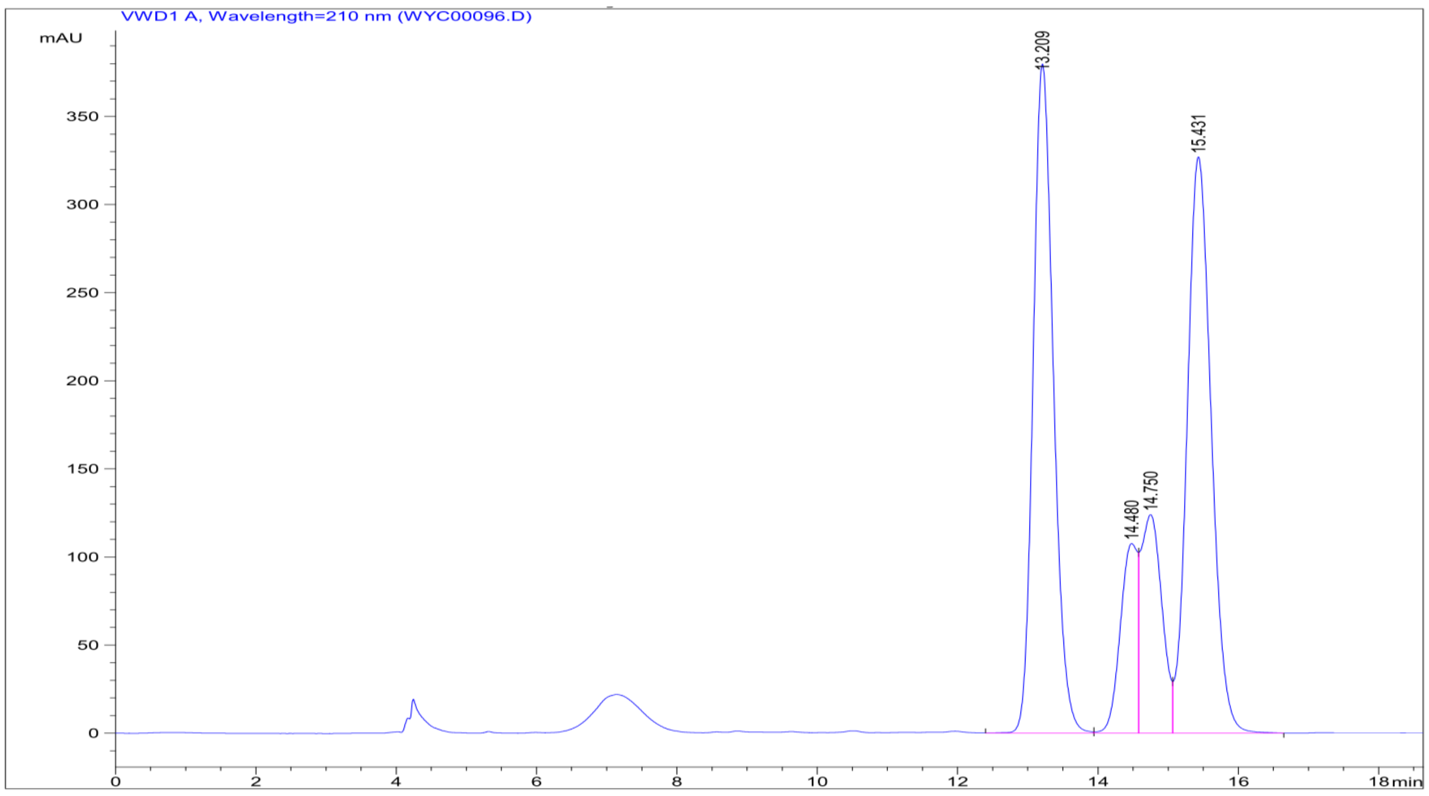

Peak #	RetTime [min]	Type	Width [min]	Area mAU *s	Height [mAU]	Area %
1	13.209	VV	0.3112	7617.26709	379.74832	38.4022
2	14.480	VV	0.2601	1822.56934	107.64051	9.1884
3	14.750	VV	0.3141	2596.99316	124.04043	13.0927
4	15.431	VB	0.3699	7798.65674	327.02087	39.3167
Totals :				1.98355e4	938.45013	

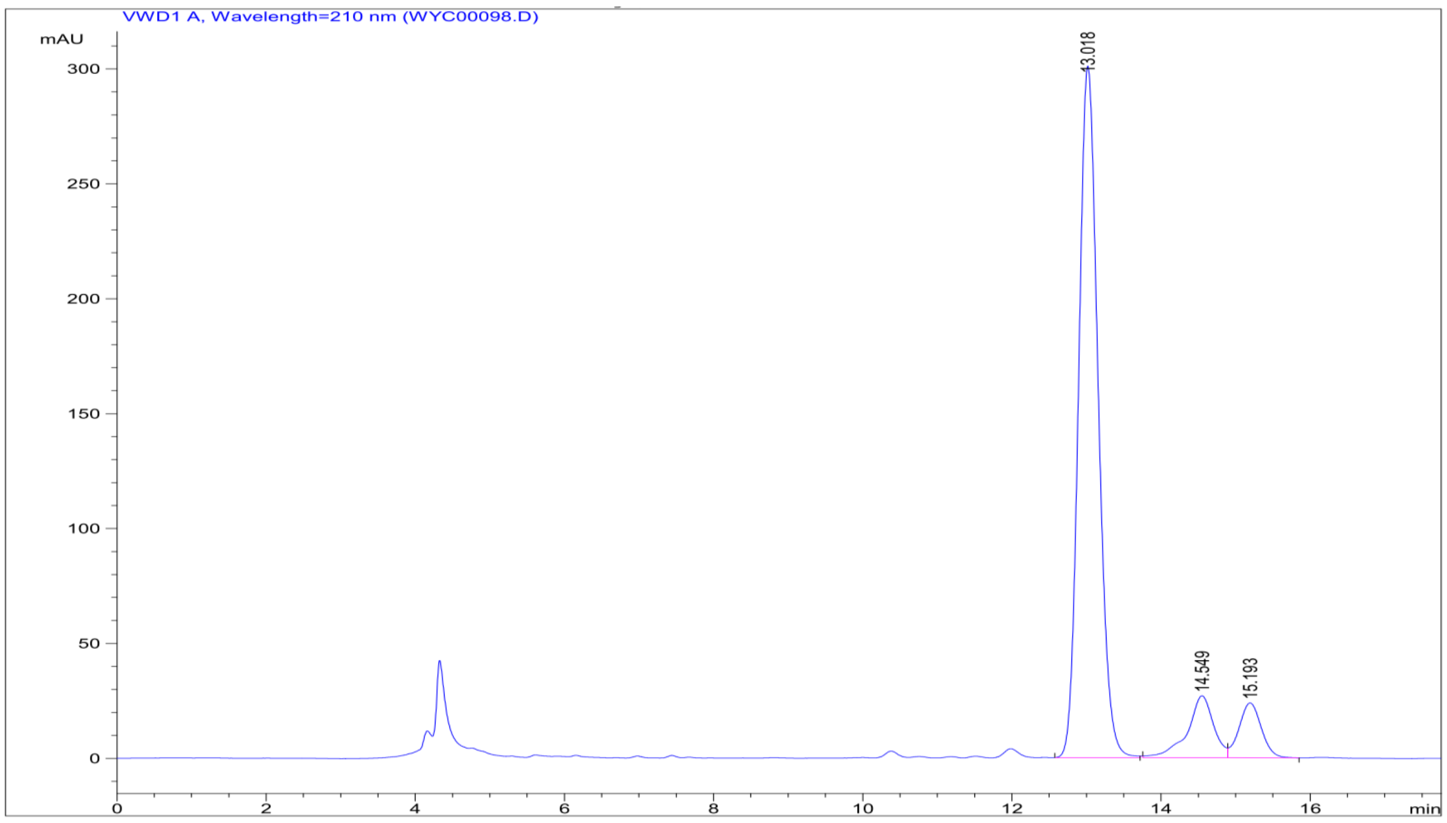

Peak #	RetTime [min]	Type	Width [min]	Area mAU *s	Height [mAU]	Area %
1	13.018	PB	0.2811	5446.70947	300.91214	82.5243
2	14.549	BV	0.3533	658.31238	26.96414	9.9742
3	15.193	VB	0.3206	495.10895	23.86945	7.5015
Totals :				6600.13080	351.74573	

Supplementary Figure 71. HPLC spectra for **12f**.

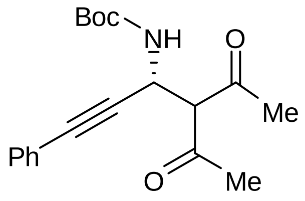

13

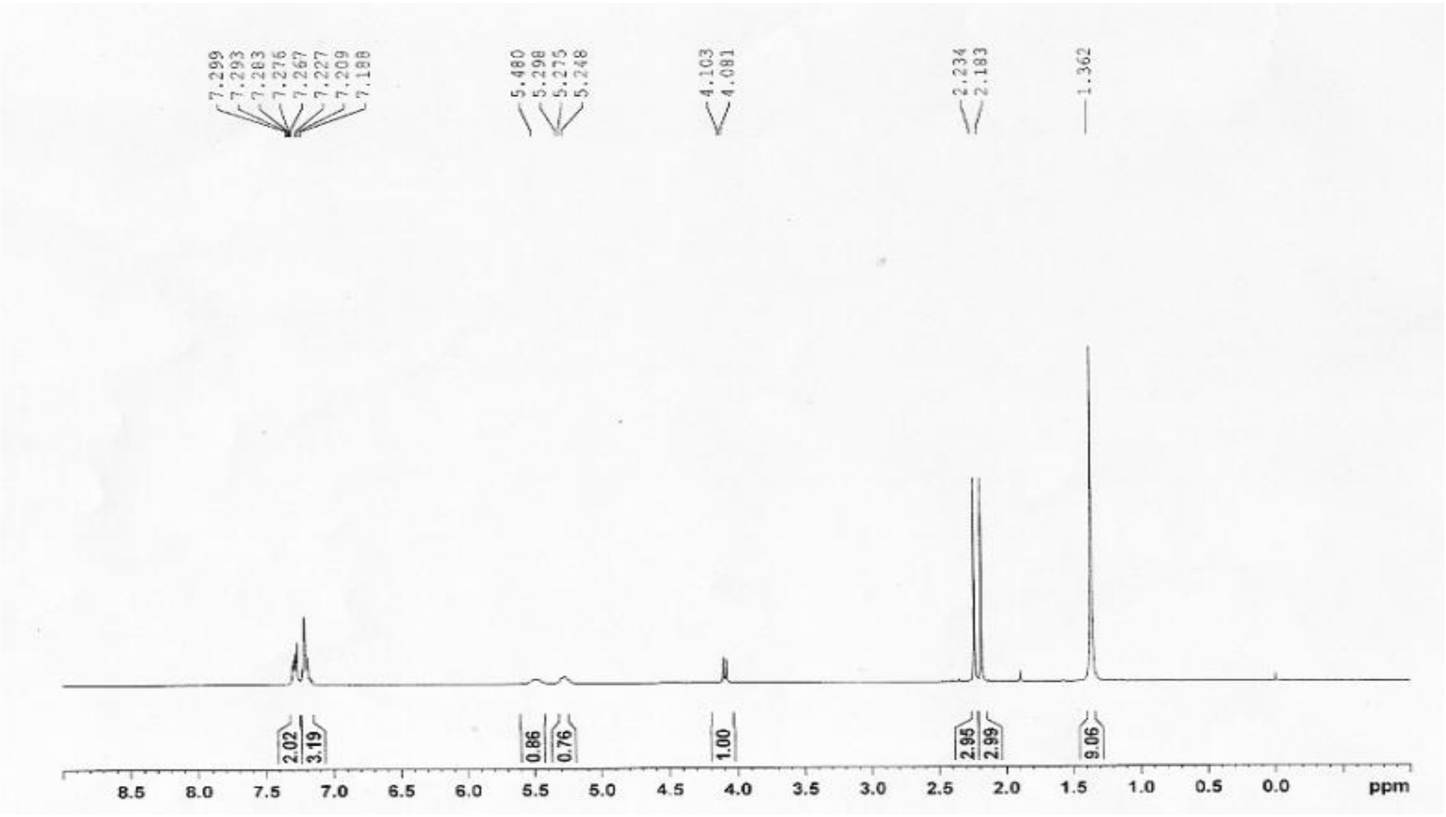

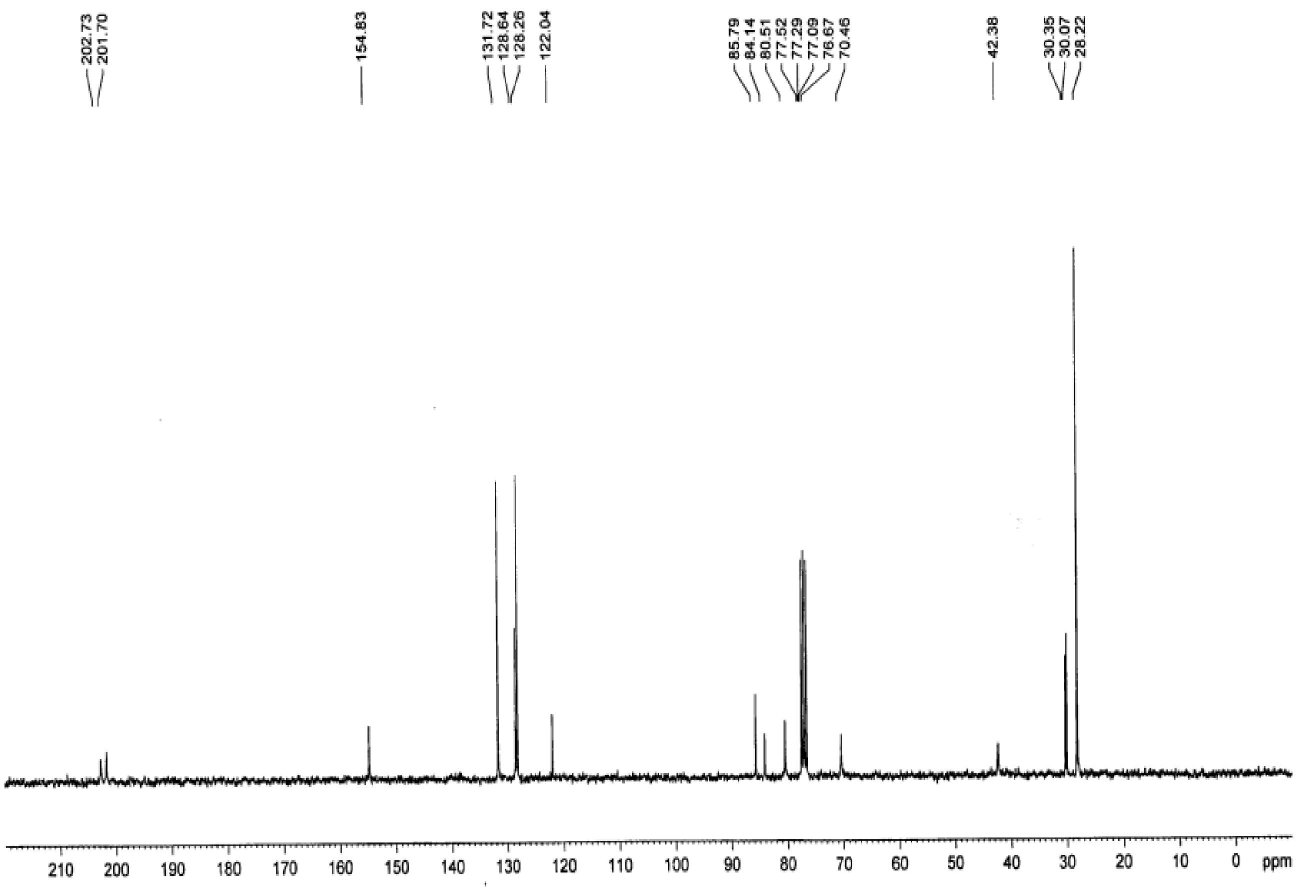

Supplementary Figure 72. ^{1}H and ^{13}C NMR spectra for **13**.

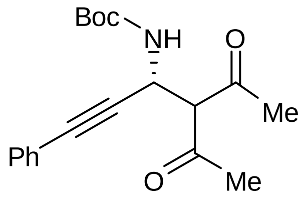

13

HPLC analysis of **13**: Daicel CHIRALPAK AD-H, *n*-hexane/*i*-PrOH = 98/2, flow rate = 1.0 mL/min, λ = 210nm;

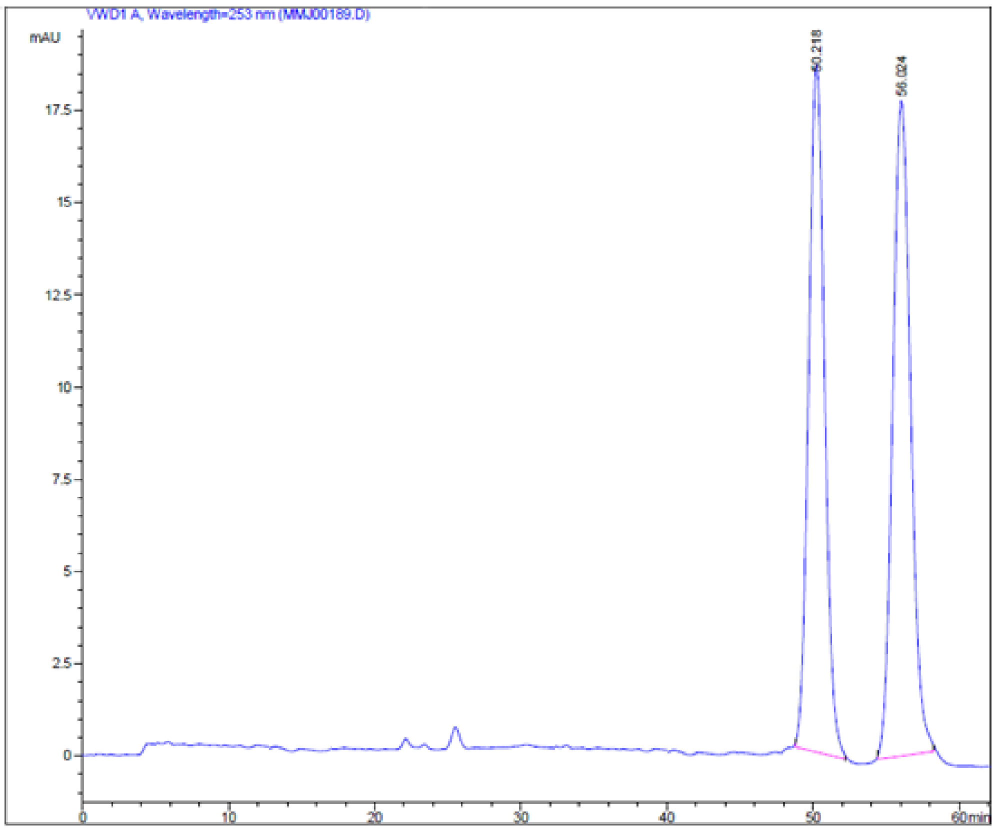

Peak #	RetTime [min]	Type	Width [min]	Area mAU *s	Height [mAU]	Area %
1	50.218	BB	1.1447	1407.96057	18.64245	48.2013
2	56.024	BB	1.2773	1513.03772	17.76934	51.7987
Totals :				2920.99829	36.41179	

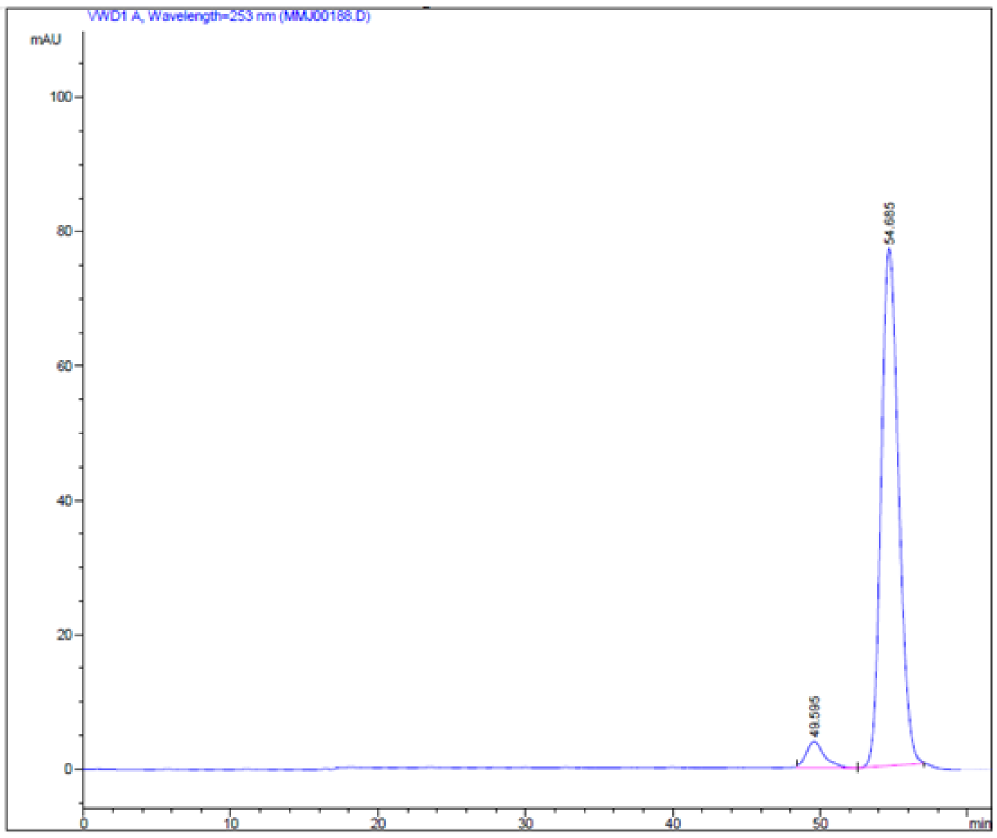

Peak #	RetTime [min]	Type	Width [min]	Area mAU *s	Height [mAU]	Area %
1	49.595	VP	1.1219	331.82639	3.88989	4.9592
2	54.685	VB	1.2332	6359.27930	77.09296	95.0408
Totals :				6691.10568	80.98285	

Supplementary Figure 73. HPLC spectra for **13**.

Supplementary Methods

Characterization of C-alkynyl *N*-Boc-*N,O*-acetals (1a-m)

1 *t*-butyl (1-ethoxy-3-phenylprop-2-yn-1-yl)carbamate **(1a)**

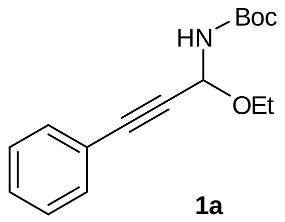

1a

Was obtained as white solid (2.42 g, 8.8 mmol, 88%) after flash chromatography (elution gradient: Ethyl Acetate/Hexane= 1/20);

^{1}**H-NMR** (500 MHz, $CDCl_3$), δ (ppm): 7.46-7.44 (m, 2H), 7.35-7.30 (m, 3H), 5.85-5.84 (d, 1H, *J* = 5.0 Hz), 5.46 (s, 1H), 3.72-3.63 (m, 2H), 1.48 (s, 9H), 1.27 (t, 3H, *J* = 7.0 Hz);

^{13}**C-NMR** (75 MHz, $CDCl_3$), δ (ppm): 154.4, 131.8, 128.8, 128.3, 121.8, 85.0, 84.5, 80.5, 72.2, 62.7, 28.3, 15.0;

HRMS calcd. for $C_{16}H_{21}NO_3Na^+$ $[M+Na]^+$: 298.1413, found: 298.1411

2 *t*-butyl (1-ethoxy-3-(*o*-tolyl)prop-2-yn-1-yl)carbamate **(1b)**

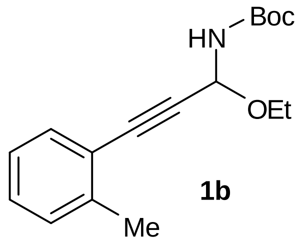

1b

Was obtained as pale yellow solid (2.46 g, 85%) after flash chromatography (elution gradient: Ethyl Acetate/Hexane= 1/20);

^{1}**H-NMR** (500 MHz, $CDCl_3$), δ (ppm): 7.40 (d, 1H, *J* = 7.5 Hz), 7.26-7.22 (m, 1H), 7.18 (d, 1H, *J* = 7.5 Hz), 7.14-7.11 (m, 1H), 5.88 (d, 1H, *J* = 9.0 Hz), 5,48(m, 1H), 3.74-3.68 (m, 2H), 2.42 (s, 3H), 1.48 (s, 9H), 1.27 (t, 3H, *J* = 7.0 Hz);

^{13}**C-NMR** (75 MHz, $CDCl_3$), δ (ppm): 154.4, 140.2, 132.1, 131.7, 129.3, 128.7, 128.2, 125.4, 121.6, 89.0, 83.3, 80.1, 72.3, 62.3, 28.4, 20.4, 14.97;

HRMS calcd. for $C_{17}H_{23}NO_3K^+$ $[M+K]^+$: 328.1309, found: 328.1313

3 *t*-butyl (1-ethoxy-3-(*m*-tolyl)prop-2-yn-1-yl)carbamate **(1c)**

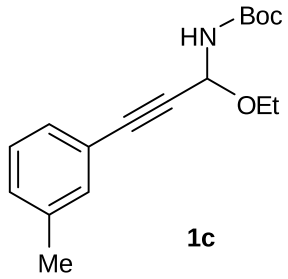

1c

Was obtained as pale yellow solid (2.40 g, 83%) after flash chromatography (elution gradient: Ethyl Acetate/Hexane= 1/20);

^{1}H-NMR (500 MHz, $CDCl_3$), δ (ppm): 7.27-7.24 (m, 2H), 7.20-7.18 (m, 1H), 7,15-7.13(m,1H), 5.84 (d, 1H, *J* = 9.0 Hz), 5.49(m, 1H), 3.72-3.65 (m, 2H), 2.32 (s, 3H), 1.48 (s, 9H),1.26 (t, 3H, *J* = 7.0 Hz);

^{13}C-NMR (75 MHz, $CDCl_3$), δ (ppm): 154.4, 137.9, 132.4, 129.7, 128.9, 128.2, 121.6, 84.7, 80.4, 72.2, 62.5, 28.2, 21.1, 15.0;

HRMS calcd. for $C_{17}H_{23}NO_3K^+$ $[M+K]^+$: 328.1309, found: 328.1313

4 *t*-butyl (1-ethoxy-3-(*p*-tolyl)prop-2-yn-1-yl)carbamate **(1d)**

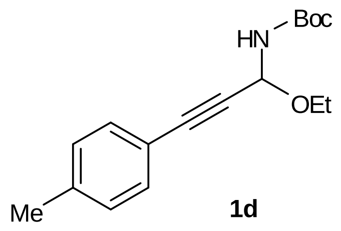

1d

Was obtained as pale yellow solid (2.40 g, 83%) after flash chromatography (elution gradient: Ethyl Acetate/Hexane= 1/20);

^{1}H-NMR (500 MHz, $CDCl_3$), δ (ppm): 7.33 (d, 2H, *J* = 8.0 Hz), 7.10 (d, 2H, *J* = 8.0 Hz), 5.84 (d, 1H, *J* = 9.0 Hz), 5.48 (s, 1H), 3.72-3.64 (m, 2H), 2.34 (s, 3H), 1.47 (s, 9H), 1.26 (t, 3H, *J* = 7.0 Hz);

^{13}C-NMR (75 MHz, $CDCl_3$), δ (ppm): 154.4, 139.0, 131.7, 129.0, 118.7, 84.6, 84.3, 80.5, 72.2, 28.3, 21.4, 15.04;

HRMS calcd. for $C_{17}H_{23}NO_3K^+$ $[M+K]^+$: 328.1309, found: 328.1313

5 *t*-butyl (1-ethoxy-3-(4-methoxyphenyl)prop-2-yn-1-yl)carbamate **(1e)**

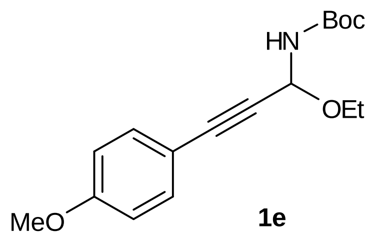

1e

Was obtained as pale yellow solid (2.68 g, 88%) after flash chromatography (elution gradient: Ethyl Acetate/Hexane= 1/8);

^{1}H-NMR (500 MHz, $CDCl_3$), δ (ppm): 7.39 (d, 2H, *J* = 8.0 Hz), 6.84 (d, 2H, *J* = 8.0 Hz), 5.83 (d, 1H, *J* = 9.0 Hz), 5.45 (d, 1H, *J* = 6.0 Hz), 3.80 (s, 2H), 3.71-3.64 (m, 2H), 1.47 (s, 9H), 1.26 (t, 3H, *J* = 7.0 Hz);

^{13}C-NMR (75 MHz, $CDCl_3$), δ (ppm): 160.0, 154.4, 133.3, 113.9, 113.8, 84.5, 83.7, 80.4, 72.3, 62.6, 55.2, 28.3, 15.0;

HRMS calcd. for $C_{17}H_{23}NO_4Na^+$ $[M+Na]^+$: 328.1519, found: 328.1517

6 *t*-butyl (3-(4-bromophenyl)-1-ethoxyprop-2-yn-1-yl)carbamate **(1f)**

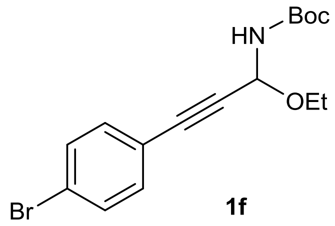

1f

Was obtained as pale yellow solid (2.90 g, 82%) after flash chromatography (elution gradient: Ethyl

Acetate/Hexane= 1/10);

^{1}H-NMR (500 MHz, $CDCl_3$), δ (ppm): 7.45 (d, 2H, *J* = 8.0 Hz), 7.31 (d, 2H, *J* = 8.0 Hz), 5.83 (d, 1H, *J* = 9.0 Hz), 5.44 (d, 1H, *J* = 6.0Hz), 3.72-3.63 (m, 2H), 1.47 (s, 9H), 1.26 (t, 3H, *J* = 7.0 Hz);

^{13}C-NMR (75 MHz, $CDCl_3$), δ (ppm): 154.3, 133.2, 131.6, 123.2, 120.7, 86.2, 83.3, 80.6, 72.1, 62.8, 28.3, 15.0;

HRMS calcd. for $C_{16}H_{20}NO_3BrNa^+$ $[M+Na]^+$: 376.0518, found 376.0517

7 *t*-butyl (3-(4-chlorophenyl)-1-ethoxyprop-2-yn-1-yl)carbamate **(1g)**

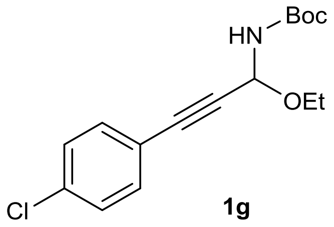

1g

Was obtained as pale yellow solid (2.47 g, 80%) after flash chromatography (elution gradient: Ethyl Acetate/Hexane= 1/10);

^{1}H-NMR (500 MHz, $CDCl_3$), δ (ppm): 7.38 (d, 2H, *J* = 8.0 Hz), 7.29 (d, 2H, *J* = 8.0 Hz), 5.84 (d, 1H, *J* = 9.0 Hz), 5.45 (d, 1H, *J* = 6.0Hz), 3.72-3.62 (m, 2H), 1.48 (s, 9H), 1.27 (t, 3H, *J* = 7.0 Hz);

^{13}C-NMR (75 MHz, $CDCl_3$), δ (ppm): 154.3, 135.0, 133.1, 128.7, 120.2, 85.9, 83.3, 80.7, 72.1, 62.9, 28.3, 15.0;

HRMS calcd. for $C_{16}H_{20}NO_3ClNa^+$ $[M+Na]^+$: 332.1023, found: 332.1026

8 *t*-butyl (1-ethoxy-3-(4-(trifluoromethyl)phenyl)prop-2-yn-1-yl)carbamate **(1h)**

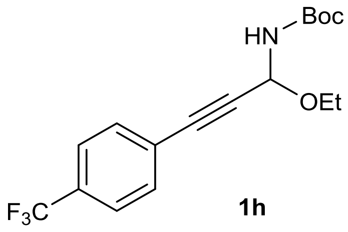

1h

Was obtained as pale yellow solid (1.44 g, 84%) after flash chromatography (elution gradient: Ethyl Acetate/Hexane= 1/15);

^{1}H-NMR (500 MHz, $CDCl_3$), δ (ppm): 7.59-7.55 (m, 4H), 5.87 (d, 1H, *J* = 9.0 Hz), 5.44 (s, 1H), 3.73-3.63 (m, 2H), 1.48 (s, 9H), 1.27 (t, 3H, *J* = 7.0 Hz);

^{13}C-NMR (75 MHz, $CDCl_3$), δ (ppm): 154.3, 132.1, 130.8, 130.4, 125.6, 125.2, 125.2, 122.0, 87.4, 82.9, 80.7, 72.1, 62.9, 28.2, 15.0;

HRMS calcd. for $C_{17}H_{20}NO_3F_3Na^+$ $[M+Na]^+$: 366.1287, found: 366.1284

9 *t*-butyl (1-ethoxy-3-(thiophen-2-yl)prop-2-yn-1-yl)carbamate **(1i)**

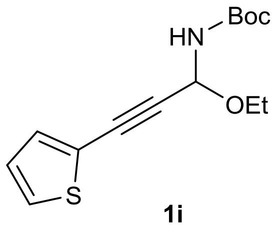

1i

Was obtained as pale yellow solid (2.47, 88%) after flash chromatography (elution gradient: Ethyl Acetate/Hexane= 1/15);

^{1}H-NMR (300 MHz, $CDCl_3$), δ (ppm): 7.28-7.23 (m, 2H), 6.98-6.95 (m, 1H), 5.86 (d, 1H, *J* = 9.3 Hz), 5.58 (d, 1H, *J* = 7.8 Hz), 3.71-3.62 (m, 2H), 1.48 (s, 9H), 1.23 (t, 3H, *J* = 7.0 Hz);

^{13}C-NMR (75 MHz, $CDCl_3$), δ (ppm): 154.3, 132.9, 127.8, 126.9, 121.6, 88.7, 80.6, 77.9, 72.3, 62.8, 28.3, 15.0;

HRMS calcd. for $C_{14}H_{19}NO_3SNa^+$ $[M+Na]^+$: 304.0977, found: 304.0979

10 (*E*)-*t*-butyl (1-ethoxy-5-phenylpent-4-en-2-yn-1-yl)carbamate **(1j)**

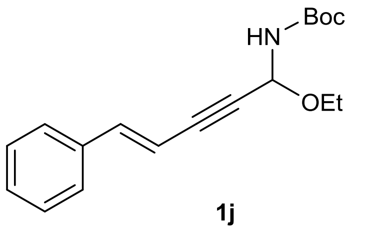

1j

Was obtained as yellow solid (2.53 g, 84%) after flash chromatography (elution gradient: Ethyl Acetate/Hexane= 1/10);

^{1}H-NMR (500 MHz, $CDCl_3$), δ (ppm): 7.38-7.30 (m, 5H), 6.99 (d, 1H, *J* = 16.5 Hz), 6.14 (dd, 1H, *J* = 1.5 Hz, *J* = 1 Hz), 5.80 (d, 1H, *J* = 9.0 Hz), 5.40 (d, 1H, *J* = 6.0Hz), 3.73-3.62 (m, 2H), 1.47 (s, 9H), 1.26 (t, 3H, *J* = 7.0 Hz);

^{13}C-NMR (75 MHz, $CDCl_3$), δ (ppm): 154.3, 142.3, 135.8, 129.0, 128.8, 126.4, 106.7, 86.9, 83.7, 80.6, 72.3, 62.7, 28.3, 15.1;

HRMS calcd. for $C_{18}H_{23}NO_3Na^+$ $[M+Na]^+$: 324.1570, found: 324.1570

11 (*E*)-*t*-butyl (5-(4-chlorophenyl)-1-ethoxypent-4-en-2-yn-1-yl)carbamate **(1k)**

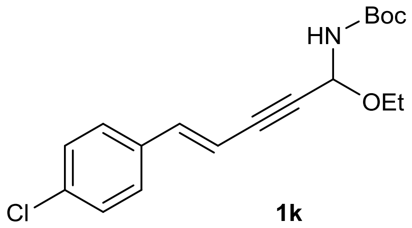

1k

Was obtained as yellow solid (1.34 g, 80%) after flash chromatography (elution gradient: Ethyl Acetate/Hexane= 1/10);

^{1}H-NMR (500 MHz, $CDCl_3$), δ (ppm): 7.29 (s, 4H), 6.96-6.93 (dd, 1H, *J* = 5.0 Hz, *J* = 5.0 Hz), 6.13-6.09 (dd, 1H, *J* = 1.0 Hz, *J* = 1.0 Hz), 5.78 (d, 1H, *J* = 9.0 Hz), 5.38 (d, 1H, *J* = 6.0Hz), 3.74-3.61 (m, 2H), 1.47 (s, 9H), 1.25 (t, 3H, *J* = 7 Hz);

^{13}C-NMR (75 MHz, $CDCl_3$), δ (ppm): 154.3, 141.5, 134.7, 134.3, 129.0, 127.5, 107.3, 87.5, 83.3, 80.6, 72.2, 62.8, 28.3, 15.0;

HRMS calcd. for $C_{18}H_{22}NO_3ClNa^+$ $[M+Na]^+$: 358.1180, found: 358.1178

12 *t*-butyl (1-ethoxy-5-phenylpent-2-yn-1-yl)carbamate **(1l)**

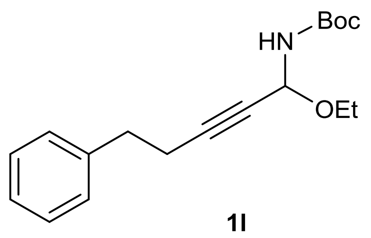

1l

Was obtained as white solid (2.18 g, 72%) after flash chromatography (elution gradient: Ethyl Acetate/Hexane= 1/20);

^{1}H-NMR (500 MHz, $CDCl_3$), δ (ppm): 7.30-7.28 (m, 2H), 7.23-7.19 (m, 3H), 5.57 (d, 1H, *J* = 7.0Hz), 5.29 (d, 1H, *J* = 10.5Hz), 3.74-3.52 (m, 2H), 2.83 (t, 2H, *J* = 7.5 Hz), 2.52-2.49 (m, 2H), 1.46 (s, 9H),1.26-1.19 (m, 3H);

^{13}C-NMR (75 MHz, $CDCl_3$), δ (ppm): 154.4, 140.3, 128.4, 126.4, 84.6, 80.2, 71.8, 62.3, 34.6, 28.3, 20.8, 15.0;

HRMS calcd. for $C_{18}H_{25}NO_3Na^+$ [M+Na$^+$]: 326.1726, found: 326.1727

13 *t*-butyl 1-ethoxyhept-2-ynylcarbamate **(1m)**

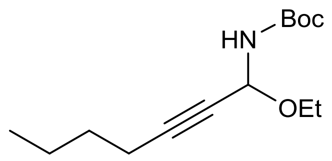

1m

Colourless solid (1.46 g, 85%) after flash chromatography (elution gradient: Ethyl Acetate/Hexane= 1/15);

^{1}H-NMR (300 MHz, $CDCl_3$), δ (ppm): 5.52 (d, 1H, *J* = 8.7 Hz), 5.34 (s, 1H), 3.56-3.47 (m, 2H), 2.16-2.11 (m, 2H), 1.45-1.31 (m, 4H), 1.39 (s, 9H), 1.17 (t, 3H, *J* = 6.9 Hz), 0.83 (t, 3H, *J* = 6.9 Hz);

^{13}C-NMR (75 MHz, $CDCl_3$), δ (ppm): 154.3, 85.5, 80.2, 76.2, 71.8, 62.3, 30.31, 28.2, 21.9, 18.2, 14.9, 13.5;

HRMS calcd. for $C_{14}H_{25}NO_3Na^+$ [M+ Na]$^+$: 278.1729, found: 278.1726

14 benzyl (1-ethoxy-3-phenylprop-2-yn-1-yl)carbamate **(1n)**

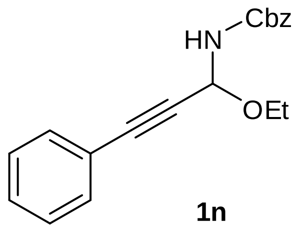

1n

Was obtained as pale yellow solid (2.66 g, 86%) after flash chromatography (elution gradient: Ethyl Acetate/Hexane= 1/15);

^{1}H-NMR (300 MHz, $CDCl_3$), δ (ppm): 7.36-7.34 (m, 2H), 7.33-7.16 (m, 8H), 5.82 (m, 1H), 5.75 (s, 1H), 5.06 (m, 2H), 3.60 (q, 2H, *J* = 3.3 Hz), 1.17 (t, 3H, *J* = 7.0 Hz);

^{13}C-NMR (75 MHz, $CDCl_3$), δ (ppm): 155.2, 136.0, 131.9, 129.0, 128.6, 128.5, 128.3, 121.7, 85.0, 84.6, 72.8, 67.4, 63.0, 15.1;

HRMS calcd. for $C_{19}H_{19}NO_3Na^+$ [M+Na]$^+$: 332.1257, found: 332.1259

Characterization of Mannich-type adducts (3a-q)

1 (3*R*)-4-methoxybenzyl

3-(*t*-butoxycarbonylamino)-2-((4-methoxyphenylthio)carbonyl)-5-phenylpent-4-ynoate **(3a)**

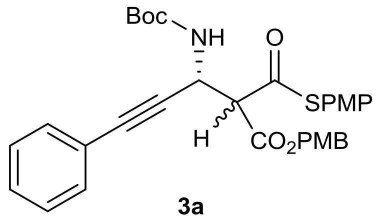

3a

Was obtained as white solid (48.9 mg, 85%) after flash chromatography (elution gradient: Ethyl Acetate/Hexane= 1/10);

$[\alpha]_D^{20}$ = + 22.8 (*c* 1.0, $CHCl_3$);

^{1}H-NMR (300 MHz, $CHCl_3$), δ (ppm): 7.22-7.18 (m, 9H), 6.85-6.73 (m, 4H), 5.59-5.51 (m, 1H), 5.32-5.30 (m, 1H), 5.22-5.01 (m, 2H), 4.11 (q, 1H, *J* = 4.2 Hz), 3.72 (q, 6H, *J* = 12.6 Hz), 1.37 (d, 9H, *J* = 4.5 Hz);

^{13}C-NMR (75 MHz, $CDCl_3$), δ(ppm): 192.8, 190.9, 166.6, 165.6, 161.0, 159.8, 154.5, 136.2, 136.1, 131.8, 130.2, 128.6, 128.2, 127.2, 127.2, 122.2, 122.2, 117.3, 117.2, 115.0, 115.0, 114.0, 85.3, 84.4, 80.3, 67.7, 67.5, 62.7, 62.1, 55.4, 55.2, 43.6, 28.3;

HRMS calcd. for $C_{32}H_{33}NO_7SK^+$ $[M+K]^+$: 614.1609, found: 614.1610

HPLC analysis: Daicel CHIRALPAK AD-H, *n*-hexane/*i*-PrOH = 80/20, flow rate = 0.8 mL/min, λ = 254 nm, retention time: $t_{(minor)}$ = 25.4 min, 41.0 min, $t_{(major)}$ = 29.5 min, 46.6 min;

2 (3*R*)-4-methoxybenzyl

3-(*t*-butoxycarbonylamino)-2-((4-methoxyphenylthio)carbonyl)-5-*o*-tolylpent-4-ynoate **(3b)**

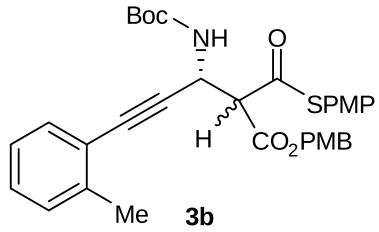

3b

Was obtained as yellow solid (49.5 mg, 84%) after flash chromatography (elution gradient: Ethyl Acetate/Hexane= 1/10);

$[\alpha]_D^{20}$ = +9.9 (*c* 1.0, $CHCl_3$);

^{1}H-NMR (300 MHz, $CDCl_3$), δ (ppm): 7.25-7.05 (m, 8H), 6.89-6.78 (m, 4H), 5.73 (s, 1H), 5.42 (d, 1H, *J* = 6.0 Hz), 5.23-5.08 (m, 2H), 4.23 (t, 1H, *J* = 10.2 Hz), 3.75 (t, 6H, *J* = 10.5 Hz), 2.33 (d, 3H, *J* = 7.8 Hz), 1.45 (s, 9H);

^{13}C-NMR (75 MHz, $CDCl_3$), δ (ppm): 192.7, 190.9, 166.6, 165.6, 161.0, 159.8, 154.7, 140.6, 136.2, 136.1, 132.2, 130.2, 129.4, 128.7, 127.2, 125.5, 122.0, 122.0, 117.3, 117.2, 115.0, 115.0, 114.0, 89.3, 83.4, 80.3, 67.7, 67.5, 62.8, 62.1, 55.4, 55.3, 53.5, 43.8, 28.3, 20.7, 20.6;

HRMS calcd. for $C_{33}H_{35}NO_7SK^+$ $[M+K]^+$: 628.1975, found: 628.1975

HPLC analysis: Daicel CHIRALPAK AD-H, *n*-hexane/*i*-PrOH = 80/20, flow rate = 0.8 mL/min, λ = 254 nm, retention time: $t_{(minor)}$ = 25.6 min, 39.4 min, $t_{(major)}$ = 34.6 min, 46.2 min;

3 (3*R*)-4-methoxybenzyl

3-(*t*-butoxycarbonylamino)-2-((4-methoxyphenylthio)carbonyl)-5-*m*-tolylpent-4-ynoate **(3c)**

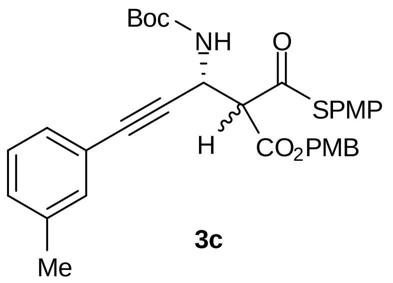

3c

Was obtained as yellow solid (47.7 mg, 81%) after flash chromatography (elution gradient: Ethyl Acetate/Hexane= 1/10);

$[\alpha]_D^{20}$ = +9.0 (*c* 1.0, $CHCl_3$);

^{1}**H-NMR** (300 MHz, $CDCl_3$), δ (ppm): 7.160-7.08 (m, 4H), 7.06-7.02 (m, 4H), 6.84-6.72 (m, 4H), 5.54(br s, 1H), 5.30 (br s, 1H), 5.08-5.00 (m, 2H), 4.10 (t, 6H, *J* = 10.5 Hz), 3.70 (t, 6H, *J* = 12.3 Hz), 2.22 (s, 3H), 1.38 (s, 9H);

^{13}**C-NMR** (75 MHz, $CDCl_3$), δ (ppm): 192.9, 191.1, 166.6, 165.6, 161.1, 159.8, 154.5, 137.9, 136.2, 136.1, 132.4, 130.2, 129.5, 128.9, 128.1, 127.2, 127.2, 122.0, 117.4, 117.2, 115.0, 115.0, 114.0, 84.9, 89.7, 80.3, 67. 7, 67.5, 62.7, 62.1, 55.4, 55.2, 43.6, 28.3, 21.2;

HRMS calcd. for $C_{33}H_{35}NO_7SK^+$ $[M+K]^+$: 628.1975, found: 628.1975

HPLC analysis: Daicel CHIRALPAK AD-H, *n*-hexane/*i*-PrOH = 80/20, flow rate = 0.8 mL/min, λ = 254 nm, retention time: $t_{(minor)}$ = 24.1 min, 35.0 min, $t_{(major)}$ = 29.0 min, 46.1 min;

4 (3*R*)-4-methoxybenzyl

3-(*t*-butoxycarbonylamino)-2-((4-methoxyphenylthio)carbonyl)-5-*p*-tolylpent-4-ynoate **(3d)**

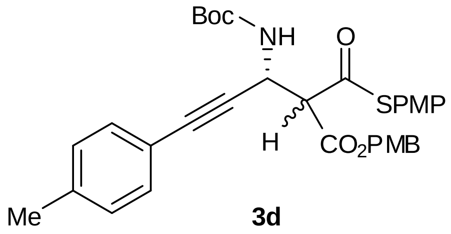

3d

Was obtained as pale yellow solid (47.2 mg, 80%) after flash chromatography (elution gradient: Ethyl Acetate/Hexane= 1/10);

$[\alpha]_D^{20}$ = +14.8 (*c* 1.0, $CHCl_3$);

^{1}**H-NMR** (300 MHz, $CDCl_3$), δ (ppm): 7.32-7.19 (m, 7H), 7.07 (d, 2H, *J* = 7.2 Hz), 6.90-6.79 (m, 4H), 5.72-5.64 (m, 1H), 5.43-5.38 (m, 1H), 5.26-5.07 (m, 2H), 4.19 (t, 1H, *J* = 12.3 Hz), 3.76 (t, 6H, *J* = 4.2 Hz), 2.32 (s, 3H), 1.45 (s, 9H);

^{13}**C-NMR** (75 MHz, $CDCl_3$), δ (ppm): 192.9, 191.0, 166.6, 165.6, 161.0, 159.8, 154.6, 138.7, 136.2, 136.1, 131.8, 130.2, 129.0, 127.3, 127.2, 119.2, 119.1, 117.4, 117.2, 115.0, 115.0, 114.0, 84.6, 80.3, 77.4, 67.7, 67.5, 62.8, 62.1, 55.4, 55.2, 43.6, 29.7, 28.3, 21.5;

HRMS calcd. for $C_{33}H_{35}NO_7SK^+$ $[M+K]^+$: 628.1975, found: 628.1975

HPLC analysis: Daicel CHIRALPAK AD-H, *n*-hexane/*i*-PrOH = 80/20, flow rate = 0.8 mL/min, λ = 254 nm, retention time: $t_{(minor)}$ = 28.0 min, 48.0 min, $t_{(major)}$ = 30.3 min, 39.0 min;

5 (3*R*)-4-methoxybenzyl

3-(*t*-butoxycarbonylamino)-5-(4-methoxyphenyl)-2-((4-methoxyphenylthio)carbonyl)pent-4-ynoate **(3e)**

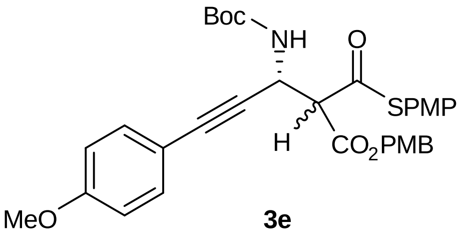

3e

Was obtained as yellow solid (50.3 mg, 83%) after flash chromatography (elution gradient: Ethyl Acetate/Hexane= 1/4);

$[\alpha]_D^{20}$ = +15.9 (*c* 1.0, $CHCl_3$);

^{1}H-NMR (300 MHz, $CDCl_3$), δ (ppm): 7.32-7.22 (m, 6H), 6.90-6.77 (m, 6H), 5.73-5.63 (d, 1H, *J* = 28.5 Hz), 5.42-5.63 (d, 1H, *J* = 10.5 Hz), 5.26-5.07 (m, 2H), 4.21-4.19 (t, 1H, *J* = 11.7 Hz), 3.77-3.75 (t, 9H, *J* =8.1 Hz), 1.45 (s, 9H);

^{13}C-NMR (75 MHz, $CDCl_3$), δ (ppm): 192.9, 191.0, 166.6, 165.6, 161.0, 159.8, 159.8, 154.6, 136.2, 136.1, 133.3, 130.2, 127.3, 127.2, 117.4, 117.2, 115.0, 115.0, 114.3, 114.2, 114.0, 113.9, 84.5, 84.0, 80.2, 77.4, 67.6, 67.5, 62.8, 62.2, 55.3, 43.7, 28.3;

HRMS calcd. for $C_{33}H_{35}NO_8SK^+$ $[M+K]^+$: 644.1714, found: 644.1716

HPLC analysis: Daicel CHIRALPAK AD-H, *n*-hexane/*i*-PrOH = 80/20, flow rate = 0.8 mL/min, λ = 254 nm, retention time: t_R = 39.8 min (mjor), t_R = 47.9 min (minor)

6 (3*R*)-4-methoxybenzyl

5-(4-bromophenyl)-3-(*t*-butoxycarbonylamino)-2-((4-methoxyphenylthio)carbonyl)pent-4-ynoate **(3f)**

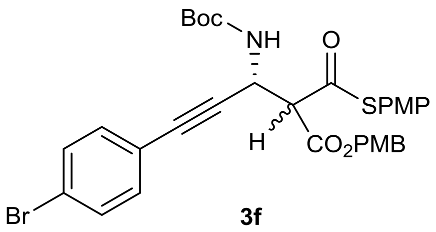

3f

Was obtained as yellow solid (58.9 mg, 90%) after flash chromatography (elution gradient: Ethyl Acetate/Hexane= 1/6);

$[\alpha]_D^{20}$ = +12.9 (*c* 1.0, $CHCl_3$);

^{1}H-NMR (300 MHz, $CDCl_3$), δ (ppm): 7.31-7.24 (m, 2H), 7.24-7.18 (m, 4H), 7.12-7.04 (m, 2H), 6.85-6.72 (m, 4H), 5.58-5.49 (m, 1H), 5.32-5.28 (m, 1H), 5.26-5.00 (m, 2H), 4.10 (q, 1H, *J* = 3.0 Hz), 3.71 (t, 6H, *J* = 11.7 Hz), 1.36 (d, 9H, *J* = 3.6 Hz);

^{13}C-NMR (75 MHz, $CDCl_3$), δ (ppm): 192.6, 190.9, 166.5, 165.5, 161.1, 159.8, 154.6, 136.2, 136.1, 133.3, 131.5, 130.2, 127.2, 127.1, 122.9, 121.2, 121.1, 117.2, 117.1, 115.1, 115.0, 114.0, 86.5, 83.5, 83.4, 80.4, 67.7, 67.6, 62.5, 61.9, 55.4, 55.2, 43.5, 28.3;

HRMS calcd. for $C_{32}H_{32}BrNO_7SK^+$ $[M+K]^+$: 692.0714, found: 692.0709

HPLC analysis: Daicel CHIRALPAK AD-H, *n*-hexane/*i*-PrOH = 80/20, flow rate = 0.8 mL/min, λ = 254 nm, retention time: $t_{(major)}$ = 37.6 min, 45.0 min, $t_{(minor)}$ = 40.0 min, 57.0 min;

7 (3*R*)-4-methoxybenzyl 3-(*t*-butoxycarbonylamino)-5-(4-chlorophenyl)-2-((4-methoxyphenylthio)carbonyl)pent-4-ynoate **(3g)**

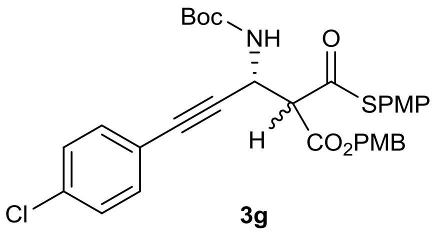

3g

Was obtained as yellow solid (49.4 mg, 81%) after flash chromatography (elution gradient: Ethyl Acetate/Hexane= 1/6);

$[\alpha]_D^{20}$ = +8.2 (*c* 1.0, $CHCl_3$);

^{1}**H-NMR** (300 MHz, $CDCl_3$), δ (ppm): 7.22-7.12 (m, 8H), 6.82-6.70 (m, 4H), 5.64-5.55 (m, 1H), 5.33-5.29 (m, 1H), 5.27-4.98 (m, 2H), 4.11 (d, 1H, *J* = 4.8 Hz), 3.67 (t, 6H, *J* = 10.5 Hz), 1.35 (s, 9H);

^{13}**C-NMR** (75 MHz, $CDCl_3$), δ (ppm): 192.7, 190.9, 166.5, 165.5, 161.0, 159.8, 154.6, 136.2, 136.1, 134.6, 133.5, 133.1, 130.3, 128.6, 127.2, 127.1, 120.7, 120.6, 117.2, 117.0, 115.1, 114.0, 114.0, 86.4, 83.4, 83.3, 80.4, 76.7, 67.7, 67.6, 62.5, 61.9, 55.4, 55.2, 43.5, 28.3;

HRMS calcd. for $C_{32}H_{32}ClNO_7SK^+$ $[M+K]^+$: 648.1219, found: 648.1219

HPLC analysis: Daicel CHIRALPAK AD-H, *n*-hexane/*i*-PrOH = 80/20, flow rate = 0.8 mL/min, λ = 254 nm, retention time: t_R = 41.3 min (major), t_R = 53.2 min (minor)

8 (3*R*)-4-methoxybenzyl 3-(*t*-butoxycarbonylamino)-2-((4-methoxyphenylthio)carbonyl)-5-(4-(trifluoromethyl)phenyl)pent-4-ynoate **(3h)**

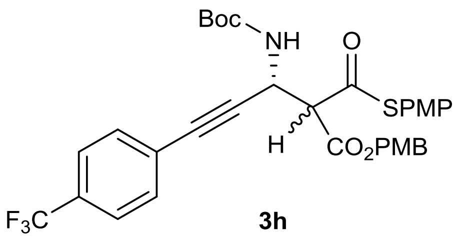

3h

Was obtained as yellow solid (51.5 mg, 80%) after flash chromatography (elution gradient: Ethyl Acetate/Hexane= 1/8);

$[\alpha]_D^{20}$ = +8.0 (*c* 1.0, $CHCl_3$);

^{1}**H-NMR** (300 MHz, $CDCl_3$), δ (ppm): 7.44-7.28 (m, 2H), 7.25-7.18 (m, 6H), 6.86-6.72 (m, 4H), 5.60-5.51 (m, 1H), 5.35-5.33 (m, 1H), 5.24-5.01 (m, 2H), 4.13 (q, 1H, *J* = 2.4 Hz), 3.74-3.68 (m, 6H), 1.37 (d, 9H, *J* = 3.3 Hz);

^{13}**C-NMR** (75 MHz, $CDCl_3$), δ (ppm): 192.5, 190.9, 166.5, 165.5, 161.1, 159.8, 154.6, 136.2, 136.1, 132.1, 130.3, 127.2, 127.1, 125.9, 125.1, 117.1, 117.0, 115.1, 115.0, 114.0, 87.8, 83.1, 80.5, 67.8, 67.6, 62.3, 61.8,

55.4, 55.2, 43.5, 28.3;

HRMS calcd. for $C_{33}H_{32}F_3NO_7SNa^+$ $[M+Na]^+$: 666.1743, found: 666.1744

HPLC analysis: Daicel CHIRALPAK AD-H, *n*-hexane/*i*-PrOH = 88/12, flow rate = 0.9 mL/min, λ = 254 nm, retention time: $t_{(major)}$ = 77.5 min, 87.8 min, $t_{(minor)}$ = 94.5 min, 115.5 min;

9 (3*R*)-4-methoxybenzyl

3-(*t*-butoxycarbonylamino)-2-((4-methoxyphenylthio)carbonyl)-5-(thiophen-2-yl)pent-4-ynoate **(3i)**

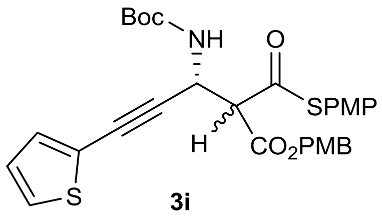

3i

Was obtained as yellow solid (50.0 mg, 86%) after flash chromatography (elution gradient: Ethyl Acetate/Hexane= 1/8);

$[\alpha]_D^{20}$ = +13.7 (*c* 1.0, $CHCl_3$);

^{1}H-NMR (300 MHz, $CDCl_3$), δ (ppm): 7.22-7.14 (m, 5H), 7.12-7.11 (m, 1H), 6.95-6.82 (m, 5H), 5.66-5.44 (m, 1H), 5.40-5.39 (m, 1H), 5.26-5.09 (m, 2H), 4.18 (t, 1H, *J* = 11.4 Hz), 3.78 (q, 6H, *J* = 4.5 Hz), 1.43 (d, 9H, *J* = 4.5Hz);

^{13}C-NMR (75 MHz, $CDCl_3$), δ (ppm): 192.9, 190.9, 166.5, 165.5, 161.1, 161.0, 159.8, 154.5, 136.2, 136.1, 132.7, 132.6, 130.2, 127.5, 127.2, 127.1, 126.9, 122.1, 122.1, 117.3, 117.1, 115.1, 115.0, 114.0, 89.2, 80.4, 77.9, 67.7, 67.6, 62.5, 61.8, 55.4, 55.3, 43.8, 28.3;

HRMS calcd. for $C_{30}H_{31}NO_7S_2Na^+$ $[M+Na]^+$: 604.1434, found: 604.1435

HPLC analysis: Daicel CHIRALPAK AD-H, *n*-hexane/*i*-PrOH = 80/20, flow rate = 0.8 mL/min, λ = 254 nm, retention time: $t_{(minor)}$ = 31.1 min, 52.8 min, $t_{(major)}$ = 39.2 min, 59.5 min;

10 (3*R*,*E*)-4-methoxybenzyl

3-(*t*-butoxycarbonylamino)-2-((4-methoxyphenylthio)carbonyl)-7-phenylhept-6-en-4-ynoate **(3j)**

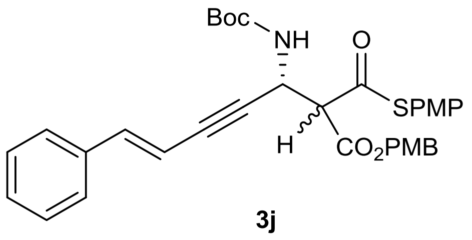

3j

Was obtained as yellowish-brown solid (52.3 mg, 87%) after flash chromatography (elution gradient: Ethyl Acetate/Hexane= 1/6);

$[\alpha]_D^{20}$ = +15.9 (*c* 1.0, $CHCl_3$);

^{1}H-NMR (300 MHz, $CDCl_3$), δ (ppm): 7.25-7.18 (m, 8H), 6.86-6.75 (m, 5H), 6.02-5.93 (m, 1H), 5.54-5.44 (m, 1H), 5.30-5.24 (m, 1H), 5.20-5.02 (m, 2H), 4.08 (t, 1H, *J* = 9.3 Hz), 3.69 (t, 6H, *J* = 23.4 Hz), 1.37 (d, 9H, *J* = 3.9 Hz);

^{13}C-NMR (75 MHz, $CDCl_3$), δ (ppm): 192.9, 191.0, 166.6, 165.6, 161.0, 159.8, 154.6, 142.3, 136.2, 136.1,

136.0, 130.2, 128.7, 127.3, 126.3, 117.4, 117.2, 115.0, 115.0, 114.0, 107.1, 87.3, 83.8, 80.3, 67.7, 67.5, 62.6, 62.1, 55.4, 55.2, 43.7, 28.3;

HRMS calcd. for $C_{34}H_{35}NO_7SNa^+$ [M+Na$^+$]: 624.2026, found: 624.2027

HPLC analysis: Daicel CHIRALPAK AD-H, *n*-hexane/*i*-PrOH = 80/20, flow rate = 0.8 mL/min, λ = 254 nm, retention time: $t_{(major)}$ = 40.7 min, 58.3 min, $t_{(minor)}$ = 53.5 min, 72.7 min;

11 (3*R*,*E*)-4-methoxybenzyl

3-(*t*-butoxycarbonylamino)-7-(4-chlorophenyl)-2-((4-methoxyphenylthio)carbonyl)hept-6-en-4-ynoate **(3k)**

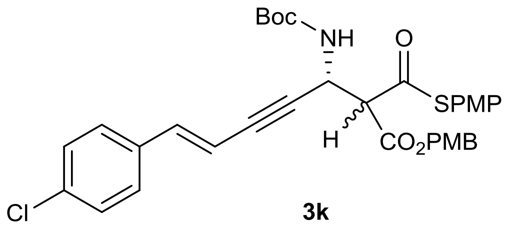

3k

Was obtained as yellowish-brown solid (50.9 mg, 80%) after flash chromatography (elution gradient: Ethyl Acetate/Hexane= 1/6);

$[\alpha]_D^{20}$ = +9.4 (*c* 1.0, $CHCl_3$);

^{1}H-NMR (300 MHz, $CDCl_3$), δ (ppm): 7.30-7.25 (m, 8H), 6.93-6.83 (m, 4H), 6.80-6.75 (m, 1H), 6.05-5.96 (m, 1H), 5.64-5.57 (m, 1H), 5.56-5.54 (m, 1H), 5.38-5.09 (m, 2H), 4.15 (q, 1H, *J* = 4.5 Hz), 3.80-3.73 (m, 6H), 1.44 (d, 9H, *J* = 3.9 Hz);

^{13}C-NMR (75 MHz, $CDCl_3$), δ (ppm): 192.9, 191.0, 166.6, 165.6, 161.0, 159.8, 154.6, 140.9, 136.2, 136.1, 134.5, 130.3, 128.9, 127.5, 127.2, 127.2, 117.3, 117.1, 115.0, 115.0, 114.0, 107.9, 107.8, 87.9, 83.4, 83.3, 80.4, 67.7, 67.5, 62.5, 62.0, 55.4, 55.2, 43.7, 28.3;

HRMS calcd. for $C_{34}H_{34}ClNO_7SNa^+$ [M+Na]$^+$: 658.1636, found: 658.1640

HPLC analysis: Daicel CHIRALPAK AD-H, *n*-hexane/*i*-PrOH = 80/20, flow rate = 0.8 mL/min, λ = 254 nm, retention time: $t_{(major)}$ = 50.2 min, 65.1 min, $t_{(minor)}$ = 75.0 min, 93.5 min;

12 (3*R*)-4-methoxybenzyl

3-(*t*-butoxycarbonylamino)-2-((4-methoxyphenylthio)carbonyl)-7-phenylhept-4-ynoate **(3l)**

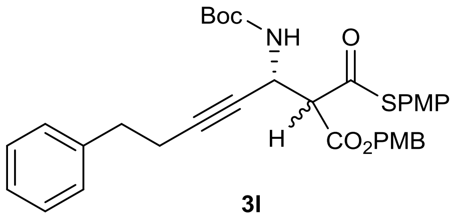

3l

Was obtained as white solid (48.9 mg, 81%) after flash chromatography (elution gradient: Ethyl Acetate/Hexane= 1/10);

$[\alpha]_D^{20}$ = +14.2 (*c* 0.6, $CHCl_3$);

^{1}H-NMR (300 MHz, $CDCl_3$), δ (ppm): 7.29-7.16 (m, 9H), 6.90-6.84 (m, 4H), 5.56-5.47 (m, 1H), 5.19-5.06 (m, 3H), 4.04 (s, 1H), 3.77 (t, 6H, *J* = 14.1 Hz), 2.72 (q, 2H, *J* = 7.8 Hz), 2.39 (q, 2H, *J* = 6.9 Hz), 1.42 (d, 9H, *J* = 4.5 Hz);

^{13}C-NMR (75 MHz, $CDCl_3$), δ (ppm): 192.8, 191.0, 166.6, 165.7, 161.0, 159.8, 154.6, 140.5, 136.2, 136.1, 130.2, 128.4, 127.3, 127.2, 126.3, 117.4, 117.2, 115.0, 115.0, 114.0, 84.5, 84.4, 80.1, 77.3, 67.6, 67.4, 62.8, 55.4, 55.3, 43.2, 34.9, 34.8, 28.3,20.8;

HRMS calcd. for $C_{34}H_{37}NO_7SNa^+$ $[M+Na]^+$: 626.2182, found: 626.2185

HPLC analysis: Daicel CHIRALPAK AD-H, *n*-hexane/*i*-PrOH = 80/20, flow rate = 0.8 mL/min, λ = 254 nm, retention time: $t_{(minor)}$ = 30.9 min, 47.4 min, $t_{(major)}$ = 39.2 min, 42.4 min;

13 (3*R*)-4-methoxybenzyl 3-(*t*-butoxycarbonylamino)-2-((4-methoxyphenylthio)carbonyl)non-4-ynoate **(3m)**

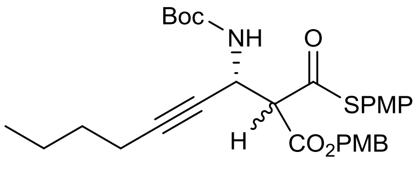

3m

Was obtained as yellow solid (44.5 mg, 80%) after flash chromatography (elution gradient: Ethyl Acetate/Hexane= 1/8);

$[\alpha]_D^{20}$= -7.5 (*c* 1.0, $CHCl_3$);

^{1}H-NMR (300 MHz, $CDCl_3$), δ (ppm): 7.26-7.20 (m, 4H), 6.86-6.80 (m, 4H), 5.45 (s, 1H), 5.14-5.01 (m, 3H), 3.99 (t, 1H, *J* = 6 Hz), 3.74 (t, 6H, *J* = 3.3 Hz), 2.08-2.01 (m, 2H), 1.36 (s, 1H), 1.34-1.14 (m, 4H), 0.83-0.78 (m, 3H);

^{13}C-NMR (75 MHz, $CDCl_3$), δ (ppm): 192.9, 190.9, 166.6, 165.6, 161.0, 159.8, 154.6, 136.2, 136.0, 130.2, 127.2, 117.5, 117.3, 115.0, 114.9, 113.9, 85.2, 80.0, 67.5, 67.3, 62.9, 55.4, 55.3, 43.2, 30.5, 28.3, 21.8, 18.3, 13.5;

HRMS calcd. for $C_{30}H_{37}NO_7SNa^+$ $[M+Na]^+$: 578.2782, found: 578.2182

HPLC analysis: Daicel CHIRALPAK AD-H, *n*-hexane/*i*-PrOH = 85/15, flow rate = 0.9 mL/min, λ = 254 nm, retention time: t($_{minor)}$ = 28.7 min, 41.6 min, $t_{(major)}$ = 34.1 min, 36.3 min;

14 (3*R*)-4-methoxybenzyl

3-(benzyloxycarbonylamino)-2-((4-methoxyphenylthio)carbonyl)-5-phenylpent-4-ynoate **(3n)**

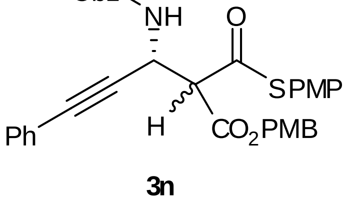

3n

Was obtained as yellow solid (50.0 mg, 82%) after flash chromatography (elution gradient: Ethyl Acetate/Hexane= 1/8);

$[\alpha]_D^{20}$= +19.0 (*c* 1.0, $CHCl_3$);

^{1}H-NMR (300 MHz, $CDCl_3$), δ (ppm): 7.31-7.22 (m, 13H), 6.89-6.78 (m, 4H), 5.98-5.90 (m, 1H), 5.48-5.44 (m, 1H), 5.24-5.44 (m, 4H), 4.20-4.09 (m, 1H), 3.70 (t, 6H, *J* = 11.7Hz);

^{13}C-NMR (75 MHz, $CDCl_3$), δ (ppm): 192.9, 190.9, 166.5, 165.5, 161.1, 159.8, 155.4, 136.2, 136.1, 131.9,

130.3, 128.7, 128.5, 128.3, 128.1, 128.0, 127.2, 122.0, 117.2, 117.0, 115.1, 114.0, 85.0, 67.8, 67.7, 67.2, 62.6, 61.2, 55.4, 55.3, 44.0;

HRMS calcd. for $C_{35}H_{31}NO_7SNa^+$ [M+Na]$^+$: 632.1713, found: 632.1715

HPLC analysis: Daicel CHIRALPAK AD-H, *n*-hexane/*i*-PrOH = 80/20, flow rate = 0.8 mL/min, λ = 254 nm, retention time: $t_{(major)}$ = 49.2 min, 55.5 min, $t_{(minor)}$ = 52.4 min, 75.0 min;

15 (*R*)-*S,S*-diphenyl 2-(1-(*t*-butoxycarbonylamino)-3-phenylprop-2-ynyl)propanebis(thioate) **(3o)**

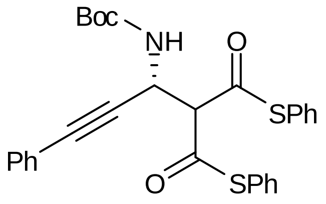

3o

Was obtained as yellow solid (41.4 mg, 80%) after flash chromatography (elution gradient: Ethyl Acetate/Hexane= 1/15);

[α]$_D^{20}$ = +1.2 (*c* 1.0, $CHCl_3$);

^{1}H-NMR (300 MHz, $CDCl_3$), δ (ppm): 7.46-7.39 (m, 12H), 7.33-7.25 (m, 3H), 5.547 (s, 1H), 5.43 (d, 1H, *J* = 7.5 Hz), 4.55 (d, 1H, *J* = 6.0 Hz), 1.45 (s, 9H);

^{13}C-NMR (75 MHz, $CDCl_3$), δ (ppm): 188.8, 154.5, 134.5, 134.4, 131.9, 130.0, 129.9, 129.4, 129.3, 128.7, 128.3, 126.5, 122.1, 85.2, 84.9, 80.5, 69.2, 44.5, 28.3;

HRMS calcd. for $C_{29}H_{27}NO_4S_2K^+$ [M+K]$^+$: 556.1006, found: 506.1013

HPLC analysis: Daicel CHIRALPAK AS-H, *n*-hexane/*i*-PrOH = 93/7, flow rate = 0.7 mL/min, λ = 254 nm, retention time: t_R = 19.0 min (major), t_R = 31.1 min (minor)

16 (*R*)-diethyl 2-(1-(*t*-butoxycarbonylamino)-3-phenylprop-2-ynyl)malonate **(3p)**

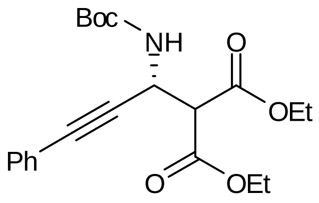

3p

Was obtained as yellow solid (29.2 mg, 75%) after flash chromatography (elution gradient: Ethyl Acetate/Hexane= 1/10);

[α]$_D^{20}$ = +21.6 (*c* 1.0, $CHCl_3$);

^{1}H-NMR (300 MHz, $CDCl_3$), δ (ppm): 7.39-7.37 (m, 2H), 7.30-7.28 (m, 3H), 5.75 (s, 1H), 5.36 (s, 1H), 4.29-4.22 (m, 4H), 3.85 (d, 1H, *J* = 5.1 Hz), 1.45 (s, 9H), 1.25-1.19 (m, 6H);

^{13}C-NMR (75 MHz, $CDCl_3$), δ (ppm): 167.3, 166.4, 154.6, 131.8, 128.5, 128.2, 122.3, 85.7, 83.8, 80.2, 62.0, 56.3, 42.7, 28.3, 14.1, 14.0;

HPLC analysis: Daicel CHIRALPAK AS-H, *n*-hexane/*i*-PrOH = 97/3, flow rate = 0.5 mL/min, λ = 254 nm,

retention time: t_R = 12.3 min (major), t_R = 14.7 min (minor);

17 (*R*)-dibenzyl 2-(1-(*t*-butoxycarbonylamino)-3-phenylprop-2-ynyl)malonate **(3q)**

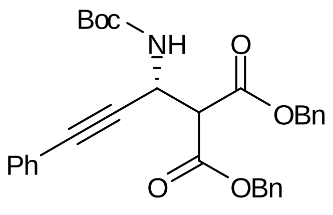

3q

Was obtained as white solid (38.0 mg, 74%) after flash chromatography (elution gradient: Ethyl Acetate/Hexane= 1/10);

$[\alpha]_D^{20}$ = +6.7 (*c* 1.0, $CHCl_3$);

^{1}H-NMR (300 MHz, $CDCl_3$), δ (ppm): 7.32-7.26 (m, 15H), 5.69 (s, 1H), 5.39 (s, 1H), 5.29-5.13 (m, 4H), 3.97 (d, 1H, *J* = 4.8 Hz), 1.44 (s, 9H);

^{13}C-NMR (75 MHz, $CDCl_3$), δ (ppm): 167.1, 166.2, 154.6, 135.3, 135.1, 135.0, 131.9, 128.6, 128.5, 128.3, 128.2, 122.2, 85.5, 84.1, 80.4, 56.3, 42.8, 41.6, 28.32;

HRMS calcd. for $C_{31}H_{31}NO_6K^+$ $[M+K]^+$: 552.1777, found: 552.1783

HPLC analysis: Daicel CHIRALPAK AS-H, *n*-hexane/*i*-PrOH = 96/4, flow rate = 0.7 mL/min, λ = 254 nm, retention time: t_R = 19.8 min (major), t_R = 22.9 min (minor)

Characterization of 4

(*R*)-*S*-4-methoxyphenyl 3-(*t*-butoxycarbonylamino)-5-phenylpent-4-ynethioate **(4)**

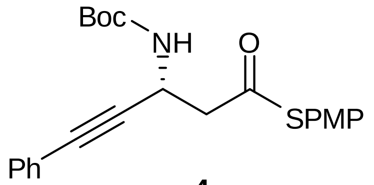

4

$[\alpha]_D^{20}$ = +34.5 (*c* 1.0, $CHCl_3$);

^{1}H-NMR (300 MHz, $CDCl_3$), δ (ppm): 7.36-7.33 (m, 2H), 7.26-7.18 (m, 5H), 6.86 (q, *J* = 1.8 Hz), 5.27 (s, 1H), 4.96 (t, 1H, *J* = 8.1 Hz), 3.75 (s, 3H), 3.08 (dd, 1H, *J* = 5.1, *J* = 5.4 Hz), 2.96 (dd, 1H, *J* = 5.7 Hz, *J* = 5.7 Hz)), 1.38 (s, 9H);

^{13}C-NMR (75 MHz, $CDCl_3$), δ (ppm): 195.9, 160.8, 154.6, 136.1, 131.8, 128.5, 128.3, 122.4, 118.0, 115.0, 86.6, 83.9, 80.2, 55.4, 48.2, 45.0, 28.4;

HRMS calcd. for $C_{23}H_{25}NO_4$ Na^+ $[M+Na]^+$: 343.1392, found: 434.1397

HPLC analysis: Daicel CHIRALPAK AD-H, *n*-hexane/*i*-PrOH = 85/15, flow rate = 0.8 mL/min, λ = 254 nm, retention time: t_R = 17.9 min (minor), t_R = 21.0 min (major);

Characterization of 5

(*R*)-3-(*t*-butoxycarbonylamino)-5-phenylpent-4-ynoic acid **(5)**

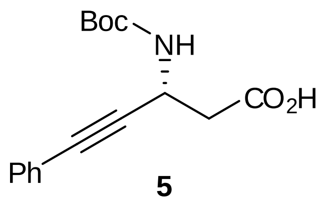

5

[α]$_D$^{20} = −21.2 (*c* 1.0, $CHCl_3$);

^{1}H-NMR (300 MHz, $CDCl_3$), δ (ppm): 7.39 (t, 2H, *J* = 7.8 Hz), 7.28 (t, 3H, *J* = 7.2 Hz), 5.45 (s, 1H), 5.03 (s,1H), 2.86 (t, 2H, *J* = 6.3 Hz), 1.46 (s, 9H);

^{13}C-NMR (75 MHz, $CDCl_3$), δ (ppm): 175.5, 154.8, 131.8, 128.5, 128.2, 122.4, 86.9, 83.3, 80.4, 40.4, 39.8, 28.3;

HRMS calcd. for $C_{16}H_{19}NO_4Na^+$ $[M+Na]^+$: 312.1205, found: 312.1206

Characterization of 6

(*S*)-3-(*t*-butoxycarbonylamino)-5-phenylpentanoic acid **(6)**

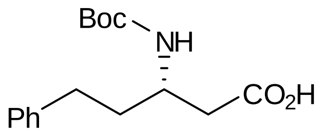

6

[α]$_D$^{20} = -2.5 (*c* 1.0, EtOH);

^{1}H-NMR (300 MHz, $CDCl_3$), δ (ppm): 7.30-7.25 (m, 2H), 7.20-7.16 (m, 3H), 5.01 (d, *J* = 6.0 Hz), 3.97 (s, 1H), 2.72-2.60 (m, 4H), 1.92-1.82 (m, 2H), 1.45 (s, 9H);

^{13}C-NMR (75 MHz, $CDCl_3$), δ (ppm): 176.6, 155.5, 141.3, 128.5, 128.4, 126.0, 79.6, 47.2, 39.2, 36.3, 32.6, 28.4

Characterization of 7

(*R*,*E*)-*t*-butyl 5-hydroxy-1-phenylpent-1-en-3-ylcarbamate **(7)**

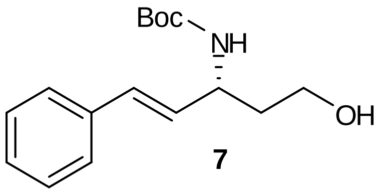

7

[α]$_D$^{20} = +30.6 (*c* 1.0, $CHCl_3$);

^{1}H-NMR (300 MHz, $CDCl_3$), δ (ppm): 7.38-7.24 (m, 5H), 6.57 (d, 1H, *J* = 15.9 Hz), 6.19 (dd, 1H, *J* = 5.7 Hz, *J* = 5.7 Hz), 4.76 (d, 1H, *J* = 7.5 Hz), 4.56 (s, 1H), 3.80-3.71 (m, 3H), 2.05-1.97 (m, 1H), 1.60 (q, 1H, *J* = 6.9 Hz), 1.47 (s, 9H);

^{13}C-NMR (75 MHz, $CDCl_3$), δ (ppm): 156.6, 136.7, 130.5, 129.8, 128.7, 127.8, 126.5, 80.2, 58.9, 49.0, 38.6, 28.5

Characterization of 9

(*R*)-*t*-butyl 5-oxo-1-phenylpent-1-yn-3-ylcarbamate **(9)**

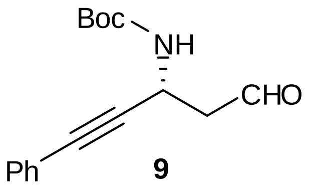

9

$[\alpha]_D^{20}$ = +131.7 (*c* 1.0, $CHCl_3$);

^{1}H-NMR (300 MHz, $CDCl_3$), δ (ppm): 9.84 (s, 1H), 7.41-7.38 (m, 2H), 7.32-7.26 (m, 3H), 5.19 (d, 1H, *J* = 8.1 Hz), 5.07 (d, 1H, *J* = 5.1 Hz), 2.93-2.90 (m, 2H), 1.46 (s, 9H);

^{13}C-NMR (75 MHz, $CDCl_3$), δ (ppm): 199.4, 154.7, 131.7, 128.6, 128.3, 122.1, 86.7, 84.1, 80.4, 49.3, 38.9, 28.3;

HRMS calcd. for $C_{16}H_{19}NO_3Na^+$ [M+Na$^+$]: 296.1250, found: 296.1257

Characterization of 10

(*R*,*E*)-ethyl 5-(*t*-butoxycarbonylamino)-7-phenylhept-2-en-6-ynoate **(10)**

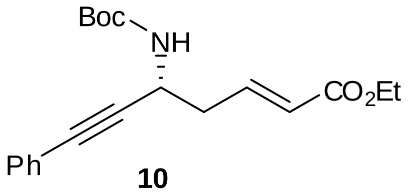

10

$[\alpha]_D^{20}$ = +63.7 (*c* 1.0, $CHCl_3$);

^{1}H-NMR (300 MHz, $CDCl_3$), δ (ppm): 7.42-7.38 (m, 2H), 7.31-7.28 (m, 3H), 7.08-6.98 (m, 1H), 5.97 (d, 1H, *J* = 15.6 Hz), 5.10 (d, 1H, *J* = 7.8 Hz), 4.83 (d, 1H, *J* = 6.6 Hz), 4.19 (q, 2H, *J* = 7.2 Hz), 2.65 (t, 2H, *J* = 13.2 Hz), 1.46 (s, 9H), 1.28 (t, 3H, *J* = 14.4 Hz);

^{13}C-NMR (75 MHz, $CDCl_3$), δ (ppm): 166.1, 154.6, 143.0, 131.7, 128.5, 128.3, 124.9, 122.3, 87.2, 84.3, 84.2, 60.3, 42.6, 38.9, 28.3, 14.2;

HRMS calcd. for $C_{20}H_{25}NO_4Na^+$ [M+Na]$^+$: 366.1680, found: 366.1676

Characterization of *syn*-propargylamines (12a-f)

1 (*R*)-ethyl 1-((*S*)-1-(*t*-butoxycarbonylamino)-3-phenylprop-2-ynyl)-2-oxocyclopentanecarboxylate **(12a)**

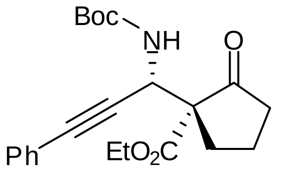

12a

Was obtained as white solid (32.4 mg, 84%) after flash chromatography (elution gradient: Ethyl Acetate/Hexane= 1/10);

$[\alpha]_D^{20}$= +2.3 (*c* 1.0, $CHCl_3$);

^{1}H-NMR (500 MHz, $CDCl_3$), δ (ppm): 7.38-7.36 (m, 2H), 7.31-7.26 (m, 3H), 6.08 (br d, 0.02H, *J* = 9.0 Hz) 5.43 (br s, 0.75 H), 5.22 (d, 1H, *J* = 9.5 Hz), 4.27-4.16 (m, 2H), 2.68-2.62 (m, 1H), 2.47-2.32 (m, 3H), 2.14-2.02 (m, 2H), 1.44 (s, 9H), 1.27 (t, 3H *J* = 7.0 Hz);

^{13}C-NMR (75 MHz, $CDCl_3$), δ (ppm): 210.4, 168.9, 154.9, 131.8, 128.5, 128.3, 122.3, 85.4, 84.0, 80.3, 64.9,

61.9, 45.6, 37.7, 31.2, 28.3, 19.2, 14.1;

HPLC analysis: Daicel CHIRALPAK AD-H, *n*-hexane/*i*-PrOH = 97/3, flow rate = 0.8 mL/min, λ = 254 nm, retention time: t_R = 17.6 min (major), t_R = 25.5 min (minor) (major diastereomer)

2 (*R*)-ethyl 1-((*S*)-1-(*t*-butoxycarbonylamino)-3-*p*-tolylprop-2-ynyl)-2-oxocyclopentanecarboxylate **(12b)**

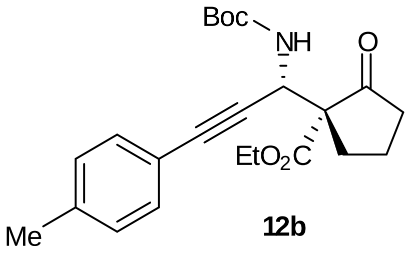

12b

Was obtained as colourless liquid (34.4 mg, 86%) after flash chromatography (elution gradient: Ethyl Acetate/Hexane= 1/10);

$[\alpha]_D^{20}$= +0.3 (*c* 1.0, $CHCl_3$);

^{1}H-NMR (500 MHz, $CDCl_3$), δ (ppm): 7.27-7.25 (d, 2H, *J* = 7.5Hz), 7.10-7.08 (d, 2H, *J* = 7.5Hz), 6.07 (br d, 0.04 H, *J* = 9.0 Hz), 5.41 (br s, 0.77 H), 5.21 (d, 1H, *J* = 10.0 Hz), 4.26-4.17 (m, 2H), 2.68-2.62 (m, 1H), 2.44-2.32 (m, 6H), 2.13-2.00 (m,2H), 1.43 (s, 9H), 1.27 (t, 3H, *J* = 7.0 Hz);

^{13}C-NMR (75 MHz, $CDCl_3$), δ (ppm): 210.5, 168.9, 155.0, 138.7, 131.6, 129.0, 119.2, 84.6, 84.1, 80.3, 64.9, 61.9, 45.6, 37.8, 31.3, 28.3, 21.5, 19.2, 14.1;

HPLC analysis: Daicel CHIRALPAK AD-H, *n*-hexane/*i*-PrOH = 97/3, flow rate = 0.8 mL/min, λ = 254 nm, retention time: t_R = 17.2 min (major), t_R = 22.6 min (minor) (major diastereomer)

3 (*R*)-ethyl 1-((*S*)-1-(*t*-butoxycarbonylamino)-3-(4-chlorophenyl)prop-2-ynyl)-2-oxocyclopentanecarboxylate **(12c)**

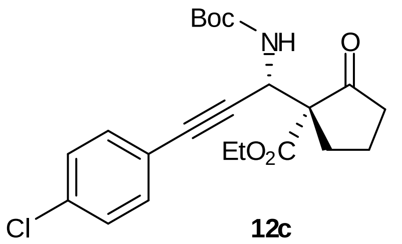

12c

Was obtained as colourless liquid (40.2 mg, 96%) after flash chromatography (elution gradient: Ethyl Acetate/Hexane= 1/10);

$[\alpha]_D^{20}$= +1.7 (*c* 1.0, $CHCl_3$);

^{1}H-NMR (500 MHz, $CDCl_3$), δ (ppm): 7.30-7.28 (m, 2H), 7.26-7.24 (m, 2H), 6.07 (br d, 0.03 H, *J* = 7.5 Hz), 5.42 (br s, 0.77H), 5.20 (d, 1H, *J* = 10.0 Hz), 4.27-4.16 (m, 2H), 2.67-2.61 (m, 1H), 2.45-2.36 (m, 2H), 2.34-2.29 (m, 1H), 2.13-2.02 (m, 2H), 1.44 (s, 9H), 1.26 (t, 3H, *J* = 7.0 Hz);

^{13}C-NMR (75 MHz, $CDCl_3$), δ (ppm): 210.3, 170.0, 155.0, 134.6, 133.0, 128.6, 120.8, 86.5, 82.9, 80.4, 64.7, 61.9, 45.5, 37.7, 31.3, 28.2, 19.1, 14.1;

HRMS calcd. for $C_{22}H_{26}ClNO_5^+$ $[M]^+$: 419.1500, found: 419.1505

HPLC analysis: Daicel CHIRALPAK AD-H, *n*-hexane/*i*-PrOH = 98/2, flow rate = 0.7 mL/min, λ = 254 nm,

retention time: t_R = 29.0 min (major), t_R = 35.9 min (minor) (major diastereomer)

4 (*R*)-ethyl 1-((*S*)-1-(*t*-butoxycarbonylamino)-5-phenylpent-2-ynyl)-2-oxocyclopentanecarboxylate **(12d)**

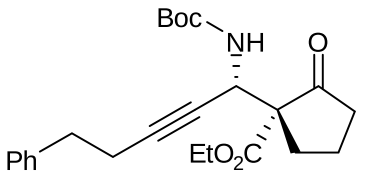

12d

Was obtained as white solid (32.4 mg, 76%) after flash chromatography (elution gradient: Ethyl Acetate/Hexane= 1/10);

$[\alpha]_D^{20}$= +9.9 (*c* 1.0, $CHCl_3$);

^{1}H-NMR (500 MHz, $CDCl_3$), δ (ppm): 7.30-7.26 (m, 2H), 7.22-7.17 (m, 3H), 5.97 (br d, 0.01H, *J* = 9.0 Hz) 5.23 (br s, 0.81 H), 4.97-4.94 (m, 1H), 4.22-4.10 (m, 2H), 2.77 (t, 2H, *J* = 7.0 Hz), 2.53-2.44 (m, 3H), 2.28 (t, 2H, *J* = 7.5 Hz), 2.13-2.08 (m, 1H), 1.98-1.92 (m, 2H), 1.43 (s, 9H), 1.24 (t, 3H *J* = 7.0 Hz);

^{13}C-NMR (75 MHz, $CDCl_3$), δ (ppm): 210.5, 168.9, 154.9, 140.4, 128.4, 128.3, 126.3, 83.9, 80.1, 64.9, 61.8, 45.3, 37.7, 34.7, 30.7, 28.3, 20.7, 19.1, 14.0;

HPLC analysis: Daicel CHIRALPAK AD-H, *n*-hexane/*i*-PrOH = 97/3, flow rate = 0.8 mL/min, λ = 210 nm, retention time: t_R = 19.9 min (major), t_R = 43.8 min (minor) (major diastereomer)

5 (*R*)-ethyl 1-((*S*)-1-(*t*-butoxycarbonylamino)-3-phenylprop-2-ynyl)-2-oxocyclohexanecarboxylate **(12e)**

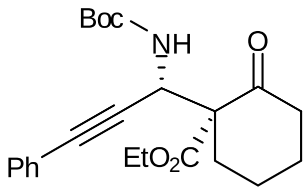

12e

Was obtained as colorless liquid (37.9 mg, 95%) after flash chromatography (elution gradient: Ethyl Acetate/Hexane= 1/10);

$[\alpha]_D^{20}$ = +64.0 (*c* 1.0, $CHCl_3$);

^{1}H-NMR (400 MHz, $CDCl_3$), δ (ppm): 7.39-7.35 (m, 2H), 7.33-7.26 (m, 3H), 5.92 (d, 0.02H, *J* = 9.6 Hz), 5.68 (d, 0.90H, *J* = 9.0 Hz), 5.32 (d, 0.95H, *J* = 9.0 Hz), 5.00 (d, 0.04H, *J* = 9.6 Hz), 4.34-4.23 (m, 2H), 2.65-2.56 (m, 2H), 2.51-2.46 (m, 1H), 2.16-2.10 (m, 1H), 1.91-1.87 (m, 3H), 1.74-1.70 (m, 1H), 1.46 (s, 9H), 1.30 (t, 3H, *J* = 7.2 Hz);

^{13}C-NMR (100 MHz, $CDCl_3$), δ (ppm): 206.9, 170.3, 155.2, 131.8, 128.4, 128.2, 122.5, 85.6, 84.2, 80.2, 66.5, 61.8, 46.0, 40.3, 33.3, 28.3, 27.2, 21.4, 14.1;

HPLC analysis: Daicel CHIRALPAK AD-H, *n*-hexane/*i*-PrOH = 98/2, flow rate = 0.8 mL/min, λ = 254 nm, retention time: t_R = 16.1 min (minor), t_R = 24.9 min (major) (major diastereomer)

6 (2*R*,3*S*)-ethyl 2-acetyl-3-(*t*-butoxycarbonylamino)-2-methyl-5-phenylpent-4-ynoate **(12f)**

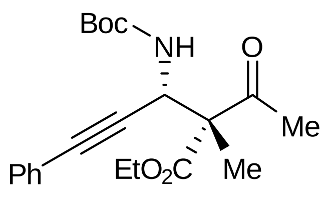

12f

Was obtained as colorless liquid (30.6 mg, 82%) after flash chromatography (elution gradient: Ethyl Acetate/Hexane= 1/15);

[α]$_D$^{20} = +77.7 (*c* 1.0, $CHCl_3$);

^{1}H-NMR (400 MHz, $CDCl_3$), δ (ppm): 7.41-7.38 (m, 2H), 7.36-7.28 (m, 3H), 5.80 (d, 0.75H, *J* = 9.6 Hz), 5.51 (d, 0.07H, *J* = 9.6 Hz), 5.22 (d, 1H, *J* = 10 Hz), 4.34-4.28 (q, 1.85H *J* = 7.2 Hz), 4.26-4.24 (m, 0.18H), 2.31 (s, 0.26H), 2.27 (s, 2.83H), 1.63 (s, 0.28H), 1.61 (s, 2.66H), 1.47 (s, 9H), 1.33 (t, 3H, *J* = 7.2 Hz);

^{13}C-NMR (100 MHz, $CDCl_3$), δ (ppm): 204.4, 170.9, 154.9, 131.8, 128.5, 128.2, 122.3, 85.4, 84.3, 80.3, 64.7, 61.8, 47.7, 28.3, 26.0, 18.8, 14.1;

HRMS calcd. for $C_{21}H_{27}NO_5Na^+$ $[M+Na]^+$: 396.1787, found: 396.1779;

HPLC analysis: Daicel CHIRALPAK AD-H, *n*-hexane/*i*-PrOH = 97/3, flow rate = 0.8 mL/min, λ = 210 nm, retention time: t_R = 13.0 min (minor), t_R = 15.2 min (major) (major diastereomer)

Characterization of 13

(*R*)-*t*-butyl 4-acetyl-5-oxo-1-phenylhex-1-yn-3-ylcarbamate **(13)**

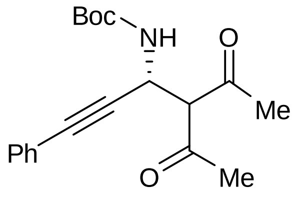

13

Was obtained as a white solid (23mg,70% yield) after flash chromatography (elution gradient: Ethyl Acetate/Hexane= 1/15);

[α]$_D$^{20} = +15.2.0(c 1.0,$CHCl_3$);

^{1}H-NMR (400 MHz, $CDCl_3$), δ (ppm): 7.30-7.28 (m, 2H), 7.21-7.19 (m, 3H), 5.48 (s, 1H), 5.28 (t, 1H, *J* = 15.0 Hz), 4.10 (d, 1H, *J* = 6.6 Hz), 2.23 (s, 3H), 2.18 (s, 3H), 1.36 (s, 9H);

^{13}C-NMR (100 MHz, $CDCl_3$), δ (ppm): 202.7, 201.7, 154.8, 131.7, 128.6, 128.3, 122.0, 85.8, 84.1, 80.5, 70.5, 42.4, 30.4, 30.1, 28.2;

HPLC analysis: Daicel CHIRALPAK AD-H, hexane/iPrOH = 98/2, flow rate = 1.0mL/min, λ = 254 nm, retention time: t_R = 49.5min (minor), t_R =54.6min (major).

DFT calculations

In order to shed light on the mechanism on the formation of C-alkynyl *N*-Boc imines, density functional theory (DFT) calculations were carried out. The hybrid density functional M06-2X[1,2] was employed. The standard 6-31G(d,p) basis sets were utilized for all atoms. Optimizations were conducted without any

constraintusing the SMD model[3] in toluene (ε = 2.3741). Frequency analyses were carried out to confirmeach structure being a local minimum (no imaginary frequency) or a transition state (only one imaginary frequency). The relative Gibbs free energies (Δ*G*) and electronic energies (Δ*E*, presented in parentheses) are given. All calculations were performed with Gaussian 09 package.[4] The 3D structure of the key transition state was prepared using CYLview.[5]

The complex (**INT-0**, 0.0 kcal/mol) of the C-alkynyl *N*,*O*-acetal **1a** and tertiary amine thiourea catalyst **D** was set as the starting point of the calculations (Supplementary Fig. 3a). Three key hydrogen bonding (one between the tertiary amine of **D** and the BocNH moiety of the **1a**; the other two between the thiourea N–H and the acetal oxygen atom of **1a**) were located. Next, a transition state (**TS**) describing the formation of the C-alkynyl *N*-Boc imine intermediate was found. In this transition state, the cleavage of the C–O bond assisted by the hydrogen bonding with the thiourea N–H moiety and the deprotonation of the BocN–H by the tertiary amine part of the catalyst were concerted. The relative energetic barrier of this transition state is 31.8 kcal/mol. The 3D structure of **TS** is depicted in Supplementary Fig. 3b. In the direct product (**INT-1**, 27.5 kcal/mol) of **TS**, the tertiary amine catalyst **G** is in a zwitterionic form. The tertiary amine part is protonated while one proton of the thiourea moiety is transferred to the ethoxide that has been dissociated from the C-alkynyl *N*,*O*-acetal. After subsequent proton transfer process (transition state for this process not located), a relatively more stabilized intermediate (**INT-2**, 15.2 kcal/mol) is formed where hydrogen bonding can be found between the ethanol and the tertiary amine moiety of the catalyst and between the C-alkynyl *N*-Boc imine and the thiourea moiety of the catalyst.

Thermodynamically, the complex of the C-alkynyl *N*-Boc imine and the catalyst (**INT-2**) is much less stabilized compared with the starting complex (**INT-0**). This is consistent with the experimental observation that the C-alkynyl *N*-Boc imine cannot be detected by ^{1}H NMR under the standard reaction conditions (without nucleophile). The equilibrium between the C-alkynyl *N*,*O*-acetal **1a** and (the in situ generated C-alkynyl *N*-Boc imine + EtOH) will shift to **1a** preferentially. It might be safe to conclude that the trapping of the very trace amount of C-alkynyl *N*-Boc imine intermediate by external nucleophiles must be quick enough to drive the equilibrium toward the formation of the C-alkynyl *N*-Boc imine intermediate.

Cartesian coordinates for all calculated stationary points

```
INT-0
Opt @ M06-2X/6-31G(d,p) intoluene (SMD model)
SCF Done: E(RM062X) = -2723.88510864 a.u.
Zero-point correction=0.731862 Hartree/Particle
Sum of electronic and thermal Free Energies=-2723.238498 a.u.
----------------------------------------------------------------
C,0,0.0376443169,4.9536565625,-0.5953446677
C,0,0.6902851989,3.9368990071,-0.602589731
C,0,1.532928442,2.734058534,-0.7074567561
H,0,2.5426113832,3.0469807289,-1.012180958
```

```
N,0,1.6754972656,2.0079785511,0.5116167719
H,0,2.5230434087,1.4102050083,0.5841289295
O,0,1.0349286279,1.8351656621,-1.7079712379
C,0,0.9838918141,2.3486490658,-3.0385736285
H,0,0.3025408717,3.206164217,-3.0737382005
H,0,1.9861896699,2.6940134599,-3.3301036432
C,0,0.580810435,1.6344898596,1.2535682576
O,0,-0.5573681615,2.0101743319,1.0529656942
O,0,0.9816099382,0.8129508518,2.2337823017
C,0,0.0442767256,0.3677104454,3.2631916723
C,0,-0.4838386095,1.5757755431,4.0288759435
C,0,-1.0744740394,-0.4639760714,2.6496277348
C,0,0.9180410474,-0.4991577623,4.160274378
H,0,0.3478969273,2.1762179181,4.4102361835
H,0,-1.1135596595,2.2003288096,3.3939457959
H,0,-1.0769040461,1.2325632732,4.8815102466
H,0,-0.6549517061,-1.2793884351,2.0509949648
H,0,-1.6726691858,-0.9063070492,3.4523330763
H,0,-1.7270166153,0.1471569281,2.0251567174
H,0,0.3263402329,-0.8806710008,4.996887302
H,0,1.3148365828,-1.3510566818,3.600322576
H,0,1.7533059977,0.0810024376,4.5637323441
C,0,6.2026452145,-1.7064430278,-1.7814832606
C,0,5.8695652768,-0.9635187242,-0.4861434282
C,0,4.468871525,-0.3317427041,-0.5426681093
C,0,3.4281994673,-1.4259921414,-0.8262873536
C,0,3.7570743219,-2.1661578075,-2.1290574057
C,0,5.1566442663,-2.7778383029,-2.083536211
H,0,5.9162680562,-1.6654133601,0.3565189099
H,0,6.6180551151,-0.1865092391,-0.3007619385
H,0,6.2350471919,-0.9874023004,-2.611531762
H,0,7.2003983006,-2.1508672567,-1.7094930139
H,0,3.4293702742,-2.1553012656,-0.0109040198
H,0,2.9953564389,-2.9357128089,-2.2871899444
H,0,3.6873162852,-1.4557737128,-2.9648573839
H,0,5.1876292065,-3.5476728047,-1.3015027259
H,0,5.3789566175,-3.2781118476,-3.0311846771
H,0,4.4515707459,0.3657786568,-1.3980785714
N,0,4.1064820538,0.4780882198,0.6335919803
C,0,3.9258903992,-0.2879015461,1.8660519545
H,0,4.8448065571,-0.799213359,2.1908080871
H,0,3.6162579703,0.4008468193,2.6565163402
H,0,3.1243682005,-1.0203835117,1.743717318
C,0,5.0227114432,1.5932046301,0.8505522093
H,0,5.2121019462,2.1077465588,-0.097093715
H,0,4.5601356959,2.3047278117,1.5424526737
H,0,5.9896496616,1.2863332854,1.2762618678
N,0,2.0995632207,-0.83976562,-0.8806120132
H,0,2.0221669646,0.0728461581,-1.3229499372
C,0,0.9517222075,-1.4453762087,-0.5150273302
S,0,0.9186827392,-2.9868349115,0.1746792481
N,0,-0.1376961167,-0.6450087537,-0.7367379445
H,0,0.0845640037,0.3016134439,-1.0444403665
C,0,-1.5153631615,-0.8664852724,-0.6320943801
C,0,-2.3122961157,0.2888744064,-0.726579016
C,0,-2.1332679695,-2.1064419527,-0.4725766865
C,0,-3.688434386,0.1926858249,-0.6406712821
H,0,-1.8367410918,1.2604018729,-0.8222956018
C,0,-3.5247317711,-2.1671222846,-0.3835655189
H,0,-1.5441661062,-3.0108054262,-0.4105808751
C,0,-4.3213424064,-1.0374437818,-0.4606317895
H,0,-5.4010033216,-1.1058025672,-0.3863641705
C,0,-4.5429090862,1.4235711666,-0.7338282711
C,0,-4.1491424595,-3.5159995252,-0.1610913494
F,0,-3.8177328939,2.5438795488,-0.857267358
F,0,-5.3702752697,1.3684253386,-1.7916386077
F,0,-5.3210237071,1.5645857801,0.3504154546
F,0,-5.4753626574,-3.49755844,-0.3607626891
F,0,-3.9461943571,-3.9556506622,1.0905453112
F,0,-3.6318165449,-4.4424792959,-0.9823299113
C,0,0.5064316821,1.2266973617,-3.934887374
H,0,1.1767332368,0.3639366305,-3.8700505203
H,0,-0.4997565118,0.907305984,-3.6492974201
H,0,0.4755829483,1.5611925583,-4.9746629317
C,0,-0.7847153676,6.1290921368,-0.552970566
C,0,-0.215597059,7.4014081175,-0.6972215746
C,0,-2.1691959515,6.0033967725,-0.3679525311
C,0,-1.0222396917,8.5320227713,-0.6541083012
H,0,0.8562489067,7.4926258467,-0.8392330978
C,0,-2.9677157159,7.1398726461,-0.3289642487
H,0,-2.6021723579,5.0144208972,-0.2577324561
C,0,-2.3977033157,8.4035631468,-0.4710915597
H,0,-0.5767406686,9.5156214505,-0.7637012349
H,0,-4.0386390352,7.0388398931,-0.1858952082
H,0,-3.0254429965,9.2885213726,-0.4390977178
--------------------------------------------------------------------
```

TS
Opt @ M06-2X/6-31G(d,p) intoluene (SMD model)
SCF Done: E(RM062X) = -2723.83072714 a.u.
Zero-point correction=0.725726 Hartree/Particle
Sum of electronic and thermal Free Energies=-2723.187893 a.u.
Imaginary Frequency = -906.9413 cm^{-1}

C,0,-0.6646684197,4.5253226103,-0.6739728841
C,0,0.2095818025,3.701371231,-0.5170086354
C,0,1.3139378654,2.7878556995,-0.4389396259
H,0,2.1816716133,3.0950718413,-1.0192061057
N,0,1.5564038524,1.9259002934,0.5241948377
H,0,2.9170547444,0.935201519,0.6207639662
O,0,0.7801016038,1.6171615969,-2.0372187431
C,0,0.0023118092,2.1153856693,-3.0991462467
H,0,-0.9303060057,2.5691523076,-2.7265425592
H,0,0.5621703498,2.9159832687,-3.6043870331
C,0,0.4982395302,1.5554766204,1.3588982003
O,0,-0.6493901369,1.9397816127,1.294585299
O,0,0.9664645972,0.6958426086,2.2789132139
C,0,0.060166418,0.1539336639,3.2925223616
C,0,-0.451674147,1.2830547205,4.1817692352
C,0,-1.0695807913,-0.6342802155,2.6398307305
C,0,0.9538064539,-0.7868130038,4.0910541123
H,0,0.3865122217,1.8694806185,4.5718743658
H,0,-1.1265852997,1.943726588,3.6371224814
H,0,-0.9914876816,0.8549765998,5.0316506196
H,0,-0.6522077283,-1.3768791161,1.9523261189
H,0,-1.6272087459,-1.1634434314,3.4191362733
H,0,-1.7559190281,0.0181760326,2.0991216861
H,0,0.3711507951,-1.2552023117,4.8887367847
H,0,1.350642755,-1.5766235156,3.446015262
H,0,1.7843914121,-0.2420021865,4.5518313615
C,0,6.4717648091,-0.9418448431,-1.6438586535
C,0,5.9320882285,-0.4736872454,-0.2885562598
C,0,4.4781726111,-0.024959259,-0.4323407065
C,0,3.5918614249,-1.1452178942,-0.9996327832
C,0,4.1533703114,-1.6180415715,-2.346487682
C,0,5.6101530595,-2.0623476813,-2.2240389321
H,0,5.9965328574,-1.3040013758,0.4250183547
H,0,6.551561048,0.342555263,0.0952396804
H,0,6.4850784596,-0.0932329011,-2.3404384982
H,0,7.5081192567,-1.2714004711,-1.5251932689
H,0,3.578062912,-1.9962209158,-0.3121872331
H,0,3.5183125481,-2.431633368,-2.7086511457
H,0,4.0730777035,-0.7958022587,-3.0715062156
H,0,5.6672294135,-2.9414823723,-1.5691792841
H,0,5.993389221,-2.3696778482,-3.2016839995
H,0,4.436568591,0.8256806207,-1.1285729173
N,0,3.8911827176,0.5177046789,0.8465330971
C,0,3.6935800408,-0.5043739865,1.9079002802
H,0,4.6395223421,-1.007792602,2.1136470855
H,0,3.3420532842,0.0116039084,2.7992582958
H,0,2.9289014817,-1.2147755213,1.5881108504
C,0,4.6462008919,1.6806181132,1.3725280575
H,0,4.8735398614,2.3645024603,0.5525770323
H,0,4.0099796518,2.1865353477,2.0998454798
H,0,5.5685779636,1.349483558,1.8509641767
N,0,2.2420399473,-0.6572957063,-1.1242294543
H,0,2.1082900449,0.1734277407,-1.6987928884
C,0,1.0881805501,-1.245329816,-0.6782498107
S,0,1.14008068,-2.7392224101,0.1528458439
N,0,0.0258319975,-0.4747468202,-0.9483628688
H,0,0.3114600629,0.642053774,-1.5079139222
C,0,-1.3254313158,-0.7440943848,-0.7798059177
C,0,-2.1647802837,0.384674026,-0.8261497658
C,0,-1.9240447915,-2.006316659,-0.6449606216
C,0,-3.54095086,0.2523057698,-0.7317801075
H,0,-1.7141270381,1.3702354215,-0.9095692332
C,0,-3.3086355695,-2.1069891483,-0.5423027424
H,0,-1.3126663523,-2.898315474,-0.6236337949
C,0,-4.1405330481,-0.9948558401,-0.5828279355
H,0,-5.2171436807,-1.0946644966,-0.5141180931
C,0,-4.3804515487,1.4948347556,-0.7320525387
C,0,-3.9101393756,-3.4680503123,-0.3425736608
F,0,-3.9041471208,2.4239264965,-1.5764853653
F,0,-5.6522535847,1.2481109657,-1.0821935328
F,0,-4.4219768972,2.0723219449,0.4851159201
F,0,-5.1945112142,-3.5132876412,-0.7325434652
F,0,-3.8863423178,-3.8411024747,0.9486906703
F,0,-3.2517545522,-4.4147119253,-1.0282302167
C,0,-0.3305618212,1.0091089919,-4.0914337741
H,0,-0.9424879342,0.2353525776,-3.617090027
H,0,-0.8892728782,1.4087972009,-4.9431308331
H,0,0.585493283,0.53999101,-4.4632070597
C,0,-1.7891627935,5.4063019427,-0.7609278057

C,0,-2.980754166,5.0364896031,-0.1191185819
C,0,-1.7243425365,6.6095277601,-1.4760364129
C,0,-4.0940338196,5.8623953817,-0.2005183592
H,0,-3.0182609132,4.0991769638,0.4248598897
C,0,-2.8416482841,7.4327149525,-1.5446249016
H,0,-0.7992784954,6.8870954002,-1.9706872703
C,0,-4.0255638048,7.060441774,-0.9096801999
H,0,-5.0171703106,5.5675864074,0.2880703702
H,0,-2.7910137908,8.3661442288,-2.0958870524
H,0,-4.8968124664,7.7050682332,-0.9701042385

INT-1
Opt @ M06-2X/6-31G(d,p) intoluene (SMD model)
SCF Done: E(RM062X) = -2723.84118782 a.u.
Zero-point correction=0.730112 Hartree/Particle
Sum of electronic and thermal Free Energies=-2723.194753 a.u.

C,0,-0.9803662587,4.3138045777,-0.6139577332
C,0,-0.0318024098,3.6375509427,-0.2715057474
C,0,1.1803513262,2.927904213,-0.0480337765
H,0,2.0046429968,3.2482736557,-0.6843950288
N,0,1.4370041921,1.9799965198,0.7832487306
H,0,2.9056797009,0.8572663805,0.7282734903
O,0,0.7744291305,1.8267426048,-2.4540644412
C,0,-0.1566708017,2.3841734777,-3.355952004
H,0,-1.0971457811,2.6283552883,-2.8390908376
H,0,-0.4051523201,1.6688811635,-4.1545051546
C,0,0.3927972335,1.5144658561,1.6295566246
O,0,-0.7095739406,1.9980803618,1.7261233296
O,0,0.8673271016,0.4885229807,2.3321775761
C,0,0.0139861359,-0.170312714,3.332535758
C,0,-0.3155769127,0.8226025145,4.4418022528
C,0,-1.2330691201,-0.7478963014,2.6753191036
C,0,0.9004312935,-1.2929447664,3.8538826518
H,0,0.6017636804,1.2640598838,4.844508097
H,0,-0.9694575245,1.6189102579,4.0846204704
H,0,-0.8232319156,0.2940704171,5.2539508326
H,0,-0.9468318621,-1.3885597762,1.834952404
H,0,-1.7630573274,-1.3604393868,3.4114271488
H,0,-1.9066517491,0.0350707748,2.3259371732
H,0,0.3444083254,-1.8912884567,4.5804821035
H,0,1.2048168549,-1.9401020441,3.0257081698
H,0,1.7880541584,-0.8927548957,4.3545080527
C,0,6.4643252683,-0.6848800474,-1.684980274
C,0,5.920575068,-0.3832455253,-0.2836693541
C,0,4.4822222012,0.1131110139,-0.3938184632
C,0,3.5711631311,-0.9094548888,-1.0913410054
C,0,4.1350417289,-1.2242031899,-2.4825536147
C,0,5.5843601872,-1.704622865,-2.4080467319
H,0,5.9512499447,-1.3025076311,0.313158893
H,0,6.5536042701,0.359706807,0.2123826343
H,0,6.4996072439,0.2456350618,-2.2666161146
H,0,7.4925256402,-1.0493229093,-1.6040493878
H,0,3.5411385561,-1.835727005,-0.5099152073
H,0,3.4909240705,-1.9781289514,-2.9445920485
H,0,4.0737522346,-0.3189392762,-3.1034971327
H,0,5.620801139,-2.6603850125,-1.8697233761
H,0,5.9707705156,-1.892390458,-3.4142071868
H,0,4.4665090746,1.0395513382,-0.9855216201
N,0,3.8833937227,0.5189912674,0.9318459724
C,0,3.7601802023,-0.586226978,1.9211050845
H,0,4.7500211188,-0.9902315542,2.1367811941
H,0,3.3229670375,-0.165614225,2.8250709003
H,0,3.0882502676,-1.3513779084,1.5241893385
C,0,4.5676952648,1.688641064,1.5358891977
H,0,4.7150298079,2.4540492665,0.7723452987
H,0,3.9274185011,2.0812161041,2.3266900157
H,0,5.5280759766,1.3824242357,1.9516876337
N,0,2.2294381571,-0.3891559946,-1.1481789082
H,0,2.0781528092,0.3794680684,-1.7970738931
C,0,1.0667615099,-1.0265845365,-0.7439513257
S,0,1.173014219,-2.5350429722,0.0893689784
N,0,-0.0057546934,-0.3069304623,-1.022988367
H,0,0.3305850253,1.093127481,-1.9324746962
C,0,-1.3143584986,-0.7207916314,-0.8302730115
C,0,-2.2477991645,0.2698906748,-0.4864794208
C,0,-1.7933117992,-2.0298673928,-1.0261094488
C,0,-3.5843111949,-0.0499506443,-0.2796232776
H,0,-1.8941109512,1.2875441495,-0.3375195947
C,0,-3.1375857447,-2.3201719614,-0.8305152675
H,0,-1.1081450735,-2.8121710875,-1.3258037865
C,0,-4.0544496616,-1.3473302782,-0.4410782286
H,0,-5.0972406067,-1.5914109474,-0.2783946154
C,0,-4.512005601,1.049456421,0.1366199395
C,0,-3.5997358741,-3.7388814856,-0.9917680862

F,0,-4.690428503,1.9639713994,-0.8349652939
F,0,-5.7281788098,0.5992199985,0.4767374917
F,0,-4.029646868,1.7255338344,1.1993783313
F,0,-4.9077345357,-3.8108942955,-1.2939892971
F,0,-3.4271193473,-4.458061799,0.1309420957
F,0,-2.9322883232,-4.3799311961,-1.9631531847
C,0,-2.1516126108,5.042643698,-0.9826523854
C,0,-2.0839001809,6.0560962272,-1.95119127
C,0,-3.3813766183,4.715289558,-0.392376599
C,0,-3.2373940637,6.736135604,-2.3181071614
H,0,-1.1283339829,6.2971099353,-2.4058065615
C,0,-4.5303887677,5.3952263879,-0.77493932
H,0,-3.4223972884,3.9241315242,0.3478999775
C,0,-4.4592767744,6.4054411776,-1.732842949
H,0,-3.1862392737,7.5215896207,-3.0648988631
H,0,-5.4830905831,5.1315265827,-0.3276688961
H,0,-5.3596729155,6.9349320465,-2.0281716769
C,0,0.4367628188,3.6445800905,-3.9529488877
H,0,1.3607116391,3.4152542757,-4.4921677318
H,0,-0.2631740261,4.1172367466,-4.64799981
H,0,0.6729124575,4.3575186908,-3.1563315404

INT-2
Opt @ M06-2X/6-31G(d,p) intoluene (SMD model)
SCF Done: E(RM062X) = -2723.85670236 a.u.
Zero-point correction=0.729070 Hartree/Particle
Sum of electronic and thermal Free Energies=-2723.214304 a.u.

C,0,1.7578950366,4.4019183704,-0.5814029625
C,0,0.8085724902,3.6797625391,-0.7959571824
C,0,-0.2967601099,2.7987213897,-1.0415788745
H,0,-0.6785177779,2.7035419446,-2.0591789734
N,0,-0.85618558,2.0773697052,-0.1488191364
O,0,-1.7829915519,0.6930967347,-2.6778300735
C,0,-1.4286262973,-0.1836421093,-3.7340202708
H,0,-2.1673174903,-0.126291389,-4.5482388488
H,0,-1.4044806984,-1.2271375023,-3.3839377688
C,0,-0.401640874,2.1618749163,1.1928451524
O,0,-0.8819881576,2.9258101875,1.9912758273
O,0,0.5232207499,1.2328124216,1.3993017634
C,0,1.1357432077,1.0777105141,2.7284085996
C,0,1.8823611625,2.3577156587,3.0823481552
C,0,0.0655242417,0.7101462639,3.7490193942
C,0,2.1079720678,-0.0767604915,2.5319068656
H,0,2.5965550035,2.6065193026,2.2911668892
H,0,1.1971737255,3.1949797848,3.2221746976
H,0,2.4399216414,2.2029048192,4.0105467838
H,0,-0.4965524311,-0.1638861928,3.4056849072
H,0,0.5499992842,0.4511198606,4.694897331
H,0,-0.6232616097,1.5364501313,3.9252792332
H,0,2.6129992952,-0.2911904051,3.4777417405
H,0,1.5787739402,-0.9787440609,2.2102871886
H,0,2.864747265,0.1776718825,1.7854609198
C,0,-6.2563396051,0.1764771572,1.4787554487
C,0,-6.0635360189,-0.17483088,0.0002470323
C,0,-4.6127300785,0.0768668504,-0.4345645923
C,0,-3.6636785963,-0.7571618221,0.4425052547
C,0,-3.8432969563,-0.3988963197,1.9218190081
C,0,-5.2907043639,-0.6097666963,2.3653660613
H,0,-6.3164622974,-1.2311446648,-0.159041117
H,0,-6.7468233813,0.4186480672,-0.6158005283
H,0,-6.080855365,1.2519610339,1.6166618533
H,0,-7.2925747742,-0.0158121688,1.7745686259
H,0,-3.877161282,-1.8251260255,0.3212487172
H,0,-3.1588071716,-1.0182247542,2.5091544985
H,0,-3.5483114127,0.6499845744,2.0670290561
H,0,-5.532046747,-1.6792257004,2.3050451648
H,0,-5.4078505197,-0.317480279,3.4134584422
H,0,-4.3830603741,1.1370807378,-0.2376793252
N,0,-4.332711142,-0.1171153758,-1.8715074314
C,0,-4.457230313,-1.4988121734,-2.3311800333
H,0,-5.4763697289,-1.8993861959,-2.2218375572
H,0,-4.1958199483,-1.5389475059,-3.3927756155
H,0,-3.7627423337,-2.1482644384,-1.7921532549
C,0,-5.1125810934,0.7829768334,-2.7157829514
H,0,-5.0351358521,1.8038860573,-2.3308511267
H,0,-4.7019748826,0.7679720675,-3.7303378473
H,0,-6.1763142157,0.5100036387,-2.7772539635
N,0,-2.3051903137,-0.5271446009,-0.0049648384
H,0,-2.0789236788,0.4258416712,-0.2898935388
C,0,-1.3047160523,-1.4209257273,-0.0386730371
S,0,-1.4049967397,-2.9464047587,0.6755932105
N,0,-0.2341504322,-0.9335842926,-0.7491777847
H,0,-2.6740123302,0.4133672567,-2.3371245366
C,0,1.1222171341,-1.2629501935,-0.6879157223

```
C,0,2.0232692784,-0.2165901119,-0.9285059349
C,0,1.6251011496,-2.5413841169,-0.4308906135
C,0,3.3893609796,-0.4438906502,-0.8938921453
H,0,1.6465576661,0.7869231847,-1.1040390423
C,0,3.0046513547,-2.7351271002,-0.3857838071
H,0,0.9479943135,-3.3695298849,-0.2678359925
C,0,3.906289021,-1.7044059194,-0.6096646979
H,0,4.9751537213,-1.8762523709,-0.5737236638
C,0,4.3073537894,0.7326998649,-1.0368917545
C,0,3.5013102329,-4.1182887199,-0.070960628
F,0,4.4008999731,1.4271346118,0.1147683597
F,0,5.5507122311,0.3721213625,-1.3754313647
F,0,3.8696430482,1.6028043888,-1.9633177118
F,0,4.8314358841,-4.2200638947,-0.207076928
F,0,2.9447049073,-5.0378883518,-0.873875452
F,0,3.2029490532,-4.4744063653,1.1874113255
H,0,-0.4371921641,-0.0618550523,-1.2399454387
C,0,2.8679151076,5.2676744533,-0.3100260403
C,0,2.6418437808,6.5554396705,0.1951542864
C,0,4.176910734,4.8224253736,-0.5396642842
C,0,3.7205451254,7.3889975976,0.461891621
H,0,1.6251384119,6.889350068,0.3726057678
C,0,5.2470560874,5.6653425419,-0.2665801482
H,0,4.3394476269,3.8229222893,-0.925700188
C,0,5.0221046979,6.9462363124,0.2330378598
H,0,3.5452062976,8.3866801366,0.8508168441
H,0,6.2601539426,5.3190314492,-0.442481119
H,0,5.8617729891,7.6003202039,0.4453281817
C,0,-0.0632189443,0.2151946362,-4.2547435137
H,0,0.6901591681,0.1382551391,-3.4629883004
H,0,0.24539722,-0.4361232381,-5.0767648361
H,0,-0.0794916008,1.247382994,-4.6163149207
-------------------------------------------------------------------
```

Supplementary References

1. Zhao, Y. & Truhlar, D. G. The M06 suite of density functionals for main group thermochemistry, kinetics, noncovalent interactions, excited states, and transition elements: two new functionals and systematic testing of four M06 functionals and twelve other functionals. *Theor. Chem. Acc.* **120,** 215–241 (2008).
2. Zhao, Y. & Truhlar, D. G. Density functionals with broad applicability in chemistry. *Acc. Chem. Res.* **41,** 157–167 (2008).
3. Marenich, A. V., Cramer, C. J. & Truhlar, D. G. Universal solvation model based on solute electron density and on a continuum model of the solvent defined by the bulk dielectric constant and atomic surface tensions. *J. Phys. Chem. B* **113,** 6378–6396 (2009).
4. Frisch, M. J. *et al.* Gaussian 09, Revision D.01 (Gaussian, 2013).
5. Legault, C. Y. CYLView, 1.0b. http://www.cylview.org (2009).
